# Supplementary material for: The Facile Solid-Phase Synthesis of Thiazolo-Pyrimidinone Derivatives
Source: Molecules. 2025 Jan 20;30(2):430. doi: 10.3390/molecules30020430 (PMC11767942; doi:10.3390/molecules30020430)

Supporting Information

The facile Solid-phase synthesis of Thiazolo-pyrimidinone derivatives

Shuanghui Hua†, Jimin Moon†, Taeho Lee*

College of Pharmacy, Research Institute of Pharmaceutical Sciences, Kyungpook National University, 80 Daehak-ro, Buk-gu, Daegu 702-701, Korea.

* = Corresoponding author. E-mail : tlee@knu.ac.kr

† = These authors contributed equally to this work.

Contents

1. **Experimental section……………………………………………………………………S2**
2. **Diversity elements…………………………………………………………………….S3**
3. **General synthesis procedures…………………………………………………….…S4**
4. **NMR Spectra of all compounds…………………………………………………..….S21**

1. Experimental

1.1 General information

All the chemicals were reagent grade and used as purchased. The Merrifield resin (loading capacity 1.29 mmol/g, 100-200 mesh) was phurchased from BeadTech (Seoul, Korea). The reactions were monitored by TLC analysis using Merck silica gel 60 F-254 thin layer plates (Merck, Darmstadt, Germany). Flash column chromatography was carried out on Merck silica gel 60 (230–400 mesh). The crude products, which were derived from the solid support, were purified by parallel chromatography using CombiFlash (Isco, Lincoln, NE, USA). The ^{1}H NMR and ^{13}C NMR spectra were recorded in d units relative to the deuterated solvent ($CDCl_3$, DMSO-d_6, etc.) as an internal reference by the Bruker 500 MHz NMR instrument (Bruker, Billerca, MA, USA). High-performance liquid chromatography (HPLC) system, specifically the Ultimate 3000, coupled with the Q-Exactive Focus quadrupole-Orbitrap MS (Thermo Fisher Scientific, Mass Spectrometry Based Convergence Research Institute, Kyungpook National University, Bremen, Germany).Mass Spectrometry Based Convergence Research Institute, Kyungpook National University The solid-phase synthesis was monitored by FT-IR using JASCO FT-IR 4600

1.2 Diversity elements

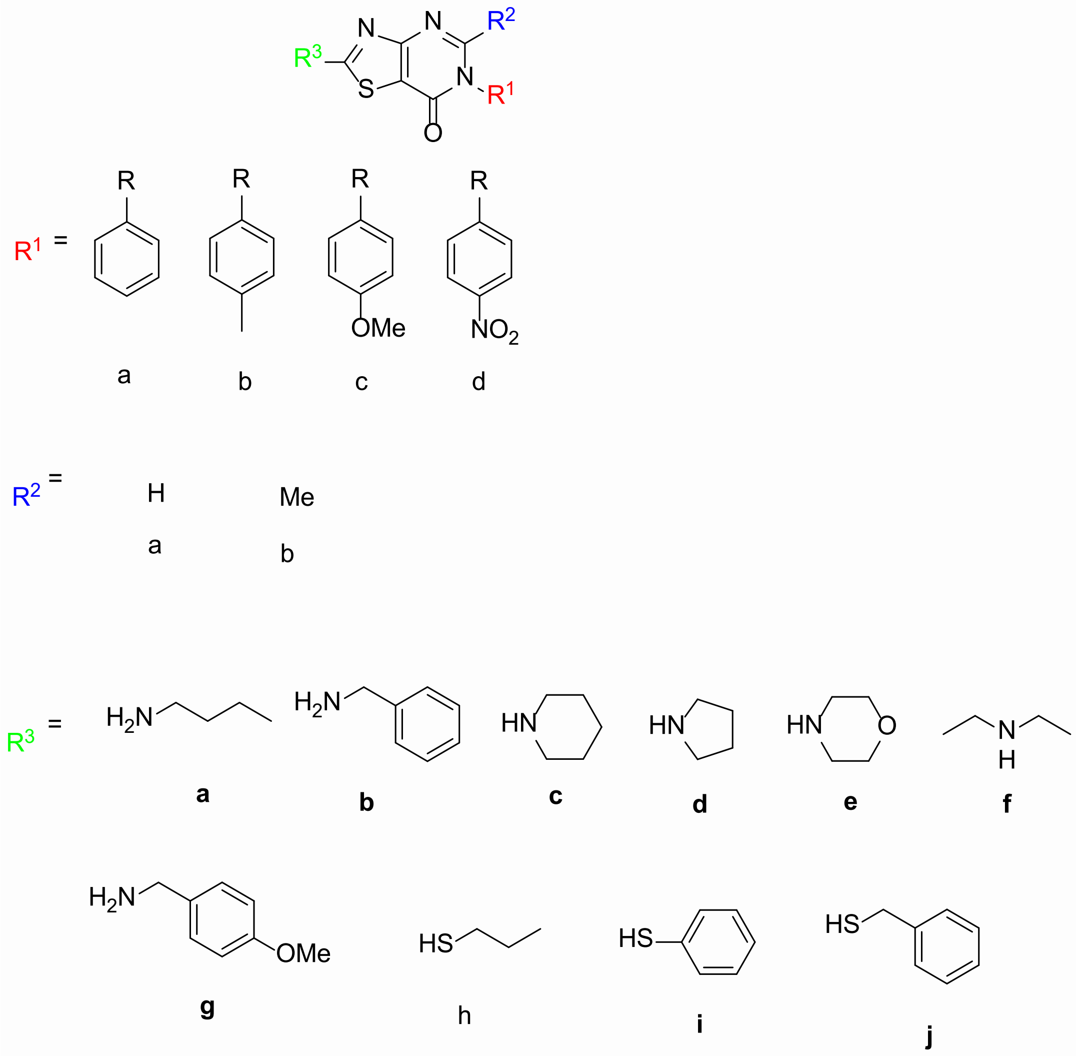

1.3 General procedure

Synthesis of 4-amino-2-(methylthio)-*N*-phenylthiazole-5-carboxamide **(7a)**

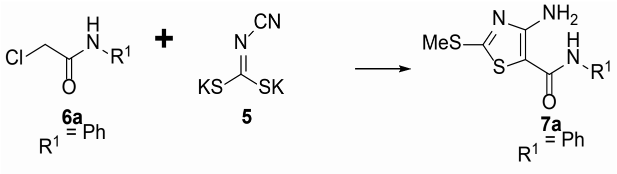

To a solution of **6a** (73.0 mg, 0.38 mmol) in H_2O (0.5 mL) was added solution of **5** (50 mg, 0.25 mmol) dissolved in acetone (2.5 mL) by dropwising at room temperature. After the addition was completed, the reaction mixture was stirred at room temperature for 1 h. The mixture was added LiOH (6.10 mg, 0.25 mmol) at room temperature and reaction mixture was heated under reflux 60 ℃ for 2 h. After cooling, CH_3I (15.85 μL, 0.25 mmol) in acetone was added dropwise. The mixture was stirred for 1 h at room temperature. The crude product was recrystallized from cold H_2O to give compound **7a**(98 %). As a solid; ^{1}H NMR (500 MHz, $CDCl_3$) δ 7.53 – 7.47 (m, 2H), 7.38 – 7.31 (m, 2H), 7.15 – 7.07 (m, 1H), 6.78 (s, 1H), 6.12 (s, 2H), 2.67 (s, 3H).^{13}C NMR (126 MHz, $CDCl_3$) δ 169.67, 162.26, 137.99, 129.08, 124.40, 120.61, 93.61, 16.19. HRMS(ESI) *m/z* $[M+H]^+$Calcd for $C_{11}H_{12}N_3OS_2^+$ 266.0416; Found 266.0418 .

Synthesis of 2-(methylthio)-6-phenylthiazolo[4,5-*d*]pyrimidin-7(6*H*)-one **(8aa)**

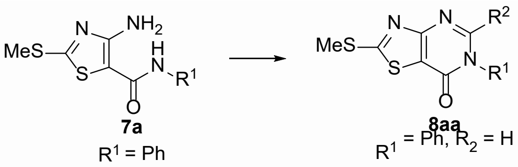

To a solution of **7a** (50 mg, 0.17 mmol) in EtOH(1 mL) was added triethylorthoformate (264.0 μL, 3.39 mmol) and added CSA (7.9 mg, 0.03 mmol), the mixture was stirred 60 ºC for 2 h. After completion of reaction(monitored by TLC), and then diluted with CH_2Cl_2, washed with brine, dried over $MgSO_4$. The solvent was removed, and the residue was purified by flash silica gel column chromatography (hexane/EtOAc, 1:1) to give **8aa** (37.60 mg, 79 %), ^{1}H NMR (500 MHz, $CDCl_3$) δ 8.24 (s, 1H), 7.64 – 7.51 (m, 3H), 7.47 – 7.40 (m, 2H), 2.86 (s, 3H). ^{13}C NMR (126 MHz, $CDCl_3$) δ 177.52, 165.85, 156.12, 149.27, 136.60, 129.76, 129.62, 127.05, 117.66, 16.36. HRMS(ESI) *m/z* $[M+H]^+$Calcd for $C_{12}H_{10}N_3OS_2^+$276.0260 ; Found 276.0281.

Synthesis of 2-(methylsulfonyl)-6-phenylthiazolo[4,5-*d*]pyrimidin-7(6*H*)-one **(9aa)**

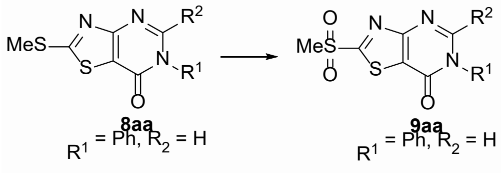

To a solution 2-(methylthio)-6-phenylthiazolo[4,5-*d*]pyrimidin-7(6*H*)-one **8aa** (100 mg, 0.26 mmol) in CH_2Cl_2 was added *m*CPBA(132 mg, 0.57 mmol) at room temperature for overnight. After completion of reaction(monitored by TLC), the crude was quenched with $NaHCO_3$ solution and extracted with CH_2Cl_2. The combined organic layer dried over $MgSO_4$. The solvent was removed, and the residue was purified by flash silica gel column chromatography (hexane : EtOAc : CH_2Cl_2) to give **9aa** (91 mg, 85%) as a solid; ^{1}H NMR (500 MHz,

$CDCl_3$) δ 8.40 (s, 1H), 7.65 – 7.58 (m, 3H), 7.46 (dd, J = 8.1, 1.5 Hz, 2H), 3.50 (s, 3H). ^{13}C NMR (126 MHz, $CDCl_3$) δ 173.63, 165.55, 155.69, 150.03, 136.60, 129.76, 129.70, 127.52, 117.66, 42.07. HRMS(ESI) *m/z* $[M+H]^+$Calcd for $C_{12}H_{10}N_3O_3S_2^+$ 308.0158; Found 308.0160.

Synthesis of 2-(butylamino)-6-phenylthiazolo[4,5-*d*]pyrimidin-7(6*H*)-one **(1aaa)**

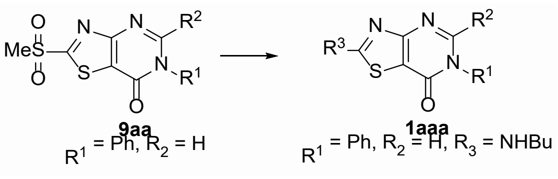

To a solution of 2-(methylsulfonyl)-6-phenylthiazolo[4,5-*d*]pyrimidin-7(6*H*)-one **9aa** (100 mg, 0.32 mmol) in CH_2Cl_2 was added butylamine (0.072 mL, 0.72 mmol) and Et_3N (0.1 mL, 0.72 mmol) at room temperature for 5 h. After completion of reaction(monitored by TLC), the reaction mixture was extracted with CH_2Cl_2. The combined organic layer was dried over $MgSO_4$. The solvent was removed and the residue was purified by flash silica gel column chromatography (hexane : EtOAc : CH_2Cl_2) to give **1aaa** (58 mg, 60%) as a yellow solid HRMS(ESI) *m/z* $[M+H]^+$Calcd for $C_{15}H_{17}N_4OS^+$ 301.1118; Found 301.1117

Preparation of 4-amino-*N*-(substituted)thiazole-5-carboxamide resin **(12a)**. (R_1 = Ph)

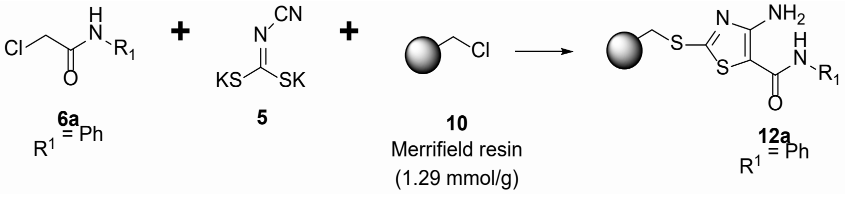

To a solution of **6a** (5.6 g, 29 mmol) in H_2O (20 mL) was added solution of **5** (2.9 g, 17.4 mmol) dissolved in acetone (100 mL) by dropwise at room temperature. After the addition was completed, the reaction mixture was stirred at room temperature for 1 h. The mixture was added LiOH (556 mg, 23.2 mmol) at room temperature and reaction mixture was heat at reflux for 1 h. After cooling, reaction solvent evaporated and was concentrated under reduced pressure and dried in a vacuum oven to give **7-Int II**. Merrifield resin **10**(4.5 g, 5.8 mmol, 1.29 mmol/g) was treated with crude **7-Int II** in acetone (100 mL) at room temperature. The reaction mixture was shaken for 10 h at room temperature and then filtered, washed several times with H_2O, DMF, MeOH, and CH_2Cl_2, and dried in a vacuum oven to give 4-amino-*N*-(substituted)thiazole-5-carboxamide resin **12a** (6.3 g , 5.8 mmol)

Prepared of 2-(methylthio)-6-phenylthiazolo[4,5-*d*]pyrimidin-7(6*H*)-one resin **(13aa)**

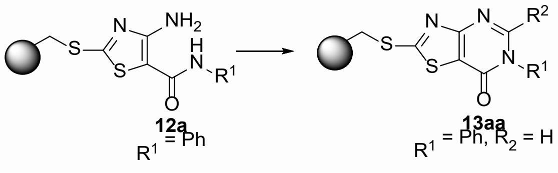

A mixture of resin **12a** (3.1 g, theoretically 2.85 mmol), triethylorthoformate (4.7 mL , 28.5 mmol) in DMF:EtOH (2:1, 40 mL) and added CSA (359 mg, 1.55 mmol) was shaken at 70 °C for 2 h, and then filtered, washed several

times with H_2O, DMF, MeOH, and CH_2Cl_2, and dried in a vacuum oven to give 2-(methylthio)-6-phenylthiazolo[4,5-*d*]pyrimidin-7(6*H*)-one resin **13aa** as a yellow solid.

Prepared of 2-(methylsulfonyl)-6-phenylthiazolo[4,5-*d*]pyrimidin-7(6*H*)-one resin **(14aa)**

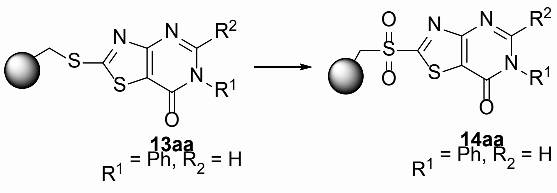

To a mixture of 2-(methylthio)-6-phenylthiazolo[4,5-d]pyrimidin-7(6*H*)-one resin **13aa** (3.1 g, 2.85 mmol,) in CH_2Cl_2 (100 ml) was added *m*CPBA (2.6 g, 11.4 mmol) at room temperature. The reaction resin mixture was shaken for overnight and then filtered, washed several times with H_2O, DMF, MeOH and CH_2Cl_2 and dried in a vacuum oven to give **14aa** (3.08 g) as a yellow solid;

Prepared of 2-(butylamino)-6-phenylthiazolo [4,5-*d*]pyrimidin-7(6*H*)-one**(1aaa)**

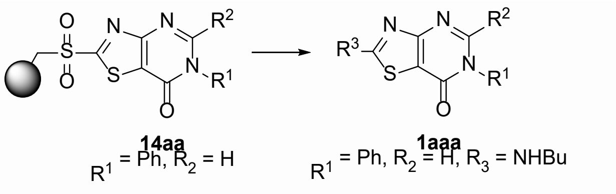

To a mixture of 2-(methylsulfonyl)-6-phenylthiazolo[4,5-*d*]pyrimidin-7(6*H*)-one resin **14aa** (0.33 g, 0.31 mmol) in CH_2Cl_2 (10 mL) was added butylamine (0.093 mL, 1.5 mmol) and Et_3N (0.13 mL, 1.5 mmol) at room temperature. The reaction mixture was shaken for overnight and then filtered, washed several times with MeOH and CH_2Cl_2. The organic solvent was removed, and the residue was purified by flash silica gel column chromatography (hexane : EtOAc) to give 1**aaa** (75 mg, 81%) as a yellow solid;

2-(butylamino)-6-phenylthiazolo [4,5-*d*]pyrimidin-7(6*H*)-one**(1aaa)**

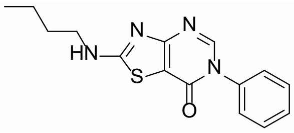

^{1}H NMR (500 MHz, $CDCl_3$) δ 8.21 (s, 1H), 7.59 – 7.50 (m, 3H), 7.44 – 7.38 (m, 2H), 3.42 – 3.36 (m, 2H), 1.88 (dd, J = 14.6, 7.3 Hz, 2H), 1.09 (t, J = 7.4 Hz, 3H). ^{13}C NMR (126 MHz, $CDCl_3$) δ 174.68, 166.40, 156.37, 149.57, 137.53, 130.14, 129.22, 127.09, 108.48, 77.48, 45.80, 31.14, 20.01, 14.11 ; HRMS(ESI) *m/z* $[M+H]^+$Calcd for $C_{15}H_{17}N_4OS^+$ 301.1118; Found:301.1117

2-(benzylamino)-6-phenylthiazolo[4,5-*d*]pyrimidin-7(6*H*)-one**(1aab)**

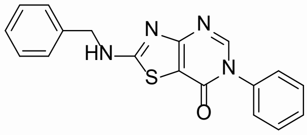

^{1}H NMR (500 MHz, $CDCl_3$) δ 8.16 (s, 1H), 7.61 – 7.50 (m, 3H), 7.43 (td, *J* = 3.5, 1.1 Hz, 6H), 6.30 (s, 1H), 4.69 (d, *J* = 5.0 Hz, 2H). ^{13}C NMR (126 MHz, $CDCl_3$) δ 165.77, 156.14, 148.66, 137.32, 135.82, 130.12, 129.61, 129.35, 129.27, 128.97, 128.69, 128.23, 127.99, 127.94, 127.59, 127.10, 49.90 ; HRMS(ESI) *m/z* $[M+H]^+$Calcd for $C_{18}H_{15}N_4OS^+$ 335.0961; Found: 335.0961

6-phenyl-2-(piperidin-1-yl)thiazolo[4,5-*d*]pyrimidin-7(6*H*)-one**(1aac)**

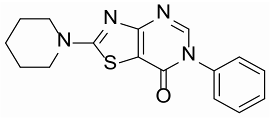

^{1}H NMR (500 MHz, $CDCl_3$) δ 8.11 (s, 1H), 7.51 (dd, *J* = 15.8, 9.7 Hz, 3H), 7.43 – 7.35 (m, 2H), 3.68 (s, 4H), 1.73 (s, 6H). ^{13}C NMR (126 MHz, $CDCl_3$) δ 173.41, 166.61, 156.14, 149.11, 137.33, 129.54, 127.14, 108.48, 77.30, 77.04, 76.79, 49.90, 25.25, 23.68; HRMS(ESI) *m/z* $[M+H]^+$Calcd for $C_{16}H_{17}N_4OS^+$ 313.1118; Found: 313.1116.

2-morpholino-6-phenylthiazolo[4,5-*d*]pyrimidin-7(6*H*)-one **(1aae)**

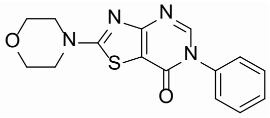

^{1}H NMR (500 MHz, $CDCl_3$) δ 8.14 (s, 1H), 7.57 – 7.46 (m, 3H), 7.42 – 7.38 (m, 2H), 3.87 – 3.83 (m, 4H), 3.75 – 3.61 (m, 4H). ^{13}C NMR (126 MHz, $CDCl_3$) δ 173.40, 167.50, 156.59, 149.26, 136.88, 129.61, 129.33, 127.11, 77.32, 77.07, 76.81, 66.08, 48.63, 48.27 ; HRMS(ESI) *m/z* $[M+H]^+$Calcd for $C_{15}H_{15}N_4O_2S^+$ 315.0910; Found: 315.0909.

2-(diethylamino)-6-phenylthiazolo[4,5-*d*]pyrimidin-7(6*H*)-one **(1aaf)**

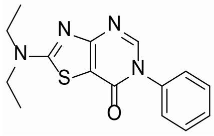

^{1}H NMR (500 MHz, $CDCl_3$) δ 8.11 (s, 1H), 7.51 (dt, *J* = 18.0, 7.1 Hz, 3H), 7.42 – 7.36 (m, 2H), 3.63 (s, 4H), 1.31 (t, *J* = 7.2 Hz, 6H). ^{13}C NMR (126 MHz, $CDCl_3$) δ 173.16, 167.93, 156.58, 149.32, 137.51, 129.54, 129.02, 127.14, 108.02, 77.30, 77.05, 76.79, 45.79, 12.32; HRMS(ESI) *m/z* $[M+H]^+$Calcd for $C_{15}H_{17}N_4OS^+$ 301.1118; Found: 301.1117.

2-((4-methoxybenzyl)amino)-6-phenylthiazolo[4,5-*d*]pyrimidin-7(6*H*)-one **(1aag)**

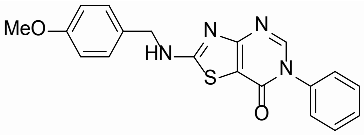

^{1}H NMR (500 MHz, $CDCl_3$) δ 8.11 (s, 1H), 7.53 (d, *J* = 7.6 Hz, 3H), 7.39 (t, *J* = 4.2 Hz, 2H), 7.32 (d, *J* = 8.7 Hz, 2H), 6.89 (d, *J* = 8.7 Hz, 2H), 6.76 (s,1H), 4.56 (d, *J* = 4.1 Hz, 2H), 3.81 (s, 3H). ^{13}C NMR (126 MHz, $CDCl_3$) δ 166.59, 160.03, 156.59, 149.57, 136.51, 129.24, 129.04, 128.80, 126.85, 114.15, 77.32, 77.07, 76.82, 55.22, 49.30.; HRMS(ESI) *m/z* $[M+H]^+$Calcd for $C_{19}H_{17}N_4O_2S+$ 365.1067; Found: 365.1068

6-phenyl-2-(propylthio)thiazolo[4,5-*d*]pyrimidin-7(6*H*)-one **(1aah)**

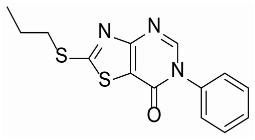

^{1}H NMR (500 MHz, CDCl3) δ 8.21 (s, 1H), 7.59 – 7.50 (m, 3H), 7.44 – 7.38 (m, 2H), 3.42 – 3.36 (m, 2H), 1.88 (dd, *J* = 14.6, 7.3 Hz, 2H), 1.09 (t, *J* = 7.4 Hz, 3H). ^{13}C NMR (126 MHz, CDCl3) δ 178.63, 166.16, 156.37, 149.18, 137.33, 129.79, 129.63, 127.06, 116.76, 77.33, 77.07, 76.82, 35.84, 22.47, 13.43 ; HRMS(ESI) *m/z* $[M+H]^+$Calcd for $C_{14}H_{14}N_3OS_2^+$ 304.0573; Found:304.0573

6-phenyl-2-(phenylthio)thiazolo[4,5-*d*]pyrimidin-7(6*H*)-one **(1aai)**

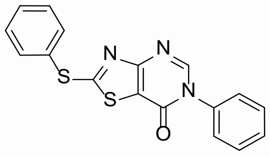

^{1}H NMR (500 MHz, CDCl3) δ 8.19 (s, 1H), 7.76 (d, *J* = 6.9 Hz, 2H), 7.54 (dd, *J* = 10.2, 7.4 Hz, 6H), 7.38 (s, 2H). ^{13}C NMR (126 MHz, CDCl3) δ 180.81, 166.62, 156.13, 149.56, 136.71, 135.90, 132.31, 130.63, 129.79, 128.35, 127.01,118.11, 77.32, 77.07, 76.81 ; HRMS(ESI) *m/z* $[M+H]^+$Calcd for $C_{17}H_{12}N_3OS_2^+$ 338.0416; Found:338.0417

2-(benzylthio)-6-phenylthiazolo[4,5-*d*]pyrimidin-7(6*H*)-one **(1aaj)**

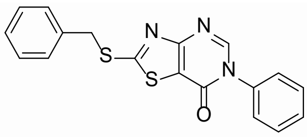

^{1}H NMR (500 MHz, CDCl3) δ 8.22 (s, 1H), 7.54 (dd, *J* = 13.7, 7.3 Hz, 3H), 7.49 – 7.44 (m, 2H), 7.41 (dd, *J* = 8.2, 1.3 Hz, 2H), 7.38 – 7.28 (m, 3H), 4.65 (s, 2H). ^{13}C NMR (126 MHz, CDCl3) δ 176.44, 166.16, 156.14, 149.79, 137.08, 135.15, 129.81, 129.28, 129.03, 128.89, 128.36, 127.05, 117.66, 77.33, 77.08, 76.82, 38.01 ; HRMS(ESI) *m/z* $[M+H]^+$Calcd for $C_{18}H_{14}N_3OS_2^+$352.0573; Found:352.0573

2-(butylamino)-5-methyl-6-phenylthiazolo[4,5-*d*]pyrimidin-7(6*H*)-one**(1aba)**

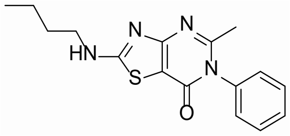

^{1}H NMR (500 MHz, CDCl3) δ 7.58 – 7.43 (m, 3H), 7.24 (dd, *J* = 4.7, 3.2 Hz, 2H), 6.22 (s, 1H), 3.43 (d, *J* = 6.0 Hz, 2H), 2.22 (s, H), 1.77 – 1.67 (m, 2H), 1.44 (dd, *J* = 15.1, 7.5 Hz, 2H), 0.97 (t, *J* = 7.4 Hz, 3H). ^{13}C NMR (126 MHz, CDCl3) δ 165.93, 158.30, 137.31, 130.01, 129.03, 128.01, 105.86, 77.30, 77.05, 76.79, 45.11, 30.93, 24.37, 20.04, 13.74 ; HRMS(ESI) *m/z* $[M+H]^+$Calcd for $C_{16}H_{19}N_4OS^+$ 315.1274; Found:315.1273

2-(benzylamino)-5-methyl-6-phenylthiazolo[4,5-*d*]pyrimidin-7(6*H*)-one **(1abb)**

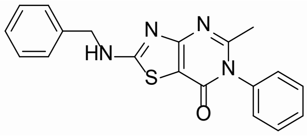

^{1}H NMR (500 MHz, $CDCl_3$) δ 7.58 – 7.48 (m, 3H), 7.41 – 7.37 (m, 5H), 7.25 – 7.21 (m, 2H), 5.94 (s, 1H), 4.66 (d, *J* = 5.6 Hz, 2H), 2.23 (s, 3H). ^{13}C NMR (126 MHz, $CDCl_3$) δ 165.56, 157.40, 137.55, 130.02, 128.89, 127.98, 127.95, 77.30, 77.05, 76.79, 49.68, 24.22; HRMS(ESI) *m/z* $[M+H]^+$Calcd for $C_{19}H_{17}N_4OS^+$ 349.1118; Found:349.1118

5-methyl-6-phenyl-2-(piperidin-1-yl)thiazolo[4,5-*d*]pyrimidin-7(6*H*)-one **(1abc)**

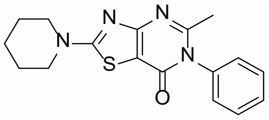

^{1}H NMR (500 MHz, $CDCl_3$) δ 7.52 (dd, *J* = 16.2, 10.1 Hz, 3H), 7.26 – 7.23 (m, 2H), 3.67 (s, 4H), 2.21 (s, 3H), 1.72 (s, 6H) ^{13}C NMR (126 MHz, $CDCl_3$) δ 173.02, 166.19, 157.87, 157.67, 137.70, 129.92, 129.28, 128.06, 105.76, 77.40, 77.15, 76.90, 49.61, 25.21, 24.19, 24.04; HRMS(ESI) *m/z* $[M+H]^+$Calcd for $C_{17}H_{19}N_4OS^+$ 327.1274; Found:327.1273

5-methyl-6-phenyl-2-(pyrrolidin-1-yl)thiazolo[4,5-*d*]pyrimidin-7(6*H*)-one **(1abd)**

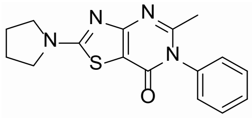

^{1}H NMR (500 MHz, CDCl3) δ 7.48 (s, 3H), 7.19 – 7.16 (m, 1H), 3.53 (d, J = 127.2 Hz, 4H), 2.15 (s, 3H), 2.04 (s, 4H). ^{13}C NMR (126 MHz, $CDCl_3$) δ 169.44, 167.06, 157.40, 137.79, 129.96, 129.03, 128.08, 77.30, 77.04, 76.79, 49.75, 25.26, 24.57; HRMS(ESI) *m/z* $[M+H]^+$Calcd for $C_{16}H_{17}N_4OS^+$313.1118; Found:313.1116

5-methyl-2-morpholino-6-phenylthiazolo[4,5-*d*]pyrimidin-7(6*H*)-one **(1abe)**

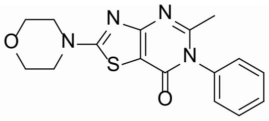

^{1}H NMR (500 MHz, CDCl3) δ 7.54 (d, J = 7.6 Hz, 3H), 7.24 (dd, J = 4.3, 3.8 Hz, 2H), 3.84 (dd, J = 5.6, 4.3 Hz, 4H), 3.70 (d, J = 4.8 Hz, 4H), 2.23 (s, 3H). ^{13}C NMR (126 MHz, $CDCl_3$) δ 173.42, 165.76, 158.18, 157.66, 137.56, 129.96, 129.38, 128.01, 106.35, 77.40, 77.14, 76.89, 66.03, 48.27, 24.20 ; HRMS(ESI) *m/z* $[M+H]^+$Calcd for $C_{16}H_{17}N_4O_2S^+$ 329.1067; Found:329.1067

2-(diethylamino)-5-methyl-6-phenylthiazolo[4,5-*d*]pyrimidin-7(6*H*)-one **(1abf)**

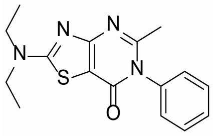

^{1}H NMR (500 MHz, CDCl3) δ 7.57 – 7.46 (m, 3H), 7.25 – 7.21 (m, 2H), 3.61 (s, 4H), 2.22 (s, 3H), 1.30 (t, J = 7.1 Hz, 6H). ^{13}C NMR (126 MHz, $CDCl_3$) δ 172.08, 166.38, 158.09, 138.44, 129.94, 129.29, 128.07, 105.19, 77.30, 77.05, 76.79, 46.24, 24.15, 12.53 ; HRMS(ESI) *m/z* $[M+H]^+$Calcd for $C_{16}H_{19}N_4OS^+$315.1274; Found:315.1273

2-((4-methoxybenzyl)amino)-5-methyl-6-phenylthiazolo[4,5-*d*]pyrimidin-7(6*H*)-one **(1abg)**

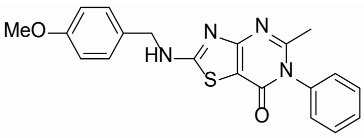

^{1}H NMR (500 MHz, CDCl3) δ 7.56 – 7.47 (m, 3H), 7.31 (d, J = 8.6 Hz, 2H), 7.23 (dd, J = 6.9, 1.5 Hz, 2H), 6.98 (t, J = 33.3 Hz, 1H), 6.90 – 6.85 (m, 2H), 4.56 (d, J = 3.1 Hz, 2H), 3.79 (s, 3H), 2.20 (s, 3H). ^{13}C NMR (126 MHz, $CDCl_3$) δ 165.64, 159.28, 157.83, 157.79, 137.63, 129.97, 129.39, 128.91, 128.02, 114.09, 105.84, 77.36, 77.10, 76.85, 55.32, 48.61, 24.16 ; HRMS(ESI) *m/z* $[M+H]^+$Calcd for $C_{20}H_{19}N_4O_2S^+$ 379.1223; Found:379.1225

5-methyl-6-phenyl-2-(propylthio)thiazolo[4,5-*d*]pyrimidin-7(6*H*)-one **(1abh)**

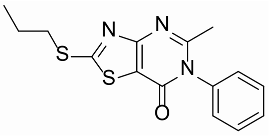

^{1}H NMR (500 MHz, CDCl3) δ 7.60 – 7.50 (m, 3H), 7.25 (dd, J = 6.9, 1.5 Hz, 2H), 3.41 – 3.35 (m, 2H), 2.28 (s, 3H), 1.87 (dd, J = 14.6, 7.3 Hz, 2H), 1.08 (t, J = 7.4 Hz, 3H). ^{13}C NMR (126 MHz, $CDCl_3$) δ 176.40, 165.28, 158.25, 157.60, 137.21, 130.14, 129.64, 127.85, 114.45, 77.36, 77.10, 76.85, 35.73, 24.23, 22.52, 13.35 ; HRMS(ESI) *m/z* $[M+H]^+$Calcd for; $C_{15}H_{16}N_3OS2^+$: 318.0729 Found: 318.0729

2-(benzylthio)-5-methyl-6-phenylthiazolo[4,5-*d*]pyrimidin-7(6*H*) **(1abi)**

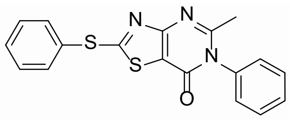

^{1}H NMR (500 MHz, CDCl3) δ 7.74 (s, 2H), 7.60 – 7.46 (m, 6H), 7.22 (s, 2H), 2.26 (s, 3H). ^{13}C NMR (126 MHz, $CDCl_3$) δ 179.96, 166.39, 158.09, 157.63, 137.54, 135.84, 131.41, 130.50, 130.22, 129.69, 128.35, 127.80, 114.83, 77.33, 77.07, 76.82, 24.40 ; HRMS(ESI) *m/z* $[M+H]^+$Calcd for : $C_{18}H_{14}N_3OS_2^+$ 352.0573 Found: 352.0578

2-(benzylthio)-5-methyl-6-phenylthiazolo[4,5-*d*]pyrimidin-7(6*H*)-one **(1abj)**

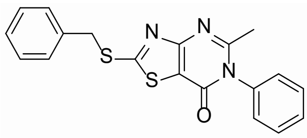

^{1}H NMR (500 MHz, $CDCl_3$) δ 7.57 (d, *J* = 7.5 Hz, 3H), 7.47 – 7.43 (m, 2H), 7.34 (d, *J* = 7.4 Hz, 3H), 7.24 (d, *J* = 1.1 Hz, 1H), 4.67 (s, 2H), 2.29 (s, 3H). ^{13}C NMR (126 MHz, $CDCl_3$) δ 175.58, 165.10, 158.54, 157.20, 136.85,

135.61, 130.24, 129.25, 128.87, 128.05, 127.82, 115.27, 77.33, 77.07, 76.82, 37.94, 24.36 ; HRMS(ESI) *m/z* $[M+H]^+$Calcd for: $C_{19}H_{16}N_3OS_2^+$ 366.0729 Found: 366.0731

2-(butylamino)-6-(4-methoxyphenyl)thiazolo[4,5-*d*]pyrimidin-7(6*H*)-one **(1caa)**

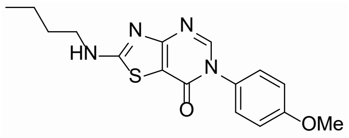

^1H NMR (500 MHz, CDCl3) δ 8.10 (s, 1H), 7.34 – 7.28 (m, 2H), 7.07 – 6.99 (m, 2H), 6.18 (s, 1H), 3.86 (s, 3H), 3.42 (d, J = 5.6 Hz, 2H), 1.74 – 1.67 (m, 2H), 1.45 (dd, J = 15.1, 7.5 Hz, 2H), 0.98 (t, J = 7.4 Hz, 3H) ; ^{13}C NMR (126 MHz, $CDCl_3$) δ 160.24, 156.59, 150.02, 130.37, 128.25, 114.78, 77.29, 77.03, 76.78, 56.03, 45.79, 30.70, 19.56, 14.35. HRMS(ESI) *m/z* $[M+H]^+$Calcd for : $C_{16}H_{19}N_4O_2S^+$: 331.1223 Found:331.1224

2-(benzylamino)-6-(4-methoxyphenyl)thiazolo[4,5-*d*]pyrimidin-7(6*H*)-one **(1cab)**

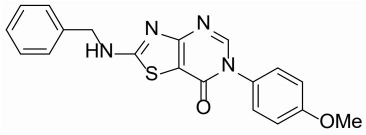

^1H NMR (500 MHz, CDCl3) δ 8.09 (s, 1H), 7.43 – 7.27 (m, 7H), 7.02 (d, J = 8.9 Hz, 2H), 6.64 (s, 1H), 4.64 (d, J = 4.3 Hz, 2H), 3.86 (s, 3H). ^{13}C NMR (126 MHz, $CDCl_3$) δ 166.09, 160.06, 156.49, 149.29, 129.69, 128.80, 128.24, 127.97, 127.94, 114.77, 108.40, 77.33, 77.08, 76.82, 55.62, 49.25; HRMS(ESI) *m/z* $[M+H]^+$Calcd for : $C_{19}H_{17}N_4O_2S_+$ 365.1067 Found: 365.1067

6-(4-methoxyphenyl)-2-(piperidin-1-yl)thiazolo[4,5-*d*]pyrimidin-7(6*H*)-one **(1cac)**

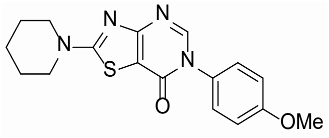

^1H NMR (500 MHz, CDCl3) δ 8.09 (s, 1H), 7.31 (d, J = 9.0 Hz, 2H), 7.01 (d, J = 9.0 Hz, 2H), 3.86 (s, 3H), 3.67 (s, 4H), 1.72 (s, 6H). ^{13}C NMR (126 MHz, $CDCl_3$) δ 173.03, 166.79, 159.98, 156.40, 149.26, 129.85, 128.26, 114.70, 108.25, 77.35, 77.09, 76.84, 55.61, 49.62, 25.23, 24.00; HRMS(ESI) *m/z* $[M+H]^+$Calcd for : $C_{17}H_{19}N_4O_2S^+$ 343.1223 Found: 343.1225

6-(4-methoxyphenyl)-2-(pyrrolidin-1-yl)thiazolo[4,5-*d*]pyrimidin-7(6*H*)-one **(1cad)**

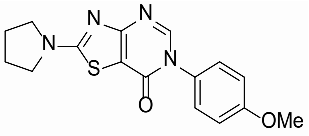

^{1}H NMR (500 MHz, CDCl3) δ 8.10 (s, 1H), 7.31 (d, J = 9.0 Hz, 2H), 7.02 (d, J = 9.0 Hz, 2H), 3.86 (s, 3H), 3.58 (s, 4H), 2.12 (s, 4H). ^{13}C NMR (126 MHz, $CDCl_3$) δ 169.70, 166.80, 159.96, 156.47, 149.25, 129.88, 128.27, 114.70, 108.35, 77.36, 77.10, 76.85, 55.61, 49.81, 25.64; HRMS(ESI) *m/z* $[M+H]^+$Calcd for: $C_{16}H_{17}N_4O_2S^+$ 329.1067 Found: 329.1066

6-(4-methoxyphenyl)-2-morpholinothiazolo[4,5-*d*]pyrimidin-7(6*H*)-one **(1cae)**

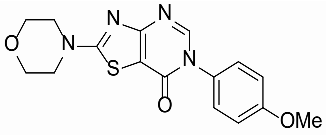

^{1}H NMR (500 MHz, CDCl3) δ 8.12 (s, 1H), 7.31 (d, J = 9.0 Hz, 2H), 7.02 (d, J = 9.0 Hz, 2H), 3.90 – 3.80 (m, 7H), 3.71 (s, 4H). ^{13}C NMR (126 MHz, $CDCl_3$) δ 171.94, 164.88, 158.58, 154.94, 148.00, 128.18, 126.75, 113.27, 75.84, 75.58, 75.33, 64.56, 54.13, 46.78; HRMS(ESI) *m/z* $[M+H]^+$Calcd for : $C_{16}H_{17}N_4O_3S^+$: 345.1016 Found: 345.1016

2-((4-methoxybenzyl)amino)-6-(4-methoxyphenyl)thiazolo[4,5-*d*]pyrimidin-7(6*H*)-one **(1cag)**

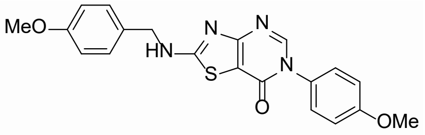

^{1}H NMR (500 MHz, CDCl3) δ 8.10 (s, 1H), 7.31 (dd, J = 10.5, 8.9 Hz, 4H), 7.02 (d, J = 9.0 Hz, 2H), 6.90 (d, J = 8.7 Hz, 2H), 6.29 (s, 1H), 4.57 (d, J = 5.2 Hz, 2H), 3.86 (s, 3H), 3.81 (s, 3H). ^{13}C NMR (126 MHz, $CDCl_3$) δ 166.38, 159.43, 157.04, 149.11, 129.39, 128.23, 114.79, 114.32, 77.29, 77.04, 76.78, 55.57, 49.32; HRMS(ESI) *m/z* $[M+H]^+$Calcd for : $C_{20}H_{19}N_4O_3S^+$ 395.1172 Found: 395.1173

6-(4-methoxyphenyl)-2-(propylthio)thiazolo[4,5-*d*]pyrimidin-7(6*H*)-one **(1cah)**

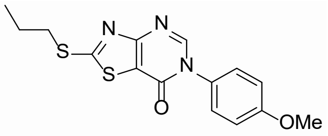

^{1}H NMR (500 MHz, CDCl3) δ 8.18 (s, 1H), 7.31 (d, J = 9.0 Hz, 2H), 7.04 (d, J = 9.0 Hz, 2H), 3.87 (s, 3H), 3.40 – 3.33 (m, 2H), 1.88 (d, J = 7.3 Hz, 2H), 1.09 (t, J = 7.4 Hz, 3H). ^{13}C NMR (126 MHz, $CDCl_3$) δ 176.74, 165.84, 160.27, 156.36, 149.43, 129.26, 128.18, 116.77, 114.91, 77.32, 77.07, 76.81, 55.65, 35.85, 22.47, 13.36 ; HRMS(ESI) *m/z* $[M+H]^+$Calcd for : $C_{15}H_{16}N_3O_2S_2^+$: 334.0678 Found: 334.0678

6-(4-methoxyphenyl)-2-(phenylthio)thiazolo[4,5-*d*]pyrimidin-7(6*H*)-one **(1cai)**

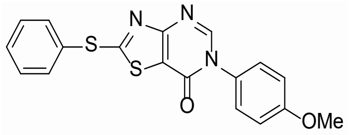

^{1}H NMR (500 MHz, CDCl3) δ 8.17 (s, 1H), 7.75 (dd, J = 8.2, 1.3 Hz, 2H), 7.60 – 7.51 (m, 3H), 7.28 (d, J = 6.8 Hz, 2H), 7.02 (d, J = 9.0 Hz, 2H), 3.85 (s, 3H). ^{13}C NMR (126 MHz, $CDCl_3$) δ 166.54, 160.26, 156.24, 149.56,

135.84, 131.50, 130.56, 129.24, 128.38, 128.15, 114.92, 77.32, 77.06, 76.81, 55.64 ; HRMS(ESI) *m/z* $[M+H]^+$Calcd for : $C_{18}H_{14}N_3O_2S_2^+$: 368.0522 Found: 368.0524

2-(benzylthio)-6-(4-methoxyphenyl)thiazolo[4,5-*d*]pyrimidin-7(6*H*)-one **(1caj)**

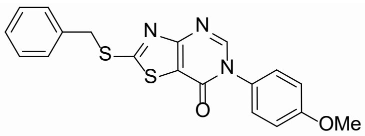

^{1}H NMR (500 MHz, CDCl3) δ 8.20 (s, 1H), 7.49 – 7.44 (m, 2H), 7.38 – 7.28 (m, 5H), 7.04 (d, J = 8.9 Hz, 2H), 4.65 (s, 2H), 3.87 (s, 3H). ^{13}C NMR (126 MHz, $CDCl_3$) δ 175.62, 165.68, 160.30, 156.39, 149.52, 135.36, 129.24, 128.85, 128.19, 128.06, 117.65, 114.93, 77.33, 77.08, 76.82, 55.66, 38.03; HRMS(ESI) *m/z* $[M+H]^+$Calcd for $C_{19}H_{16}N_3O_2S_2^+$: 382.0678 Found: 382.0677

2-(butylamino)-6-(4-methoxyphenyl)-5-methylthiazolo[4,5-*d*]pyrimidin-7(6*H*)-one **(1cba)**

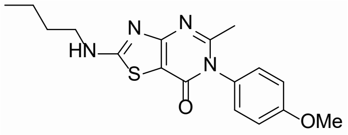

^{1}H NMR (500 MHz, CDCl3) δ 7.14 (d, J = 8.9 Hz, 2H), 7.04 (d, J = 8.9 Hz, 2H), 6.15 (s, 1H), 3.87 (s, 3H), 3.43 (d, J = 6.0 Hz, 2H), 2.24 (s, 3H), 1.70 (s, 2H), 1.45 (d, J = 7.6 Hz, 2H), 0.97 (s, 3H). ^{13}C NMR (126 MHz, $CDCl_3$) δ 165.80, 160.01, 158.34, 158.05, 130.13, 129.00, 115.19, 105.85, 77.33, 77.07, 76.82, 55.57, 45.59, 30.97, 24.22, 20.09, 13.76; HRMS(ESI) *m/z* $[M+H]^+$Calcd for : $C_{17}H_{21}N_4O_2S^+$: 345.1380 Found: 345.1016

2-(benzylamino)-6-(4-methoxyphenyl)-5-methylthiazolo[4,5-*d*]pyrimidin-7(6*H*)-one **(1cbb)**

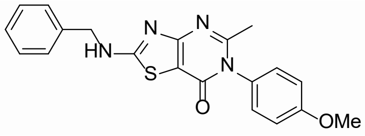

^{1}H NMR (500 MHz, CDCl3) δ 7.38 (d, J = 7.2 Hz, 5H), 7.16 – 7.12 (m, 2H), 7.06 – 7.01 (m, 2H), 6.10 (s, 1H), 4.66 (d, J = 4.6 Hz, 2H), 3.86 (s, 3H), 2.24 (s, 3H). ^{13}C NMR (126 MHz, $CDCl_3$) δ 165.58, 160.01, 158.40, 158.09, 136.99, 130.04, 128.97, 128.69, 127.91, 127.75, 115.19, 105.88, 77.38, 77.12, 76.87, 55.56, 49.01, 24.20; HRMS(ESI) *m/z* $[M+H]^+$Calcd for : $C_{20}H_{19}N_4O_2S^+$: 379.1223 Found: 379.1223

6-(4-methoxyphenyl)-5-methyl-2-(piperidin-1-yl)thiazolo[4,5-*d*]pyrimidin-7(6*H*)-one **(1cbc)**

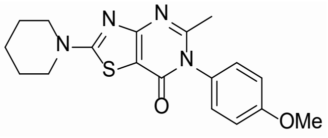

^{1}H NMR (500 MHz, CDCl3) δ 7.15 (d, J = 8.9 Hz, 2H), 7.03 (d, J = 8.9 Hz, 2H), 3.86 (s, 3H), 3.66 (s, 4H), 2.23 (s, 3H), 1.71 (s, 6H). ^{13}C NMR (126 MHz, $CDCl_3$) δ 173.00, 166.11, 159.95, 158.39, 157.92, 130.17, 129.02,

115.13, 105.74, 77.40, 77.14, 76.89, 55.54, 49.59, 25.21, 24.21, 24.04; HRMS(ESI) *m/z* $[M+H]^+$Calcd for: $C_{18}H_{21}N_4O_2S^+$: 357.1380 Found: 357.1379

6-(4-methoxyphenyl)-5-methyl-2-(pyrrolidin-1-yl)thiazolo[4,5-*d*]pyrimidin-7(6*H*)-one **(1cbd)**

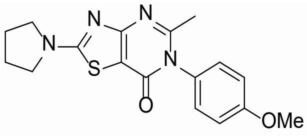

^1H NMR (500 MHz, CDCl3) δ 7.18 – 7.11 (m, 2H), 7.05 – 7.00 (m, 2H), 3.86 (s, 3H), 3.57 (s, 4H), 2.2 (s, 3H), 2.1 (s, 4H). ^{13}C NMR (126 MHz, $CDCl_3$) δ 169.64, 166.15, 159.95, 158.36, 158.03, 130.22, 129.04, 115.14, 105.84, 77.37, 77.11, 76.86, 55.55, 49.72, 25.64, 24.23 ; HRMS(ESI) *m/z* $[M+H]^+$Calcd for: : $C_{17}H_{19}N_4O_2S^+$: 343.1223 Found: 343.1223

6-(4-methoxyphenyl)-5-methyl-2-morpholinothiazolo[4,5-*d*]pyrimidin-7(6*H*)-one **(1cbe)**

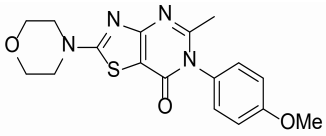

^1H NMR (500 MHz, CDCl3) δ 7.15 (d, J = 8.9 Hz, 2H), 7.04 (d, J = 8.9 Hz, 2H), 3.87 (s, 3H), 3.85 – 3.82 (m, 4H), 3.69 (s, 4H), 2.25 (s, 3H). ^{13}C NMR (126 MHz, $CDCl_3$) δ 173.42, 165.70, 160.05, 158.74, 157.95, 130.00, 128.97, 115.20, 77.36, 77.11, 76.85, 66.04, 55.56, 48.26, 24.23; HRMS(ESI) *m/z* $[M+H]^+$Calcd for: $C_{17}H_{19}N_4O_3S^+$: 359.1172 Found: 359.1172

2-(diethylamino)-6-(4-methoxyphenyl)-5-methylthiazolo[4,5-*d*]pyrimidin-7(6*H*)-one **(1cbf)**

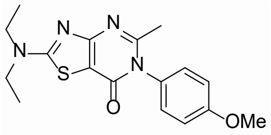

^1H NMR (500 MHz, CDCl3) δ 7.14 (d, J = 8.9 Hz, 2H), 7.03 (d, J = 8.9 Hz, 2H), 3.86 (s, 3H), 3.58 (s, 4H), 2.23 (s, 3H), 1.30 (t, J = 7.1 Hz, 6H). ^{13}C NMR (126 MHz, $CDCl_3$) δ 171.66, 166.38, 160.26, 157.65, 129.92, 129.02, 115.18, 106.08, 77.29, 77.04, 76.78, 55.55, 46.03, 24.18; HRMS(ESI) *m/z* $[M+H]^+$Calcd for : $C_{17}H_{21}N_4O_2S^+$: 345.1380 Found: 345.1380

2-((4-methoxybenzyl)amino)-6-(4-methoxyphenyl)-5-methylthiazolo[4,5-*d*]pyrimidin-7(6*H*)-one **(1cbg)**

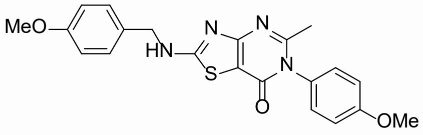

^1H NMR (500 MHz, CDCl3) δ 7.31 (d, J = 8.7 Hz, 2H), 7.14 (d, J = 8.9 Hz, 2H), 7.03 (d, J = 8.9 Hz, 2H), 6.89 (d, J = 8.7 Hz, 2H), 6.07 (s, 1H), 4.57 (d, J = 5.1 Hz, 2H), 3.87 (s, 3H), 3.81 (s, 3H), 2.24 (s, 3H). ^{13}C NMR (126

MHz, $CDCl_3$) δ 172.74, 165.56, 160.02, 159.27, 158.38, 158.05, 130.08, 129.37, 128.99, 115.18, 114.09, 105.64, 77.36, 77.10, 76.85, 55.56, 55.31, 48.60, 24.19; HRMS(ESI) *m/z* $[M+H]^+$Calcd for: $C_{21}H_{21}N_4O_3S^+$: 409.1329 Found: 409.1329

6-(4-methoxyphenyl)-5-methyl-2-(propylthio)thiazolo[4,5-*d*]pyrimidin-7(6*H*)-one **(1cbh)**

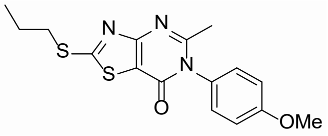

^{1}H NMR (500 MHz, CDCl3) δ 7.18 – 7.10 (m, 2H), 7.09 – 6.99 (m, 2H), 3.87 (s, 3H), 3.45 – 3.33 (m, 2H), 2.30 (s, 3H), 1.87 (dd, J = 14.6, 7.3 Hz, 2H), 1.08 (t, J = 7.4 Hz, 3H). ^{13}C NMR (126 MHz, $CDCl_3$) δ 176.27, 165.20, 160.22, 158.80, 157.85, 129.61, 128.84, 115.34, 114.41, 77.37, 77.12, 76.86, 55.58, 35.71, 24.26, 22.52, 13.35 ; HRMS(ESI) *m/z* $[M+H]^+$Calcd for: $C_{16}H_{18}N_3O_2S_2^+$: 348.0835 Found: 348.0836

6-(4-methoxyphenyl)-5-methyl-2-(phenylthio)thiazolo[4,5-*d*]pyrimidin-7(6*H*)-one **(1cbi)**

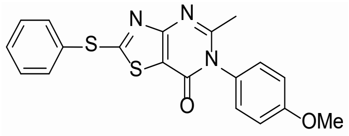

^{1}H NMR (500 MHz, CDCl3) δ 7.80 – 7.71 (m, 2H), 7.60 – 7.48 (m, 3H), 7.14 – 7.05 (m, 2H), 7.05 – 6.94 (m, 2H), 3.86 (s, 3H), 2.27 (s, 3H). ^{13}C NMR (126 MHz, $CDCl_3$) δ 179.38, 165.96, 160.19, 158.92, 157.72, 135.77, 131.29, 130.42, 129.60, 128.85, 128.50, 115.34, 115.19, 77.14, 76.88, 55.58, 24.33; HRMS(ESI) *m/z* $[M+H]^+$Calcd for : $C_{19}H_{16}N_3O_2S_2^+$: 382.0678 ; Found: 382.0678

2-(benzylthio)-6-(4-methoxyphenyl)-5-methylthiazolo[4,5-*d*]pyrimidin-7(6*H*)-one **(1cbj)**

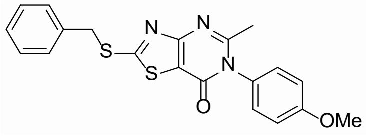

^{1}H NMR (500 MHz, CDCl3) δ 7.45 (d, J = 7.2 Hz, 2H), 7.37 – 7.25 (m, 3H), 7.15 (d, J = 8.9 Hz, 2H), 7.06 (d, J = 8.9 Hz, 2H), 4.66 (s, 2H), 3.88 (s, 3H), 2.31 (s, 3H). ^{13}C NMR (126 MHz, $CDCl_3$) δ 175.25, 165.09, 160.25, 158.92, 157.90, 135.48, 129.58, 129.20, 128.85, 128.83, 127.99, 115.38, 114.75, 77.38, 77.13, 76.87, 55.59, 37.96, 24.31; HRMS(ESI) *m/z* $[M+H]^+$Calcd for: $C_{20}H_{18}N_3O_2S_2^+$: 396.0835 Found: 396.0837

2-(butylamino)-5-methyl-6-(*p*-tolyl)thiazolo[4,5-*d*]pyrimidin-7(6*H*)-one **(1bba)**

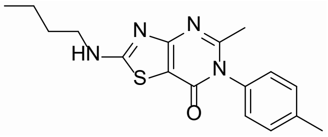

^{1}H NMR (500 MHz, CDCl3) δ 7.33 (d, J = 8.0 Hz, 2H), 7.17 – 7.06 (m, 2H), 6.25 (s, 1H), 3.42 (d, J = 6.2 Hz, 2H), 2.43 (s, 3H), 2.22 (s, 3H), 1.72 (d, J = 7.5 Hz, 2H), 1.43 (dd, J = 15.1, 7.5 Hz, 2H), 0.96 (t, J = 7.4 Hz, 3H). ^{13}C NMR (126 MHz, $CDCl_3$) δ 173.84, 165.92, 157.80, 157.70, 137.69, 129.96, 129.34, 128.06, 105.27, 77.38,

77.13, 76.87, 45.57, 30.90, 24.15, 20.11, 13.77 ; HRMS(ESI) *m/z* [M+H]$^+$Calcd for : $C_{17}H_{21}N_4OS^+$: 329.1431 Found: 329.1066

2-(benzylamino)-5-methyl-6-(*p*-tolyl)thiazolo[4,5-d]pyrimidin-7(6*H*)-one **(1bbb)**

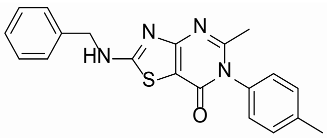

^{1}H NMR (500 MHz, CDCl3) δ 7.38 (t, J = 4.3 Hz, 5H), 7.33 (d, J = 8.0 Hz, 2H), 7.10 (d, J = 8.2 Hz, 2H), 6.32 (s, 1H), 4.65 (d, J = 3.1 Hz, 2H), 2.43 (s, 3H), 2.23 (s, 3H). ^{13}C NMR (126 MHz, $CDCl_3$) δ 172.73, 165.64, 157.88, 157.80, 137.60, 136.90, 129.98, 129.38, 128.73, 128.00, 127.93, 127.82, 105.95, 77.34, 77.09, 76.84, 49.09, 24.16 ; HRMS(ESI) *m/z* [M+H]$^+$Calcd for : $C_{20}H_{19}N_4OS^+$: 363.1274 Found: 363.1274

5-methyl-2-(piperidin-1-yl)-6-(*p*-tolyl)thiazolo[4,5-*d*]pyrimidin-7(6*H*)-one **(1bbc)**

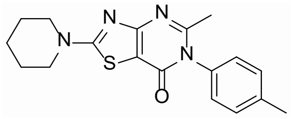

^{1}H NMR (500 MHz, CDCl3) δ 7.33 (d, J = 7.9 Hz, 2H), 7.15 – 7.08 (m, 2H), 3.66 (s, 4H), 2.43 (s, 3H), 2.22 (s, 3H), 1.71 (s, 6H). ^{13}C NMR (126 MHz, $CDCl_3$) δ 173.03, 166.19, 157.85, 157.66, 137.73, 129.91, 129.27, 128.07, 105.78, 77.37, 77.12, 76.87, 49.61, 25.21, 24.16, 24.04; HRMS(ESI) *m/z* [M+H]$^+$Calcd for: $C_{18}H_{21}N_4OS^+$ 341.1431 Found: 341.1429

5-methyl-2-morpholino-6-(p-tolyl)thiazolo[4,5-*d*]pyrimidin-7(6*H*)-one **(1bbe)**

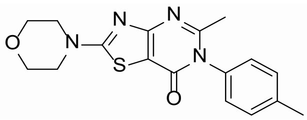

^{1}H NMR (500 MHz, CDCl3) δ 7.34 (d, J = 8.1 Hz, 2H), 7.12 (d, J = 8.2 Hz, 2H), 3.89 – 3.79 (m, 4H), 3.69 (s, 4H), 2.43 (s, 3H), 2.23 (s, 3H). ^{13}C NMR (126 MHz, $CDCl_3$) δ 174.07, 165.92, 158.99, 157.88, 139.32, 134.71, 130.65, 127.64, 106.75, 77.30, 77.05, 76.79, 66.07, 48.27, 24.20, 21.27.; HRMS(ESI) *m/z* [M+H]$^+$Calcd for: : $C_{17}H_{19}N_4O_2S^+$: 343.1223 Found: 343.1224

2-((4-methoxybenzyl)amino)-5-methyl-6-(*p*-tolyl)thiazolo[4,5-*d*]pyrimidin-7(6*H*)-one **(1bbg)**

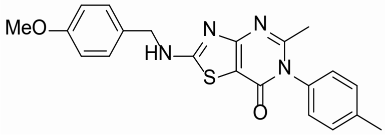

^{1}H NMR (500 MHz, CDCl3) δ 7.32 (dd, J = 11.4, 4.9 Hz, 5H), 7.13 – 7.08 (m, 2H), 6.92 – 6.86 (m, 2H), 6.13 (s, 1H), 4.57 (d, J = 4.2 Hz, 2H), 3.84 – 3.79 (m, 3H), 2.43 (s, 3H), 2.23 (s, 3H). ^{13}C NMR (126 MHz, $CDCl_3$) δ 173.18, 165.58, 159.36, 158.13, 157.91, 139.43, 134.94, 130.63, 129.39, 127.65, 114.16, 105.64, 77.32, 77.07, 76.81, 55.33, 48.70, 24.18, 21.27; HRMS(ESI) *m/z* [M+H]$^+$Calcd for : $C_{21}H_{21}N_4O_2S^+$: 393.1380 Found: 393.1380

5-methyl-2-(propylthio)-6-(*p*-tolyl)thiazolo[4,5-*d*]pyrimidin-7(6*H*)-one **(1bbh)**

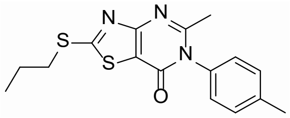

^{1}H NMR (500 MHz, CDCl3) δ 7.39 – 7.32 (m, 2H), 7.15 – 7.09 (m, 2H), 3.42 – 3.37 (m, 2H), 2.44 (s, 3H), 2.28 (s, 3H), 1.87 (dd, J = 14.6, 7.3 Hz, 2H), 1.08 (t, J = 7.4 Hz, 3H). ^{13}C NMR (126 MHz, $CDCl_3$) δ 175.80, 165.34, 158.97, 157.85, 140.16, 134.25, 130.81, 127.49, 114.82, 77.30, 77.04, 76.79, 35.72, 24.23, 22.54, 21.29, 13.35; HRMS(ESI) *m/z* $[M+H]^+$Calcd for: : $C_{16}H_{18}N_3OS_2^+$: 332.0886 Found: 332.0883

2-(benzylthio)-5-methyl-6-(*p*-tolyl)thiazolo[4,5-*d*]pyrimidin-7(6*H*)-one **(1bbj)**

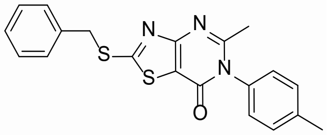

^{1}H NMR (500 MHz, CDCl3) δ 7.45 (d, J = 7.2 Hz, 2H), 7.35 (dd, J = 10.2, 7.6 Hz, 5H), 7.12 (d, J = 8.3 Hz, 2H), 4.66 (s, 2H), 2.45 (s, 3H), 2.30 (s, 3H). ^{13}C NMR (126 MHz, $CDCl_3$) δ 175.35, 165.16, 158.38, 157.62, 137.19, 135.47, 130.16, 129.67, 129.21, 128.84, 128.01, 127.86, 114.78, 77.41, 77.16, 76.91, 37.97, 24.29; HRMS(ESI) *m/z* $[M+H]^+$Calcd for: $C_{20}H_{18}N_3OS_2^+$: 380.0886 Found: 380.0883

2-(butylamino)-6-(4-nitrophenyl)thiazolo[4,5-*d*]pyrimidin-7(6*H*)-one **(1daa)**

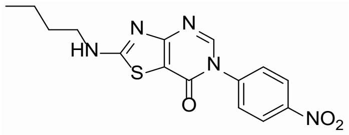

^{1}H NMR (500 MHz, CDCl3) δ 8.43 – 8.38 (m, 2H), 8.12 (s, 1H), 7.68 – 7.61 (m, 2H), 6.13 (s, 1H), 3.44 (d, J = 6.2 Hz, 2H), 1.78 – 1.68 (m, 2H), 1.46 (dd, J = 15.1, 7.5 Hz, 2H), 0.99 (t, J = 7.4 Hz, 3H). ^{13}C NMR (126 MHz, $CDCl_3$) δ 193.95, 166.84, 155.70, 148.30, 142.40, 127.98, 125.15, 77.28, 77.03, 76.77, 45.12, 30.92, 19.57, 13.22 ; HRMS(ESI) *m/z* $[M+H]^+$Calcd for: $C_{15}H_{16}N_5O_3S^+$: 346.0968 Found: 346.0970

6-(4-nitrophenyl)-2-(piperidin-1-yl)thiazolo[4,5-*d*]pyrimidin-7(6*H*)-one **(1dac)**

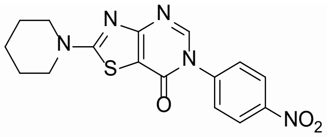

^{1}H NMR (500 MHz, CDCl3) δ 8.40 (d, J = 9.0 Hz, 2H), 8.11 (s, 1H), 7.65 (d, J = 9.0 Hz, 2H), 3.70 (s, 4H), 1.74 (s, 6H). ^{13}C NMR (126 MHz, $CDCl_3$) δ 213.16, 172.96, 166.37, 154.58, 147.82, 142.39, 128.15, 124.81, 108.05, 77.31, 77.06, 76.81, 50.12, 25.24, 23.94 ; HRMS(ESI) *m/z* $[M+H]^+$Calcd for: : $C_{16}H_{16}N_5O_3S^+$: 358.0968 Found: 358.0969

2-morpholino-6-(4-nitrophenyl)thiazolo[4,5-*d*]pyrimidin-7(6*H*)-one **(1dae)**

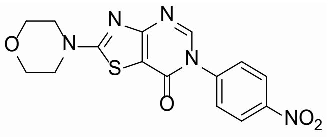

^{1}H NMR (500 MHz, CDCl3) δ 8.41 (d, J = 9.0 Hz, 2H), 8.14 (s, 1H), 7.65 (d, J = 9.1 Hz, 2H), 3.89 – 3.84 (m, 4H), 3.73 (s, 4H). ^{13}C NMR (126 MHz, $CDCl_3$) δ 173.85, 166.15, 156.15, 148.30, 142.41, 127.52, 125.51, 77.30, 77.04, 76.79, 65.66, 47.97; HRMS(ESI) *m/z* $[M+H]^+$Calcd for: : $C_{15}H_{14}N_5O_4S^+$: 360.0761 Found:360.0762

2-(diethylamino)-6-(4-nitrophenyl)thiazolo[4,5-*d*]pyrimidin-7(6*H*)-one **(1daf)**

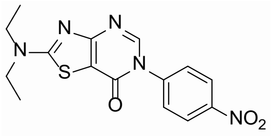

^{1}H NMR (500 MHz, CDCl3) δ 8.40 (d, J = 9.0 Hz, 2H), 8.11 (s, 1H), 7.64 (d, J = 9.0 Hz, 2H), 3.64 (s, 4H), 1.33 (t, J = 7.2 Hz, 6H). ^{13}C NMR (126 MHz, $CDCl_3$) δ 172.33, 166.80, 155.32, 147.76, 147.74, 142.41, 128.16, 124.81, 108.26, 77.33, 77.07, 76.82, 46.61, 12.44 ; HRMS(ESI) *m/z* $[M+H]^+$Calcd for : $C_{15}H_{16}N_5O_3S^+$: 346.0968 Found: 346.0970

6-(4-nitrophenyl)-2-(propylthio)thiazolo[4,5-*d*]pyrimidin-7(6*H*)-one **(1dah)**

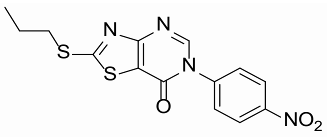

^{1}H NMR (500 MHz, CDCl3) δ 8.44 (d, J = 9.0 Hz, 2H), 8.20 (s, 1H), 7.66 (d, J = 9.0 Hz, 2H), 3.45 – 3.36 (m, 2H), 1.89 (d, J = 7.3 Hz, 2H), 1.10 (t, J = 7.4 Hz, 3H). ^{13}C NMR (126 MHz, $CDCl_3$) δ 177.95, 165.11, 155.91, 147.91, 141.95, 128.19, 125.03, 116.76, 77.31, 77.06, 76.81, 35.98, 22.42, 13.34; HRMS(ESI) *m/z* $[M+H]^+$Calcd for : $C_{14}H_{13}N_4O_3S_2^+$: 349.0424 Found: 349.0425

6-(4-nitrophenyl)-2-(phenylthio)thiazolo[4,5-*d*]pyrimidin-7(6*H*)-one **(1dai)**

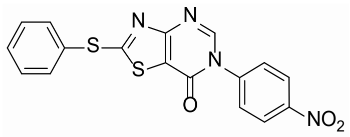

^{1}H NMR (500 MHz, CDCl3) δ 8.42 (d, J = 9.0 Hz, 2H), 8.18 (s, 1H), 7.75 (s, 2H), 7.62 (d, J = 9.0 Hz, 3H), 7.56 (s, 2H). ^{13}C NMR (126 MHz, $CDCl_3$) δ 181.36, 166.42, 155.25, 148.10, 148.04, 141.75, 135.84, 131.70, 130.68, 128.20, 128.09, 125.01, 117.43, 77.34, 77.09, 76.83; HRMS(ESI) *m/z* $[M+H]^+$Calcd for: $C_{17}H_{11}N_4O_3S_2^+$: 383.0267 Found: 383.0268

2-(benzylthio)-6-(4-nitrophenyl)thiazolo[4,5-*d*]pyrimidin-7(6*H*)-one **(1daj)**

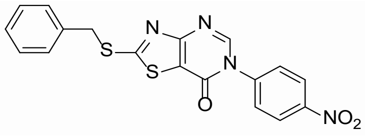

^{1}H NMR (500 MHz, CDCl3) δ 8.44 (d, J = 9.0 Hz, 2H), 8.22 (s, 1H), 7.65 (d, J = 9.0 Hz, 2H), 7.46 (s, 2H), 7.36 (s, 3H), 4.66 (s, 2H). ^{13}C NMR (126 MHz, $CDCl_3$) δ 177.74, 165.12, 155.91, 147.97, 142.77, 135.38, 129.23, 129.23, 128.90, 128.19, 128.19, 125.06, 117.43, 77.30, 77.30, 77.05, 76.80, 38.15; HRMS(ESI) *m/z* $[M+H]^+$Calcd for : $C_{18}H_{13}N_4O_3S_2^+$: 397.0424 Found: 397.0428

2-(butylamino)-5-methyl-6-(4-nitrophenyl)thiazolo[4,5-*d*]pyrimidin-7(6*H*)-one **(1dba)**

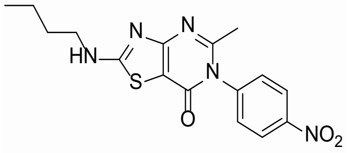

^{1}H NMR (500 MHz, CDCl3) δ 8.44 – 8.40 (m, 2H), 7.49 – 7.45 (m, 2H), 5.86 (s, 1H), 3.45 (d, J = 6.4 Hz, H), 2.24 (s, 3H), 1.75 – 1.62 (m, 2H), 1.45 (dd, J = 15.1, 7.4 Hz, 2H), 0.98 (t, J = 7.4 Hz, 3H). ^{13}C NMR (126 MHz, $CDCl_3$) δ 148.32, 142.63, 129.47, 124.69, 77.28, 77.03, 76.77, 45.57, 32.20, 24.56, 19.59, 14.11; HRMS(ESI) *m/z* $[M+H]^+$Calcd for : $C_{16}H_{18}N_5O_3S^+$: 360.1125 Found: 360.1125

2-(benzylamino)-5-methyl-6-(4-nitrophenyl)thiazolo[4,5-*d*]pyrimidin-7(6*H*)-one **(1dbb)**

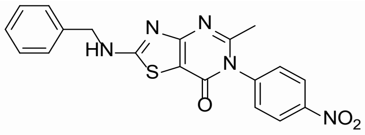

^{1}H NMR (500 MHz, CDCl3) δ 8.42 (d, J = 9.0 Hz, 2H), 7.47 (d, J = 9.0 Hz, 2H), 7.39 (dd, J = 3.7, 1.8 Hz, 5H), 6.21 (s, 1H), 4.67 (d, J = 5.4 Hz, 2H), 2.25 (s, 3H). ^{13}C NMR (126 MHz, $CDCl_3$) δ 165.78, 157.04, 149.12, 143.21, 130.36, 129.03, 128.58, 127.98, 125.50, 77.28, 77.03, 76.77, 50.12, 25.02 ; HRMS(ESI) *m/z* $[M+H]^+$Calcd for: : $C_{19}H_{16}N_5O_3S^+$: 394.0968 Found: 394.0972

5-methyl-6-(4-nitrophenyl)-2-(pyrrolidin-1-yl)thiazolo[4,5-*d*]pyrimidin-7(6*H*)-one **(1dbd)**

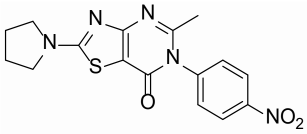

^{1}H NMR (500 MHz, CDCl3) δ 8.42 (d, J = 8.9 Hz, 2H), 7.48 (d, J = 9.0 Hz, 2H), 3.63 (d, J = 75.6 Hz, 4H), 2.24 (s, 3H), 2.13 (s, 4H). ^{13}C NMR (126 MHz, $CDCl_3$) δ 169.87, 166.36, 157.15, 156.48, 148.22, 143.39, 129.76, 125.23, 105.64, 77.32, 77.06, 76.81, 49.87, 25.64, 24.16; HRMS(ESI) *m/z* $[M+H]^+$Calcd for : $C_{16}H_{16}N_5O_3S^+$: 358.0968 Found: 358.0968

5-methyl-2-morpholino-6-(4-nitrophenyl)thiazolo[4,5-*d*]pyrimidin-7(6*H*)-one **(1dbe)**

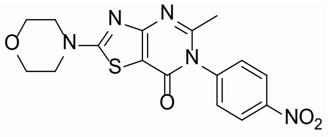

^{1}H NMR (500 MHz, CDCl3) δ 8.42 (d, J = 8.9 Hz, 2H), 7.48 (d, J = 9.0 Hz, 2H), 3.62 (d, J = 138.3 Hz, 4H), 2.24 (s, 3H), 2.13 (s, 4H). ^{13}C NMR (126 MHz, $CDCl_3$) δ 173.40, 165.91, 157.18, 156.15, 148.30, 143.00, 129.68, 125.30, 118.57, 105.42, 77.32, 77.07, 76.81, 66.02, 48.35, 24.18; HRMS(ESI) *m/z* $[M+H]^+$Calcd for : $C_{16}H_{16}N_5O_4S^+$: 374.0918 Found: 374.0920

2-(benzylthio)-5-methyl-6-(4-nitrophenyl)thiazolo[4,5-*d*]pyrimidin-7(6*H*)-one **(1dbj)**

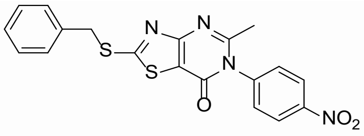

^{1}H NMR (500 MHz, CDCl3) δ 8.45 (d, J = 9.0 Hz, 2H), 7.49 (d, J = 9.0 Hz, 3H), 7.35 (s, 4H), 4.67 (s, 2H), 2.31 (s, 3H). ^{13}C NMR (126 MHz, $CDCl_3$) δ 176.25, 165.21, 157.11, 157.00, 148.46, 142.66, 135.22, 129.55, 129.20, 129.09, 128.88, 128.11, 125.48, 119.24, 114.61, 77.34, 77.09, 76.83, 38.06, 24.24; LC-MS (ESI) m/z 411 ([M+1] +). HRMS(ESI) *m/z* $[M+H]^+$Calcd for : $C_{19}H_{15}N_4O_3S_2^+$: 411.0580 Found: 411.0583

1.5 NMR Spectra

1aaa

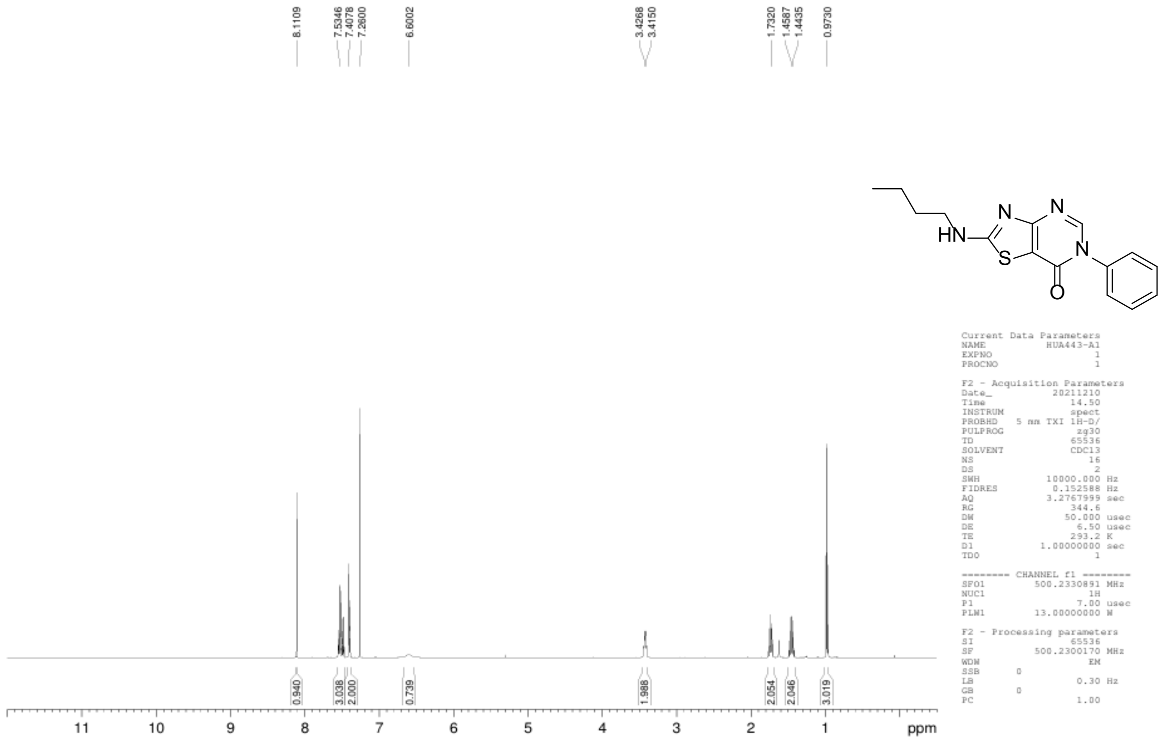

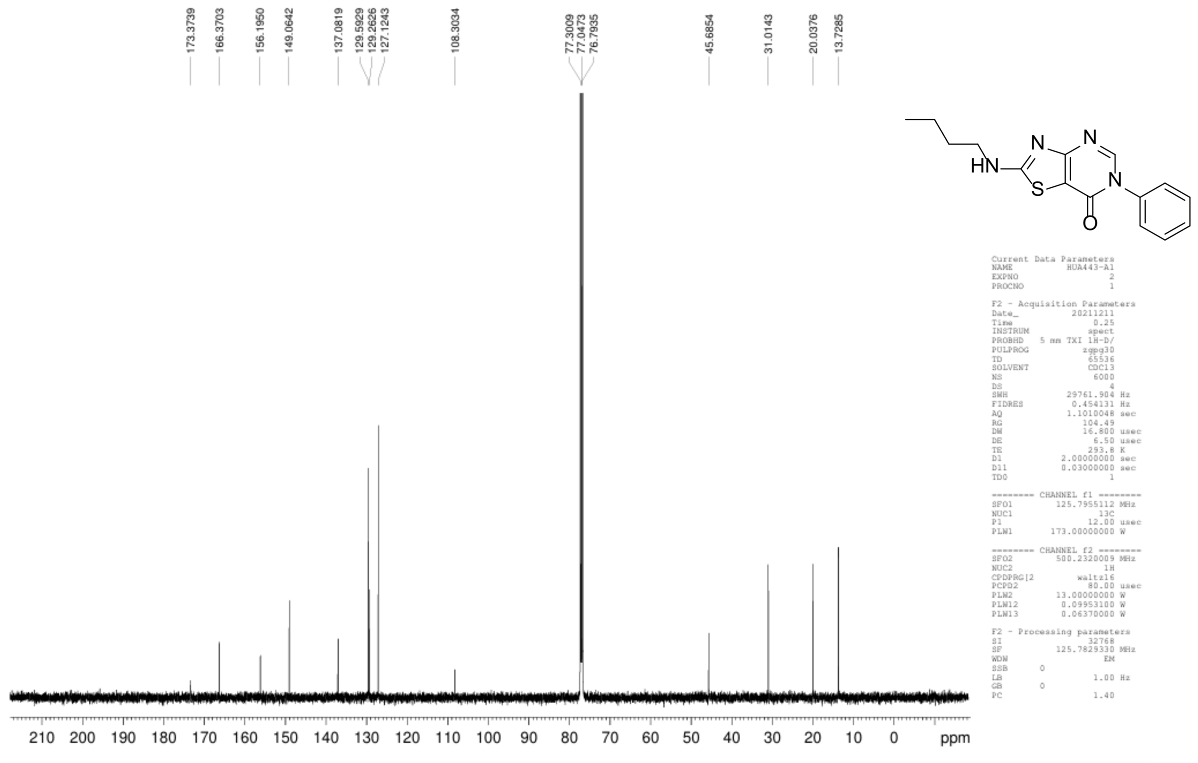

1aab

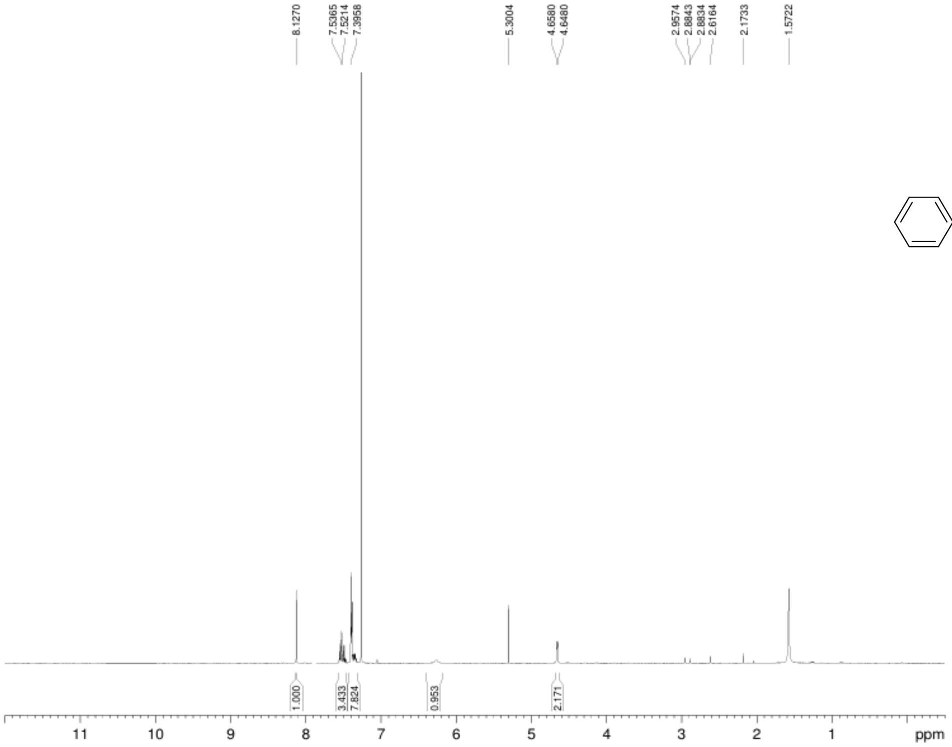
8.1270
7.5365
7.5214
7.3958
5.3004
4.6580
4.6480
2.9574
2.8843
2.8834
2.6164
2.1733
1.5722
1.000
3.433
7.824
0.953
2.171
11 10 9 8 7 6 5 4 3 2 1 ppm

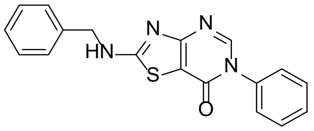
HN
N
N
N
S
O

Current Data Parameters
NAME HUA443-A4
EXPNO 1
PROCNO 1
F2 - Acquisition Parameters
Date_ 20211210
Time 15.11
INSTRUM spect
PROBHD 5 mm TXI 1H-D/
PULPROG zg30
TD 65536
SOLVENT CDCl3
NS 16
DS 2
SWH 10000.000 Hz
FIDRES 0.152588 Hz
AQ 3.2767999 sec
RG 488.68
DW 50.000 usec
DE 6.50 usec
TE 293.2 K
D1 1.00000000 sec
TD0 1
======== CHANNEL f1 ========
SFO1 500.2330891 MHz
NUC1 1H
P1 7.00 usec
PLW1 13.00000000 W
F2 - Processing parameters
SI 65536
SF 500.2300171 MHz
WDW EM
SSB 0
LB 0.30 Hz
GB 0
PC 1.00

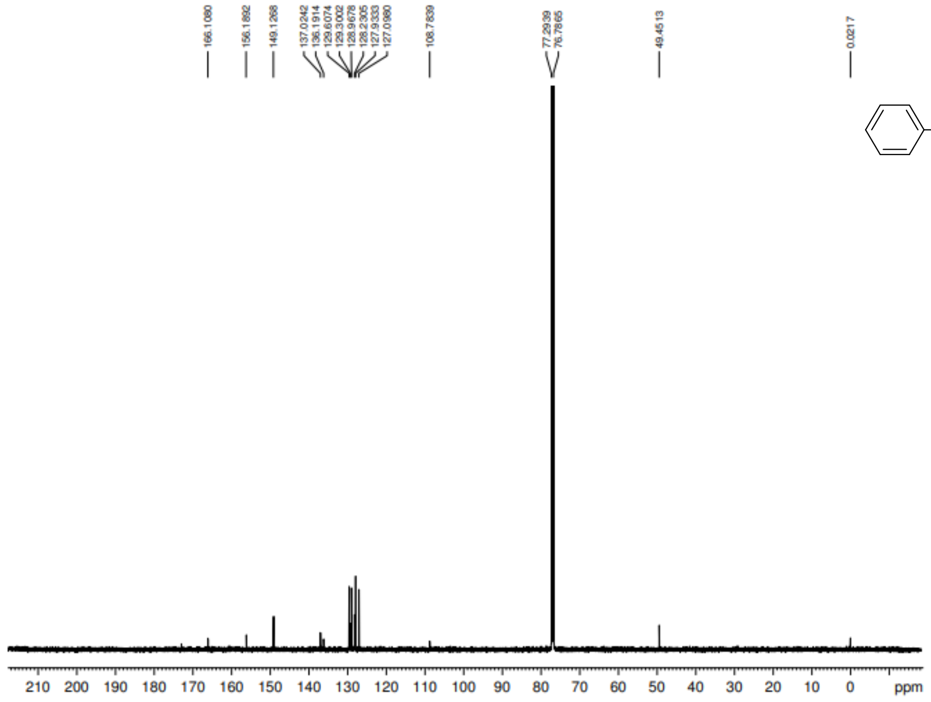
166.1080
156.1892
149.1268
137.0242
136.1914
129.6074
129.3002
128.9678
128.2305
127.9333
127.0980
108.7839
77.2939
76.7865
49.4513
0.0217
210 200 190 180 170 160 150 140 130 120 110 100 90 80 70 60 50 40 30 20 10 0 ppm

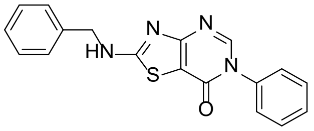
HN
N
N
N
S
O

Current Data Parameters
NAME 2aab-C
EXPNO 1
PROCNO 1
F2 - Acquisition Parameters
Date_ 20240515
Time 3.38
INSTRUM spect
PROBHD 5 mm TXI 1H-D/
PULPROG zgpg30
TD 65536
SOLVENT CDCl3
NS 3500
DS 4
SWH 29761.904 Hz
FIDRES 0.454131 Hz
AQ 1.1010048 sec
RG 59.75
DW 16.800 usec
DE 6.50 usec
TE 296.9 K
D1 2.00000000 sec
D11 0.03000000 sec
TD0 1
======== CHANNEL f1 ========
SFO1 125.7955112 MHz
NUC1 13C
P1 12.00 usec
PLW1 173.00000000 W
======== CHANNEL f2 ========
SFO2 500.2320009 MHz
NUC2 1H
CPDPRG[2 waltz16
PCPD2 80.00 usec
PLW2 13.00000000 W
PLW12 0.09953100 W
PLW13 0.06370000 W
F2 - Processing parameters
SI 32768
SF 125.7829330 MHz
WDW EM
SSB 0
LB 1.00 Hz
GB 0
PC 1.40

1aac

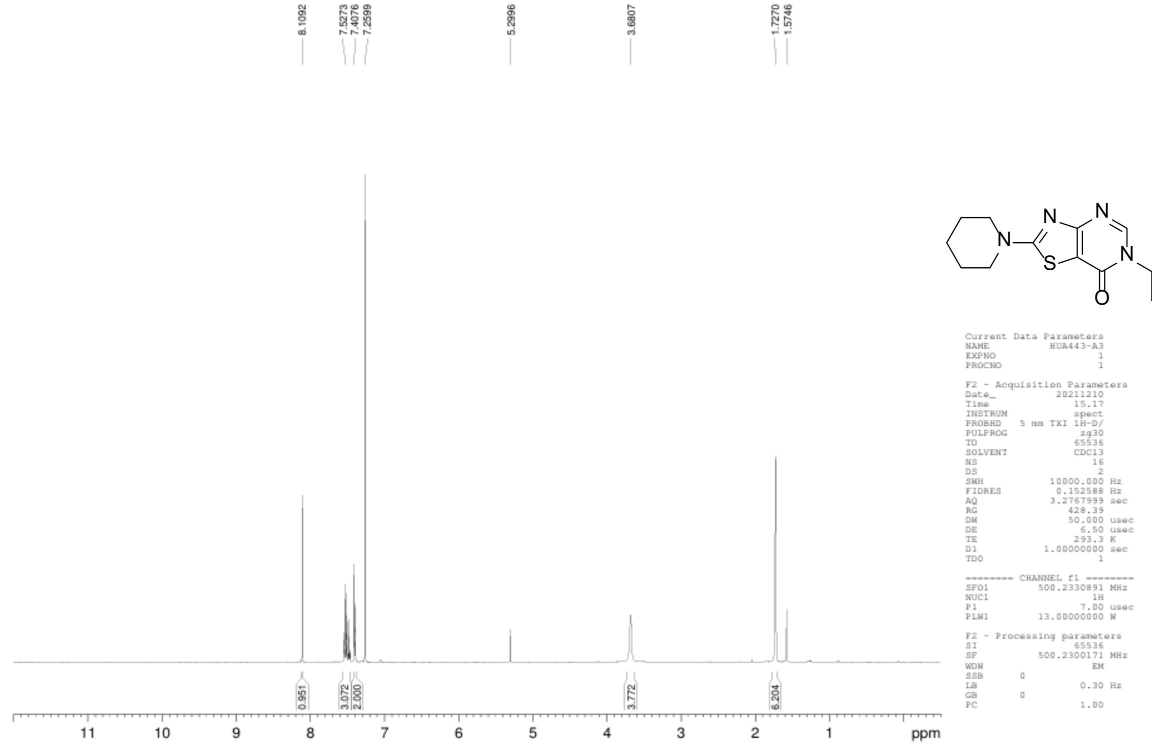

8.1092
7.5273
7.4076
7.2599
5.2996
3.6807
1.7270
1.5746
0.951
3.072
2.000
3.772
6.204
11 10 9 8 7 6 5 4 3 2 1 ppm
N
N
N
S
N
O
Current Data Parameters
NAME HUA443-A3
EXPNO 1
PROCNO 1
F2 - Acquisition Parameters
Date_ 20211210
Time 15.17
INSTRUM spect
PROBHD 5 mm TXI 1H-D/
PULPROG zg30
TD 65536
SOLVENT CDCl3
NS 16
DS 2
SWH 10000.000 Hz
FIDRES 0.152588 Hz
AQ 3.2767999 sec
RG 428.39
DW 50.000 usec
DE 6.50 usec
TE 293.3 K
D1 1.00000000 sec
TD0 1
======== CHANNEL f1 ========
SFO1 500.2330891 MHz
NUC1 1H
P1 7.00 usec
PLW1 13.00000000 W
F2 - Processing parameters
SI 65536
SF 500.2300171 MHz
WDW EM
SSB 0
LB 0.30 Hz
GB 0
PC 1.00

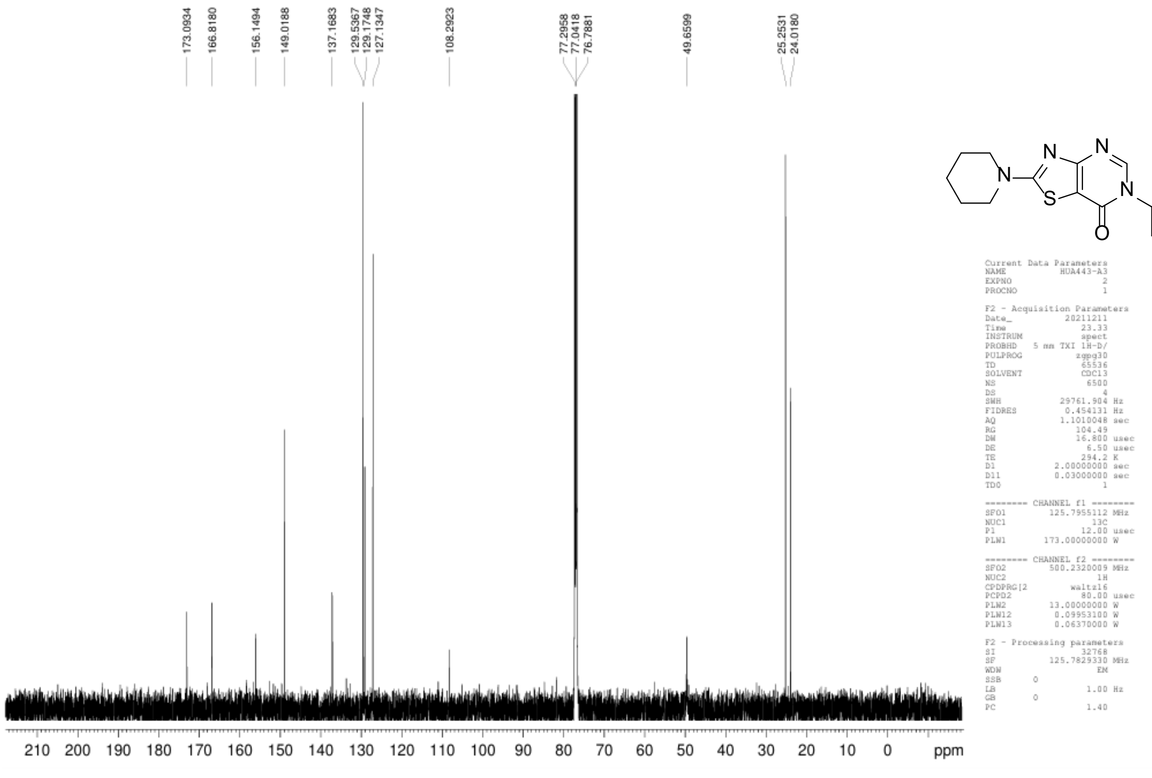

173.0934
166.8180
156.1494
149.0188
137.1683
129.5367
129.1748
127.1347
108.2923
77.2958
77.0418
76.7881
49.6599
25.2531
24.0180
210 200 190 180 170 160 150 140 130 120 110 100 90 80 70 60 50 40 30 20 10 0 ppm
N
N
N
S
N
O
Current Data Parameters
NAME HUA443-A3
EXPNO 2
PROCNO 1
F2 - Acquisition Parameters
Date_ 20211211
Time 23.33
INSTRUM spect
PROBHD 5 mm TXI 1H-D/
PULPROG zgpg30
TD 65536
SOLVENT CDCl3
NS 6500
DS 4
SWH 29761.904 Hz
FIDRES 0.454131 Hz
AQ 1.1010048 sec
RG 104.49
DW 16.800 usec
DE 6.50 usec
TE 294.2 K
D1 2.00000000 sec
D11 0.03000000 sec
TD0 1
======== CHANNEL f1 ========
SFO1 125.7955112 MHz
NUC1 13C
P1 12.00 usec
PLW1 173.00000000 W
======== CHANNEL f2 ========
SFO2 500.2320009 MHz
NUC2 1H
CPDPRG[2 waltz16
PCPD2 80.00 usec
PLW2 13.00000000 W
PLW12 0.09953100 W
PLW13 0.06370000 W
F2 - Processing parameters
SI 32768
SF 125.7829330 MHz
WDW EM
SSB 0
LB 1.00 Hz
GB 0
PC 1.40

1aae

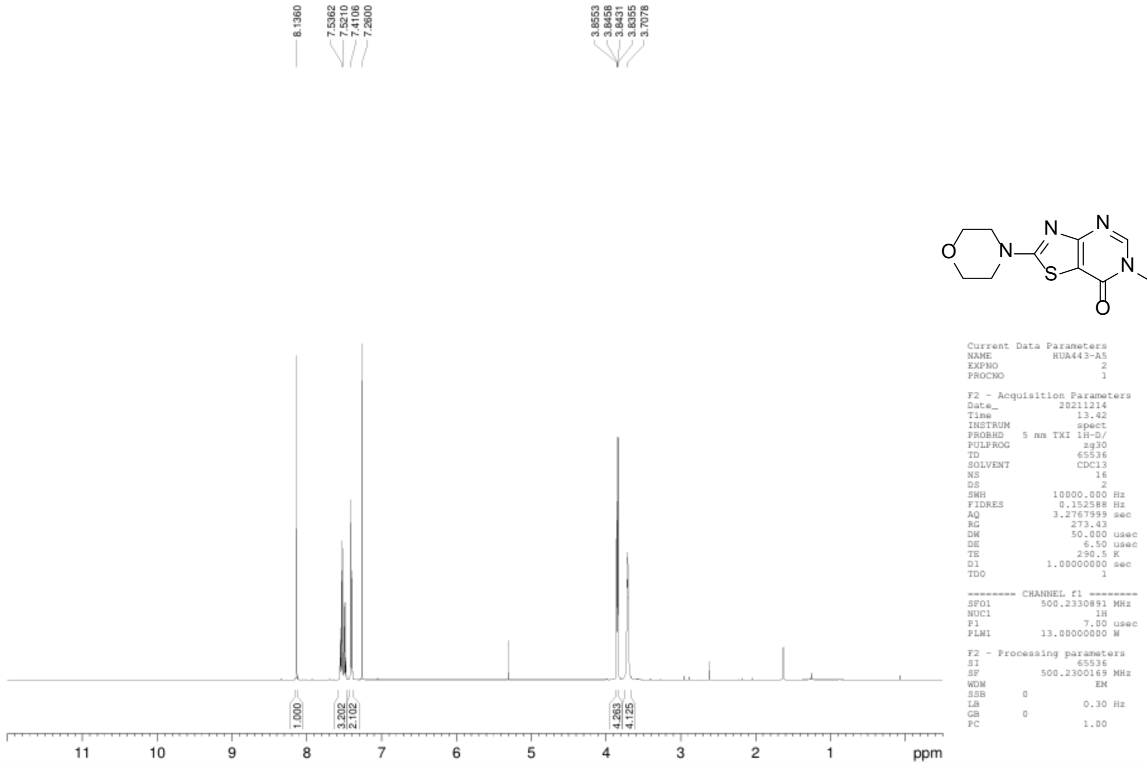
8.1360
7.5362
7.5210
7.4106
7.2600
3.8553
3.8458
3.8431
3.8355
3.7078
1.000
3.202
2.102
4.263
4.125
11 10 9 8 7 6 5 4 3 2 1 ppm
N
N
O
N
S
N
O
N
Current Data Parameters
NAME HUA443-A5
EXPNO 2
PROCNO 1
F2 - Acquisition Parameters
Date_ 20211214
Time 13.42
INSTRUM spect
PROBHD 5 mm TXI 1H-D/
PULPROG zg30
TD 65536
SOLVENT CDCl3
NS 16
DS 2
SWH 10000.000 Hz
FIDRES 0.152588 Hz
AQ 3.2767999 sec
RG 273.43
DW 50.000 usec
DE 6.50 usec
TE 290.5 K
D1 1.00000000 sec
TD0 1
======== CHANNEL f1 ========
SFO1 500.2330891 MHz
NUC1 1H
P1 7.00 usec
PLW1 13.00000000 W
F2 - Processing parameters
SI 65536
SF 500.2300169 MHz
WDW EM
SSB 0
LB 0.30 Hz
GB 0
PC 1.00

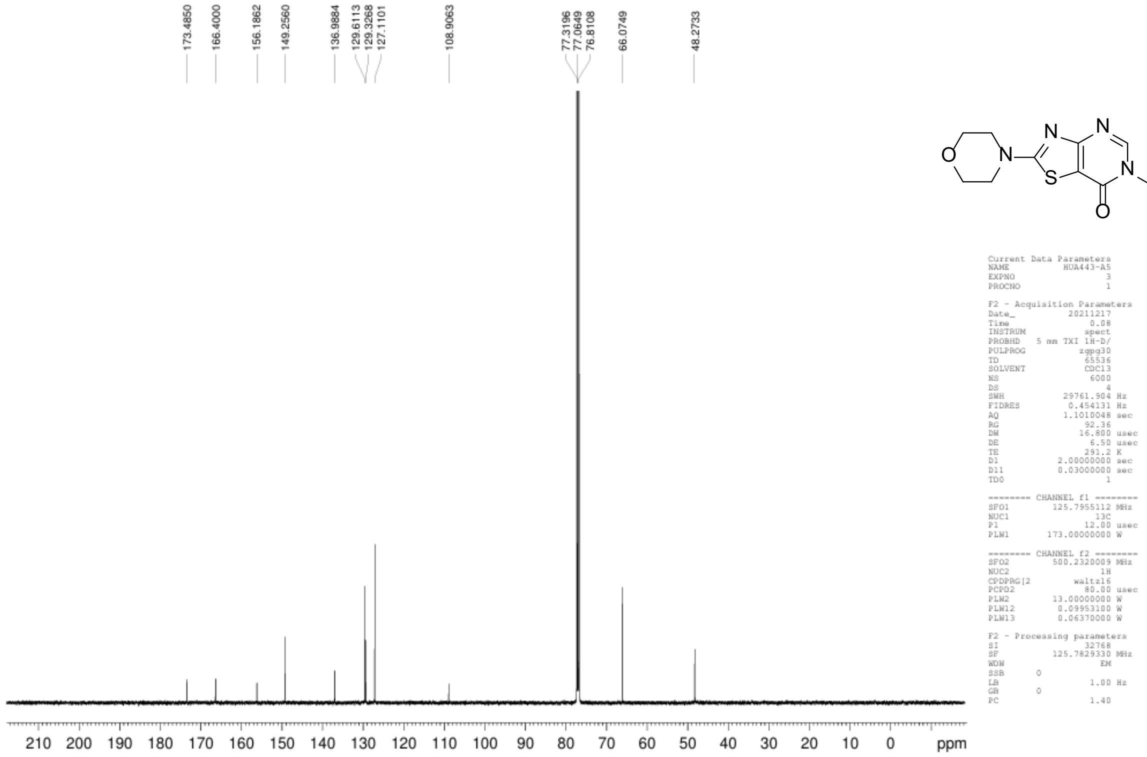
173.4850
166.4000
156.1862
149.2560
136.9884
129.6113
129.3268
127.1101
108.9063
77.3196
77.0649
76.8108
66.0749
48.2733
210 200 190 180 170 160 150 140 130 120 110 100 90 80 70 60 50 40 30 20 10 0 ppm
N
N
O
N
S
N
O
N
Current Data Parameters
NAME HUA443-A5
EXPNO 3
PROCNO 1
F2 - Acquisition Parameters
Date_ 20211217
Time 0.08
INSTRUM spect
PROBHD 5 mm TXI 1H-D/
PULPROG zgpg30
TD 65536
SOLVENT CDCl3
NS 6000
DS 4
SWH 29761.904 Hz
FIDRES 0.454131 Hz
AQ 1.1010048 sec
RG 92.36
DW 16.800 usec
DE 6.50 usec
TE 291.2 K
D1 2.00000000 sec
D11 0.03000000 sec
TD0 1
======== CHANNEL f1 ========
SFO1 125.7955112 MHz
NUC1 13C
P1 12.00 usec
PLW1 173.00000000 W
======== CHANNEL f2 ========
SFO2 500.2320009 MHz
NUC2 1H
CPDPRG[2 waltz16
PCPD2 80.00 usec
PLW2 13.00000000 W
PLW12 0.09953100 W
PLW13 0.06370000 W
F2 - Processing parameters
SI 32768
SF 125.7829330 MHz
WDW EM
SSB 0
LB 1.00 Hz
GB 0
PC 1.40

1aaf

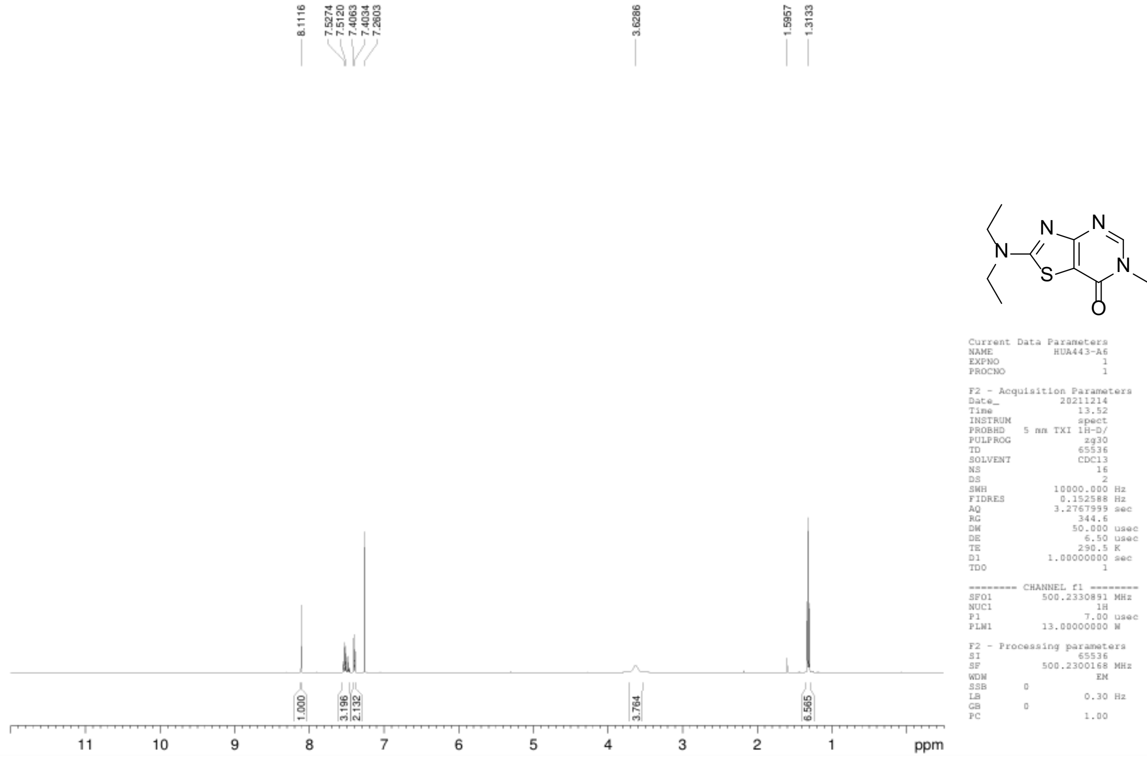
8.1116
7.5274
7.5120
7.4063
7.4034
7.2603
3.6286
1.5957
1.3133
1.000
3.196
2.132
3.764
6.565
11 10 9 8 7 6 5 4 3 2 1 ppm
N
N
N
S
N
O
Current Data Parameters
NAME HUA443-A6
EXPNO 1
PROCNO 1
F2 - Acquisition Parameters
Date_ 20211214
Time 13.52
INSTRUM spect
PROBHD 5 mm TXI 1H-D/
PULPROG zg30
TD 65536
SOLVENT CDCl3
NS 16
DS 2
SWH 10000.000 Hz
FIDRES 0.152588 Hz
AQ 3.2767999 sec
RG 344.6
DW 50.000 usec
DE 6.50 usec
TE 290.5 K
D1 1.00000000 sec
TD0 1
======== CHANNEL f1 ========
SFO1 500.2330891 MHz
NUC1 1H
P1 7.00 usec
PLW1 13.00000000 W
F2 - Processing parameters
SI 65536
SF 500.2300168 MHz
WDW EM
SSB 0
LB 0.30 Hz
GB 0
PC 1.00

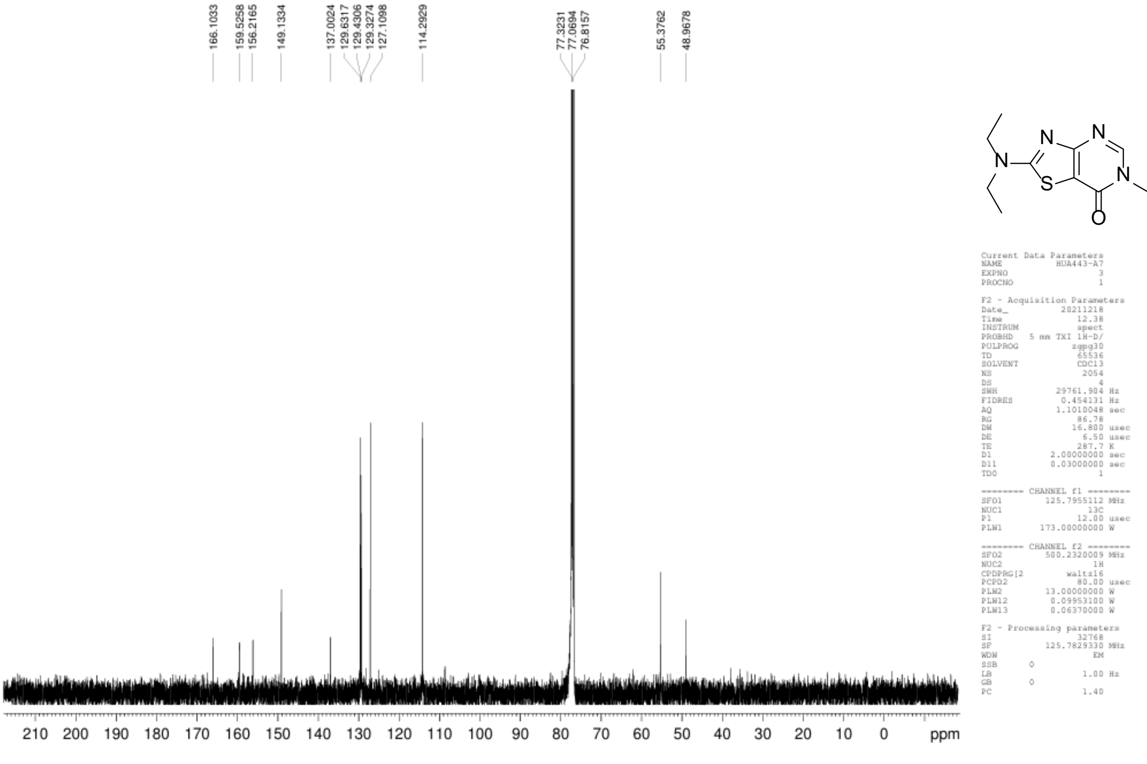
166.1033
159.5258
156.2165
149.1334
137.0024
129.6317
129.4306
129.3274
127.1098
114.2929
77.3231
77.0694
76.8157
55.3762
48.9678
210 200 190 180 170 160 150 140 130 120 110 100 90 80 70 60 50 40 30 20 10 0 ppm
N
N
N
S
N
O
Current Data Parameters
NAME HUA443-A7
EXPNO 3
PROCNO 1
F2 - Acquisition Parameters
Date_ 20211218
Time 12.38
INSTRUM spect
PROBHD 5 mm TXI 1H-D/
PULPROG zgpg30
TD 65536
SOLVENT CDCl3
NS 2054
DS 4
SWH 29761.904 Hz
FIDRES 0.454131 Hz
AQ 1.1010048 sec
RG 86.78
DW 16.800 usec
DE 6.50 usec
TE 287.7 K
D1 2.00000000 sec
D11 0.03000000 sec
TD0 1
======== CHANNEL f1 ========
SFO1 125.7955112 MHz
NUC1 13C
P1 12.00 usec
PLW1 173.00000000 W
======== CHANNEL f2 ========
SFO2 500.2320009 MHz
NUC2 1H
CPDPRG[2 waltz16
PCPD2 80.00 usec
PLW2 13.00000000 W
PLW12 0.09953100 W
PLW13 0.06370000 W
F2 - Processing parameters
SI 32768
SF 125.7829330 MHz
WDW EM
SSB 0
LB 1.00 Hz
GB 0
PC 1.40

1aag

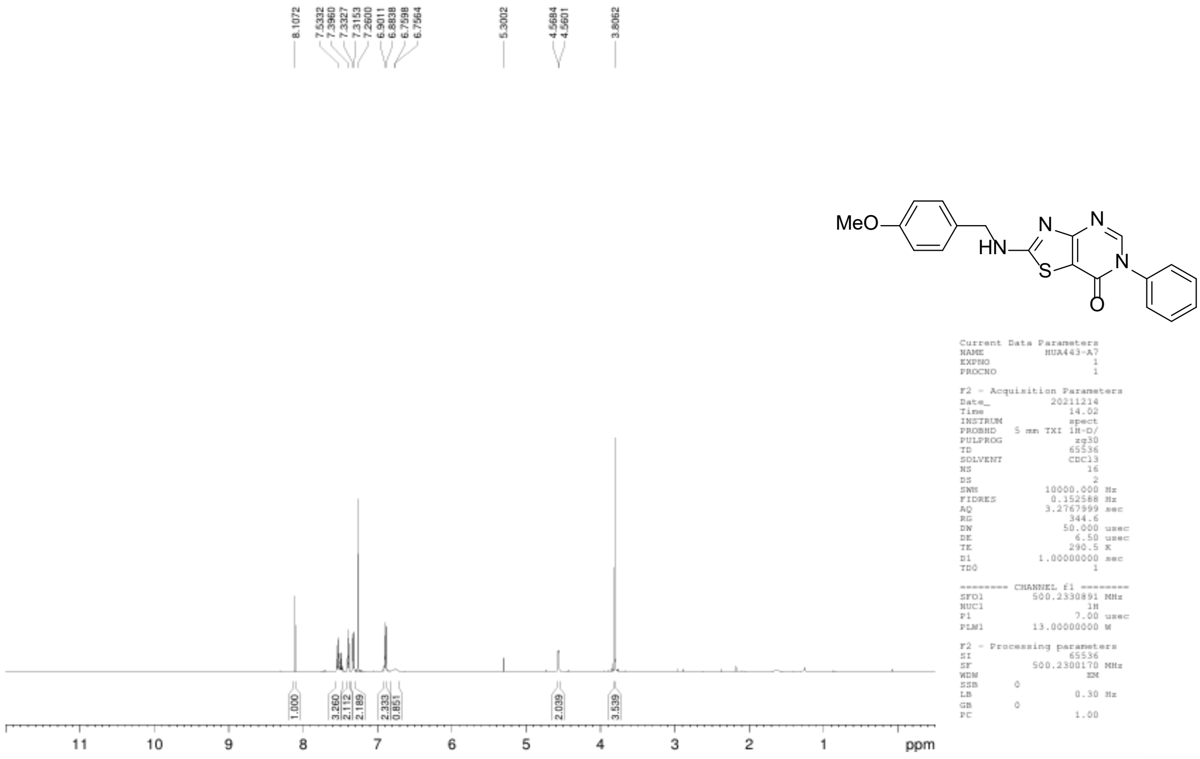

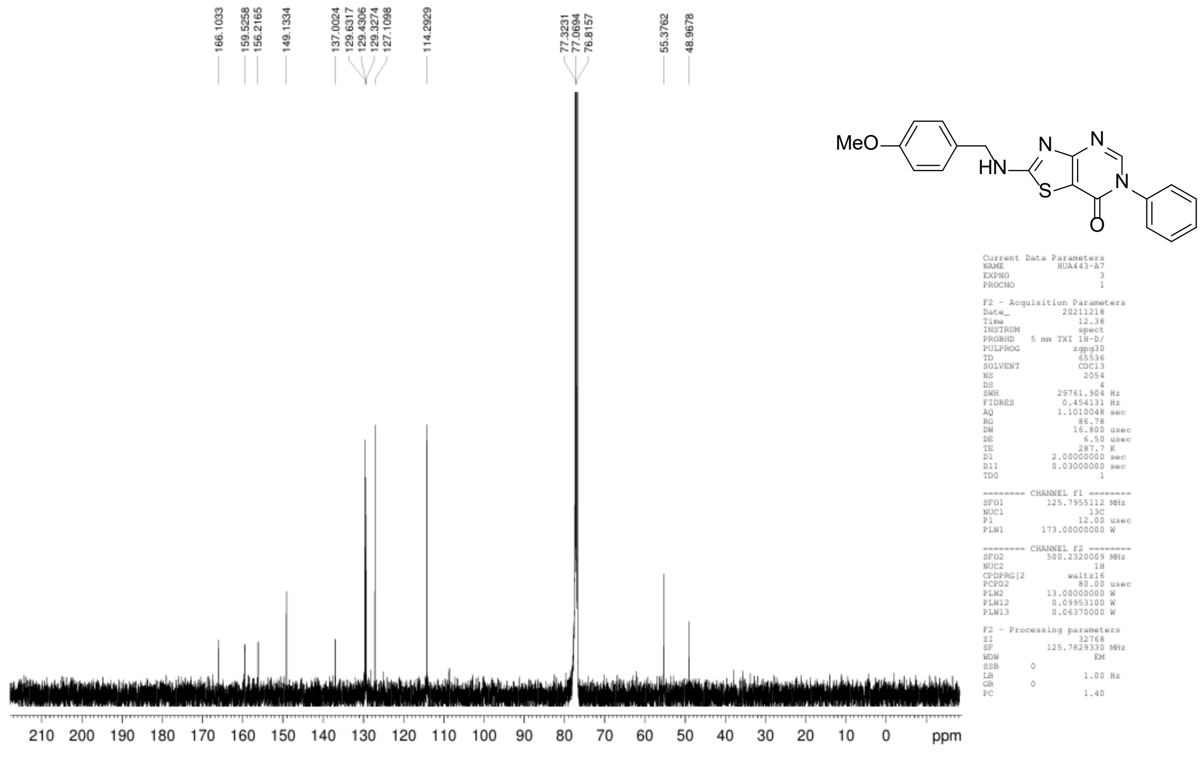

1aah

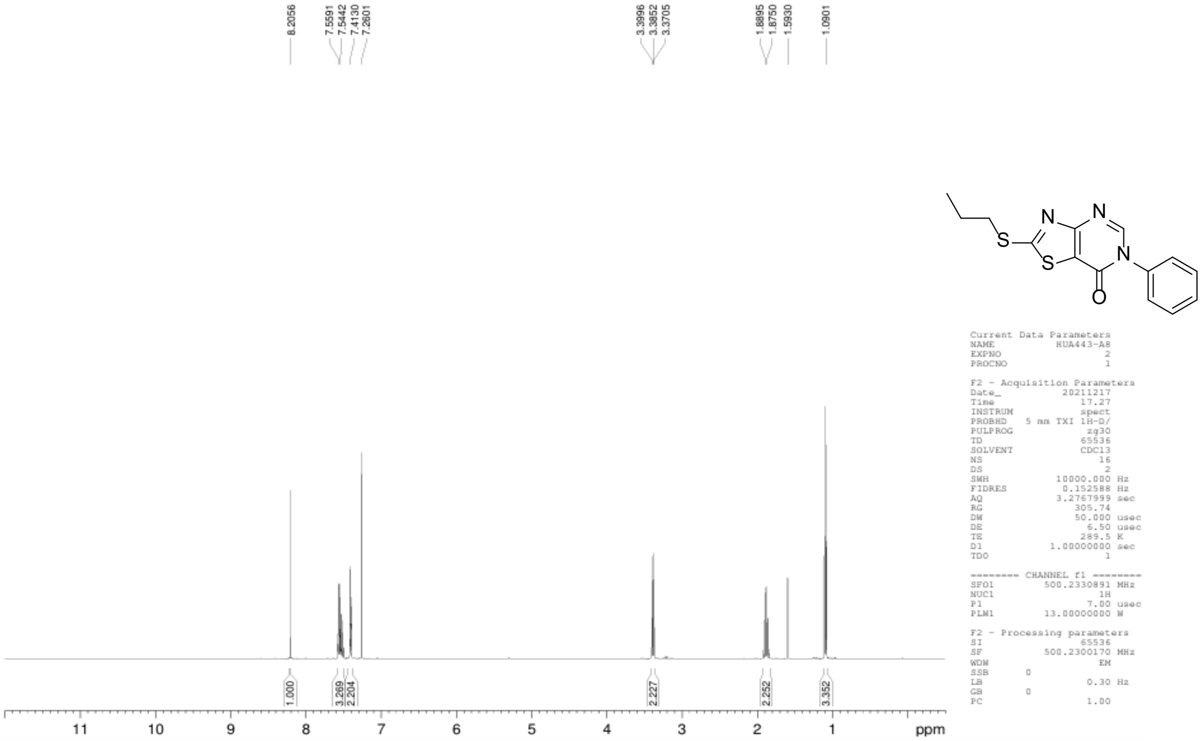
8.2056
7.5591
7.5442
7.4130
7.2601
3.3996
3.3852
3.3705
1.8895
1.8750
1.5930
1.0901
N
N
S
N
S
O
1.000
3.269
2.204
2.227
2.252
3.352
11 10 9 8 7 6 5 4 3 2 1 ppm
Current Data Parameters
NAME HUA443-A8
EXPNO 2
PROCNO 1
F2 - Acquisition Parameters
Date_ 20211217
Time 17.27
INSTRUM spect
PROBHD 5 mm TXI 1H-D/
PULPROG zg30
TD 65536
SOLVENT CDCl3
NS 16
DS 2
SWH 10000.000 Hz
FIDRES 0.152588 Hz
AQ 3.2767999 sec
RG 305.74
DW 50.000 usec
DE 6.50 usec
TE 289.5 K
D1 1.00000000 sec
TD0 1
======== CHANNEL f1 ========
SFO1 500.2330891 MHz
NUC1 1H
P1 7.00 usec
PLW1 13.00000000 W
F2 - Processing parameters
SI 65536
SF 500.2300170 MHz
WDW EM
SSB 0
LB 0.30 Hz
GB 0
PC 1.00

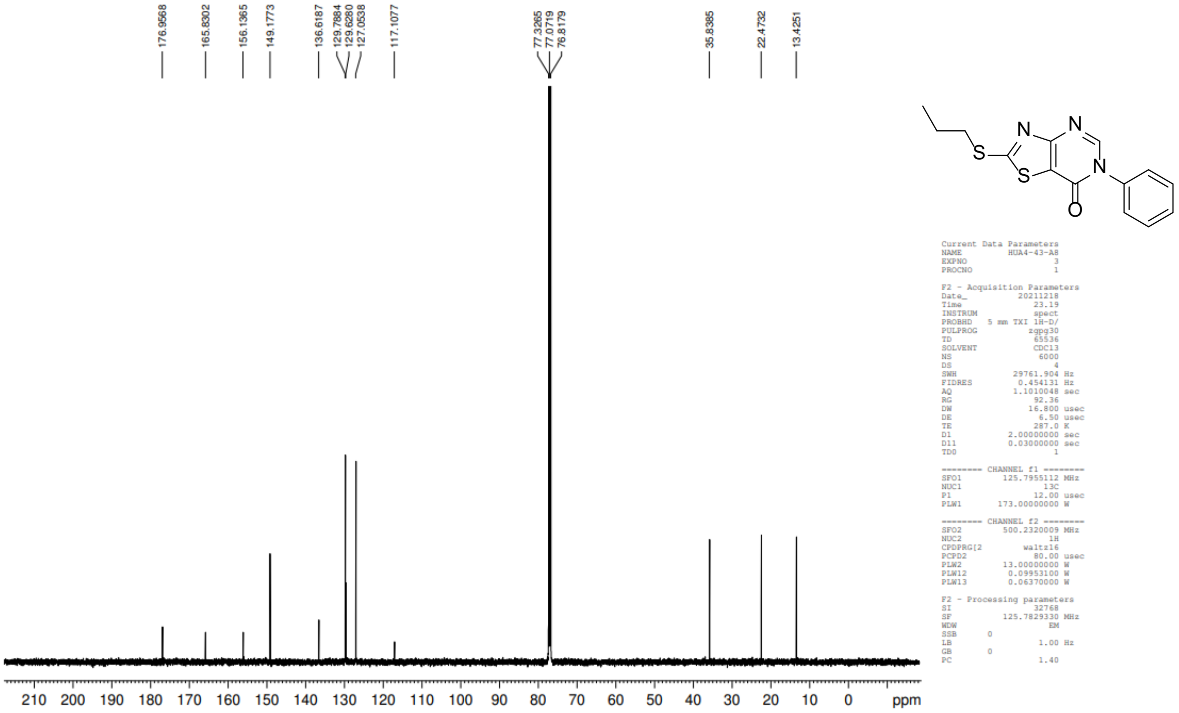
176.9568
165.8302
156.1365
149.1773
136.6187
129.7884
129.6280
127.0538
117.1077
77.3265
77.0719
76.8179
35.8385
22.4732
13.4251
N
N
S
N
S
O
210 200 190 180 170 160 150 140 130 120 110 100 90 80 70 60 50 40 30 20 10 0 ppm
Current Data Parameters
NAME HUA4-43-A8
EXPNO 3
PROCNO 1
F2 - Acquisition Parameters
Date_ 20211218
Time 23.19
INSTRUM spect
PROBHD 5 mm TXI 1H-D/
PULPROG zgpg30
TD 65536
SOLVENT CDCl3
NS 6000
DS 4
SWH 29761.904 Hz
FIDRES 0.454131 Hz
AQ 1.1010048 sec
RG 92.36
DW 16.800 usec
DE 6.50 usec
TE 287.0 K
D1 2.00000000 sec
D11 0.03000000 sec
TD0 1
======== CHANNEL f1 ========
SFO1 125.7955112 MHz
NUC1 13C
P1 12.00 usec
PLW1 173.00000000 W
======== CHANNEL f2 ========
SFO2 500.2320009 MHz
NUC2 1H
CPDPRG[2 waltz16
PCPD2 80.00 usec
PLW2 13.00000000 W
PLW12 0.09953100 W
PLW13 0.06370000 W
F2 - Processing parameters
SI 32768
SF 125.7829330 MHz
WDW EM
SSB 0
LB 1.00 Hz
GB 0
PC 1.40

1aai

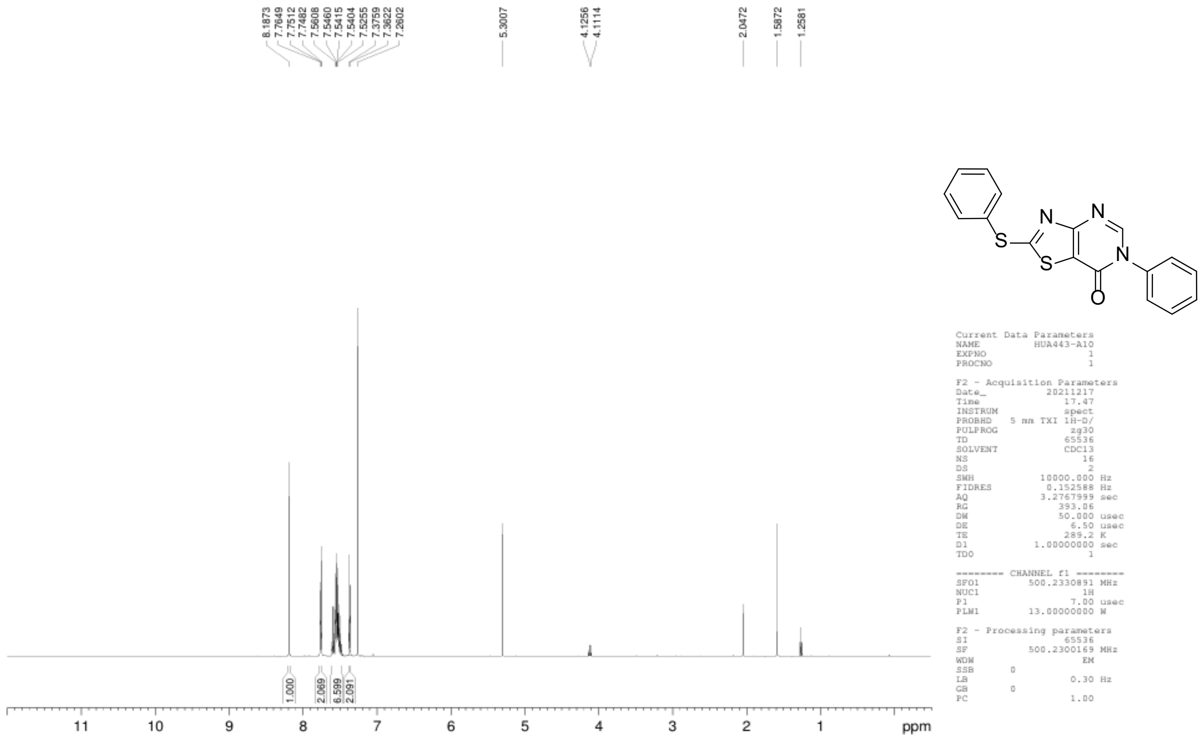
8.1873
7.7649
7.7512
7.7482
7.5608
7.5460
7.5415
7.5404
7.5255
7.3759
7.3622
7.2602
5.3007
4.1256
4.1114
2.0472
1.5872
1.2581
N
N
S
S
N
O
Current Data Parameters
NAME HUA443-A10
EXPNO 1
PROCNO 1
F2 - Acquisition Parameters
Date_ 20211217
Time 17.47
INSTRUM spect
PROBHD 5 mm TXI 1H-D/
PULPROG zg30
TD 65536
SOLVENT CDCl3
NS 16
DS 2
SWH 10000.000 Hz
FIDRES 0.152588 Hz
AQ 3.2767999 sec
RG 393.06
DW 50.000 usec
DE 6.50 usec
TE 289.2 K
D1 1.00000000 sec
TD0 1
======== CHANNEL f1 ========
SFO1 500.2330891 MHz
NUC1 1H
P1 7.00 usec
PLW1 13.00000000 W
F2 - Processing parameters
SI 65536
SF 500.2300169 MHz
WDW EM
SSB 0
LB 0.30 Hz
GB 0
PC 1.00
1.000
2.069
6.599
2.091
11
10
9
8
7
6
5
4
3
2
1
ppm

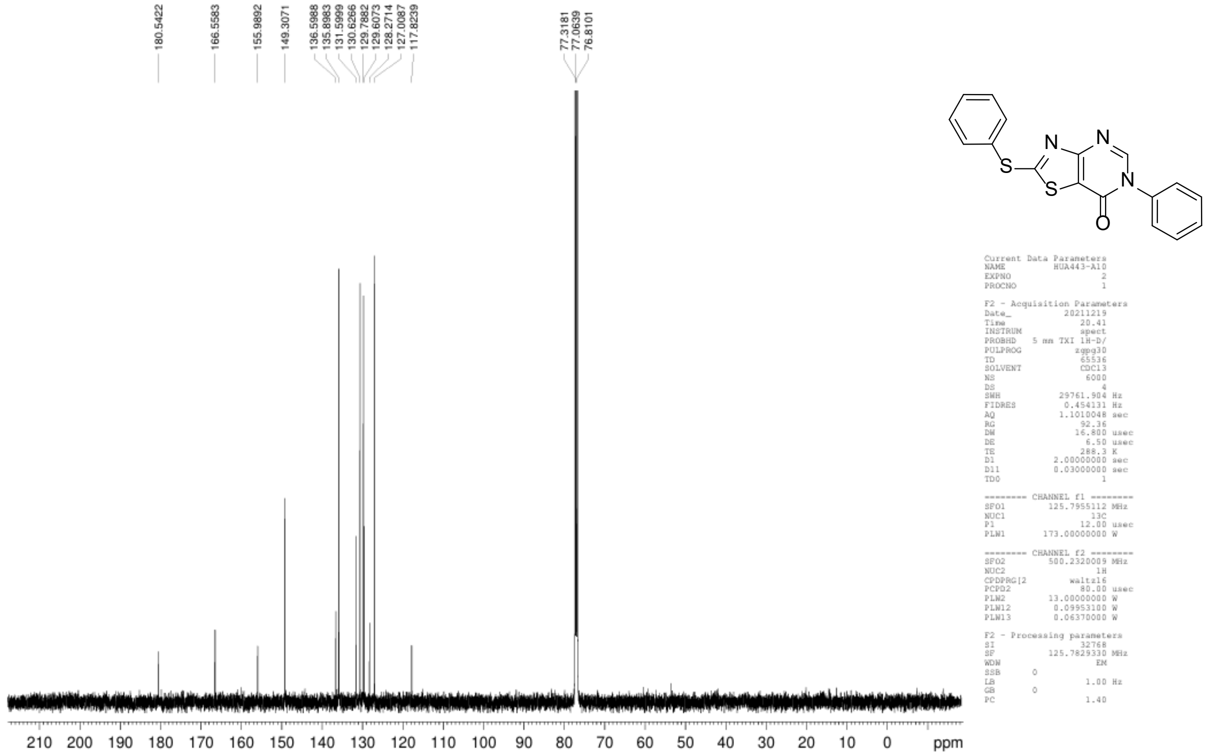
180.5422
166.5583
155.9892
149.3071
136.5988
135.8983
131.5999
130.6266
129.7882
129.6073
128.2714
127.0087
117.8239
77.3181
77.0639
76.8101
N
N
S
S
N
O
Current Data Parameters
NAME HUA443-A10
EXPNO 2
PROCNO 1
F2 - Acquisition Parameters
Date_ 20211219
Time 20.41
INSTRUM spect
PROBHD 5 mm TXI 1H-D/
PULPROG zgpg30
TD 65536
SOLVENT CDCl3
NS 6000
DS 4
SWH 29761.904 Hz
FIDRES 0.454131 Hz
AQ 1.1010048 sec
RG 92.36
DW 16.800 usec
DE 6.50 usec
TE 288.3 K
D1 2.00000000 sec
D11 0.03000000 sec
TD0 1
======== CHANNEL f1 ========
SFO1 125.7955112 MHz
NUC1 13C
P1 12.00 usec
PLW1 173.00000000 W
======== CHANNEL f2 ========
SFO2 500.2320009 MHz
NUC2 1H
CPDPRG[2 waltz16
PCPD2 80.00 usec
PLW2 13.00000000 W
PLW12 0.09953100 W
PLW13 0.06370000 W
F2 - Processing parameters
SI 32768
SF 125.7829330 MHz
WDW EM
SSB 0
LB 1.00 Hz
GB 0
PC 1.40
210
200
190
180
170
160
150
140
130
120
110
100
90
80
70
60
50
40
30
20
10
0
ppm

1aaj

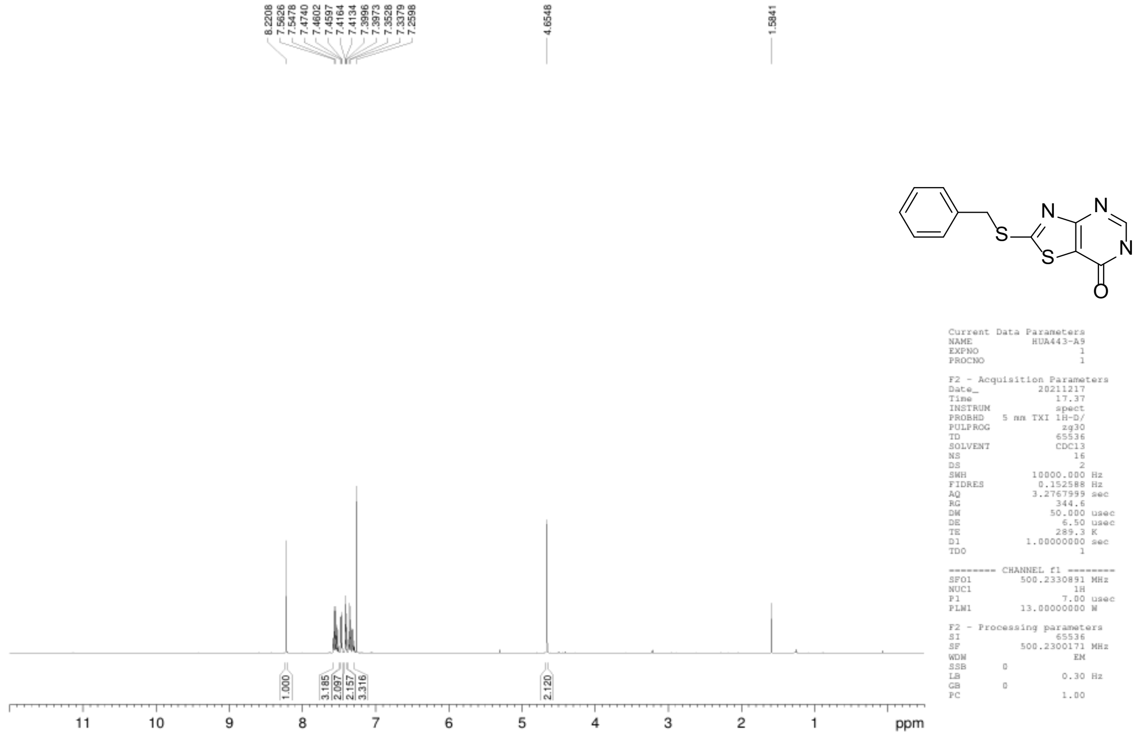
8.2208
7.5626
7.5478
7.4740
7.4602
7.4597
7.4164
7.4134
7.3996
7.3973
7.3528
7.3379
7.2598
4.6548
1.5841
1.000
3.185
2.097
2.157
3.316
2.120
11 10 9 8 7 6 5 4 3 2 1 ppm
N
N
S
S
N
O
Current Data Parameters
NAME HUA443-A9
EXPNO 1
PROCNO 1
F2 - Acquisition Parameters
Date_ 20211217
Time 17.37
INSTRUM spect
PROBHD 5 mm TXI 1H-D/
PULPROG zg30
TD 65536
SOLVENT CDCl3
NS 16
DS 2
SWH 10000.000 Hz
FIDRES 0.152588 Hz
AQ 3.2767999 sec
RG 344.6
DW 50.000 usec
DE 6.50 usec
TE 289.3 K
D1 1.00000000 sec
TD0 1
======== CHANNEL f1 ========
SFO1 500.2330891 MHz
NUC1 1H
P1 7.00 usec
PLW1 13.00000000 W
F2 - Processing parameters
SI 65536
SF 500.2300171 MHz
WDW EM
SSB 0
LB 0.30 Hz
GB 0
PC 1.00

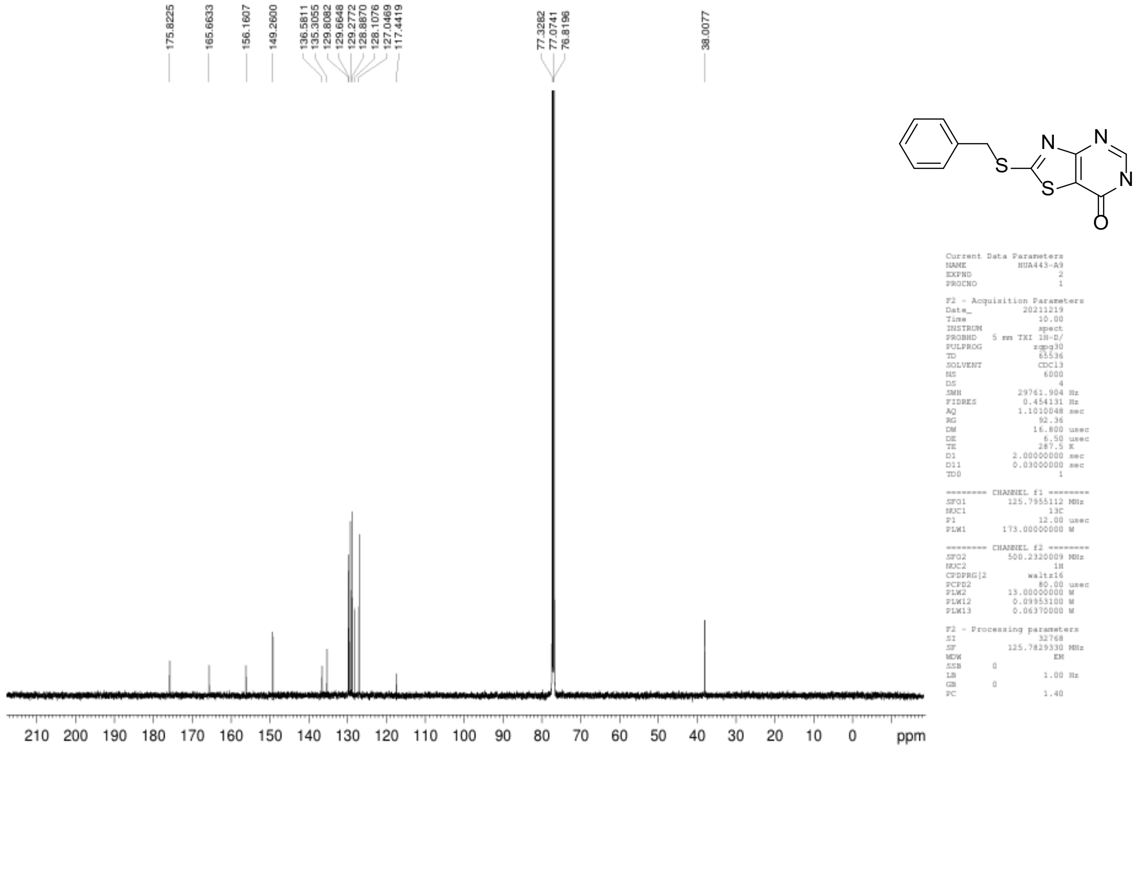
175.8225
165.6633
156.1607
149.2600
136.5811
135.3055
129.8082
129.6648
129.2772
128.8870
128.1076
127.0469
117.4419
77.3282
77.0741
76.8196
38.0077
210 200 190 180 170 160 150 140 130 120 110 100 90 80 70 60 50 40 30 20 10 0 ppm
N
N
S
S
N
O
Current Data Parameters
NAME HUA443-A9
EXPNO 2
PROCNO 1
F2 - Acquisition Parameters
Date_ 20211219
Time 10.00
INSTRUM spect
PROBHD 5 mm TXI 1H-D/
PULPROG zgpg30
TD 65536
SOLVENT CDCl3
NS 6000
DS 4
SWH 29761.904 Hz
FIDRES 0.454131 Hz
AQ 1.1010048 sec
RG 92.36
DW 16.800 usec
DE 6.50 usec
TE 287.5 K
D1 2.00000000 sec
D11 0.03000000 sec
TD0 1
======== CHANNEL f1 ========
SFO1 125.7955112 MHz
NUC1 13C
P1 12.00 usec
PLW1 173.00000000 W
======== CHANNEL f2 ========
SFO2 500.2320009 MHz
NUC2 1H
CPDPRG[2 waltz16
PCPD2 80.00 usec
PLW2 13.00000000 W
PLW12 0.09953100 W
PLW13 0.06370000 W
F2 - Processing parameters
SI 32768
SF 125.7829330 MHz
WDW EM
SSB 0
LB 1.00 Hz
GB 0
PC 1.40

1aba

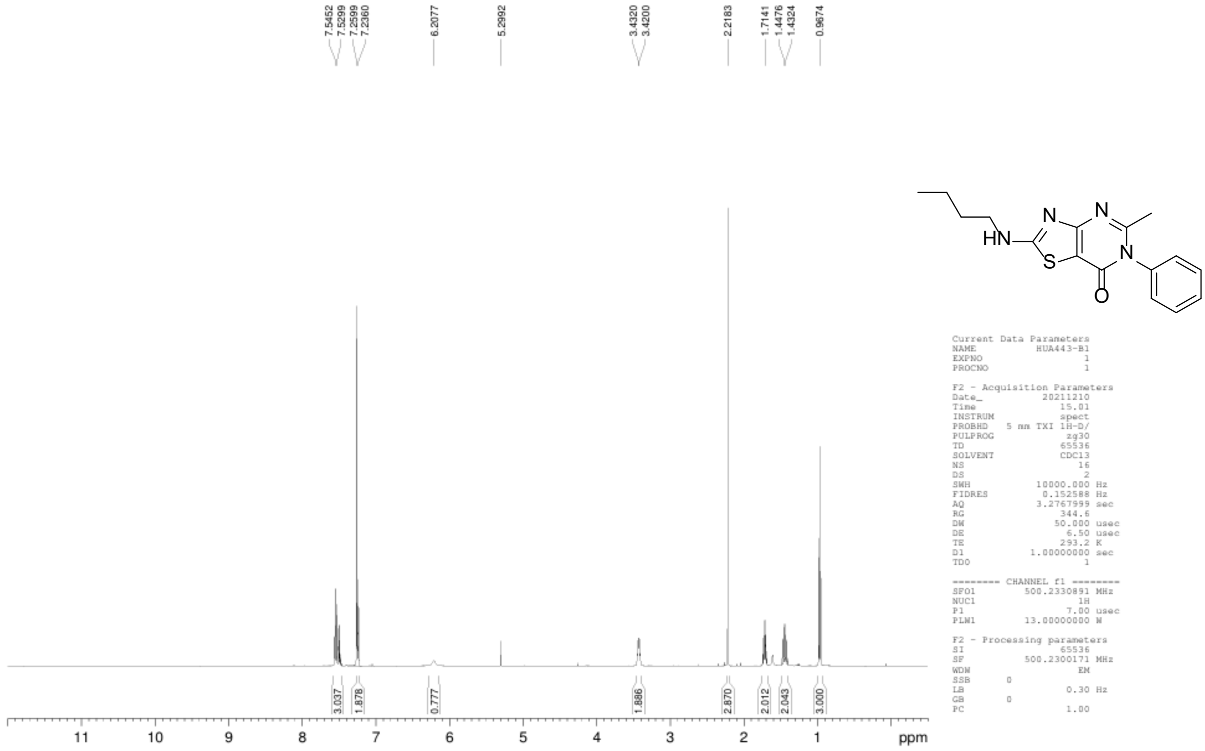
7.5452
7.5299
7.2599
7.2360
6.2077
5.2992
3.4320
3.4200
2.2183
1.7141
1.4476
1.4324
0.9674
3.037
1.878
0.777
1.886
2.870
2.012
2.043
3.000
11 10 9 8 7 6 5 4 3 2 1 ppm
HN
N
N
S
N
O
Current Data Parameters
NAME HUA443-B1
EXPNO 1
PROCNO 1
F2 - Acquisition Parameters
Date_ 20211210
Time 15.01
INSTRUM spect
PROBHD 5 mm TXI 1H-D/
PULPROG zg30
TD 65536
SOLVENT CDCl3
NS 16
DS 2
SWH 10000.000 Hz
FIDRES 0.152588 Hz
AQ 3.2767999 sec
RG 344.6
DW 50.000 usec
DE 6.50 usec
TE 293.2 K
D1 1.00000000 sec
TD0 1
======== CHANNEL f1 ========
SFO1 500.2330891 MHz
NUC1 1H
P1 7.00 usec
PLW1 13.00000000 W
F2 - Processing parameters
SI 65536
SF 500.2300171 MHz
WDW EM
SSB 0
LB 0.30 Hz
GB 0
PC 1.00

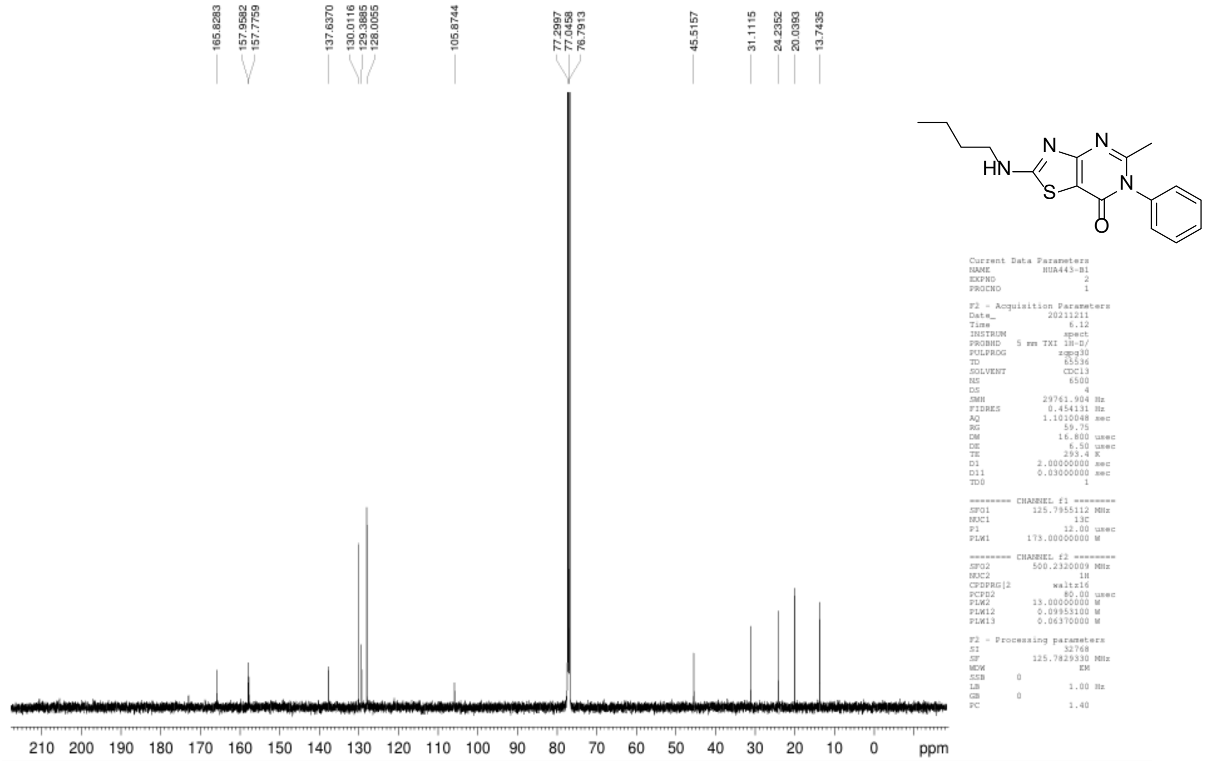
165.8283
157.9582
157.7759
137.6370
130.0116
129.3885
128.0055
105.8744
77.2997
77.0458
76.7913
45.5157
31.1115
24.2352
20.0393
13.7435
210 200 190 180 170 160 150 140 130 120 110 100 90 80 70 60 50 40 30 20 10 0 ppm
HN
N
N
S
N
O
Current Data Parameters
NAME HUA443-B1
EXPNO 2
PROCNO 1
F2 - Acquisition Parameters
Date_ 20211211
Time 6.12
INSTRUM spect
PROBHD 5 mm TXI 1H-D/
PULPROG zgpg30
TD 65536
SOLVENT CDCl3
NS 6500
DS 4
SWH 29761.904 Hz
FIDRES 0.454131 Hz
AQ 1.1010048 sec
RG 59.75
DW 16.800 usec
DE 6.50 usec
TE 293.4 K
D1 2.00000000 sec
D11 0.03000000 sec
TD0 1
======== CHANNEL f1 ========
SFO1 125.7955112 MHz
NUC1 13C
P1 12.00 usec
PLW1 173.00000000 W
======== CHANNEL f2 ========
SFO2 500.2320009 MHz
NUC2 1H
CPDPRG[2 waltz16
PCPD2 80.00 usec
PLW2 13.00000000 W
PLW12 0.09953100 W
PLW13 0.06370000 W
F2 - Processing parameters
SI 32768
SF 125.7829330 MHz
WDW EM
SSB 0
LB 1.00 Hz
GB 0
PC 1.40

1abb

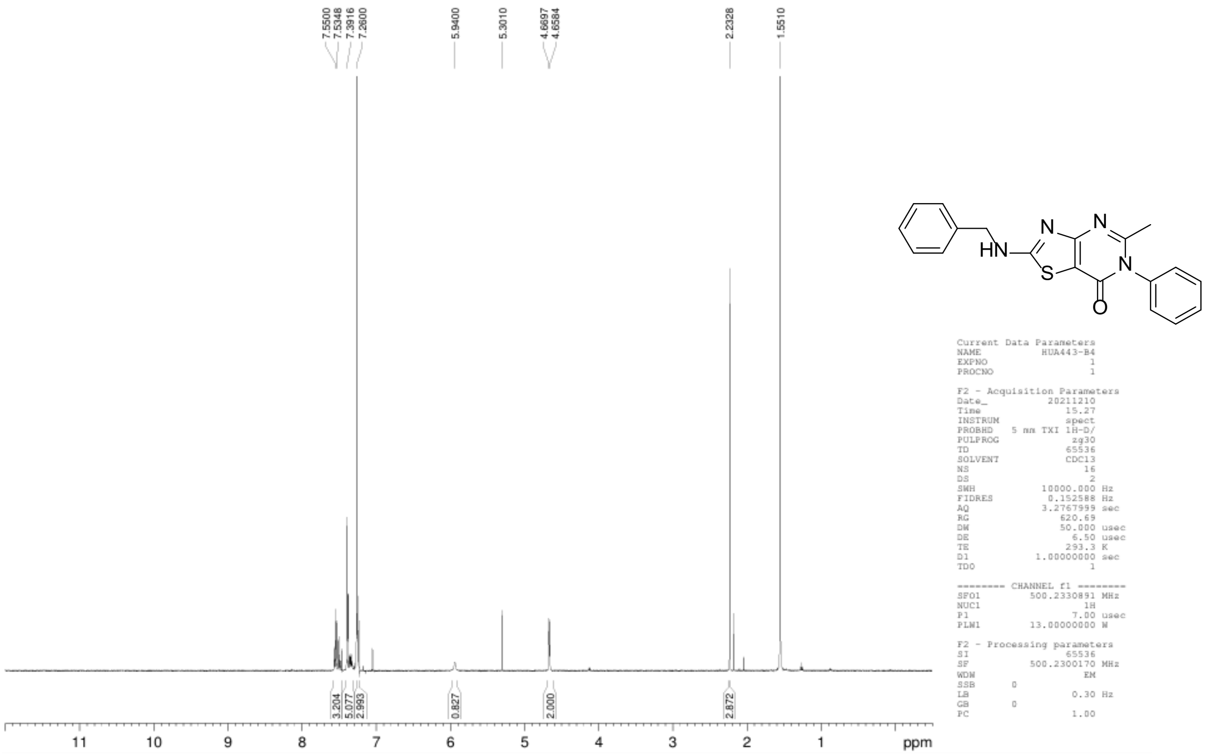

7.5500
7.5348
7.3916
7.2600
5.9400
5.3010
4.6697
4.6584
2.2328
1.5510
HN
N
N
N
S
O
Current Data Parameters
NAME HUA443-B4
EXPNO 1
PROCNO 1
F2 - Acquisition Parameters
Date_ 20211210
Time 15.27
INSTRUM spect
PROBHD 5 mm TXI 1H-D/
PULPROG zg30
TD 65536
SOLVENT CDCl3
NS 16
DS 2
SWH 10000.000 Hz
FIDRES 0.152588 Hz
AQ 3.2767999 sec
RG 620.69
DW 50.000 usec
DE 6.50 usec
TE 293.3 K
D1 1.00000000 sec
TD0 1
======== CHANNEL f1 ========
SFO1 500.2330891 MHz
NUC1 1H
P1 7.00 usec
PLW1 13.00000000 W
F2 - Processing parameters
SI 65536
SF 500.2300170 MHz
WDW EM
SSB 0
LB 0.30 Hz
GB 0
PC 1.00
3.204
5.077
2.993
0.827
2.000
2.872
11
10
9
8
7
6
5
4
3
2
1
ppm

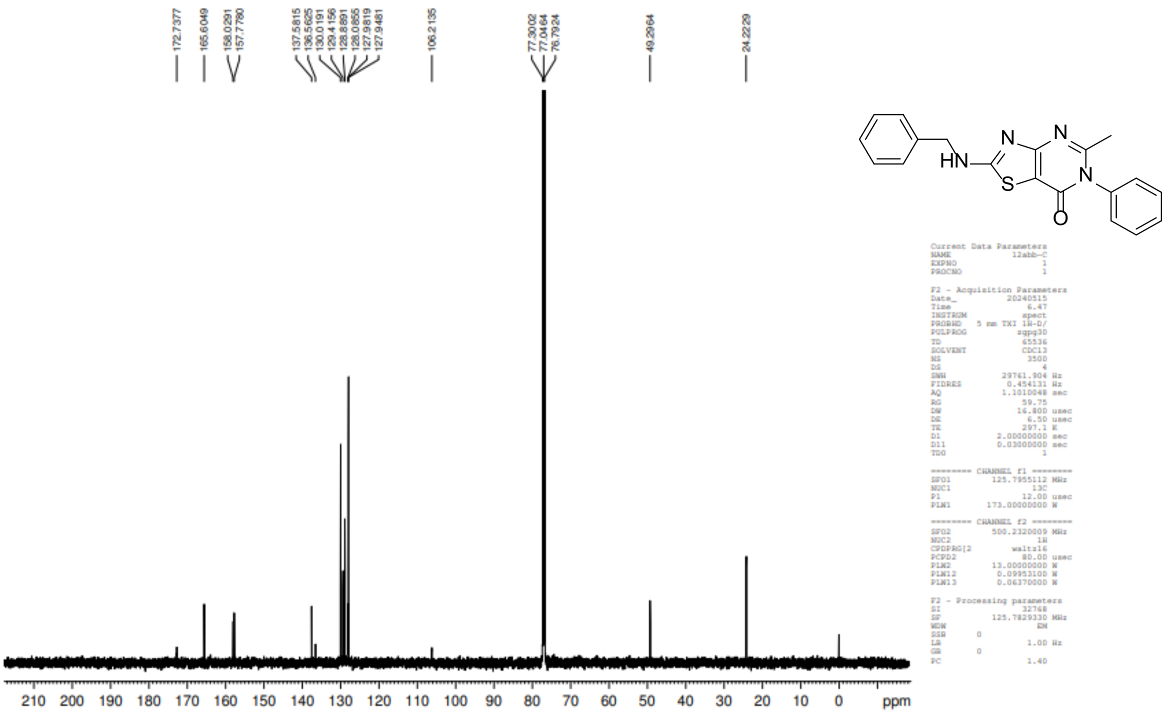

172.7377
165.6049
158.0291
157.7780
137.5815
136.5625
130.0191
129.4156
128.8891
128.0855
127.9819
127.9481
106.2135
77.3002
77.0464
76.7924
49.2964
24.2229
HN
N
N
N
S
O
Current Data Parameters
NAME 12abb-C
EXPNO 1
PROCNO 1
F2 - Acquisition Parameters
Date_ 20240515
Time 6.47
INSTRUM spect
PROBHD 5 mm TXI 1H-D/
PULPROG zgpg30
TD 65536
SOLVENT CDCl3
NS 3500
DS 4
SWH 29761.904 Hz
FIDRES 0.454131 Hz
AQ 1.1010048 sec
RG 59.75
DW 16.800 usec
DE 6.50 usec
TE 297.1 K
D1 2.00000000 sec
D11 0.03000000 sec
TD0 1
======== CHANNEL f1 ========
SFO1 125.7955112 MHz
NUC1 13C
P1 12.00 usec
PLW1 173.00000000 W
======== CHANNEL f2 ========
SFO2 500.2320009 MHz
NUC2 1H
CPDPRG[2 waltz16
PCPD2 80.00 usec
PLW2 13.00000000 W
PLW12 0.09953100 W
PLW13 0.06370000 W
F2 - Processing parameters
SI 32768
SF 125.7829330 MHz
WDW EM
SSB 0
LB 1.00 Hz
GB 0
PC 1.40
210
200
190
180
170
160
150
140
130
120
110
100
90
80
70
60
50
40
30
20
10
0
ppm

1abc

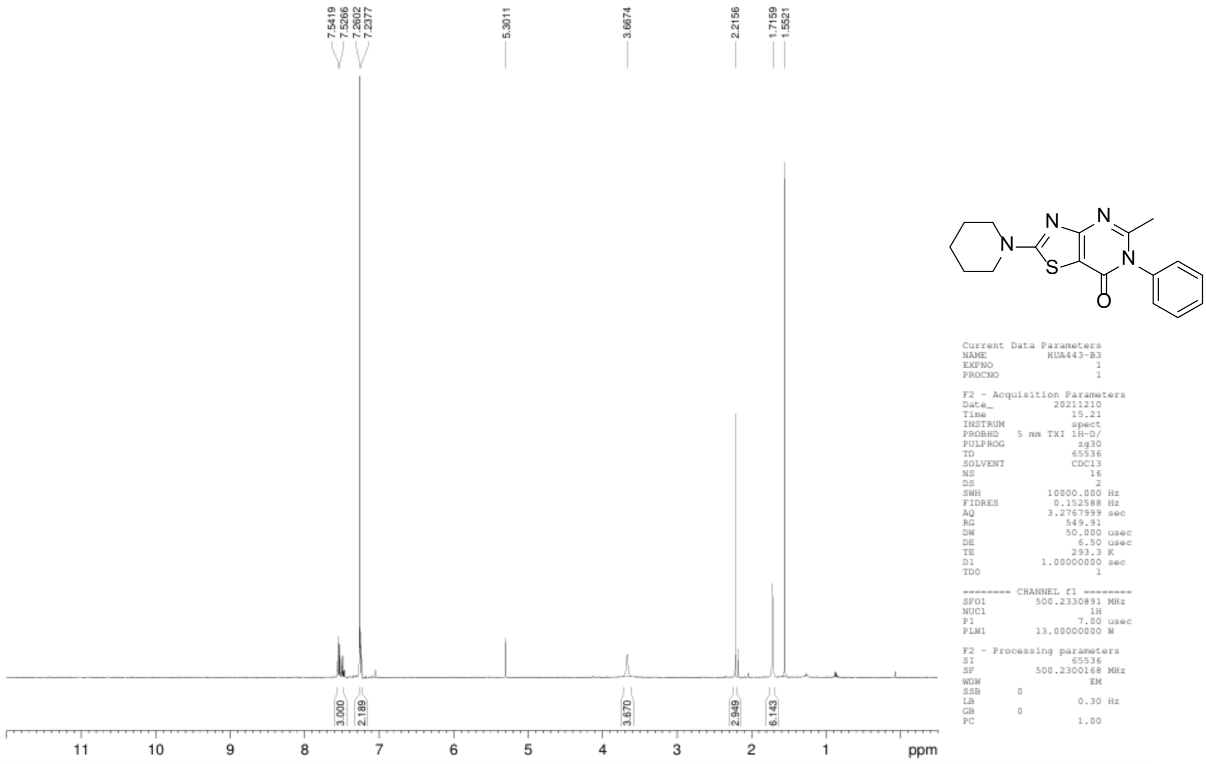
7.5419
7.5266
7.2602
7.2377
5.3011
3.6674
2.2156
1.7159
1.5521
3.000
2.189
3.670
2.949
6.143
11 10 9 8 7 6 5 4 3 2 1 ppm

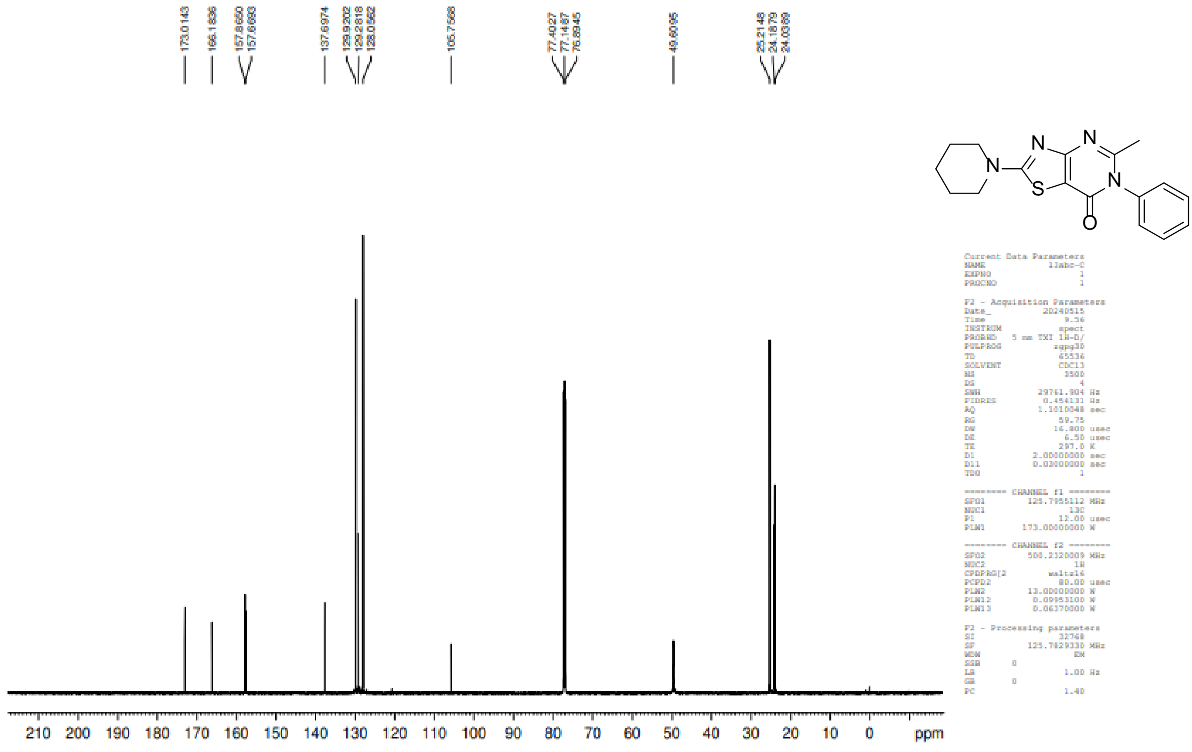
173.0143
166.1836
157.8650
157.6693
137.6974
129.9202
129.2818
128.0562
105.7568
77.4027
77.1487
76.8945
49.6095
25.2148
24.1879
24.0389
210 200 190 180 170 160 150 140 130 120 110 100 90 80 70 60 50 40 30 20 10 0 ppm

1abd

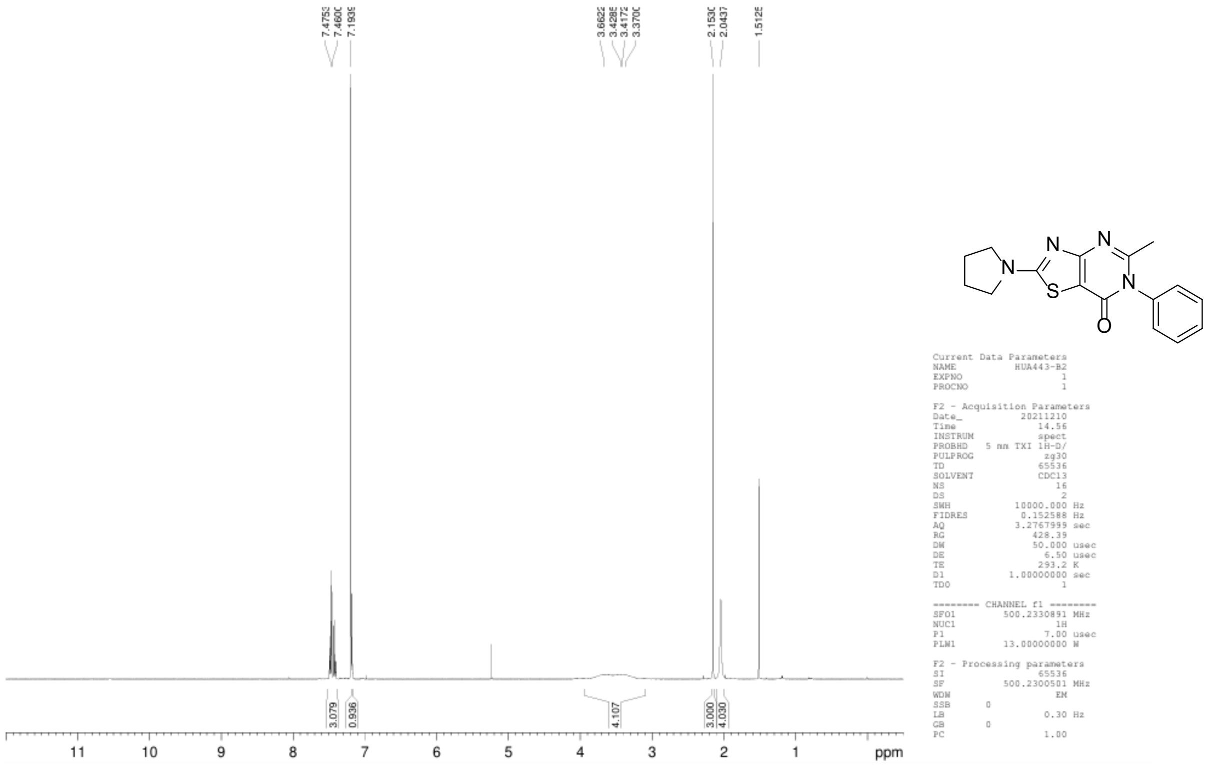

7.4753
7.4600
7.1939
3.6622
3.4285
3.4172
3.3700
2.1530
2.0437
1.5125
3.079
0.936
4.107
3.000
4.030
11 10 9 8 7 6 5 4 3 2 1 ppm
N
N
N
S
N
O
Current Data Parameters
NAME HUA443-B2
EXPNO 1
PROCNO 1
F2 - Acquisition Parameters
Date_ 20211210
Time 14.56
INSTRUM spect
PROBHD 5 mm TXI 1H-D/
PULPROG zg30
TD 65536
SOLVENT CDCl3
NS 16
DS 2
SWH 10000.000 Hz
FIDRES 0.152588 Hz
AQ 3.2767999 sec
RG 428.39
DW 50.000 usec
DE 6.50 usec
TE 293.2 K
D1 1.00000000 sec
TD0 1
======== CHANNEL f1 ========
SFO1 500.2330891 MHz
NUC1 1H
P1 7.00 usec
PLW1 13.00000000 W
F2 - Processing parameters
SI 65536
SF 500.2300501 MHz
WDW EM
SSB 0
LB 0.30 Hz
GB 0
PC 1.00

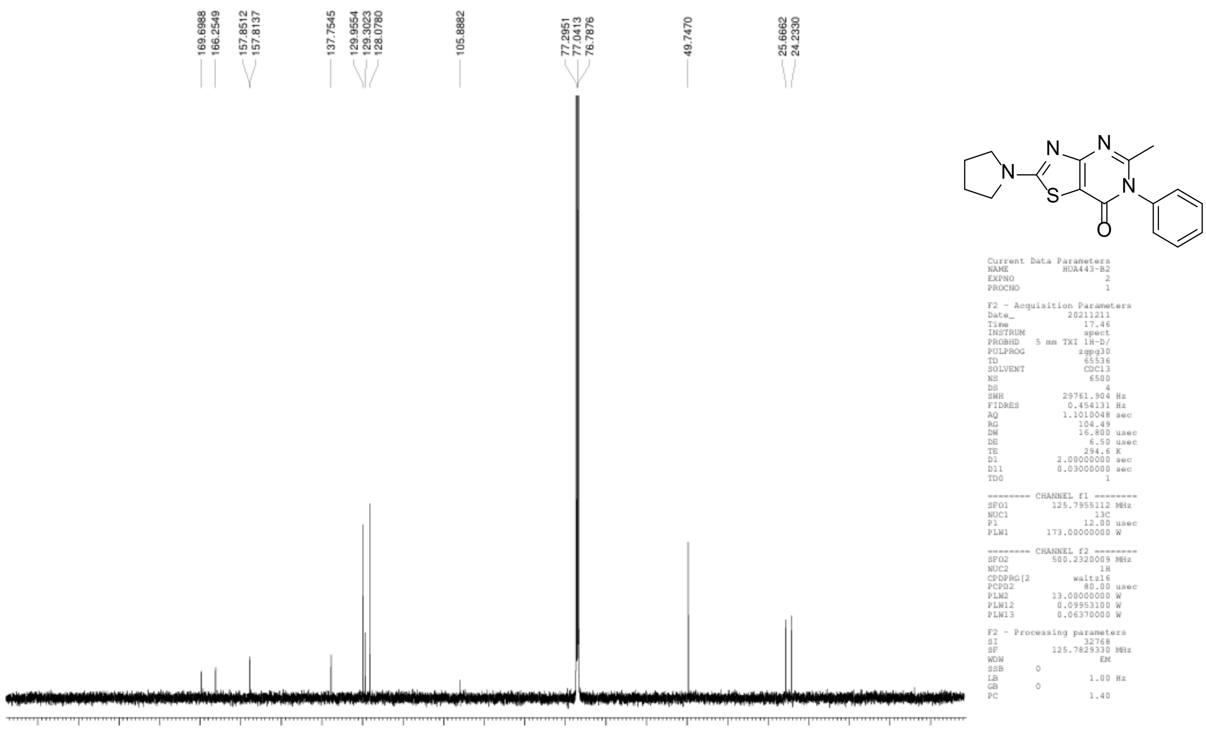

169.6988
166.2549
157.8512
157.8137
137.7545
129.9554
129.3023
128.0780
105.8882
77.2951
77.0413
76.7876
49.7470
25.6662
24.2330
210 200 190 180 170 160 150 140 130 120 110 100 90 80 70 60 50 40 30 20 10 0 ppm
N
N
N
S
N
O
Current Data Parameters
NAME HUA443-B2
EXPNO 2
PROCNO 1
F2 - Acquisition Parameters
Date_ 20211211
Time 17.46
INSTRUM spect
PROBHD 5 mm TXI 1H-D/
PULPROG zgpg30
TD 65536
SOLVENT CDCl3
NS 6500
DS 4
SWH 29761.904 Hz
FIDRES 0.454131 Hz
AQ 1.1010048 sec
RG 104.49
DW 16.800 usec
DE 6.50 usec
TE 294.6 K
D1 2.00000000 sec
D11 0.03000000 sec
TD0 1
======== CHANNEL f1 ========
SFO1 125.7955112 MHz
NUC1 13C
P1 12.00 usec
PLW1 173.0000000 W
======== CHANNEL f2 ========
SFO2 500.2320009 MHz
NUC2 1H
CPDPRG[2 waltz16
PCPD2 80.00 usec
PLW2 13.00000000 W
PLW12 0.09953100 W
PLW13 0.06370000 W
F2 - Processing parameters
SI 32768
SF 125.7829330 MHz
WDW EM
SSB 0
LB 1.00 Hz
GB 0
PC 1.40

1abe

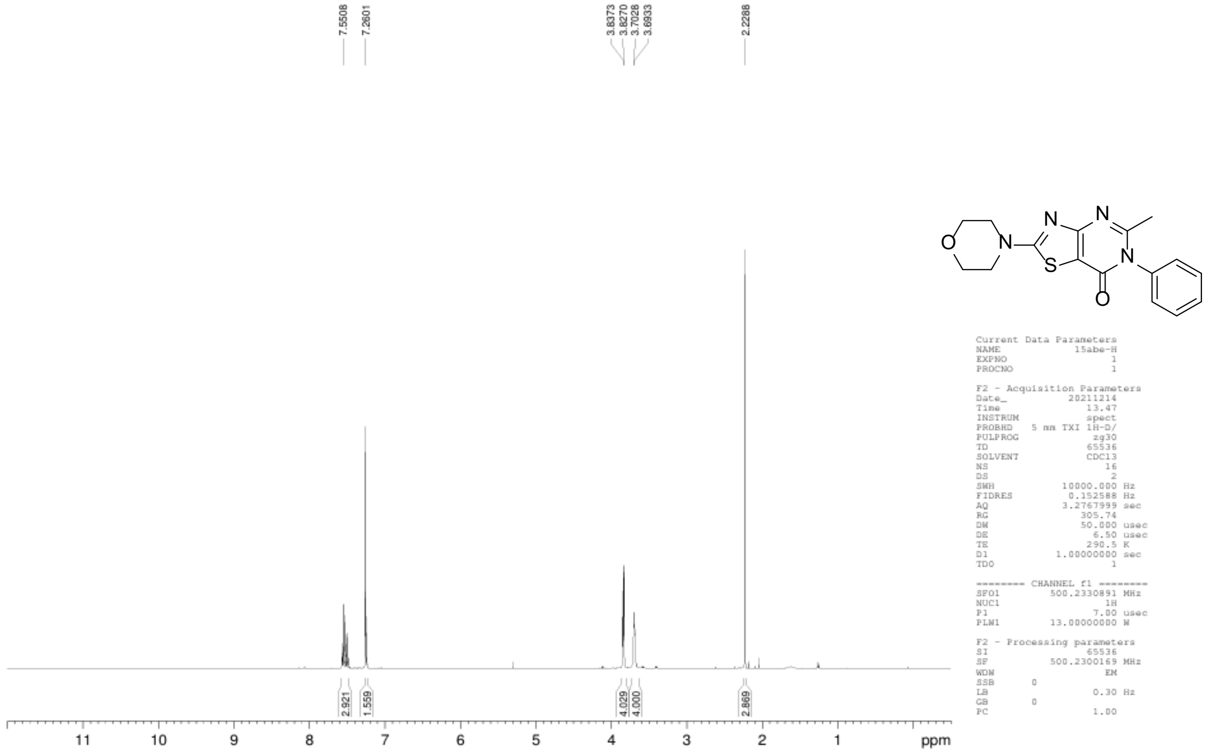

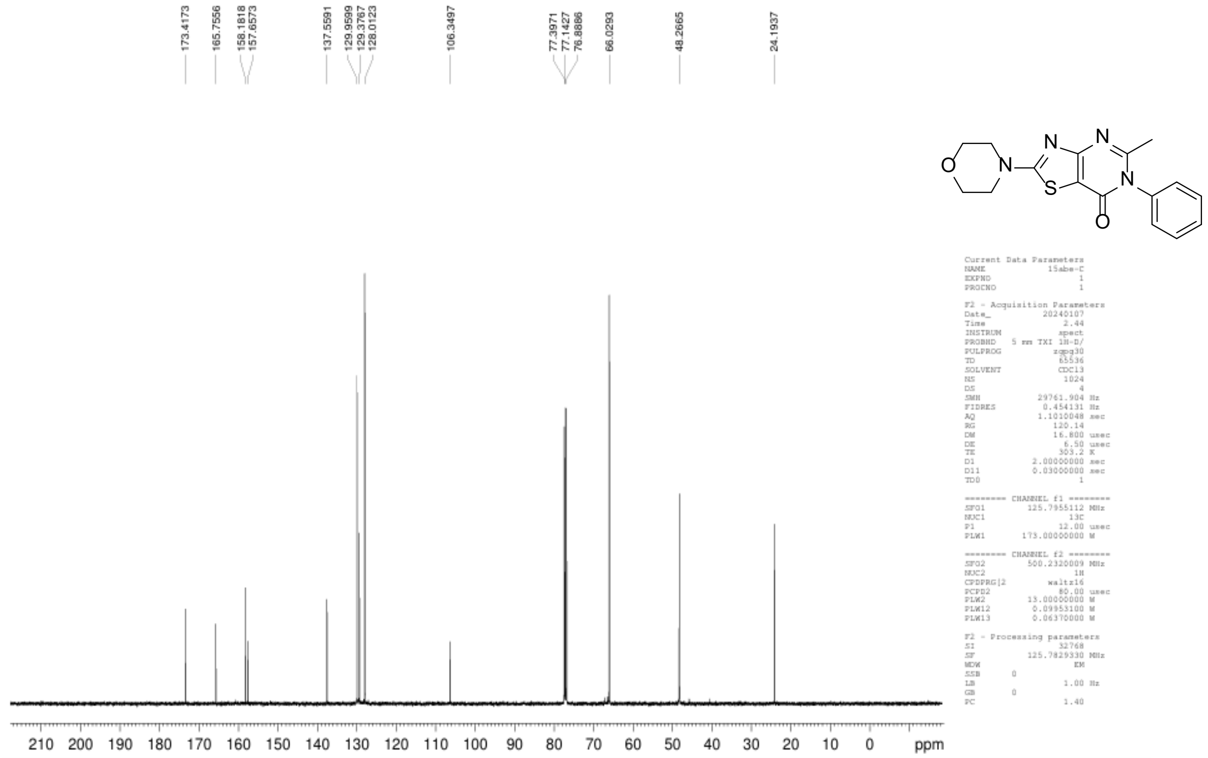

1abf

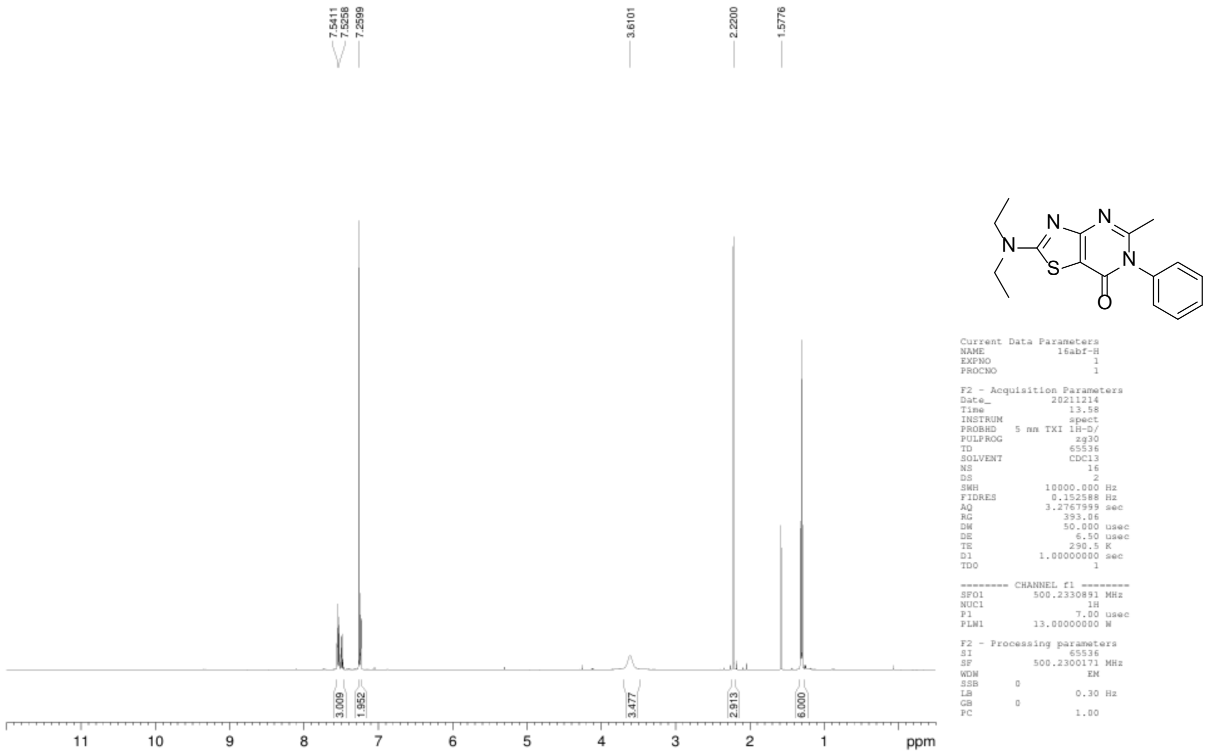
7.5411
7.5258
7.2599
3.6101
2.2200
1.5776
3.009
1.952
3.477
2.913
6.000
11 10 9 8 7 6 5 4 3 2 1 ppm
N
N
N
S
N
O
Current Data Parameters
NAME 16abf-H
EXPNO 1
PROCNO 1
F2 - Acquisition Parameters
Date_ 20211214
Time 13.58
INSTRUM spect
PROBHD 5 mm TXI 1H-D/
PULPROG zg30
TD 65536
SOLVENT CDCl3
NS 16
DS 2
SWH 10000.000 Hz
FIDRES 0.152588 Hz
AQ 3.2767999 sec
RG 393.06
DW 50.000 usec
DE 6.50 usec
TE 290.5 K
D1 1.00000000 sec
TD0 1
======== CHANNEL f1 ========
SFO1 500.2330891 MHz
NUC1 1H
P1 7.00 usec
PLW1 13.00000000 W
F2 - Processing parameters
SI 65536
SF 500.2300171 MHz
WDW EM
SSB 0
LB 0.30 Hz
GB 0
PC 1.00

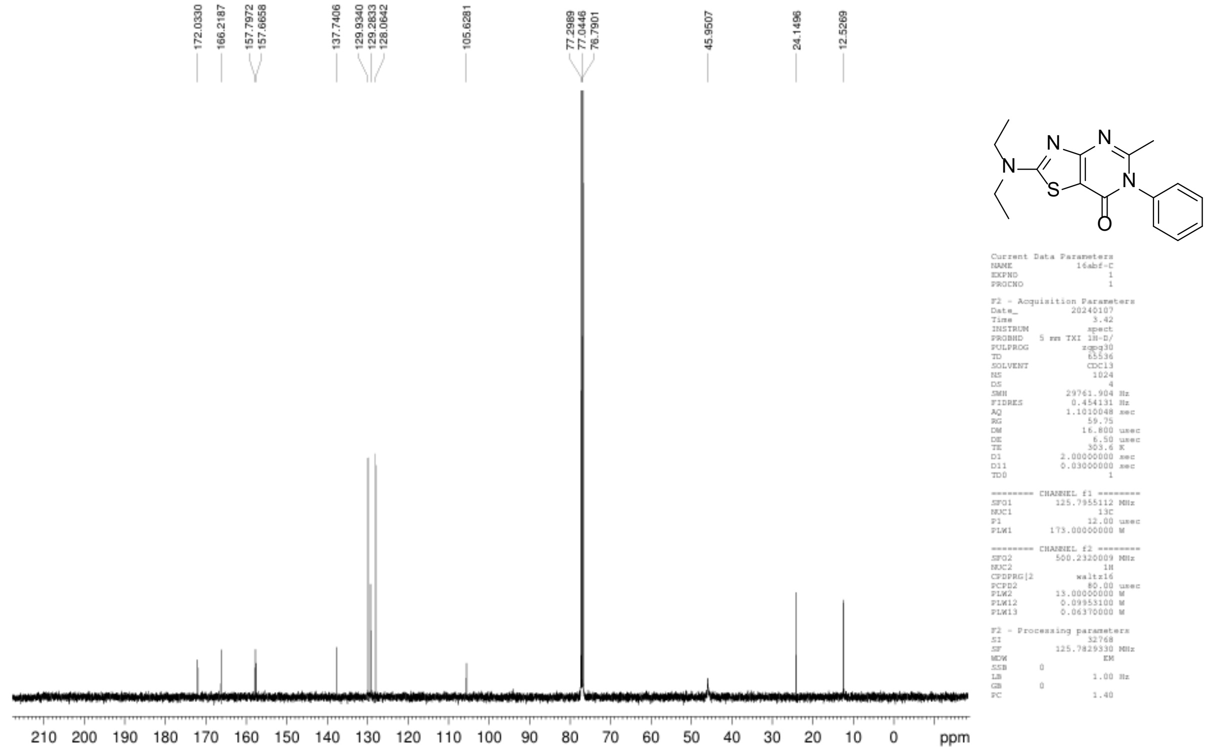
172.0330
166.2187
157.7972
157.6658
137.7406
129.9340
129.2833
128.0642
105.6281
77.2989
77.0446
76.7901
45.9507
24.1496
12.5269
210 200 190 180 170 160 150 140 130 120 110 100 90 80 70 60 50 40 30 20 10 0 ppm
N
N
N
S
N
O
Current Data Parameters
NAME 16abf-C
EXPNO 1
PROCNO 1
F2 - Acquisition Parameters
Date_ 20240107
Time 3.42
INSTRUM spect
PROBHD 5 mm TXI 1H-D/
PULPROG zgpg30
TD 65536
SOLVENT CDCl3
NS 1024
DS 4
SWH 29761.904 Hz
FIDRES 0.454131 Hz
AQ 1.1010048 sec
RG 59.75
DW 16.800 usec
DE 6.50 usec
TE 303.6 K
D1 2.00000000 sec
D11 0.03000000 sec
TD0 1
======== CHANNEL f1 ========
SFO1 125.7955112 MHz
NUC1 13C
P1 12.00 usec
PLW1 173.00000000 W
======== CHANNEL f2 ========
SFO2 500.2320009 MHz
NUC2 1H
CPDPRG[2 waltz16
PCPD2 80.00 usec
PLW2 13.00000000 W
PLW12 0.09953100 W
PLW13 0.06370000 W
F2 - Processing parameters
SI 32768
SF 125.7829330 MHz
WDW EM
SSB 0
LB 1.00 Hz
GB 0
PC 1.40

1abg

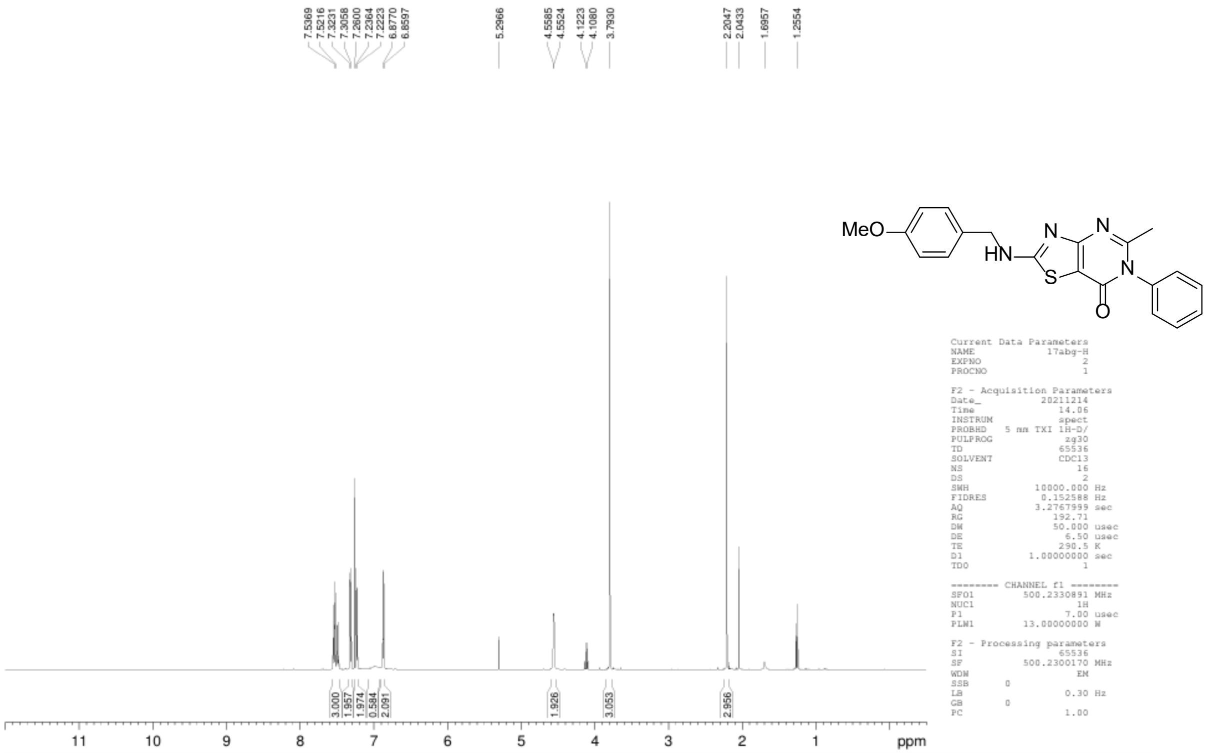
7.5369
7.5216
7.3231
7.3058
7.2600
7.2364
7.2223
6.8770
6.8597
5.2966
4.5585
4.5524
4.1223
4.1080
3.7930
2.2047
2.0433
1.6957
1.2554
MeO
HN
N
S
N
N
O
3.000
1.957
1.974
0.584
2.091
1.926
3.053
2.956
11
10
9
8
7
6
5
4
3
2
1
ppm

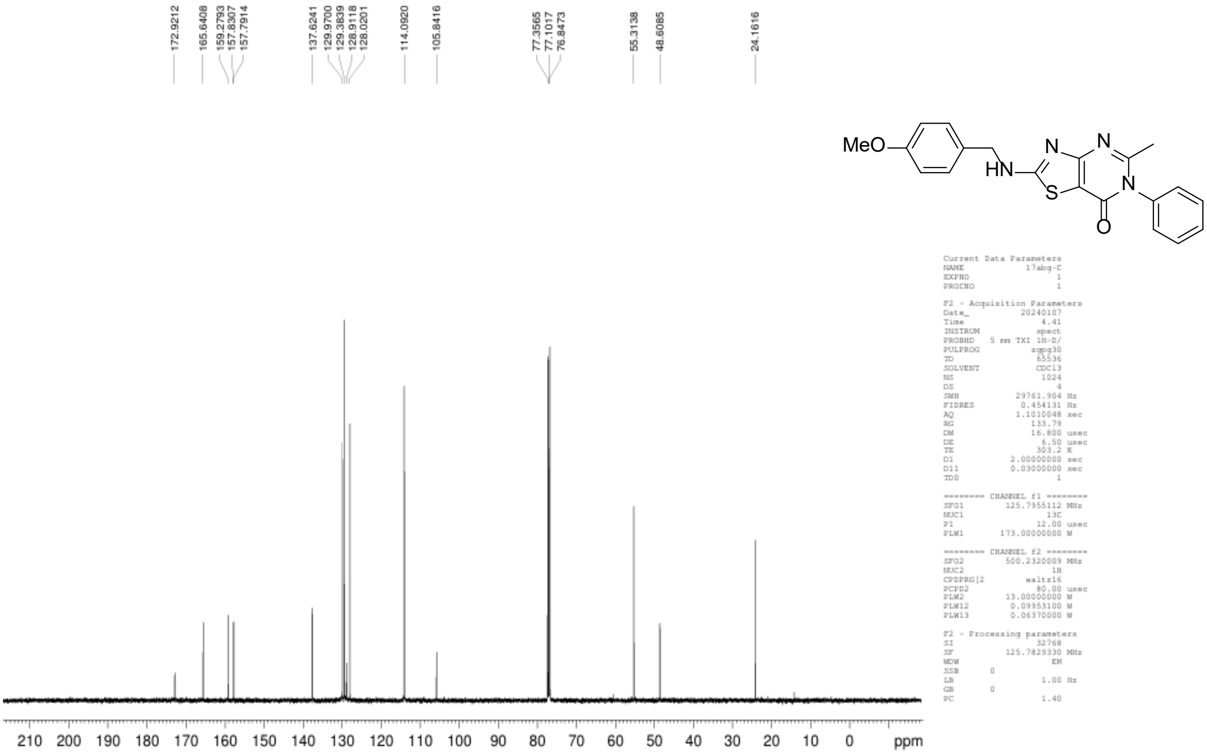
172.9212
165.6408
159.2793
157.8307
157.7914
137.6241
129.9700
129.3839
128.9118
128.0201
114.0920
105.8416
77.3565
77.1017
76.8473
55.3138
48.6085
24.1616
MeO
HN
N
S
N
N
O
210
200
190
180
170
160
150
140
130
120
110
100
90
80
70
60
50
40
30
20
10
0
ppm

1abh

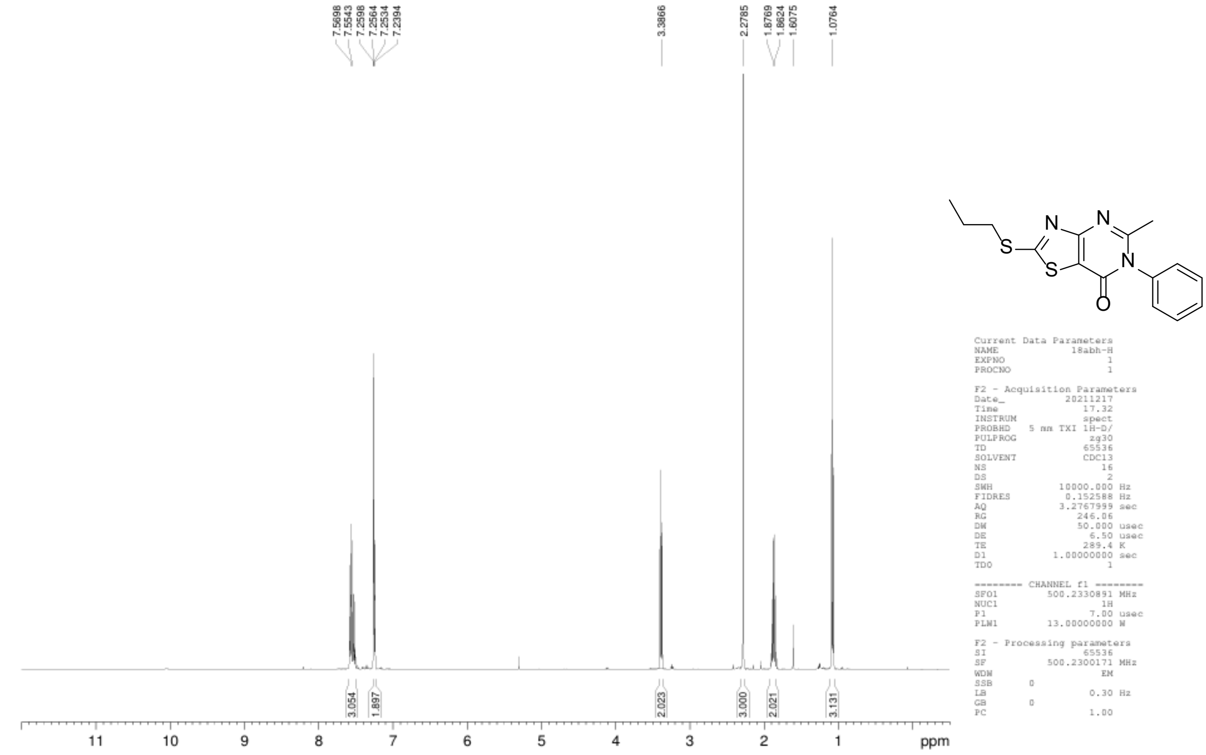

7.5698
7.5543
7.2598
7.2564
7.2534
7.2394
3.3866
2.2785
1.8769
1.8624
1.6075
1.0764
N
N
S
S
N
O
3.054
1.897
2.023
3.000
2.021
3.131
11
10
9
8
7
6
5
4
3
2
1
ppm
Current Data Parameters
NAME 18abh-H
EXPNO 1
PROCNO 1
F2 - Acquisition Parameters
Date_ 20211217
Time 17.32
INSTRUM spect
PROBHD 5 mm TXI 1H-D/
PULPROG zg30
TD 65536
SOLVENT CDCl3
NS 16
DS 2
SWH 10000.000 Hz
FIDRES 0.152588 Hz
AQ 3.2767999 sec
RG 246.06
DW 50.000 usec
DE 6.50 usec
TE 289.4 K
D1 1.00000000 sec
TD0 1
======== CHANNEL f1 ========
SFO1 500.2330891 MHz
NUC1 1H
P1 7.00 usec
PLW1 13.00000000 W
F2 - Processing parameters
SI 65536
SF 500.2300171 MHz
WDW EM
SSB 0
LB 0.30 Hz
GB 0
PC 1.00

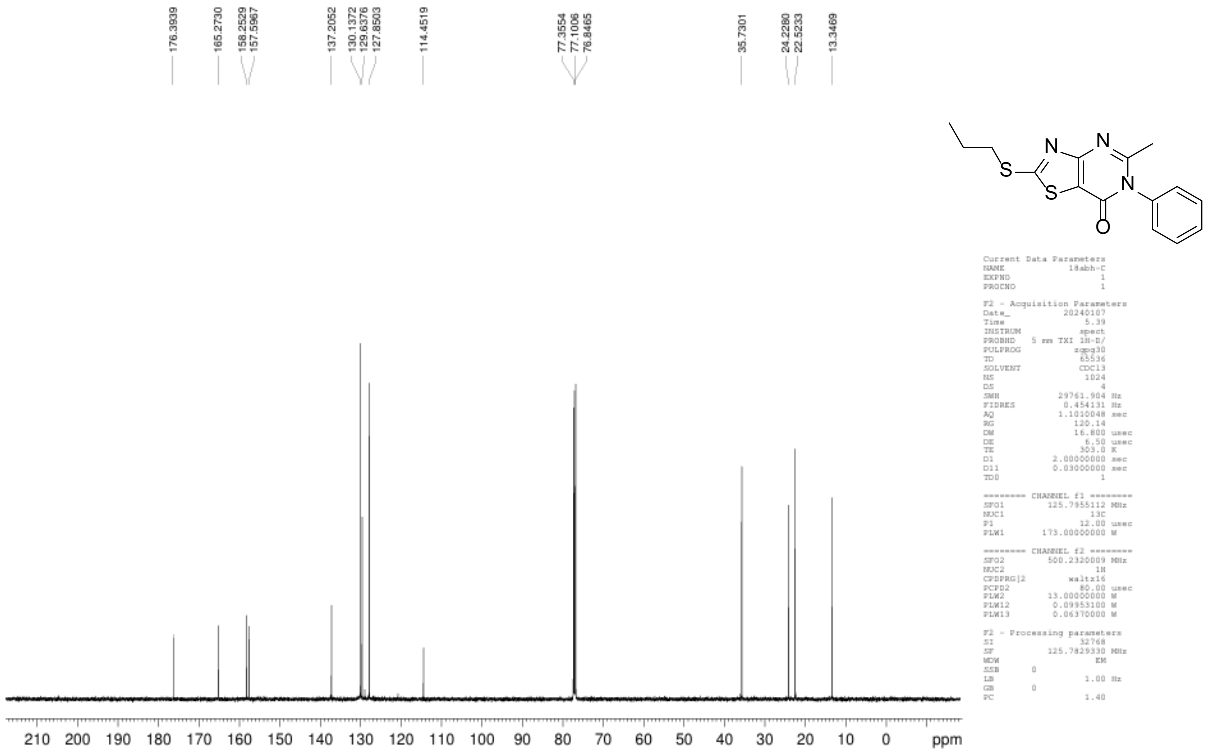

176.3939
165.2730
158.2529
157.5967
137.2052
130.1372
129.6376
127.8503
114.4519
77.3554
77.1006
76.8465
35.7301
24.2280
22.5233
13.3469
N
N
S
S
N
O
210
200
190
180
170
160
150
140
130
120
110
100
90
80
70
60
50
40
30
20
10
0
ppm
Current Data Parameters
NAME 18abh-C
EXPNO 1
PROCNO 1
F2 - Acquisition Parameters
Date_ 20240107
Time 5.39
INSTRUM spect
PROBHD 5 mm TXI 1H-D/
PULPROG zgpg30
TD 65536
SOLVENT CDCl3
NS 1024
DS 4
SWH 29761.904 Hz
FIDRES 0.454131 Hz
AQ 1.1010048 sec
RG 120.14
DW 16.800 usec
DE 6.50 usec
TE 303.0 K
D1 2.00000000 sec
D11 0.03000000 sec
TD0 1
======== CHANNEL f1 ========
SFO1 125.7955112 MHz
NUC1 13C
P1 12.00 usec
PLW1 173.00000000 W
======== CHANNEL f2 ========
SFO2 500.2320009 MHz
NUC2 1H
CPDPRG[2 waltz16
PCPD2 80.00 usec
PLW2 13.00000000 W
PLW12 0.09953100 W
PLW13 0.06370000 W
F2 - Processing parameters
SI 32768
SF 125.7829330 MHz
WDW EM
SSB 0
LB 1.00 Hz
GB 0
PC 1.40

1abi

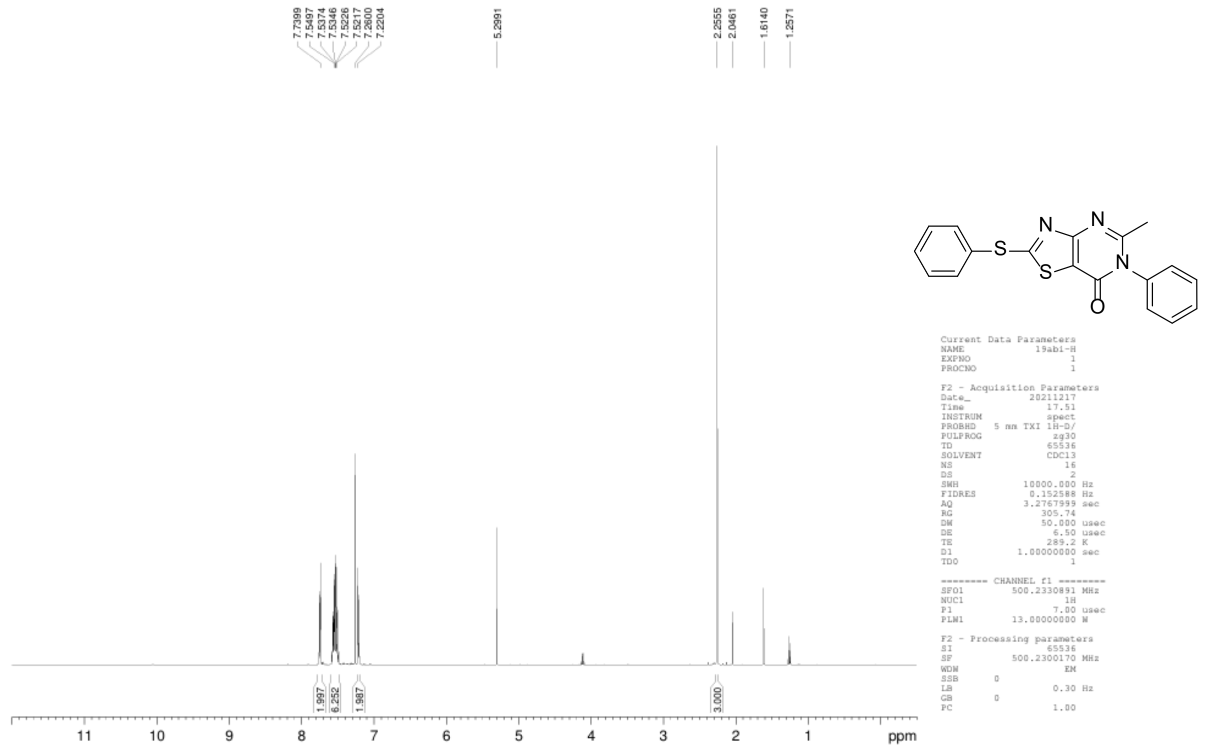
7.7399
7.5497
7.5374
7.5346
7.5226
7.5217
7.2800
7.2204
5.2991
2.2555
2.0461
1.6140
1.2571
1.997
6.252
1.987
3.000
11 10 9 8 7 6 5 4 3 2 1 ppm
N
N
S
S
N
O
Current Data Parameters
NAME 19abi-H
EXPNO 1
PROCNO 1
F2 - Acquisition Parameters
Date_ 20211217
Time 17.51
INSTRUM spect
PROBHD 5 mm TXI 1H-D/
PULPROG zg30
TD 65536
SOLVENT CDCl3
NS 16
DS 2
SWH 10000.000 Hz
FIDRES 0.152588 Hz
AQ 3.2767999 sec
RG 305.74
DW 50.000 usec
DE 6.50 usec
TE 289.2 K
D1 1.00000000 sec
TD0 1
======== CHANNEL f1 ========
SFO1 500.2330891 MHz
NUC1 1H
P1 7.00 usec
PLW1 13.00000000 W
F2 - Processing parameters
SI 65536
SF 500.2300170 MHz
WDW EM
SSB 0
LB 0.30 Hz
GB 0
PC 1.00

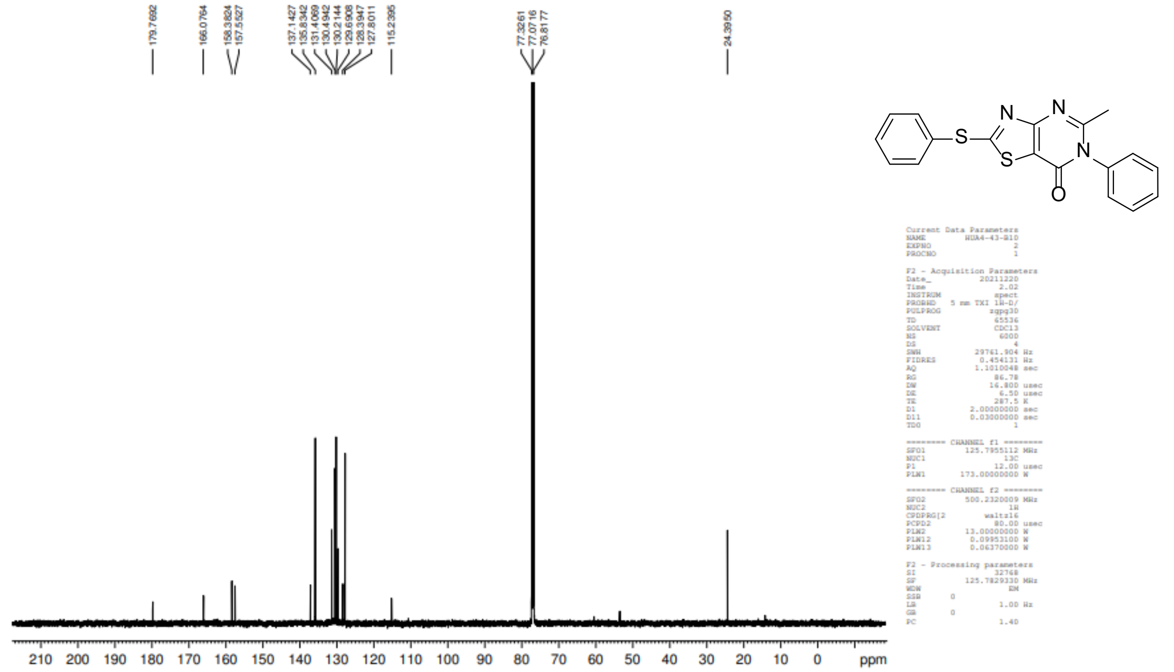
179.7692
166.0764
158.3824
157.5527
137.1427
135.6342
131.4069
130.4942
130.2144
129.6908
128.3947
127.8011
115.2395
77.3261
77.0716
76.8177
24.3950
210 200 190 180 170 160 150 140 130 120 110 100 90 80 70 60 50 40 30 20 10 0 ppm
N
N
S
S
N
O
Current Data Parameters
NAME HUA4-43-B10
EXPNO 2
PROCNO 1
F2 - Acquisition Parameters
Date_ 20211220
Time 2.02
INSTRUM spect
PROBHD 5 mm TXI 1H-D/
PULPROG zgpg30
TD 65536
SOLVENT CDCl3
NS 6000
DS 4
SWH 29761.904 Hz
FIDRES 0.454131 Hz
AQ 1.1010048 sec
RG 86.78
DW 16.800 usec
DE 6.50 usec
TE 287.5 K
D1 2.00000000 sec
D11 0.03000000 sec
TD0 1
======== CHANNEL f1 ========
SFO1 125.7955112 MHz
NUC1 13C
P1 12.00 usec
PLW1 173.00000000 W
======== CHANNEL f2 ========
SFO2 500.2320009 MHz
NUC2 1H
CPDPRG[2 waltz16
PCPD2 80.00 usec
PLW2 13.00000000 W
PLW12 0.09953100 W
PLW13 0.06370000 W
F2 - Processing parameters
SI 32768
SF 125.7829330 MHz
WDW EM
SSB 0
LB 1.00 Hz
GB 0
PC 1.40

1abj

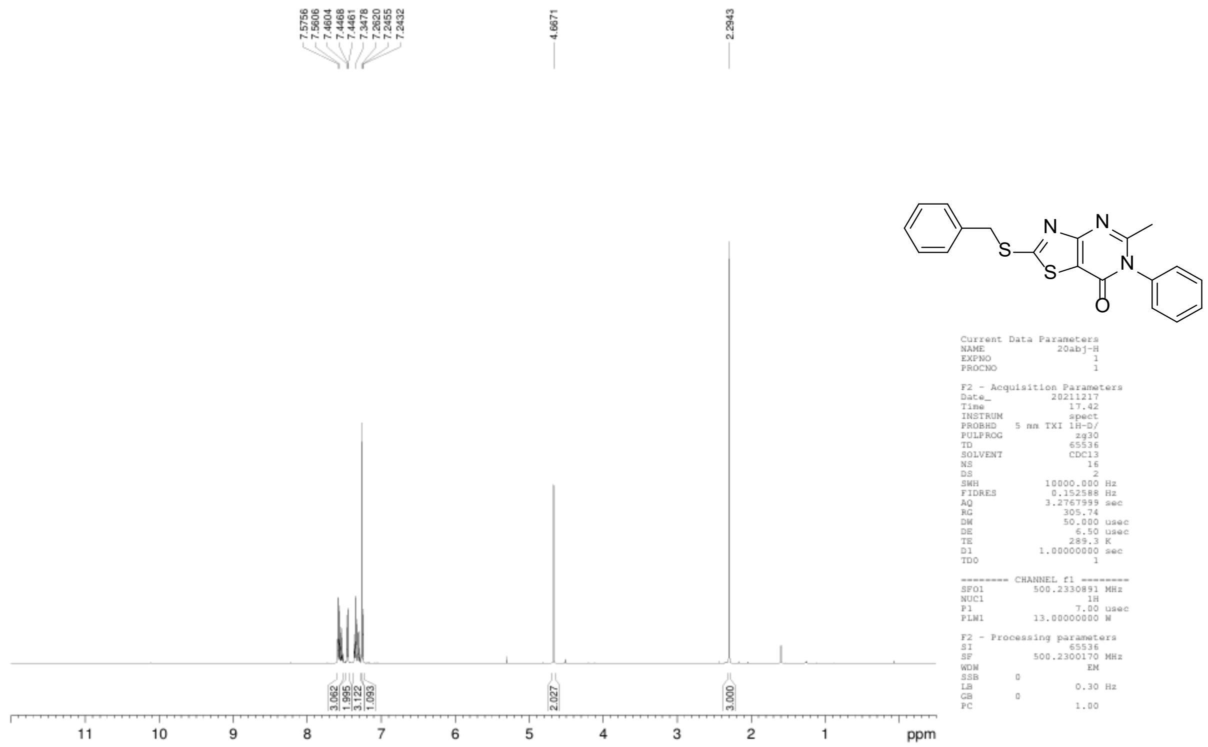
7.5756
7.5606
7.4604
7.4468
7.4461
7.3478
7.2620
7.2455
7.2432
4.6671
2.2943
3.062
1.995
3.122
1.093
2.027
3.000
11 10 9 8 7 6 5 4 3 2 1 ppm
N
N
S
S
N
O
Current Data Parameters
NAME 20abj-H
EXPNO 1
PROCNO 1
F2 - Acquisition Parameters
Date_ 20211217
Time 17.42
INSTRUM spect
PROBHD 5 mm TXI 1H-D/
PULPROG zg30
TD 65536
SOLVENT CDCl3
NS 16
DS 2
SWH 10000.000 Hz
FIDRES 0.152588 Hz
AQ 3.2767999 sec
RG 305.74
DW 50.000 usec
DE 6.50 usec
TE 289.3 K
D1 1.00000000 sec
TD0 1
======== CHANNEL f1 ========
SFO1 500.2330891 MHz
NUC1 1H
P1 7.00 usec
PLW1 13.00000000 W
F2 - Processing parameters
SI 65536
SF 500.2300170 MHz
WDW EM
SSB 0
LB 0.30 Hz
GB 0
PC 1.00

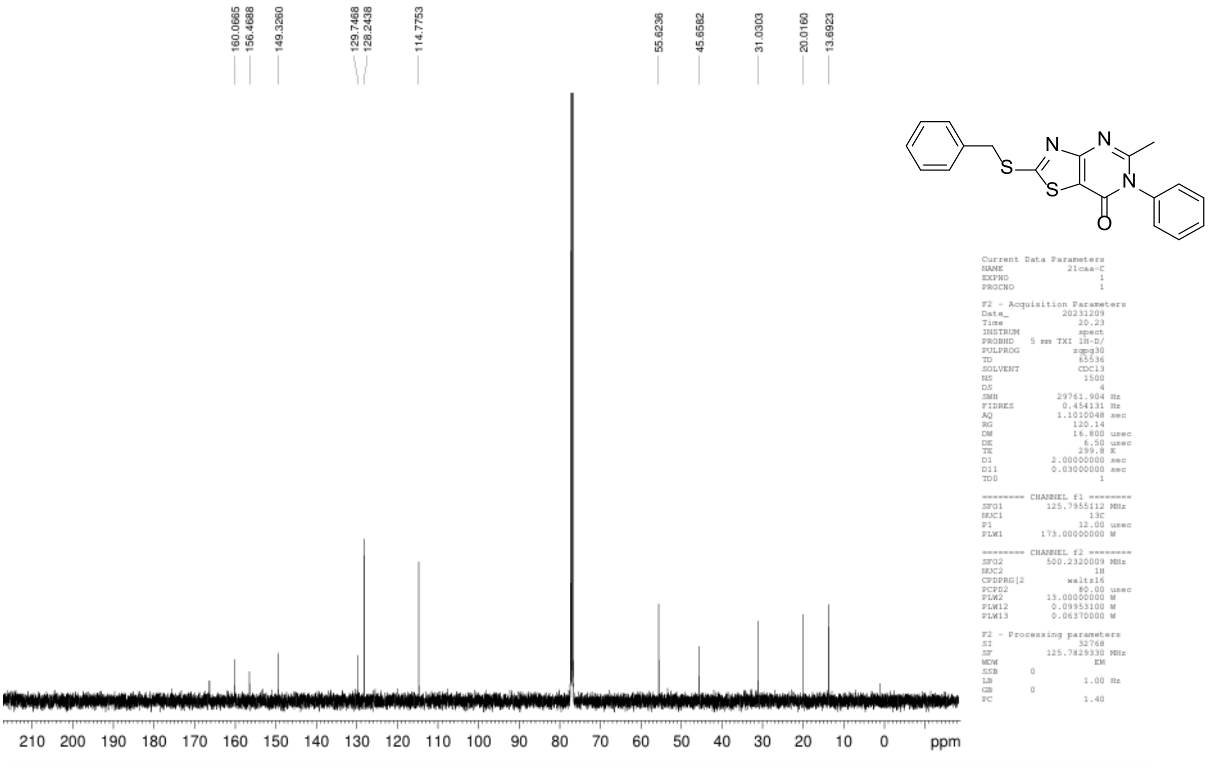
160.0665
156.4688
149.3260
129.7468
128.2438
114.7753
55.6236
45.6582
31.0303
20.0160
13.6923
210 200 190 180 170 160 150 140 130 120 110 100 90 80 70 60 50 40 30 20 10 0 ppm
N
N
S
S
N
O

1caa

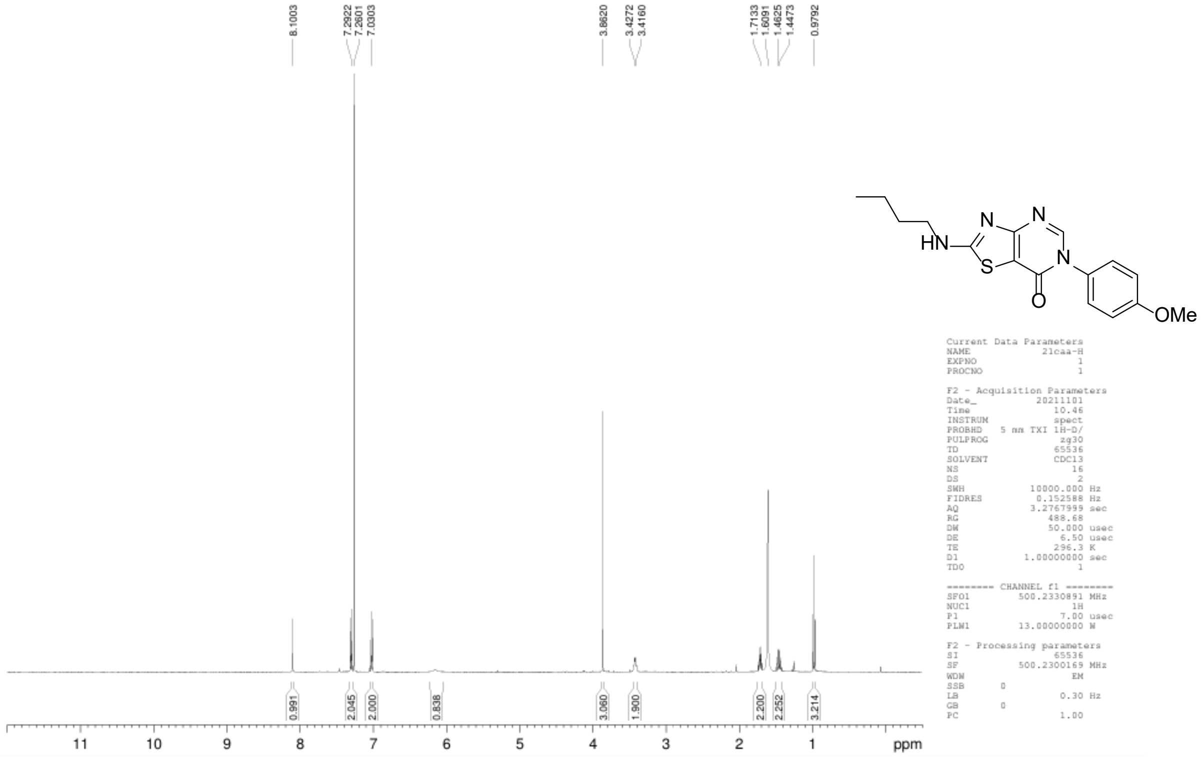
8.1003
7.2922
7.2601
7.0303
3.8620
3.4272
3.4160
1.7133
1.6091
1.4625
1.4473
0.9792
HN
N
N
S
N
O
OMe
0.991
2.045
2.000
0.838
3.060
1.900
2.200
2.252
3.214
11 10 9 8 7 6 5 4 3 2 1 ppm

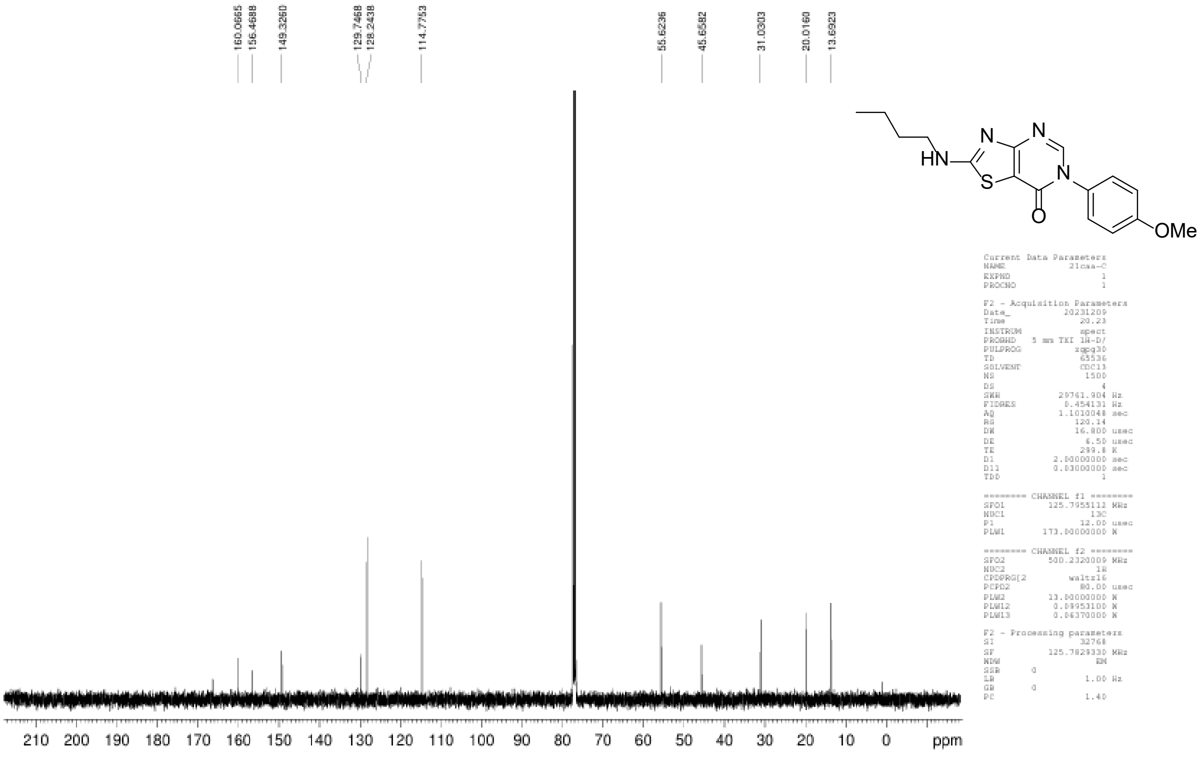
160.0665
156.4688
149.3260
129.7468
128.2438
114.7753
55.6236
45.5582
31.0303
20.0160
13.6923
HN
N
N
S
N
O
OMe
210 200 190 180 170 160 150 140 130 120 110 100 90 80 70 60 50 40 30 20 10 0 ppm

1cab

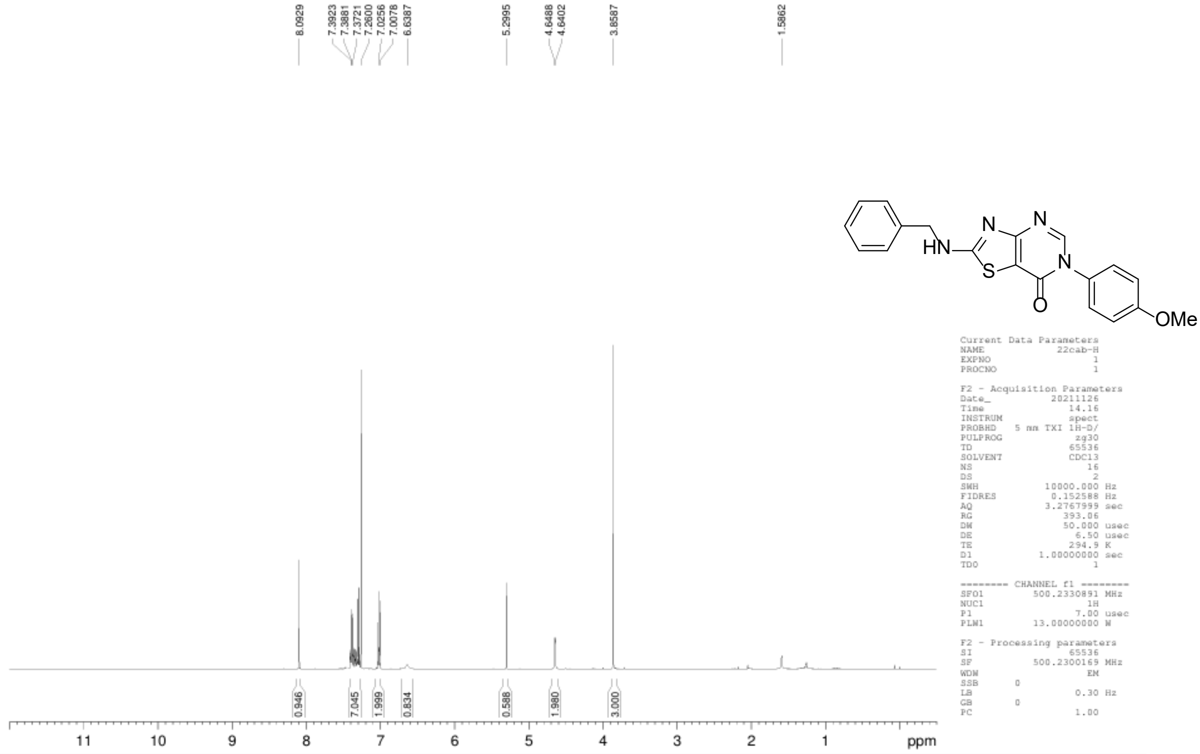
8.0929
7.3923
7.3881
7.3721
7.2600
7.0256
7.0078
6.6387
5.2995
4.6488
4.6402
3.8587
1.5862
HN
N
N
S
N
O
OMe
Current Data Parameters
NAME 22cab-H
EXPNO 1
PROCNO 1
F2 - Acquisition Parameters
Date_ 20211126
Time 14.16
INSTRUM spect
PROBHD 5 mm TXI 1H-D/
PULPROG zg30
TD 65536
SOLVENT CDCl3
NS 16
DS 2
SWH 10000.000 Hz
FIDRES 0.152588 Hz
AQ 3.2767999 sec
RG 393.06
DW 50.000 usec
DE 6.50 usec
TE 294.9 K
D1 1.00000000 sec
TD0 1
======== CHANNEL f1 ========
SFO1 500.2330891 MHz
NUC1 1H
P1 7.00 usec
PLW1 13.00000000 W
F2 - Processing parameters
SI 65536
SF 500.2300169 MHz
WDW EM
SSB 0
LB 0.30 Hz
GB 0
PC 1.00
0.946
7.045
1.999
0.834
0.588
1.980
3.000
11
10
9
8
7
6
5
4
3
2
1
ppm

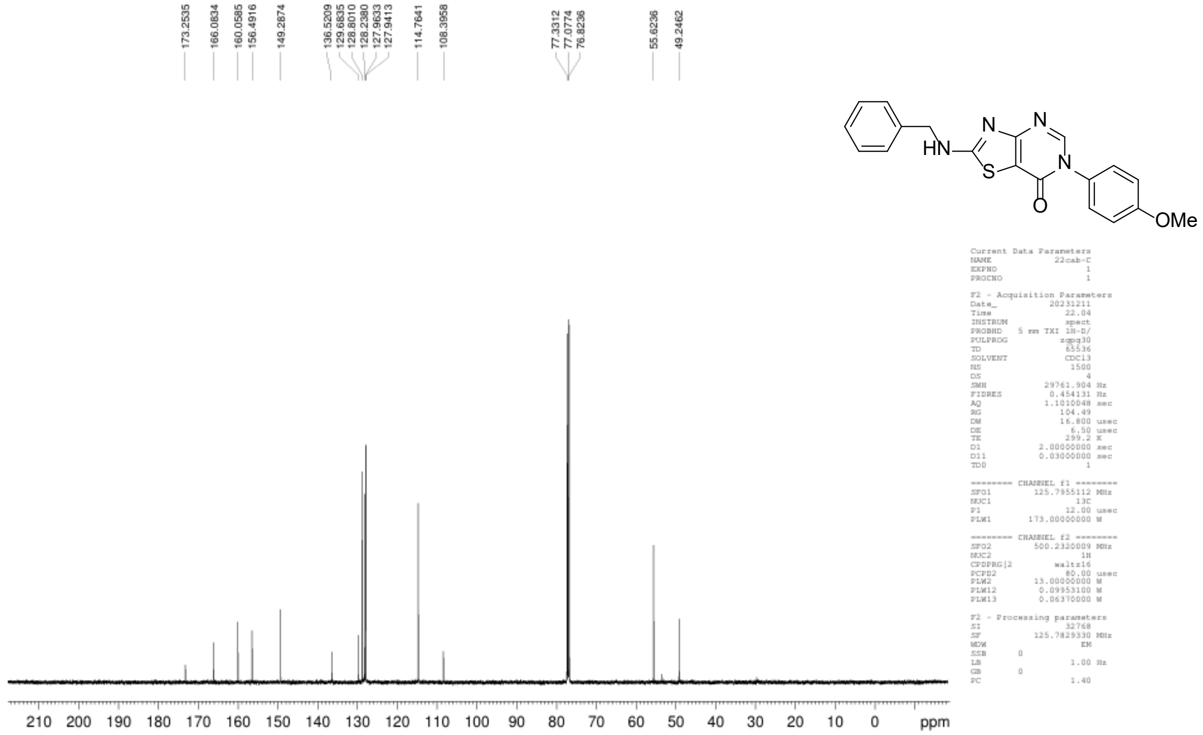
173.2535
166.0834
160.0585
156.4916
149.2874
136.5209
129.6835
128.8010
128.2380
127.9633
127.9413
114.7641
108.3958
77.3312
77.0774
76.8236
55.6236
49.2462
HN
N
N
S
N
O
OMe
Current Data Parameters
NAME 22cab-C
EXPNO 1
PROCNO 1
F2 - Acquisition Parameters
Date_ 20231211
Time 22.04
INSTRUM spect
PROBHD 5 mm TXI 1H-D/
PULPROG zgpg30
TD 65536
SOLVENT CDCl3
NS 1500
DS 4
SWH 29761.904 Hz
FIDRES 0.454131 Hz
AQ 1.1010048 sec
RG 104.49
DW 16.800 usec
DE 6.50 usec
TE 299.2 K
D1 2.00000000 sec
D11 0.03000000 sec
TD0 1
======== CHANNEL f1 ========
SFO1 125.7955112 MHz
NUC1 13C
P1 12.00 usec
PLW1 173.00000000 W
======== CHANNEL f2 ========
SFO2 500.2320009 MHz
NUC2 1H
CPDPRG[2 waltz16
PCPD2 80.00 usec
PLW2 13.00000000 W
PLW12 0.09953100 W
PLW13 0.06370000 W
F2 - Processing parameters
SI 32768
SF 125.7829330 MHz
WDW EM
SSB 0
LB 1.00 Hz
GB 0
PC 1.40
210
200
190
180
170
160
150
140
130
120
110
100
90
80
70
60
50
40
30
20
10
0
ppm

1cac

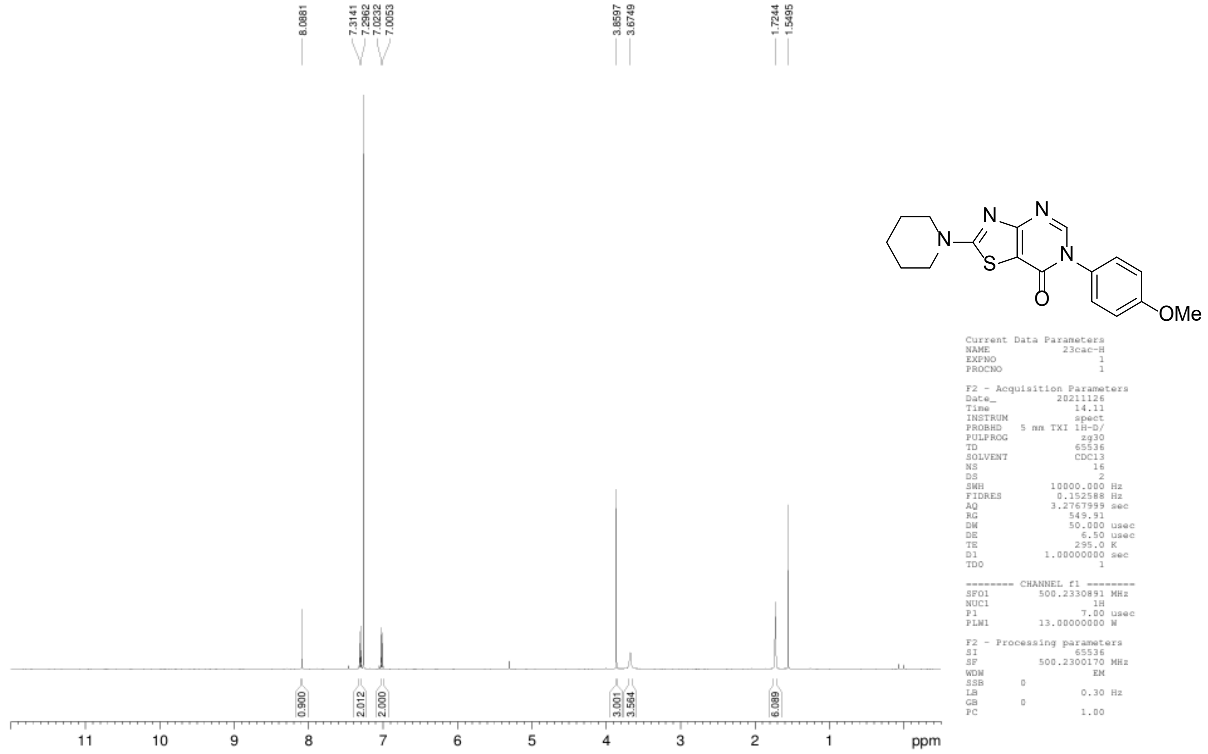
8.0881
7.3141
7.2962
7.0232
7.0053
3.8597
3.6749
1.7244
1.5495
0.900
2.012
2.000
3.001
3.564
6.089
ppm
N
S
O
OMe
Current Data Parameters
NAME 23cac-H
EXPNO 1
PROCNO 1
F2 - Acquisition Parameters
Date_ 20211126
Time 14.11
INSTRUM spect
PROBHD 5 mm TXI 1H-D/
PULPROG zg30
TD 65536
SOLVENT CDCl3
NS 16
DS 2
SWH 10000.000 Hz
FIDRES 0.152588 Hz
AQ 3.2767999 sec
RG 549.91
DW 50.000 usec
DE 6.50 usec
TE 295.0 K
D1 1.00000000 sec
TD0 1
======== CHANNEL f1 ========
SFO1 500.2330891 MHz
NUC1 1H
P1 7.00 usec
PLW1 13.00000000 W
F2 - Processing parameters
SI 65536
SF 500.2300170 MHz
WDW EM
SSB 0
LB 0.30 Hz
GB 0
PC 1.00

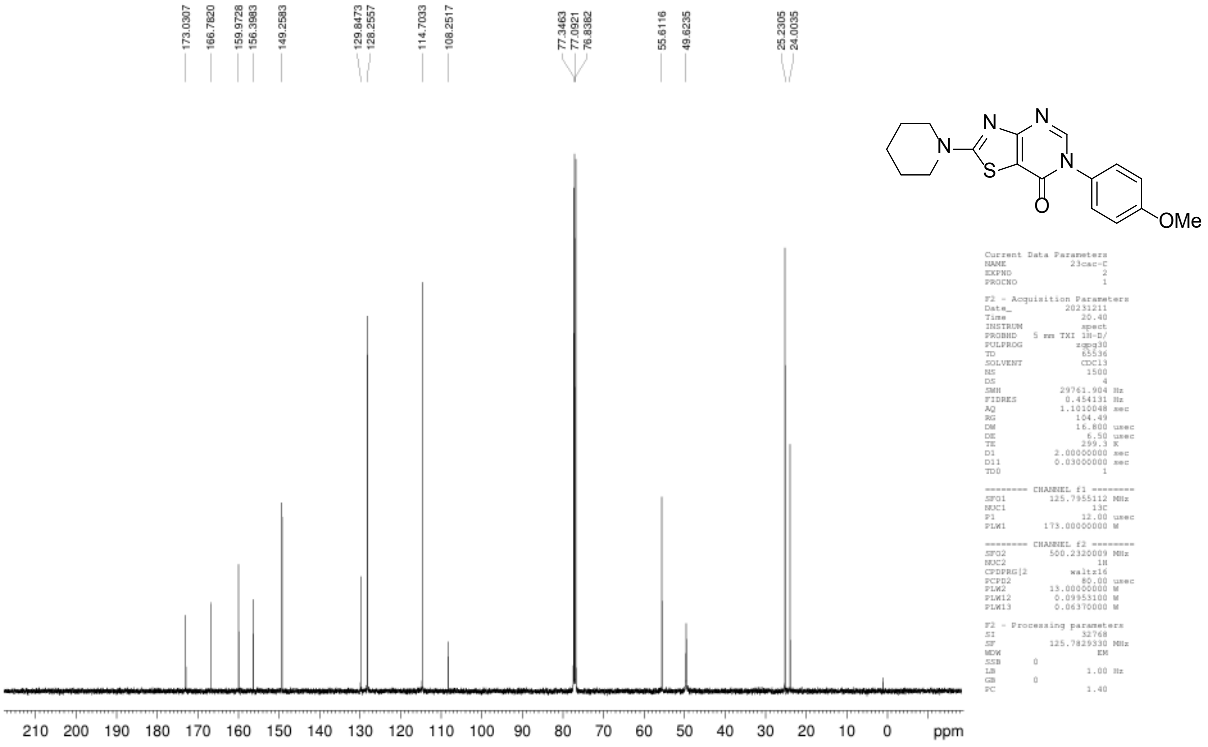
173.0307
166.7820
159.9728
156.3983
149.2583
129.8473
128.2557
114.7033
108.2517
77.3463
77.0921
76.8382
55.6116
49.6235
25.2305
24.0035
ppm
N
S
O
OMe
Current Data Parameters
NAME 23cac-C
EXPNO 2
PROCNO 1
F2 - Acquisition Parameters
Date_ 20231211
Time 20.40
INSTRUM spect
PROBHD 5 mm TXI 1H-D/
PULPROG zgpg30
TD 65536
SOLVENT CDCl3
NS 1500
DS 4
SWH 29761.904 Hz
FIDRES 0.454131 Hz
AQ 1.1010048 sec
RG 104.49
DW 16.800 usec
DE 6.50 usec
TE 299.3 K
D1 2.00000000 sec
D11 0.03000000 sec
TD0 1
======== CHANNEL f1 ========
SFO1 125.7955112 MHz
NUC1 13C
P1 12.00 usec
PLW1 173.00000000 W
======== CHANNEL f2 ========
SFO2 500.2320009 MHz
NUC2 1H
CPDPRG[2 waltz16
PCPD2 80.00 usec
PLW2 13.00000000 W
PLW12 0.09953100 W
PLW13 0.06370000 W
F2 - Processing parameters
SI 32768
SF 125.7829330 MHz
WDW EM
SSB 0
LB 1.00 Hz
GB 0
PC 1.40

1cad

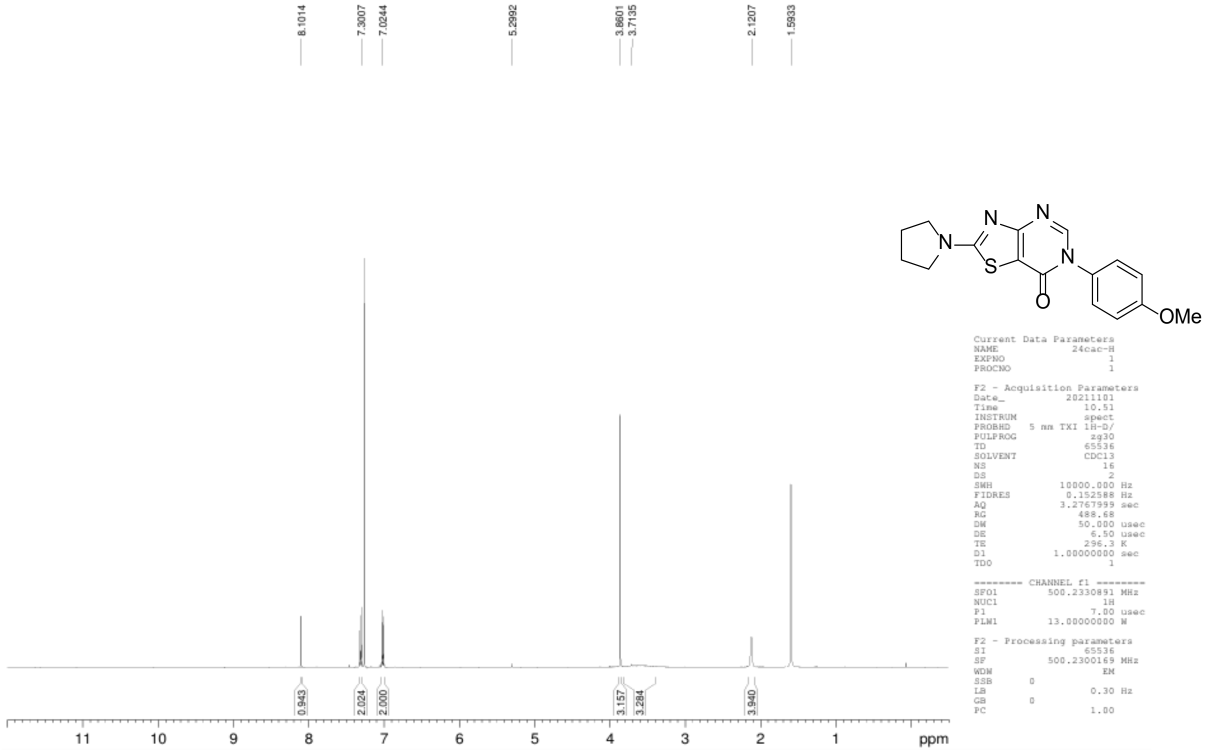
8.1014
7.3007
7.0244
5.2992
3.8601
3.7135
2.1207
1.5933
N
N
N
S
N
O
OMe
Current Data Parameters
NAME 24cac-H
EXPNO 1
PROCNO 1
F2 - Acquisition Parameters
Date_ 20211101
Time 10.51
INSTRUM spect
PROBHD 5 mm TXI 1H-D/
PULPROG zg30
TD 65536
SOLVENT CDCl3
NS 16
DS 2
SWH 10000.000 Hz
FIDRES 0.152588 Hz
AQ 3.2767999 sec
RG 488.68
DW 50.000 usec
DE 6.50 usec
TE 296.3 K
D1 1.00000000 sec
TD0 1
======== CHANNEL f1 ========
SFO1 500.2330891 MHz
NUC1 1H
P1 7.00 usec
PLW1 13.00000000 W
F2 - Processing parameters
SI 65536
SF 500.2300169 MHz
WDW EM
SSB 0
LB 0.30 Hz
GB 0
PC 1.00
0.943
2.024
2.000
3.157
3.284
3.940
11
10
9
8
7
6
5
4
3
2
1
ppm

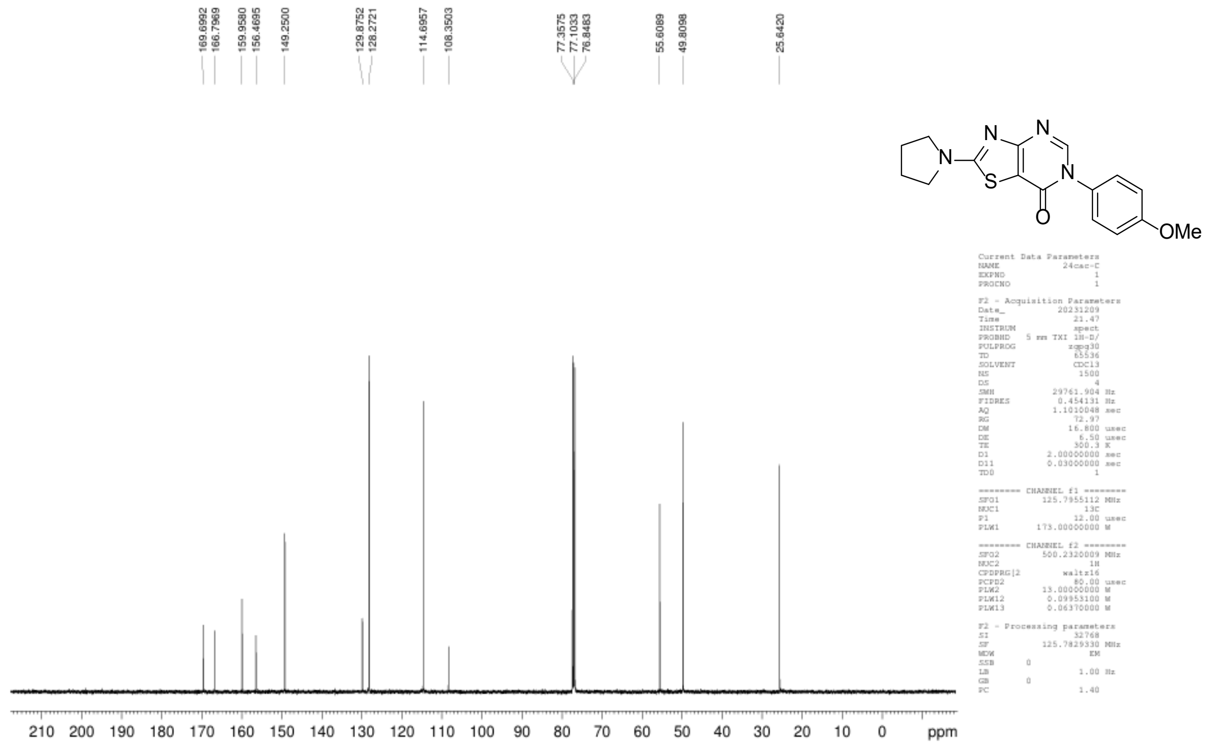
169.6992
166.7969
159.9580
156.4695
149.2500
129.8752
128.2721
114.6957
108.3503
77.3575
77.1033
76.8483
55.6089
49.8098
25.6420
N
N
N
S
N
O
OMe
Current Data Parameters
NAME 24cac-C
EXPNO 1
PROCNO 1
F2 - Acquisition Parameters
Date_ 20231209
Time 21.47
INSTRUM spect
PROBHD 5 mm TXI 1H-D/
PULPROG zgpg30
TD 65536
SOLVENT CDCl3
NS 1500
DS 4
SWH 29761.904 Hz
FIDRES 0.454131 Hz
AQ 1.1010048 sec
RG 72.97
DW 16.800 usec
DE 6.50 usec
TE 300.3 K
D1 2.00000000 sec
D11 0.03000000 sec
TD0 1
======== CHANNEL f1 ========
SFO1 125.7955112 MHz
NUC1 13C
P1 12.00 usec
PLW1 173.00000000 W
======== CHANNEL f2 ========
SFO2 500.2320009 MHz
NUC2 1H
CPDPRG[2 waltz16
PCPD2 80.00 usec
PLW2 13.00000000 W
PLW12 0.09953100 W
PLW13 0.06370000 W
F2 - Processing parameters
SI 32768
SF 125.7829330 MHz
WDW EM
SSB 0
LB 1.00 Hz
GB 0
PC 1.40
210
200
190
180
170
160
150
140
130
120
110
100
90
80
70
60
50
40
30
20
10
0
ppm

1cae

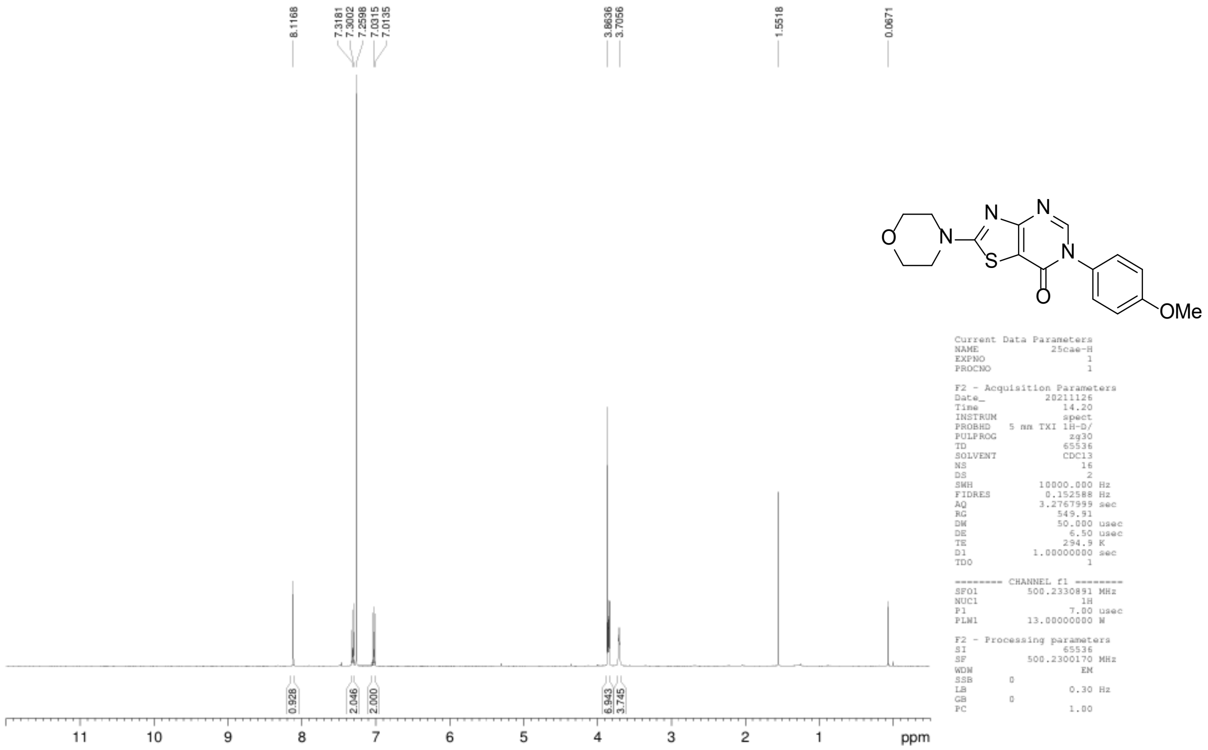
8.1168
7.3181
7.3002
7.2598
7.0315
7.0135
3.8636
3.7056
1.5518
0.0671
0.928
2.046
2.000
6.943
3.745
11
10
9
8
7
6
5
4
3
2
1
ppm
N
N
O
N
S
N
O
OMe
Current Data Parameters
NAME 25cae-H
EXPNO 1
PROCNO 1
F2 - Acquisition Parameters
Date_ 20211126
Time 14.20
INSTRUM spect
PROBHD 5 mm TXI 1H-D/
PULPROG zg30
TD 65536
SOLVENT CDCl3
NS 16
DS 2
SWH 10000.000 Hz
FIDRES 0.152588 Hz
AQ 3.2767999 sec
RG 549.91
DW 50.000 usec
DE 6.50 usec
TE 294.9 K
D1 1.00000000 sec
TD0 1
======== CHANNEL f1 ========
SFO1 500.2330891 MHz
NUC1 1H
P1 7.00 usec
PLW1 13.00000000 W
F2 - Processing parameters
SI 65536
SF 500.2300170 MHz
WDW EM
SSB 0
LB 0.30 Hz
GB 0
PC 1.00

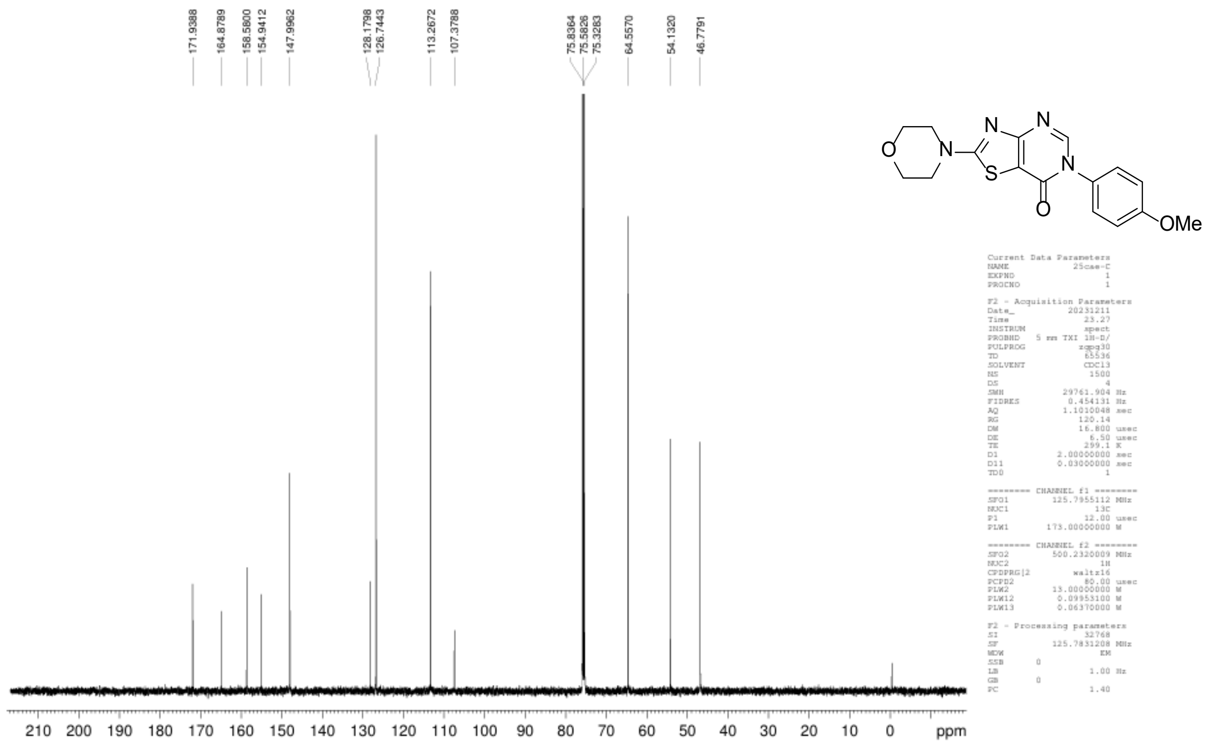
171.9388
164.8789
158.5800
154.9412
147.9962
128.1798
126.7443
113.2672
107.3788
75.8364
75.5826
75.3283
64.5570
54.1320
46.7791
210
200
190
180
170
160
150
140
130
120
110
100
90
80
70
60
50
40
30
20
10
0
ppm
N
N
O
N
S
N
O
OMe
Current Data Parameters
NAME 25cae-C
EXPNO 1
PROCNO 1
F2 - Acquisition Parameters
Date_ 20231211
Time 23.27
INSTRUM spect
PROBHD 5 mm TXI 1H-D/
PULPROG zgpg30
TD 65536
SOLVENT CDCl3
NS 1500
DS 4
SWH 29761.904 Hz
FIDRES 0.454131 Hz
AQ 1.1010048 sec
RG 120.14
DW 16.800 usec
DE 6.50 usec
TE 299.1 K
D1 2.00000000 sec
D11 0.03000000 sec
TD0 1
======== CHANNEL f1 ========
SFO1 125.7955112 MHz
NUC1 13C
P1 12.00 usec
PLW1 173.00000000 W
======== CHANNEL f2 ========
SFO2 500.2320009 MHz
NUC2 1H
CPDPRG[2 waltz16
PCPD2 80.00 usec
PLW2 13.00000000 W
PLW12 0.09953100 W
PLW13 0.06370000 W
F2 - Processing parameters
SI 32768
SF 125.7831208 MHz
WDW EM
SSB 0
LB 1.00 Hz
GB 0
PC 1.40

1cag

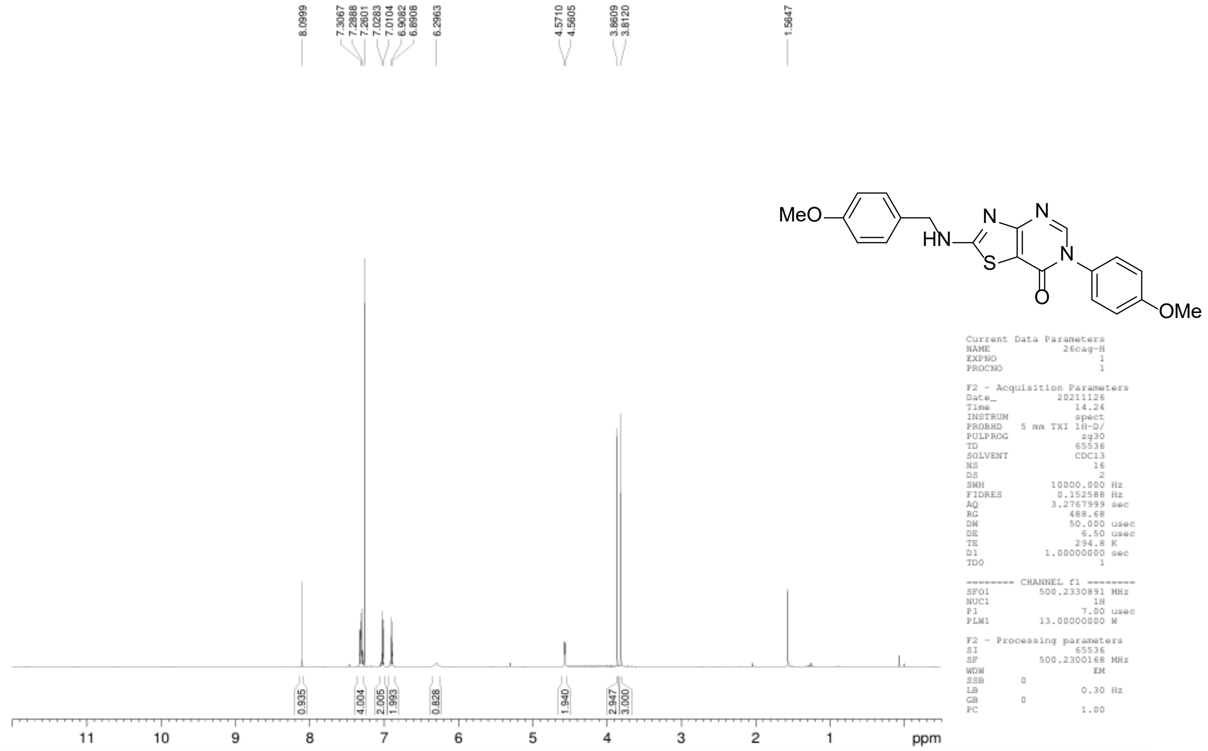
8.0999
7.3067
7.2888
7.2601
7.0283
7.0104
6.9082
6.8908
6.2963
4.5710
4.5605
3.8609
3.8120
1.5647
MeO
HN
N
S
N
N
O
OMe
0.935
4.004
2.005
1.993
0.828
1.940
2.947
3.000
ppm

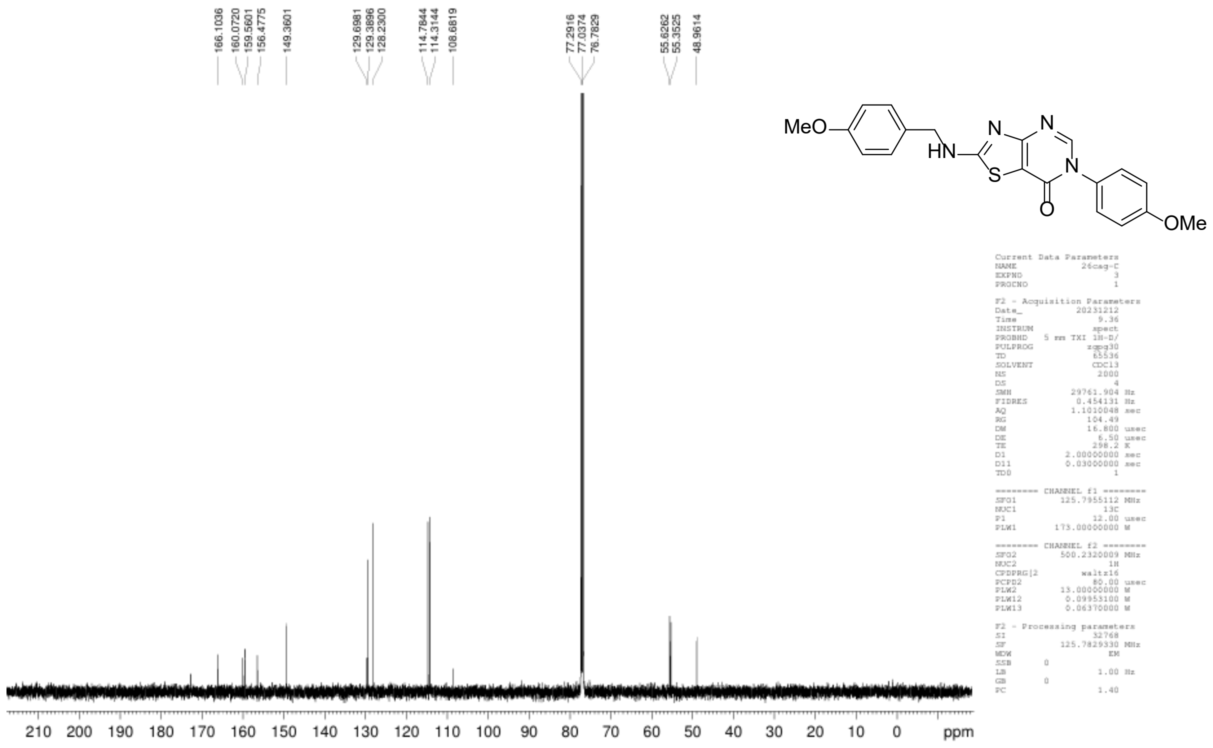
166.1036
160.0720
159.5601
156.4775
149.3601
129.6981
129.3896
128.2300
114.7844
114.3144
108.6819
77.2916
77.0374
76.7829
55.6262
55.3525
48.9614
MeO
HN
N
S
N
N
O
OMe
ppm

1cah

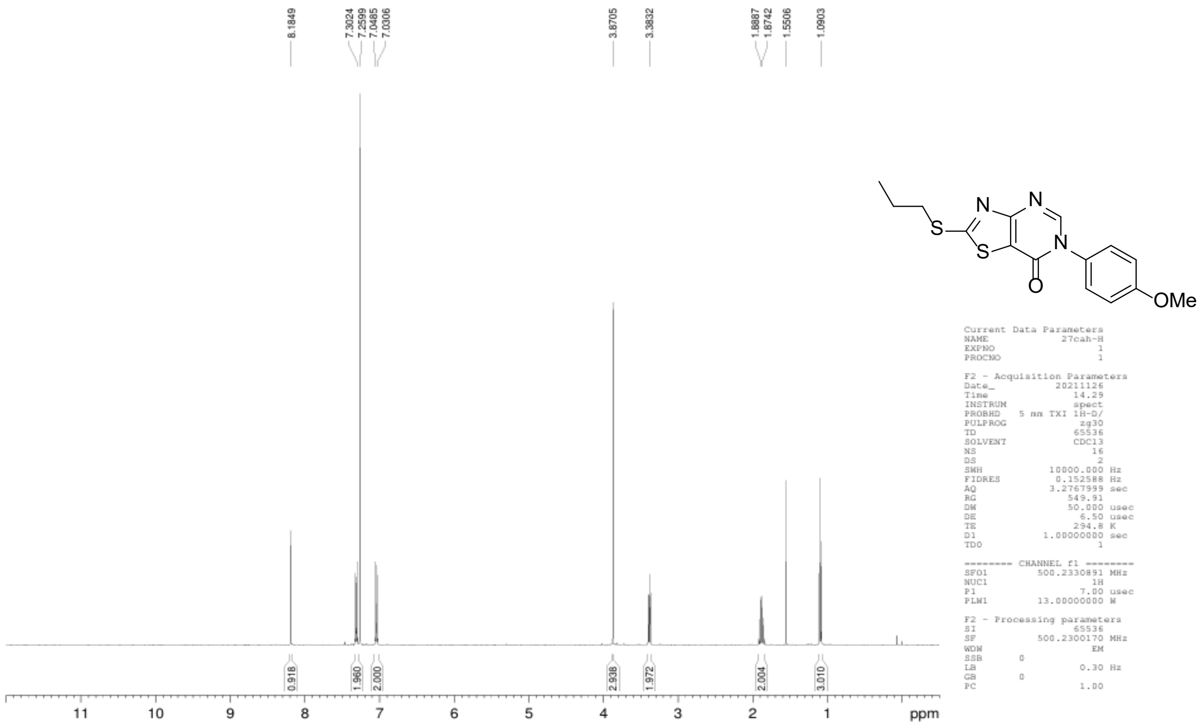
8.1849
7.3024
7.2599
7.0485
7.0306
3.8705
3.3832
1.8887
1.8742
1.5506
1.0903
0.918
1.960
2.000
2.938
1.972
2.004
3.010
11
10
9
8
7
6
5
4
3
2
1
ppm
N
N
S
S
N
O
OMe
Current Data Parameters
NAME 27cah-H
EXPNO 1
PROCNO 1
F2 - Acquisition Parameters
Date_ 20211126
Time 14.29
INSTRUM spect
PROBHD 5 mm TXI 1H-D/
PULPROG zg30
TD 65536
SOLVENT CDCl3
NS 16
DS 2
SWH 10000.000 Hz
FIDRES 0.152588 Hz
AQ 3.2767999 sec
RG 549.91
DW 50.000 usec
DE 6.50 usec
TE 294.8 K
D1 1.00000000 sec
TD0 1
======== CHANNEL f1 ========
SFO1 500.2330891 MHz
NUC1 1H
P1 7.00 usec
PLW1 13.00000000 W
F2 - Processing parameters
SI 65536
SF 500.2300170 MHz
WDW EM
SSB 0
LB 0.30 Hz
GB 0
PC 1.00

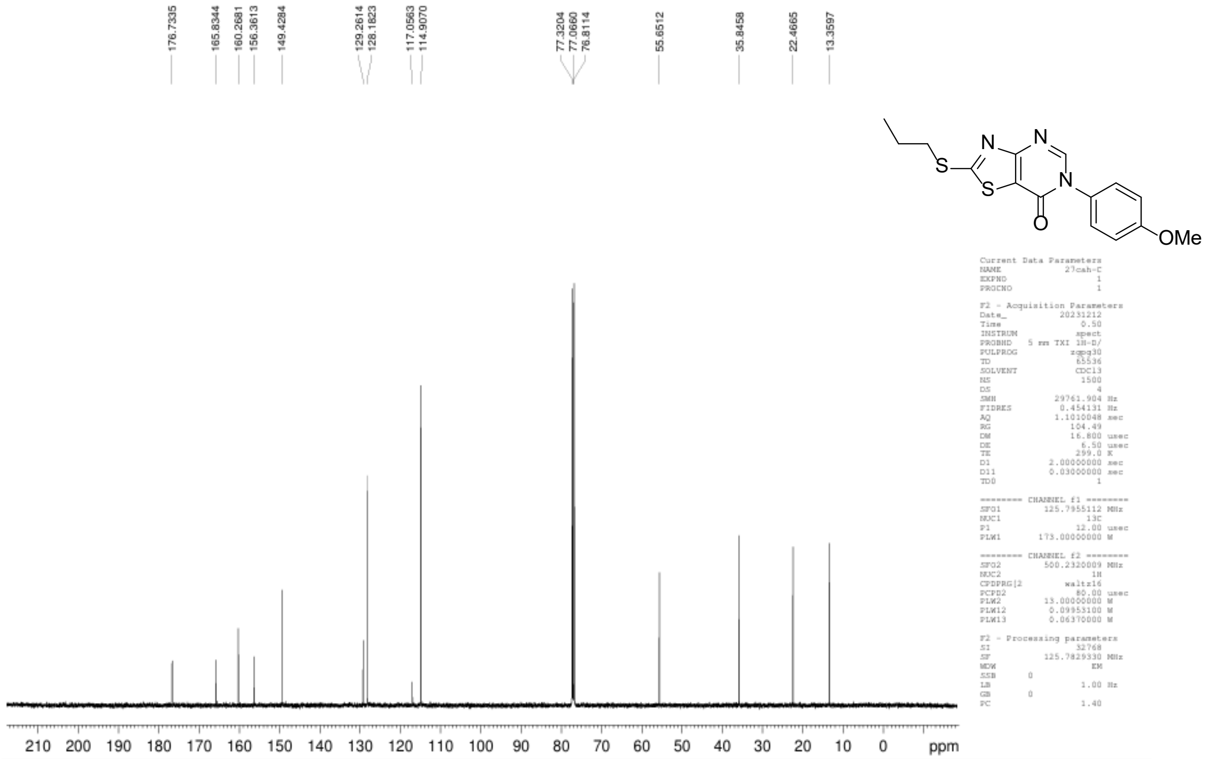
176.7335
165.8344
160.2681
156.3613
149.4284
129.2614
128.1823
117.0563
114.9070
77.3204
77.0660
76.8114
55.6512
35.8458
22.4665
13.3597
210
200
190
180
170
160
150
140
130
120
110
100
90
80
70
60
50
40
30
20
10
0
ppm
N
N
S
S
N
O
OMe
Current Data Parameters
NAME 27cah-C
EXPNO 1
PROCNO 1
F2 - Acquisition Parameters
Date_ 20231212
Time 0.50
INSTRUM spect
PROBHD 5 mm TXI 1H-D/
PULPROG zgpg30
TD 65536
SOLVENT CDCl3
NS 1500
DS 4
SWH 29761.904 Hz
FIDRES 0.454131 Hz
AQ 1.1010048 sec
RG 104.49
DW 16.800 usec
DE 6.50 usec
TE 299.0 K
D1 2.00000000 sec
D11 0.03000000 sec
TD0 1
======== CHANNEL f1 ========
SFO1 125.7955112 MHz
NUC1 13C
P1 12.00 usec
PLW1 173.00000000 W
======== CHANNEL f2 ========
SFO2 500.2320009 MHz
NUC2 1H
CPDPRG[2 waltz16
PCPD2 80.00 usec
PLW2 13.00000000 W
PLW12 0.09953100 W
PLW13 0.06370000 W
F2 - Processing parameters
SI 32768
SF 125.7829330 MHz
WDW EM
SSB 0
LB 1.00 Hz
GB 0
PC 1.40

1cai

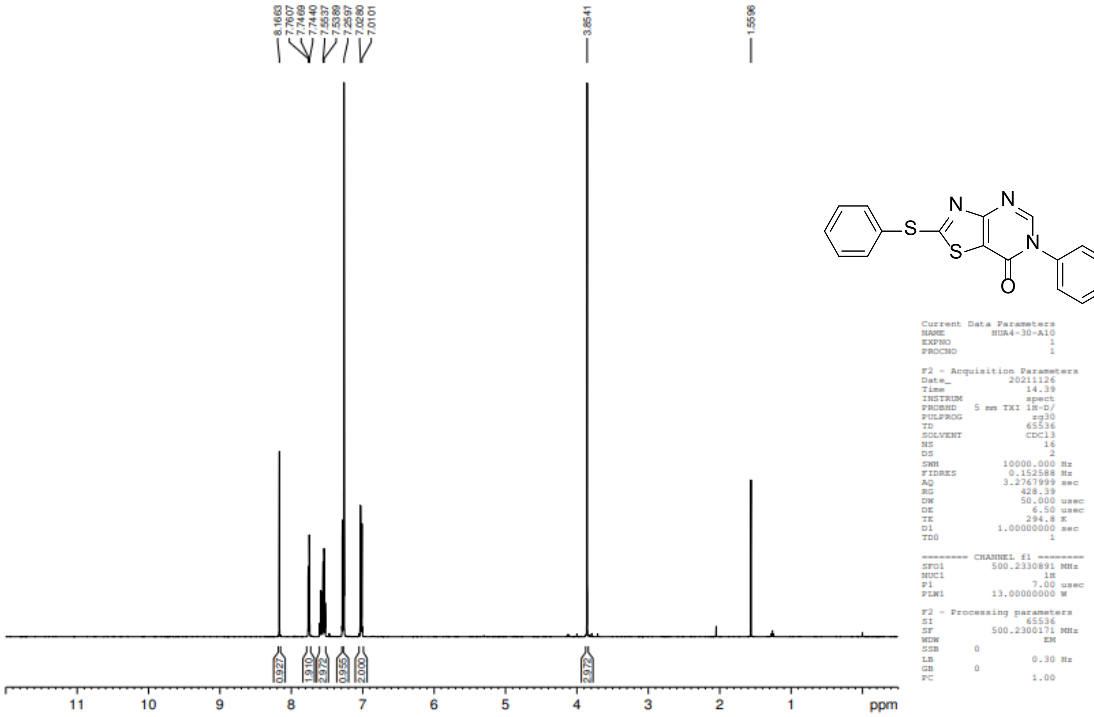

8.1663
7.7607
7.7469
7.7440
7.5537
7.5389
7.2597
7.0280
7.0101
3.8541
1.5596
N
N
S
S
N
O
OMe
0.927
1.910
2.972
0.955
2.000
2.972
11 10 9 8 7 6 5 4 3 2 1 ppm

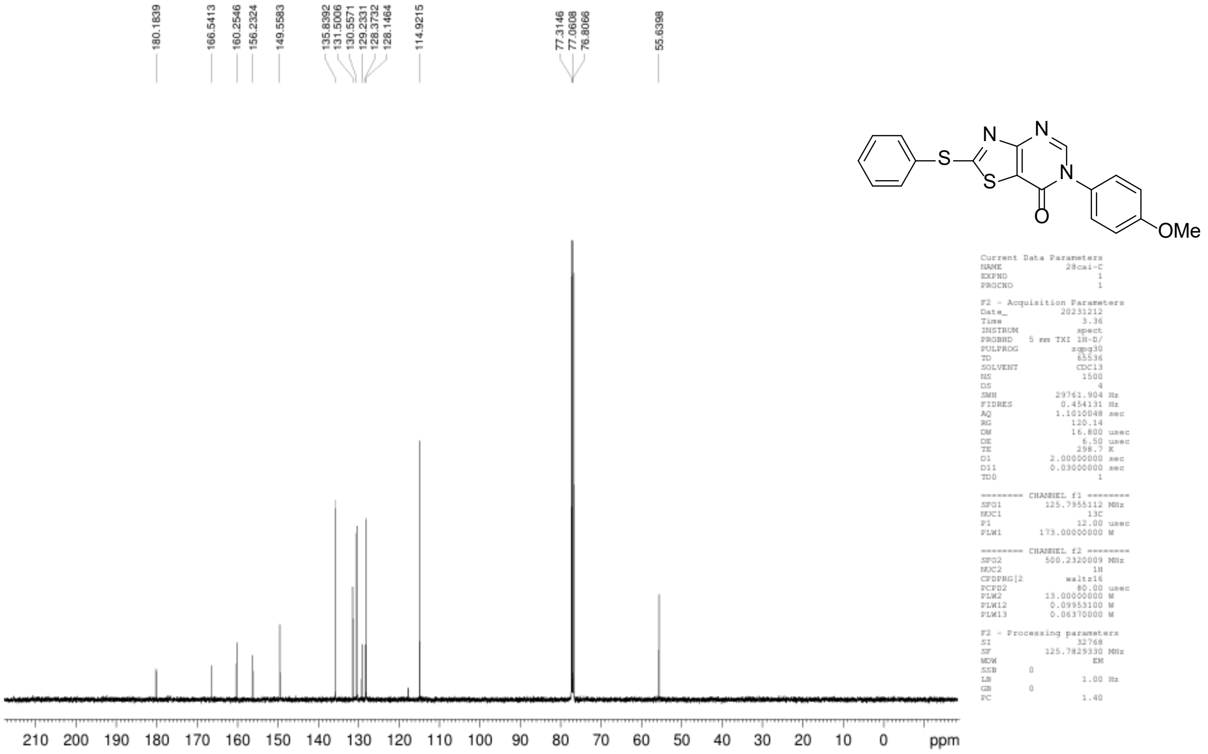

180.1839
166.5413
160.2546
156.2324
149.5583
135.8392
131.5006
130.5571
129.2331
128.3732
128.1464
114.9215
77.3146
77.0608
76.8066
55.6398
N
N
S
S
N
O
OMe
210 200 190 180 170 160 150 140 130 120 110 100 90 80 70 60 50 40 30 20 10 0 ppm

1caj

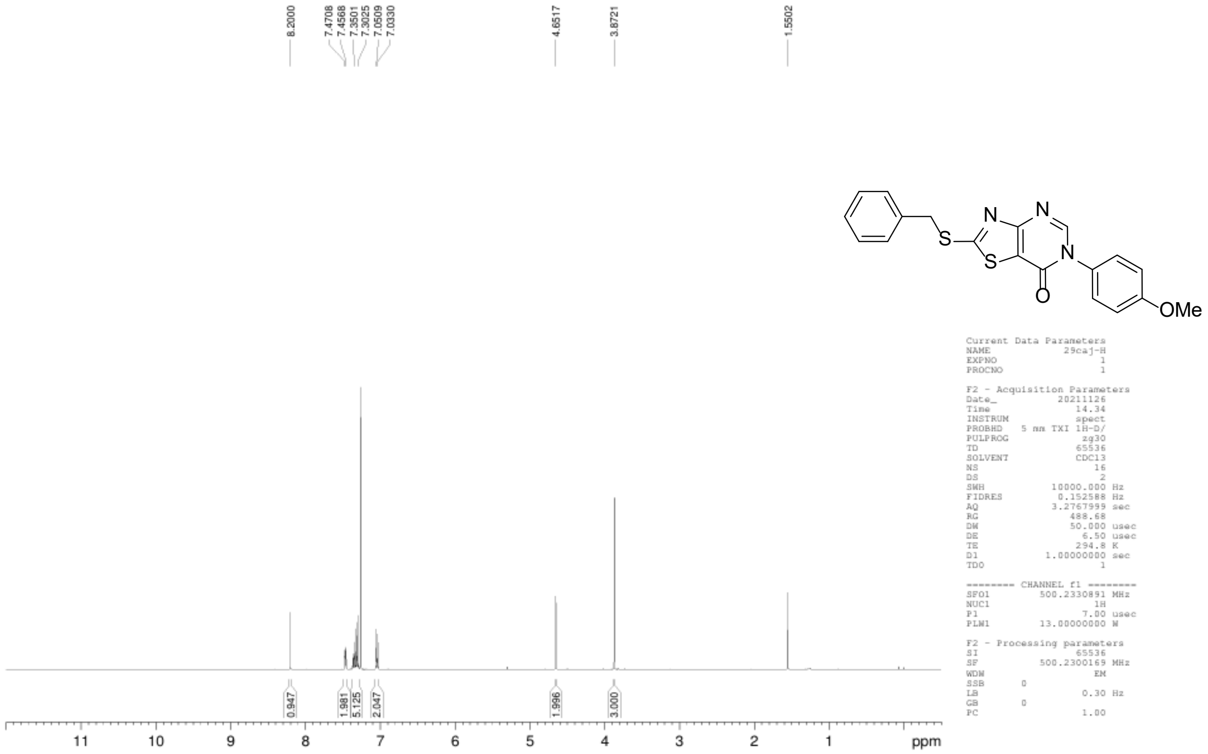
8.2000
7.4708
7.4568
7.3501
7.3025
7.0509
7.0330
4.6517
3.8721
1.5502
N
N
S
S
N
O
OMe
Current Data Parameters
NAME 29caj-H
EXPNO 1
PROCNO 1
F2 - Acquisition Parameters
Date_ 20211126
Time 14.34
INSTRUM spect
PROBHD 5 mm TXI 1H-D/
PULPROG zg30
TD 65536
SOLVENT CDCl3
NS 16
DS 2
SWH 10000.000 Hz
FIDRES 0.152588 Hz
AQ 3.2767999 sec
RG 488.68
DW 50.000 usec
DE 6.50 usec
TE 294.8 K
D1 1.00000000 sec
TD0 1
======== CHANNEL f1 ========
SFO1 500.2330891 MHz
NUC1 1H
P1 7.00 usec
PLW1 13.00000000 W
F2 - Processing parameters
SI 65536
SF 500.2300169 MHz
WDW EM
SSB 0
LB 0.30 Hz
GB 0
PC 1.00
0.947
1.981
5.125
2.047
1.996
3.000
11
10
9
8
7
6
5
4
3
2
1
ppm

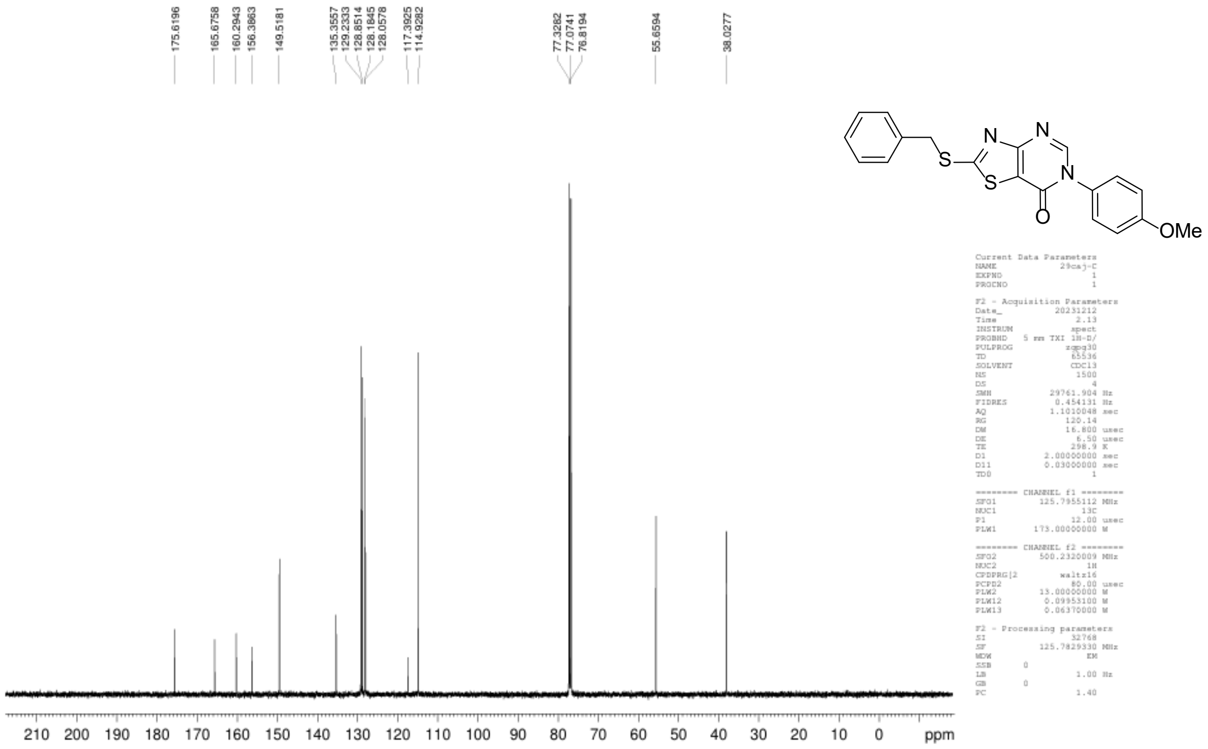
175.6196
165.6758
160.2943
156.3863
149.5181
135.3557
129.2333
128.8514
128.1845
128.0578
117.3925
114.9282
77.3282
77.0741
76.8194
55.6594
38.0277
N
N
S
S
N
O
OMe
Current Data Parameters
NAME 29caj-C
EXPNO 1
PROCNO 1
F2 - Acquisition Parameters
Date_ 20231212
Time 2.13
INSTRUM spect
PROBHD 5 mm TXI 1H-D/
PULPROG zgpg30
TD 65536
SOLVENT CDCl3
NS 1500
DS 4
SWH 29761.904 Hz
FIDRES 0.454131 Hz
AQ 1.1010048 sec
RG 120.14
DW 16.800 usec
DE 6.50 usec
TE 298.9 K
D1 2.00000000 sec
D11 0.03000000 sec
TD0 1
======== CHANNEL f1 ========
SFO1 125.7955112 MHz
NUC1 13C
P1 12.00 usec
PLW1 173.00000000 W
======== CHANNEL f2 ========
SFO2 500.2320009 MHz
NUC2 1H
CPDPRG[2 waltz16
PCPD2 80.00 usec
PLW2 13.00000000 W
PLW12 0.09953100 W
PLW13 0.06370000 W
F2 - Processing parameters
SI 32768
SF 125.7829330 MHz
WDW EM
SSB 0
LB 1.00 Hz
GB 0
PC 1.40
210
200
190
180
170
160
150
140
130
120
110
100
90
80
70
60
50
40
30
20
10
0
ppm

1cba

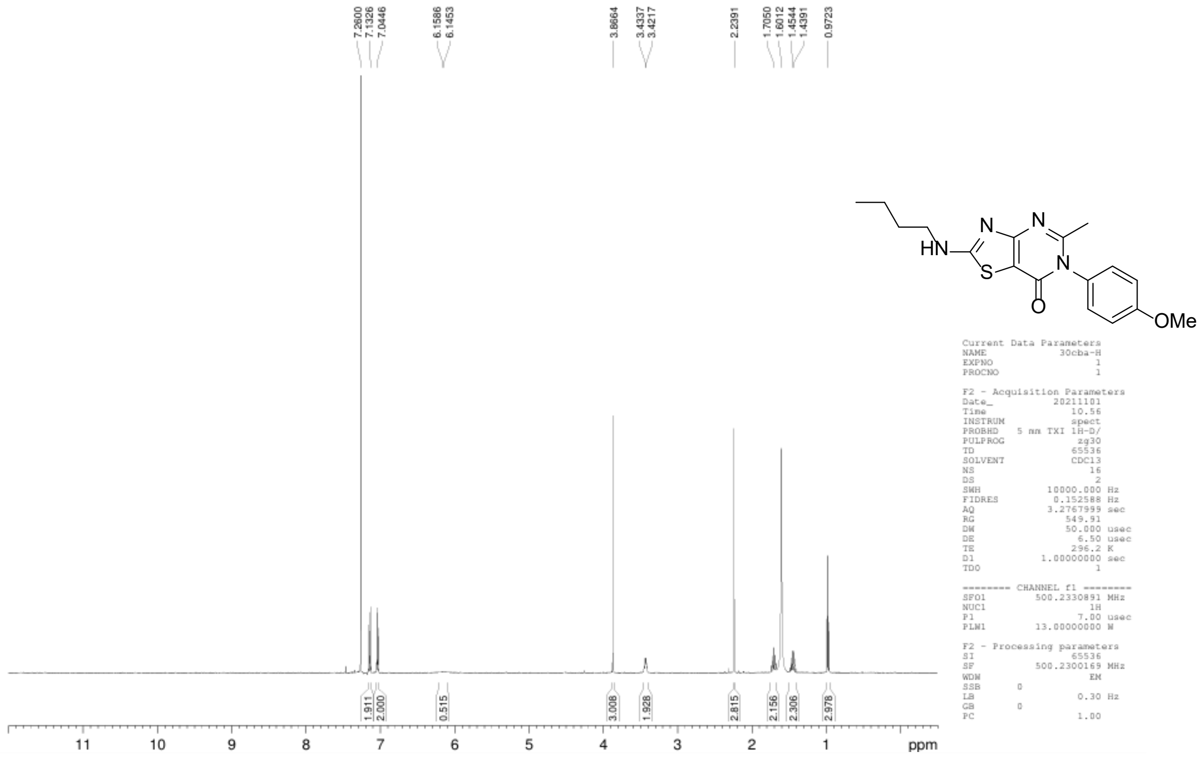

7.2600
7.1326
7.0446
6.1586
6.1453
3.8664
3.4337
3.4217
2.2391
1.7050
1.6012
1.4544
1.4391
0.9723
HN
N
N
S
N
O
OMe
Current Data Parameters
NAME 30cba-H
EXPNO 1
PROCNO 1
F2 - Acquisition Parameters
Date_ 20211101
Time 10.56
INSTRUM spect
PROBHD 5 mm TXI 1H-D/
PULPROG zg30
TD 65536
SOLVENT CDCl3
NS 16
DS 2
SWH 10000.000 Hz
FIDRES 0.152588 Hz
AQ 3.2767999 sec
RG 549.91
DW 50.000 usec
DE 6.50 usec
TE 296.2 K
D1 1.00000000 sec
TD0 1
======== CHANNEL f1 ========
SFO1 500.2330891 MHz
NUC1 1H
P1 7.00 usec
PLW1 13.00000000 W
F2 - Processing parameters
SI 65536
SF 500.2300169 MHz
WDW EM
SSB 0
LB 0.30 Hz
GB 0
PC 1.00
1.911
2.000
0.515
3.008
1.928
2.815
2.156
2.306
2.978
11
10
9
8
7
6
5
4
3
2
1
ppm

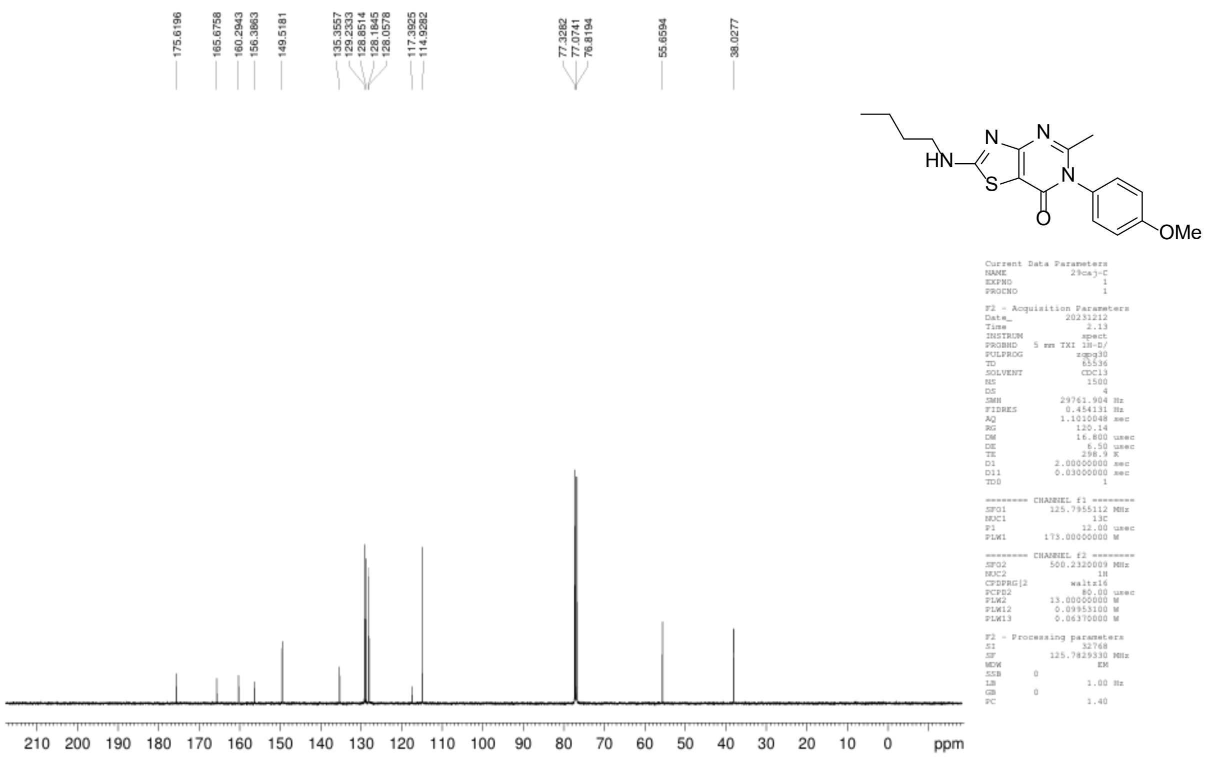

175.6196
165.6758
160.2943
156.3863
149.5181
135.3557
129.2333
128.8514
128.1845
128.0578
117.3925
114.9282
77.3282
77.0741
76.8194
55.6594
38.0277
HN
N
N
S
N
O
OMe
Current Data Parameters
NAME 29caj-C
EXPNO 1
PROCNO 1
F2 - Acquisition Parameters
Date_ 20231212
Time 2.13
INSTRUM spect
PROBHD 5 mm TXI 1H-D/
PULPROG zgpg30
TD 65536
SOLVENT CDCl3
NS 1500
DS 4
SWH 29761.904 Hz
FIDRES 0.454131 Hz
AQ 1.1010048 sec
RG 120.14
DW 16.800 usec
DE 6.50 usec
TE 298.9 K
D1 2.00000000 sec
D11 0.03000000 sec
TD0 1
======== CHANNEL f1 ========
SFO1 125.7955112 MHz
NUC1 13C
P1 12.00 usec
PLW1 173.00000000 W
======== CHANNEL f2 ========
SFO2 500.2320009 MHz
NUC2 1H
CPDPRG[2 waltz16
PCPD2 80.00 usec
PLW2 13.00000000 W
PLW12 0.09953100 W
PLW13 0.06370000 W
F2 - Processing parameters
SI 32768
SF 125.7829330 MHz
WDW EM
SSB 0
LB 1.00 Hz
GB 0
PC 1.40
210
200
190
180
170
160
150
140
130
120
110
100
90
80
70
60
50
40
30
20
10
0
ppm

1cbb

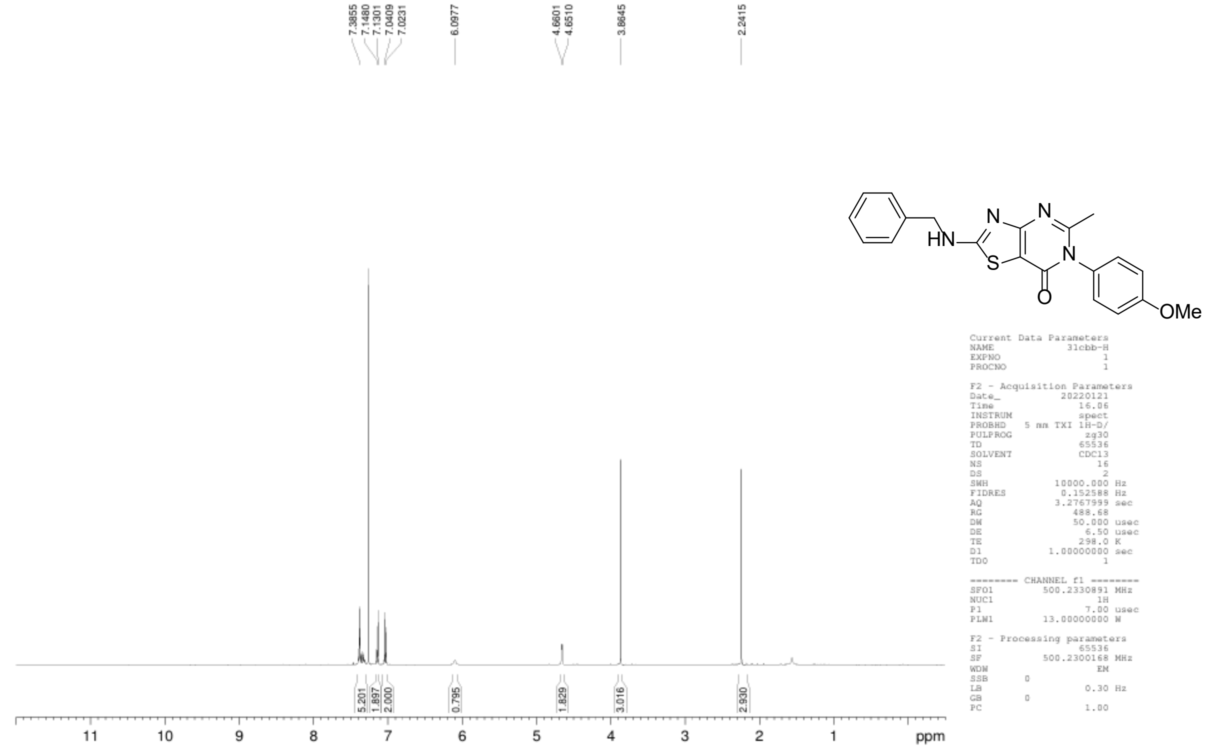
7.3855
7.1480
7.1301
7.0409
7.0231
6.0977
4.6601
4.6510
3.8645
2.2415
N
N
HN
S
N
O
OMe
Current Data Parameters
NAME 31cbb-H
EXPNO 1
PROCNO 1
F2 - Acquisition Parameters
Date_ 20220121
Time 16.06
INSTRUM spect
PROBHD 5 mm TXI 1H-D/
PULPROG zg30
TD 65536
SOLVENT CDCl3
NS 16
DS 2
SWH 10000.000 Hz
FIDRES 0.152588 Hz
AQ 3.2767999 sec
RG 488.68
DW 50.000 usec
DE 6.50 usec
TE 298.0 K
D1 1.00000000 sec
TD0 1
======== CHANNEL f1 ========
SFO1 500.2330891 MHz
NUC1 1H
P1 7.00 usec
PLW1 13.00000000 W
F2 - Processing parameters
SI 65536
SF 500.2300168 MHz
WDW EM
SSB 0
LB 0.30 Hz
GB 0
PC 1.00
5.201
1.897
2.000
0.795
1.829
3.016
2.930
11
10
9
8
7
6
5
4
3
2
1
ppm

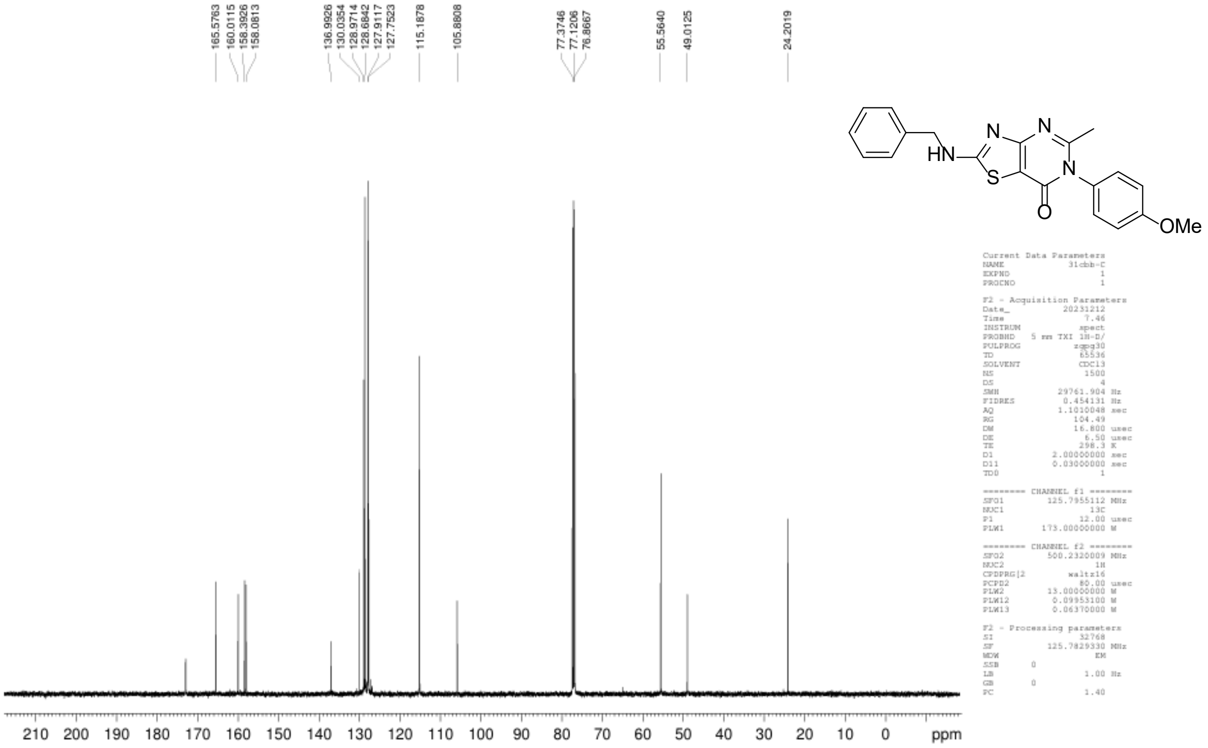
165.5763
160.0115
158.3926
158.0813
136.9926
130.0354
128.9714
128.6842
127.9117
127.7523
115.1878
105.8808
77.3746
77.1206
76.8667
55.5640
49.0125
24.2019
N
N
HN
S
N
O
OMe
Current Data Parameters
NAME 31cbb-C
EXPNO 1
PROCNO 1
F2 - Acquisition Parameters
Date_ 20231212
Time 7.46
INSTRUM spect
PROBHD 5 mm TXI 1H-D/
PULPROG zgpg30
TD 65536
SOLVENT CDCl3
NS 1500
DS 4
SWH 29761.904 Hz
FIDRES 0.454131 Hz
AQ 1.1010048 sec
RG 104.49
DW 16.800 usec
DE 6.50 usec
TE 298.3 K
D1 2.00000000 sec
D11 0.03000000 sec
TD0 1
======== CHANNEL f1 ========
SFO1 125.7955112 MHz
NUC1 13C
P1 12.00 usec
PLW1 173.00000000 W
======== CHANNEL f2 ========
SFO2 500.2320009 MHz
NUC2 1H
CPDPRG[2 waltz16
PCPD2 80.00 usec
PLW2 13.00000000 W
PLW12 0.09953100 W
PLW13 0.06370000 W
F2 - Processing parameters
SI 32768
SF 125.7829330 MHz
WDW EM
SSB 0
LB 1.00 Hz
GB 0
PC 1.40
210
200
190
180
170
160
150
140
130
120
110
100
90
80
70
60
50
40
30
20
10
0
ppm

1cbc

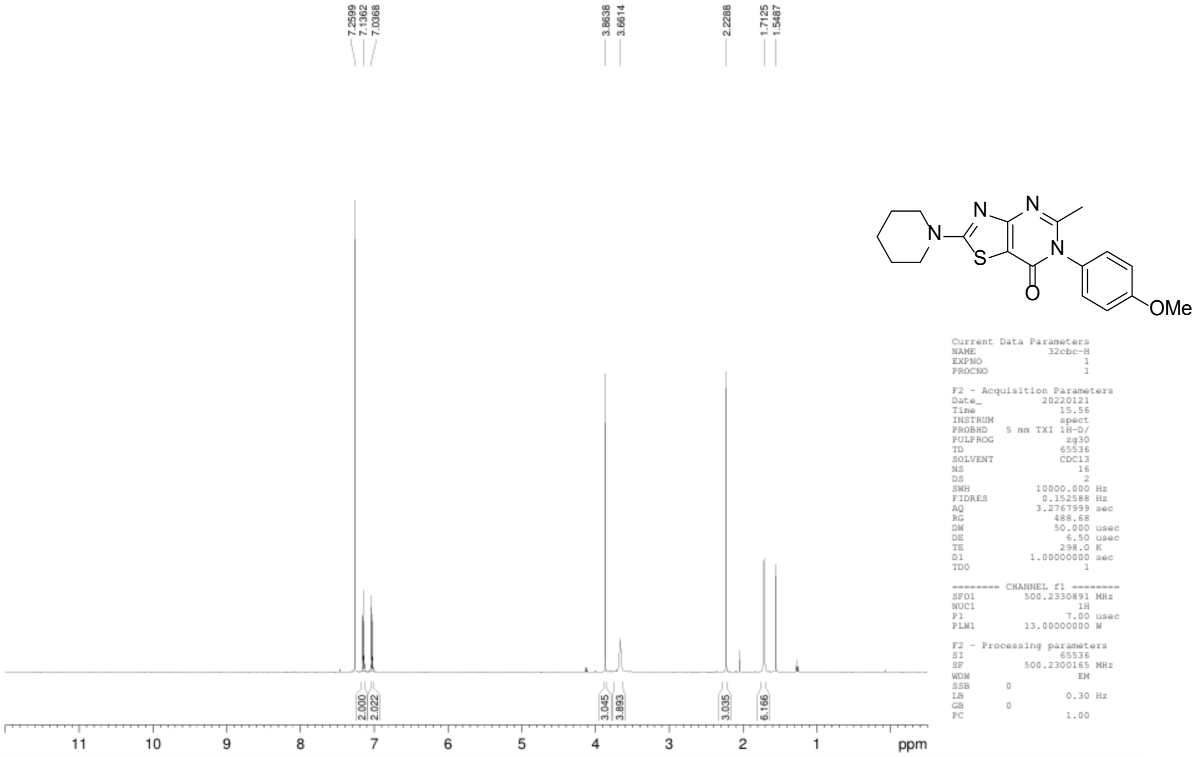
7.2599
7.1362
7.0368
3.8638
3.6614
2.2288
1.7125
1.5487
2.000
2.022
3.045
3.893
3.035
6.166
ppm
N
N
N
N
S
O
OMe
Current Data Parameters
NAME 32cbc-H
EXPNO 1
PROCNO 1
F2 - Acquisition Parameters
Date_ 20220121
Time 15.56
INSTRUM spect
PROBHD 5 mm TXI 1H-D/
PULPROG zg30
TD 65536
SOLVENT CDCl3
NS 16
DS 2
SWH 10000.000 Hz
FIDRES 0.152588 Hz
AQ 3.2767999 sec
RG 488.68
DW 50.000 usec
DE 6.50 usec
TE 298.0 K
D1 1.00000000 sec
TD0 1
======== CHANNEL f1 ========
SFO1 500.2330891 MHz
NUC1 1H
P1 7.00 usec
PLW1 13.00000000 W
F2 - Processing parameters
SI 65536
SF 500.2300165 MHz
WDW EM
SSB 0
LB 0.30 Hz
GB 0
PC 1.00

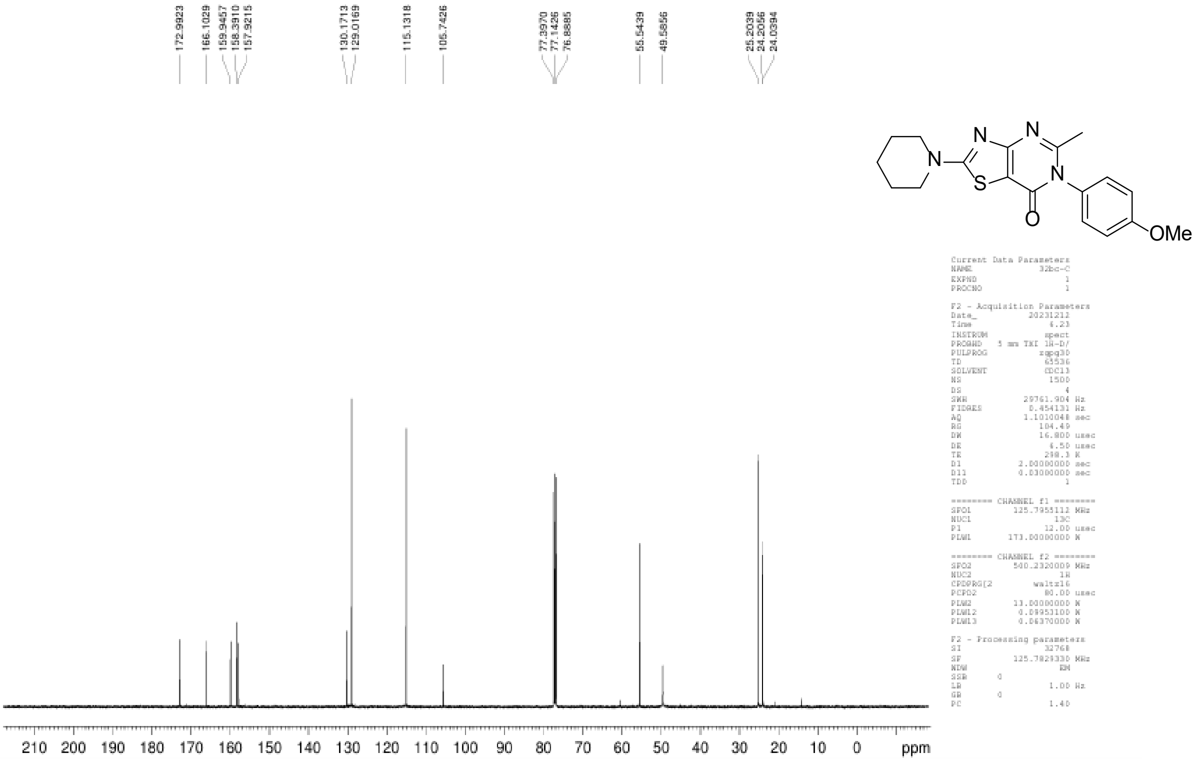
172.9923
166.1029
159.9457
158.3910
157.9215
130.1713
129.0169
115.1318
105.7426
77.3970
77.1426
76.8885
55.5439
49.5856
25.2039
24.2056
24.0394
ppm
N
N
N
N
S
O
OMe
Current Data Parameters
NAME 32bc-C
EXPNO 1
PROCNO 1
F2 - Acquisition Parameters
Date_ 20231212
Time 6.23
INSTRUM spect
PROBHD 5 mm TXI 1H-D/
PULPROG zgpg30
TD 65536
SOLVENT CDCl3
NS 1500
DS 4
SWH 29761.904 Hz
FIDRES 0.454131 Hz
AQ 1.1010048 sec
RG 104.49
DW 16.800 usec
DE 6.50 usec
TE 298.3 K
D1 2.00000000 sec
D11 0.03000000 sec
TD0 1
======== CHANNEL f1 ========
SFO1 125.7955113 MHz
NUC1 13C
P1 12.00 usec
PLW1 173.00000000 W
======== CHANNEL f2 ========
SFO2 500.2320009 MHz
NUC2 1H
CPDPRG[2 waltz16
PCPD2 80.00 usec
PLW2 13.00000000 W
PLW12 0.08953100 W
PLW13 0.06370000 W
F2 - Processing parameters
SI 32768
SF 125.7824330 MHz
WDW EM
SSB 0
LB 1.00 Hz
GB 0
PC 1.40

1cbd

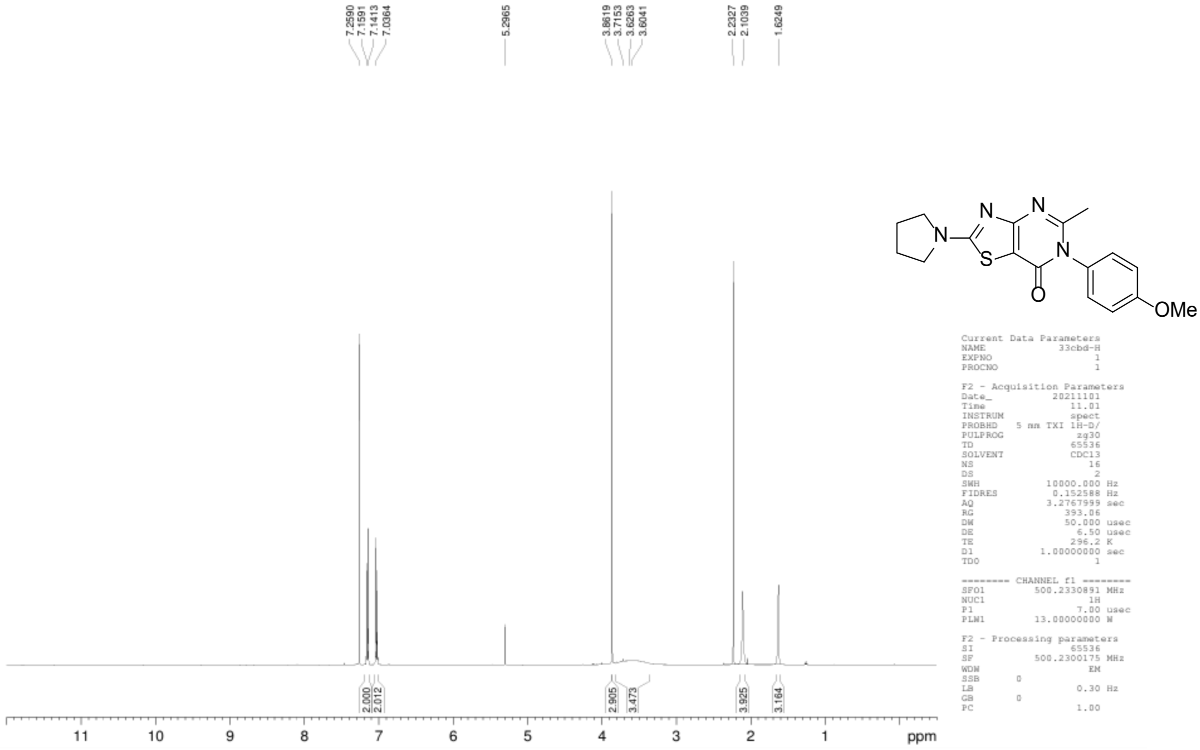
7.2590
7.1591
7.1413
7.0364
5.2965
3.8619
3.7153
3.6263
3.6041
2.2327
2.1039
1.6249
N
N
N
N
S
O
OMe
2.000
2.012
2.905
3.473
3.925
3.164
11 10 9 8 7 6 5 4 3 2 1 ppm

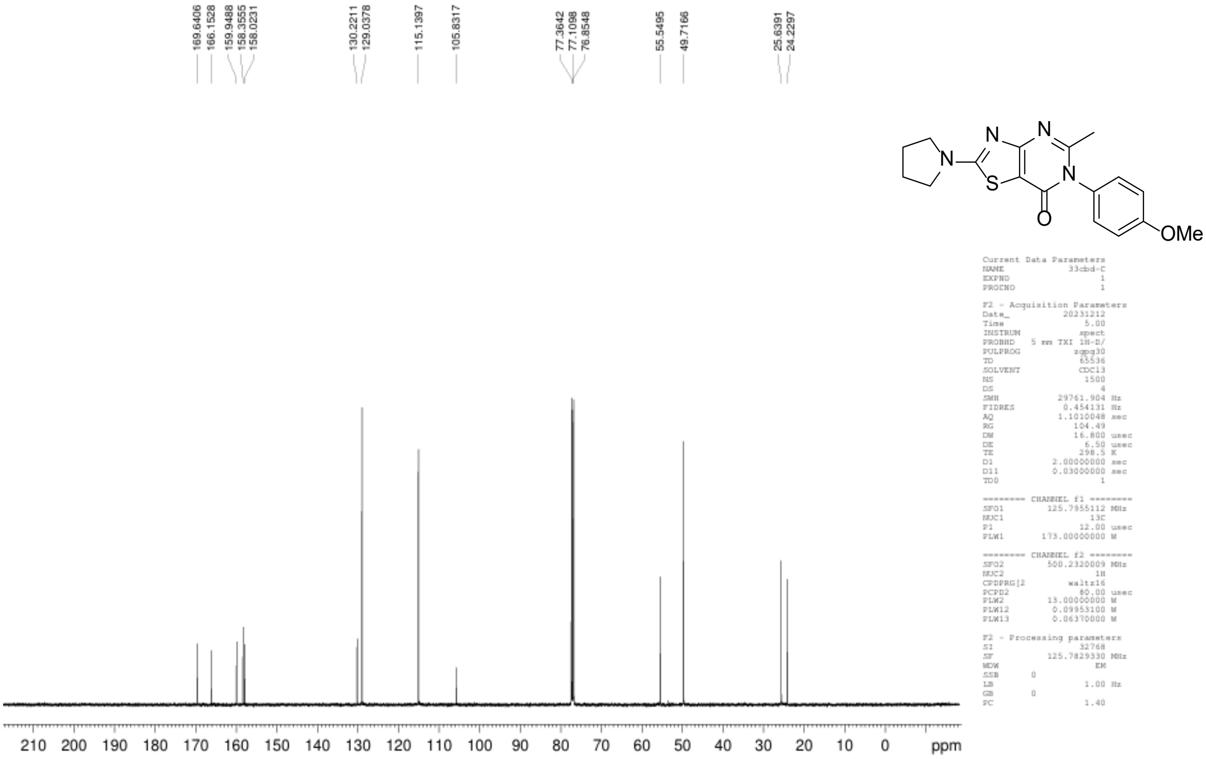
169.6406
166.1528
159.9488
158.3555
158.0231
130.2211
129.0378
115.1397
105.8317
77.3642
77.1098
76.8548
55.5495
49.7166
25.6391
24.2297
N
N
N
N
S
O
OMe
210 200 190 180 170 160 150 140 130 120 110 100 90 80 70 60 50 40 30 20 10 0 ppm

1cbe

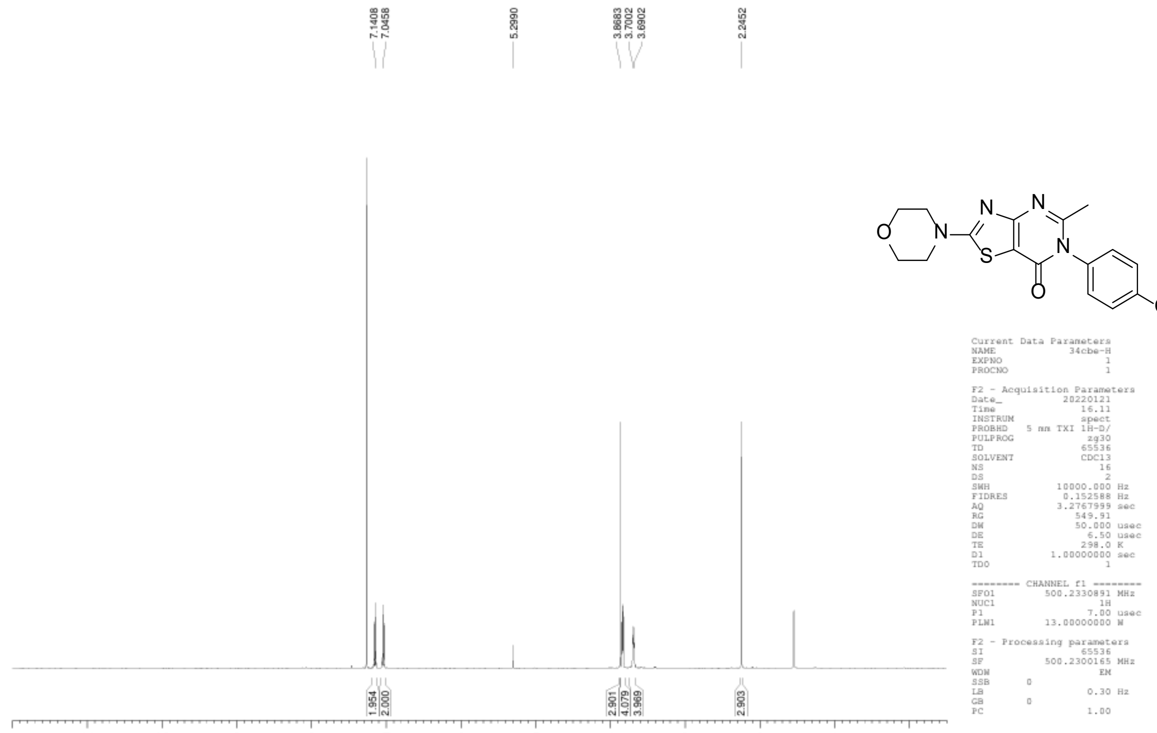
7.1408
7.0458
5.2990
3.8683
3.7002
3.6902
2.2452
1.954
2.000
2.901
4.079
3.969
2.903
11 10 9 8 7 6 5 4 3 2 1 ppm
N
N
O
N
S
N
O
OMe

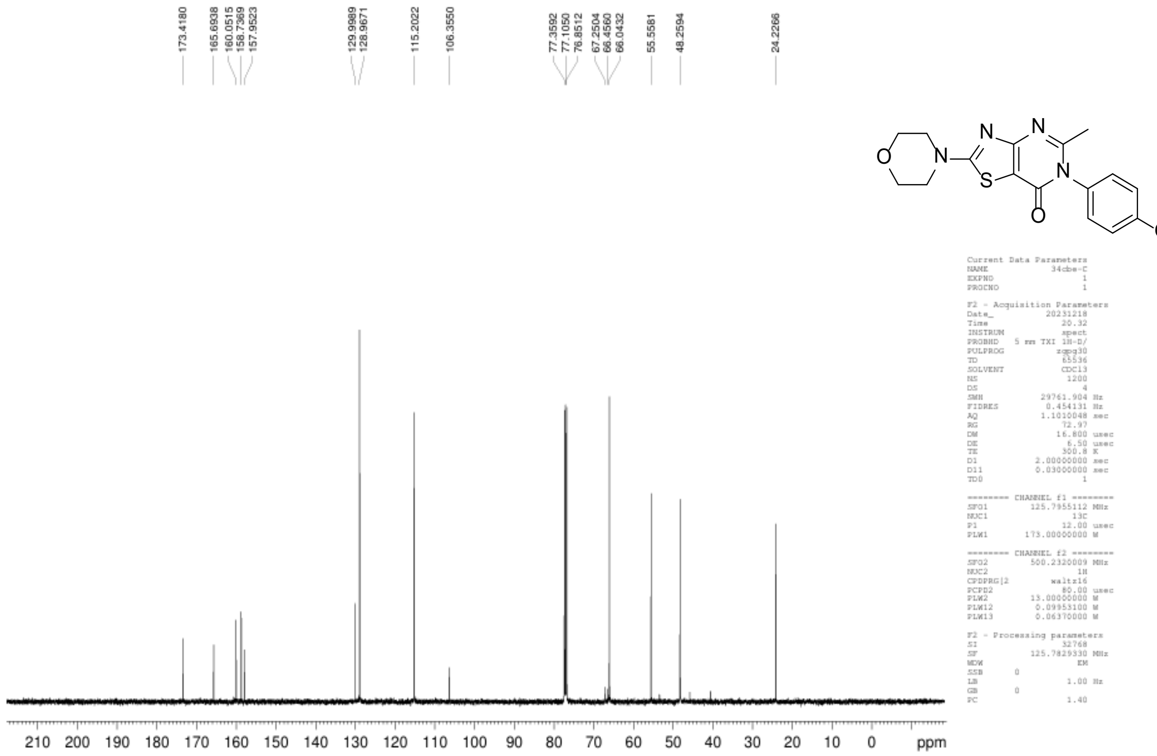
173.4180
165.6938
160.0515
158.7369
157.9523
129.9989
128.9671
115.2022
106.3550
77.3592
77.1050
76.8512
67.2504
66.4560
66.0432
55.5581
48.2594
24.2266
210 200 190 180 170 160 150 140 130 120 110 100 90 80 70 60 50 40 30 20 10 0 ppm
N
N
O
N
S
N
O
OMe

1cbf

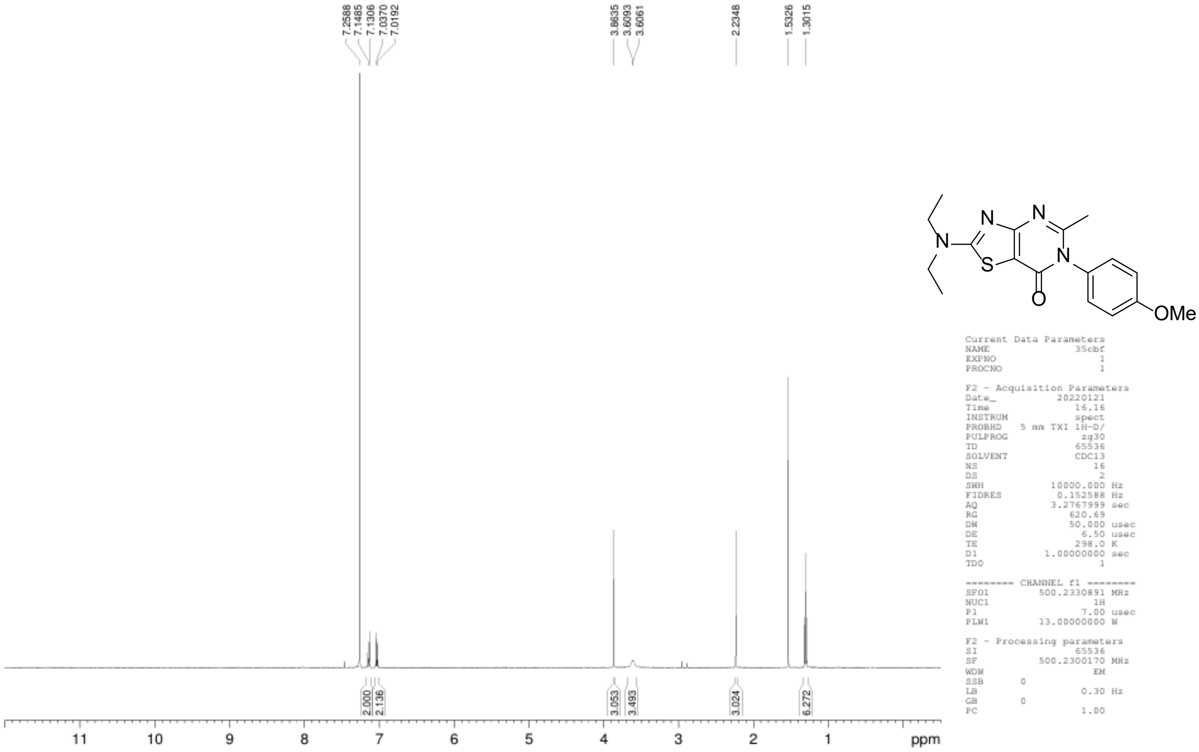
7.2588
7.1485
7.1306
7.0370
7.0192
3.8635
3.6093
3.6061
2.2348
1.5326
1.3015
2.000
2.136
3.053
3.493
3.024
6.272
ppm
N
S
O
OMe

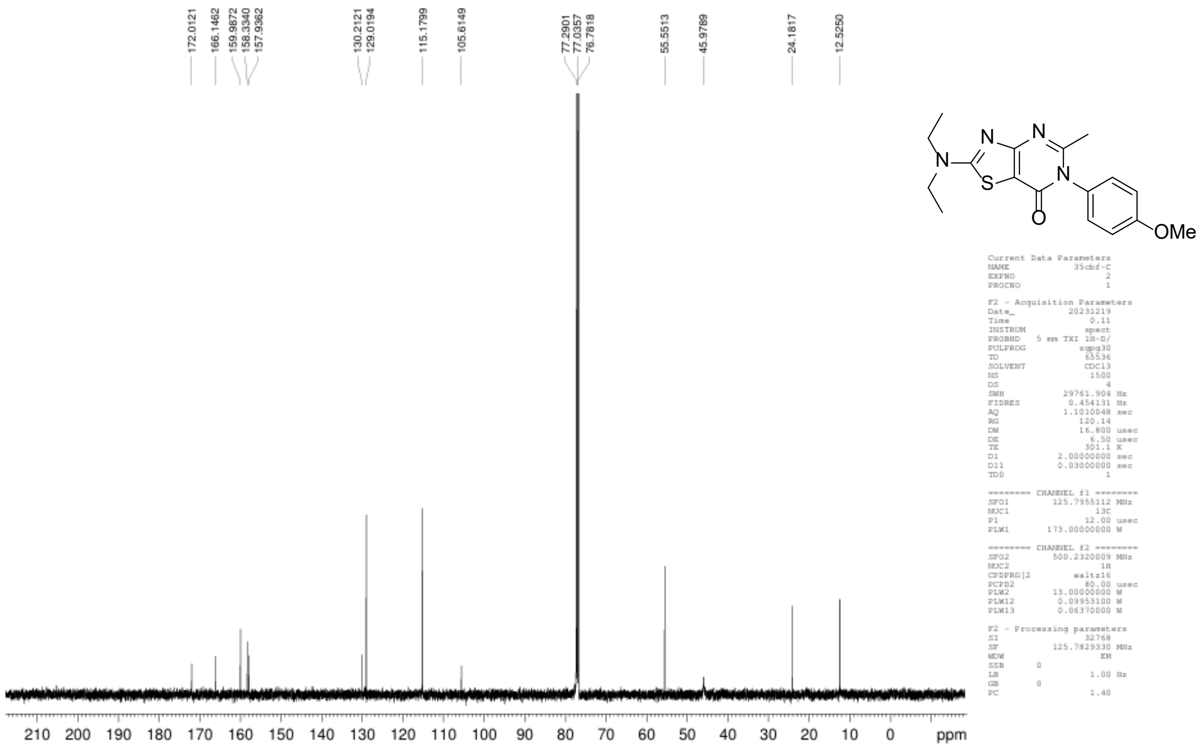
172.0121
166.1462
159.9872
158.3340
157.9362
130.2121
129.0194
115.1799
105.6149
77.2901
77.0357
76.7818
55.5513
45.9789
24.1817
12.5250
ppm
N
S
O
OMe

1cbg

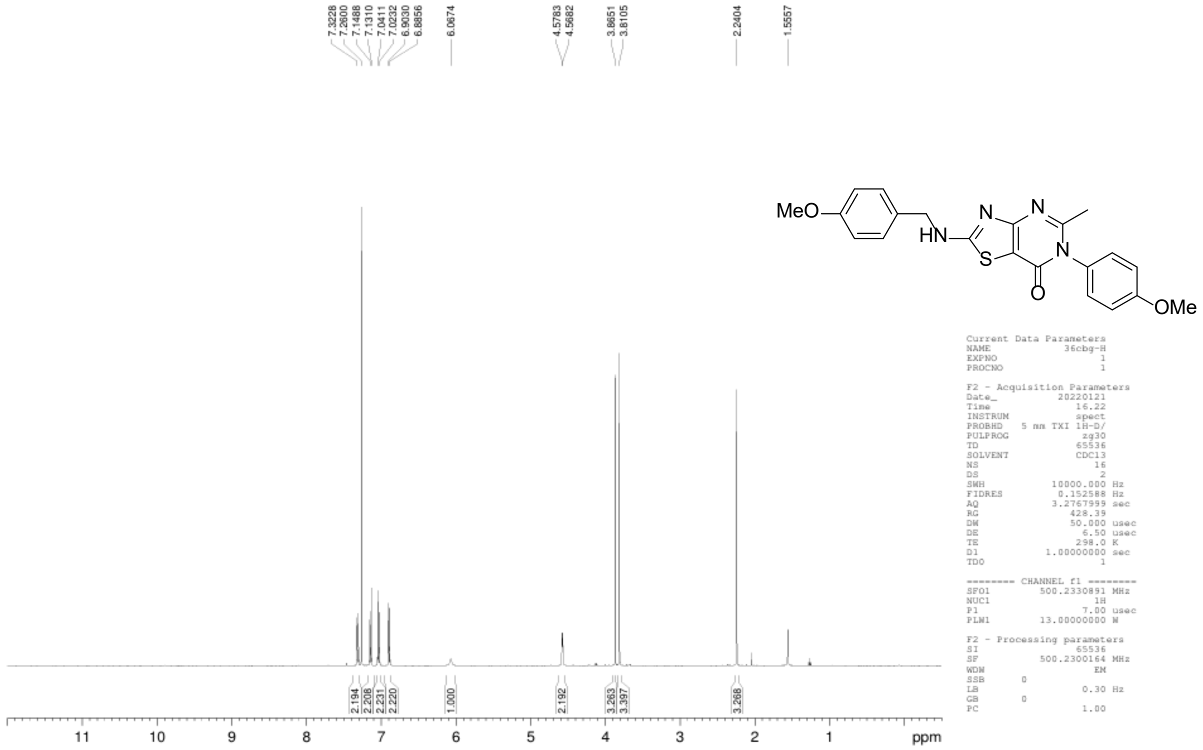
7.3228
7.2600
7.1488
7.1310
7.0411
7.0232
6.9030
6.8856
6.0674
4.5783
4.5682
3.8651
3.8105
2.2404
1.5557
MeO
HN
N
N
S
N
O
OMe
2.194
2.208
2.231
2.220
1.000
2.192
3.263
3.397
3.268
ppm

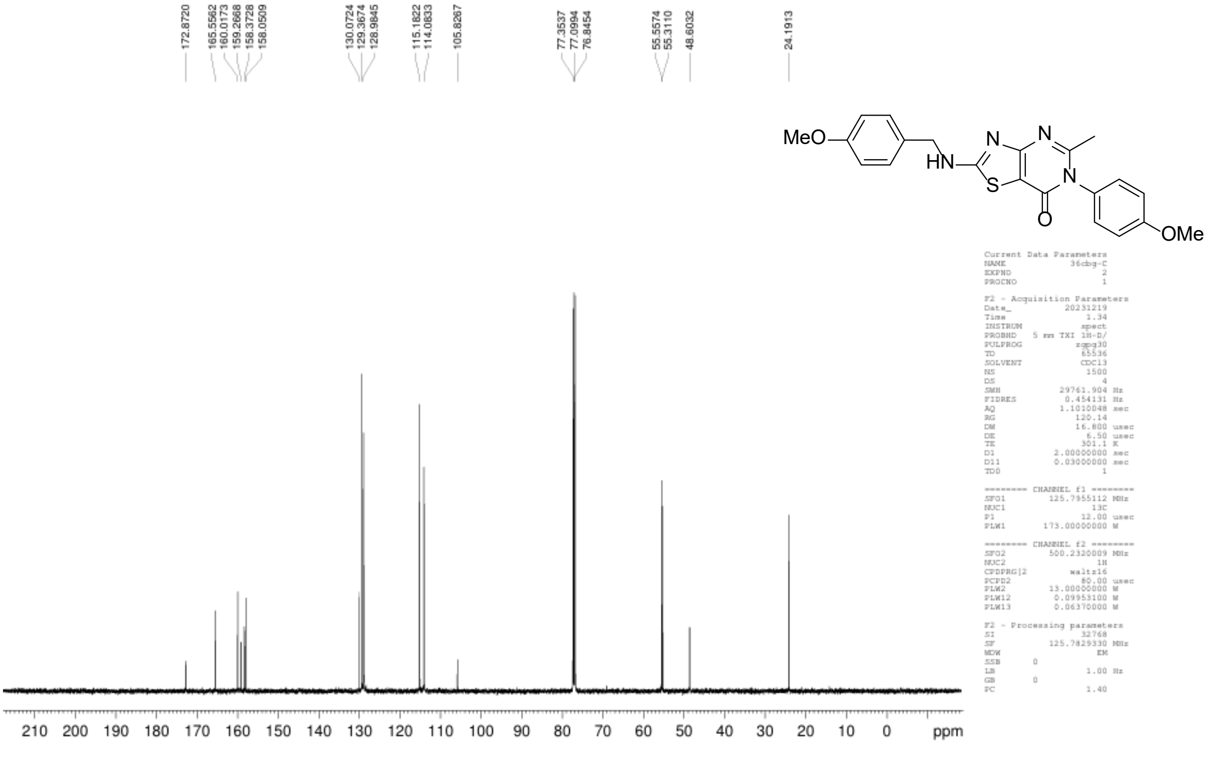
172.8720
165.5562
160.0173
159.2668
158.3728
158.0509
130.0724
129.3674
128.9845
115.1822
114.0833
105.8267
77.3537
77.0994
76.8454
55.5574
55.3110
48.6032
24.1913
MeO
HN
N
N
S
N
O
OMe
ppm

1cbh

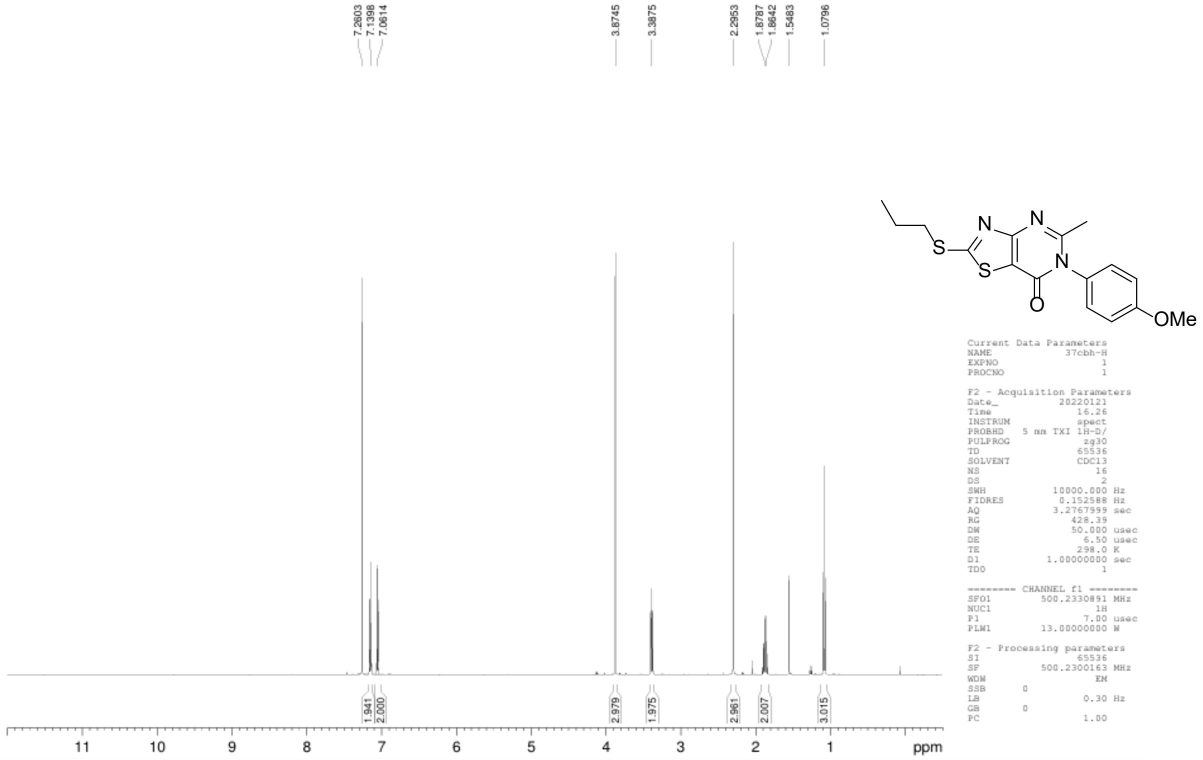

7.2603
7.1398
7.0614
3.8745
3.3875
2.2953
1.8787
1.8642
1.5483
1.0796
N
N
S
S
N
O
OMe
Current Data Parameters
NAME 37cbh-H
EXPNO 1
PROCNO 1
F2 - Acquisition Parameters
Date_ 20220121
Time 16.26
INSTRUM spect
PROBHD 5 mm TXI 1H-D/
PULPROG zg30
TD 65536
SOLVENT CDCl3
NS 16
DS 2
SWH 10000.000 Hz
FIDRES 0.152588 Hz
AQ 3.2767999 sec
RG 428.39
DW 50.000 usec
DE 6.50 usec
TE 298.0 K
D1 1.00000000 sec
TD0 1
======== CHANNEL f1 ========
SFO1 500.2330891 MHz
NUC1 1H
P1 7.00 usec
PLW1 13.00000000 W
F2 - Processing parameters
SI 65536
SF 500.2300163 MHz
WDW EM
SSB 0
LB 0.30 Hz
GB 0
PC 1.00
1.941
2.000
2.979
1.975
2.961
2.007
3.015
11
10
9
8
7
6
5
4
3
2
1
ppm

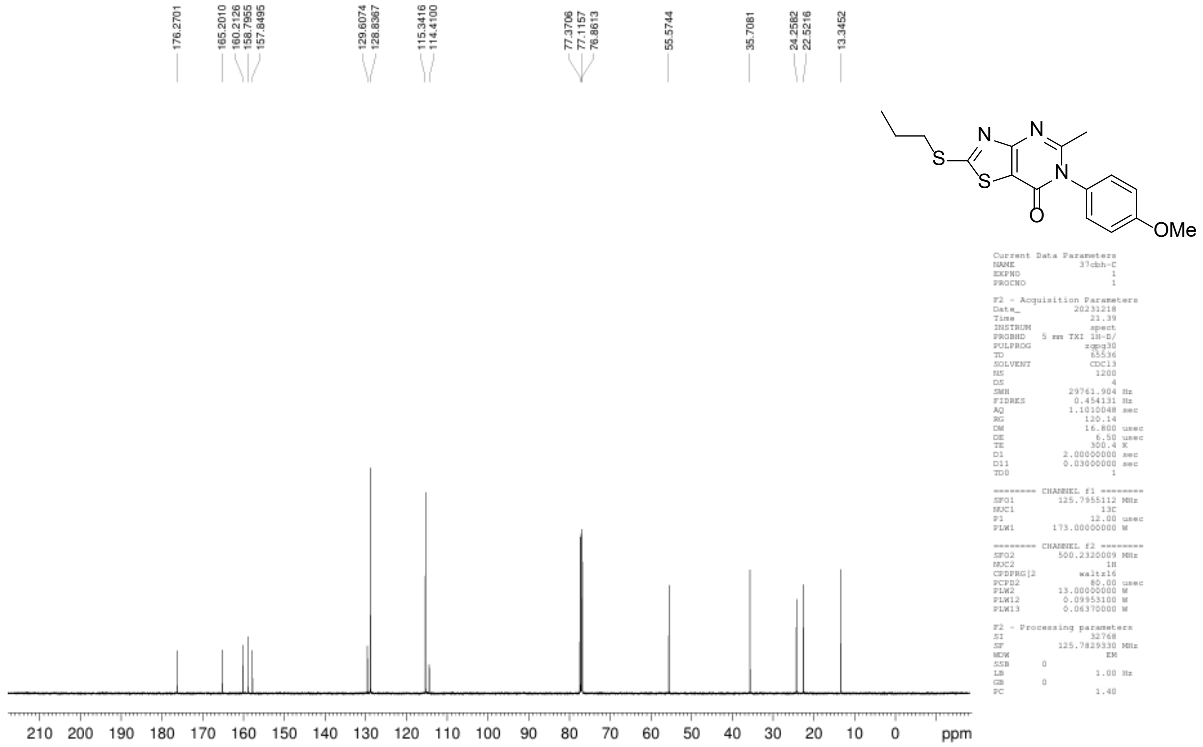

176.2701
165.2010
160.2126
158.7955
157.8495
129.6074
128.8367
115.3416
114.4100
77.3706
77.1157
76.8613
55.5744
35.7081
24.2582
22.5216
13.3452
N
N
S
S
N
O
OMe
Current Data Parameters
NAME 37cbh-C
EXPNO 1
PROCNO 1
F2 - Acquisition Parameters
Date_ 20231218
Time 21.39
INSTRUM spect
PROBHD 5 mm TXI 1H-D/
PULPROG zgpg30
TD 65536
SOLVENT CDCl3
NS 1200
DS 4
SWH 29761.904 Hz
FIDRES 0.454131 Hz
AQ 1.1010048 sec
RG 120.14
DW 16.800 usec
DE 6.50 usec
TE 300.4 K
D1 2.00000000 sec
D11 0.03000000 sec
TD0 1
======== CHANNEL f1 ========
SFO1 125.7955112 MHz
NUC1 13C
P1 12.00 usec
PLW1 173.00000000 W
======== CHANNEL f2 ========
SFO2 500.2320009 MHz
NUC2 1H
CPDPRG[2 waltz16
PCPD2 80.00 usec
PLW2 13.00000000 W
PLW12 0.09953100 W
PLW13 0.06370000 W
F2 - Processing parameters
SI 32768
SF 125.7829330 MHz
WDW EM
SSB 0
LB 1.00 Hz
GB 0
PC 1.40
210
200
190
180
170
160
150
140
130
120
110
100
90
80
70
60
50
40
30
20
10
0
ppm

1cbi

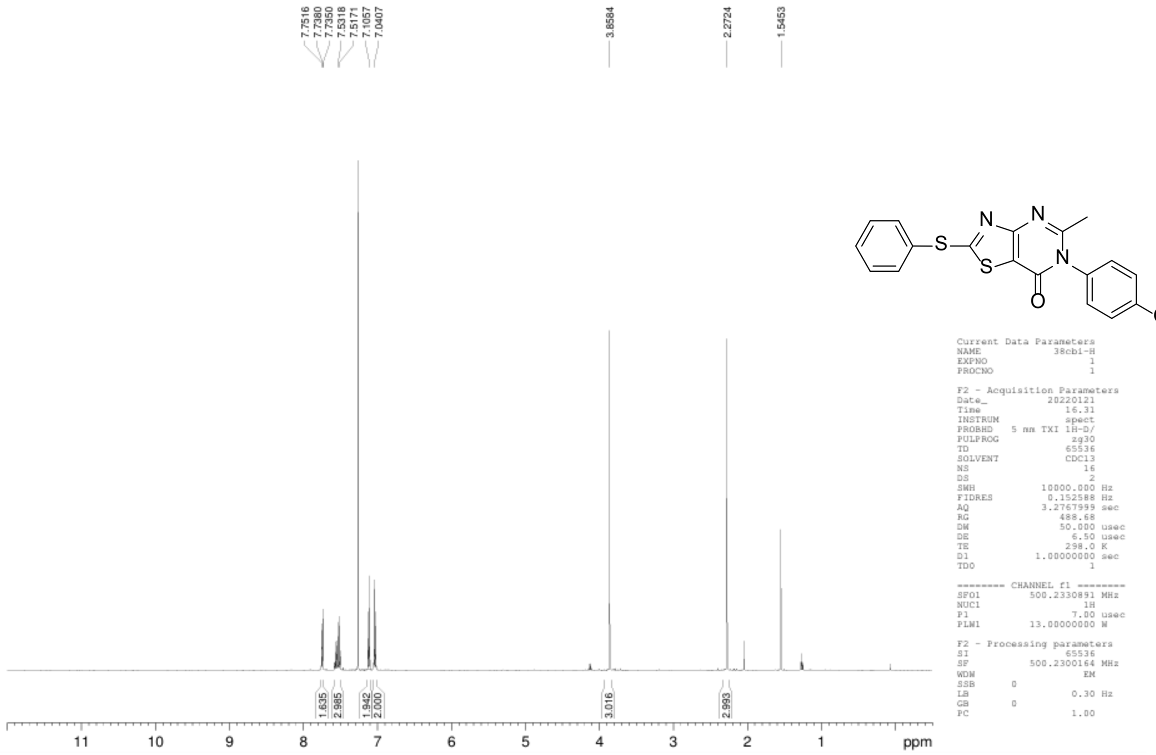
7.7516
7.7380
7.7350
7.5318
7.5171
7.1057
7.0407
3.8584
2.2724
1.5453
1.635
2.985
1.942
2.000
3.016
2.993
ppm
OMe
Current Data Parameters
NAME 38cbi-H

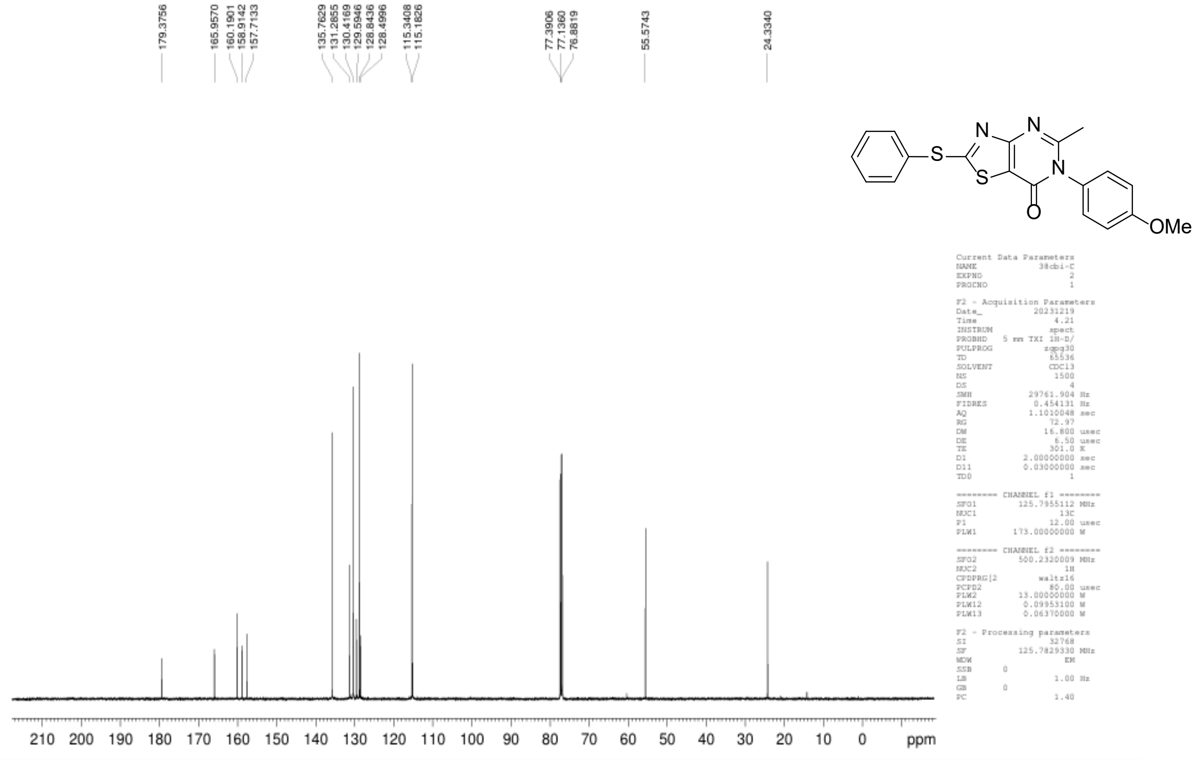
179.3756
165.9570
160.1901
158.9142
157.7133
135.7629
131.2855
130.4169
129.5946
128.8436
128.4996
115.3408
115.1826
77.3906
77.1360
76.8819
55.5743
24.3340
ppm
OMe
Current Data Parameters
NAME 38cbi-C

1cbj

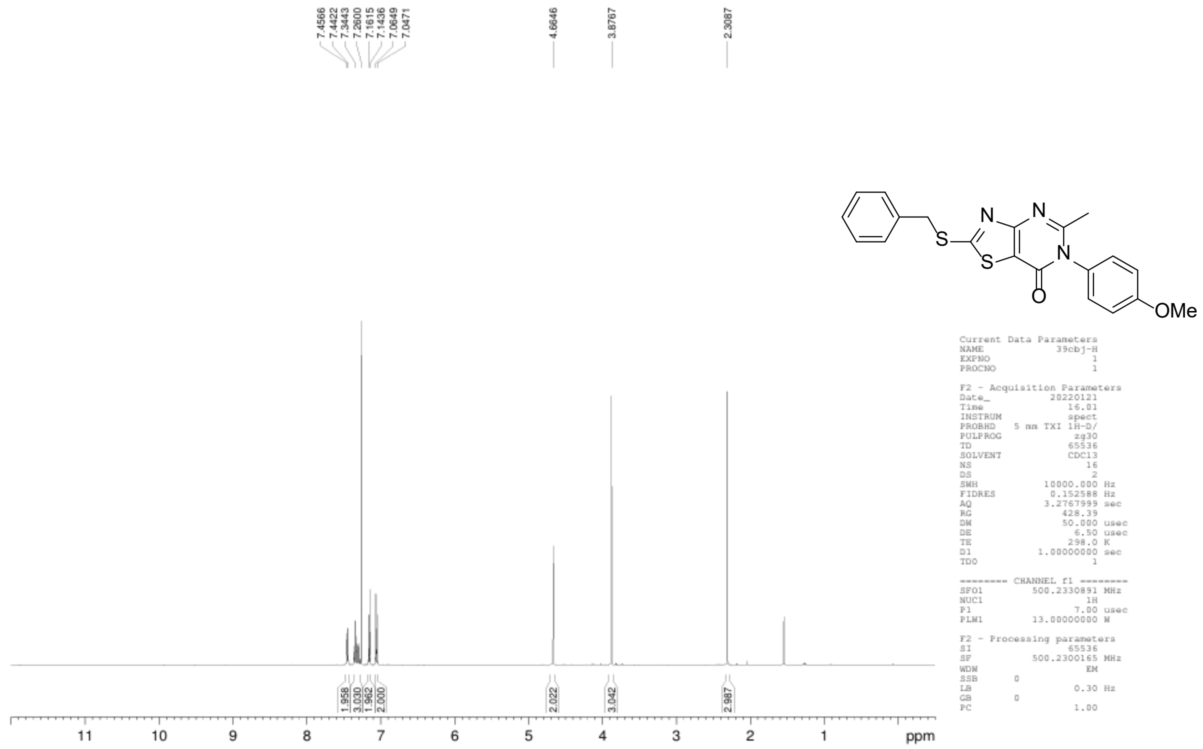
7.4566
7.4422
7.3443
7.2600
7.1615
7.1436
7.0649
7.0471
4.6646
3.8767
2.3087
N
N
S
S
N
O
OMe
Current Data Parameters
NAME 39cbj-H
EXPNO 1
PROCNO 1
F2 - Acquisition Parameters
Date_ 20220121
Time 16.01
INSTRUM spect
PROBHD 5 mm TXI 1H-D/
PULPROG zg30
TD 65536
SOLVENT CDCl3
NS 16
DS 2
SWH 10000.000 Hz
FIDRES 0.152588 Hz
AQ 3.2767999 sec
RG 428.39
DW 50.000 usec
DE 6.50 usec
TE 298.0 K
D1 1.00000000 sec
TD0 1
======== CHANNEL f1 ========
SFO1 500.2330891 MHz
NUC1 1H
P1 7.00 usec
PLW1 13.00000000 W
F2 - Processing parameters
SI 65536
SF 500.2300165 MHz
WDW EM
SSB 0
LB 0.30 Hz
GB 0
PC 1.00
1.958
3.030
1.962
2.000
2.022
3.042
2.987
11
10
9
8
7
6
5
4
3
2
1
ppm

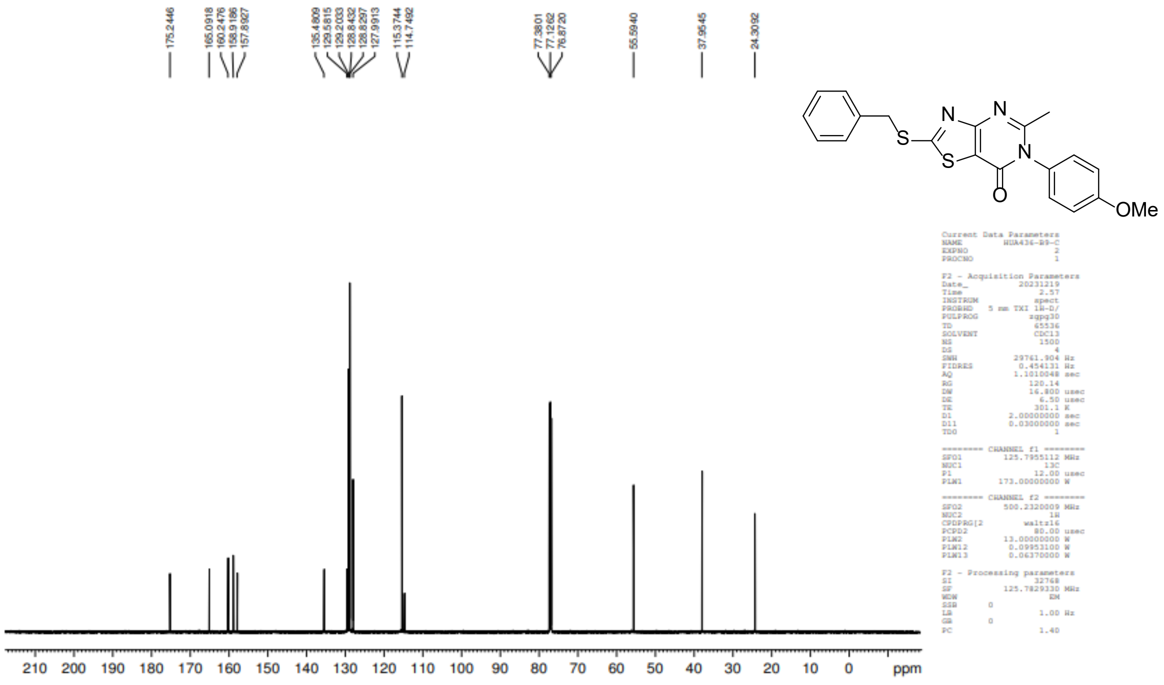
175.2446
165.0918
160.2476
158.9186
157.8927
135.4809
129.5815
129.2033
128.8432
128.8297
127.9913
115.3744
114.7492
77.3801
77.1262
76.8720
55.5940
37.9545
24.3092
N
N
S
S
N
O
OMe
210
200
190
180
170
160
150
140
130
120
110
100
90
80
70
60
50
40
30
20
10
0
ppm

1bba

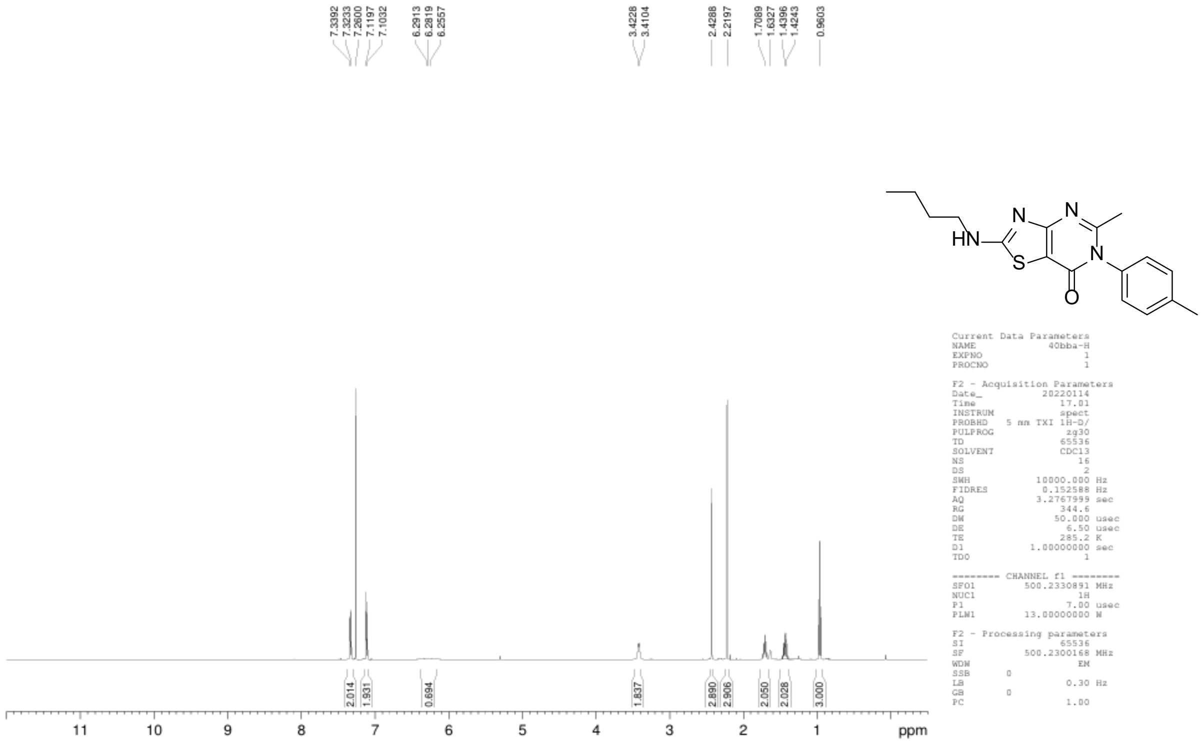
7.3392
7.3233
7.2600
7.1197
7.1032
6.2913
6.2819
6.2557
3.4228
3.4104
2.4288
2.2197
1.7089
1.6327
1.4396
1.4243
0.9603
HN
N
N
S
N
O
Current Data Parameters
NAME 40bba-H
EXPNO 1
PROCNO 1
F2 - Acquisition Parameters
Date_ 20220114
Time 17.01
INSTRUM spect
PROBHD 5 mm TXI 1H-D/
PULPROG zg30
TD 65536
SOLVENT CDCl3
NS 16
DS 2
SWH 10000.000 Hz
FIDRES 0.152588 Hz
AQ 3.2767999 sec
RG 344.6
DW 50.000 usec
DE 6.50 usec
TE 285.2 K
D1 1.00000000 sec
TD0 1
======== CHANNEL f1 ========
SFO1 500.2330891 MHz
NUC1 1H
P1 7.00 usec
PLW1 13.00000000 W
F2 - Processing parameters
SI 65536
SF 500.2300168 MHz
WDW EM
SSB 0
LB 0.30 Hz
GB 0
PC 1.00
2.014
1.931
0.694
1.837
2.890
2.906
2.050
2.028
3.000
11
10
9
8
7
6
5
4
3
2
1
ppm

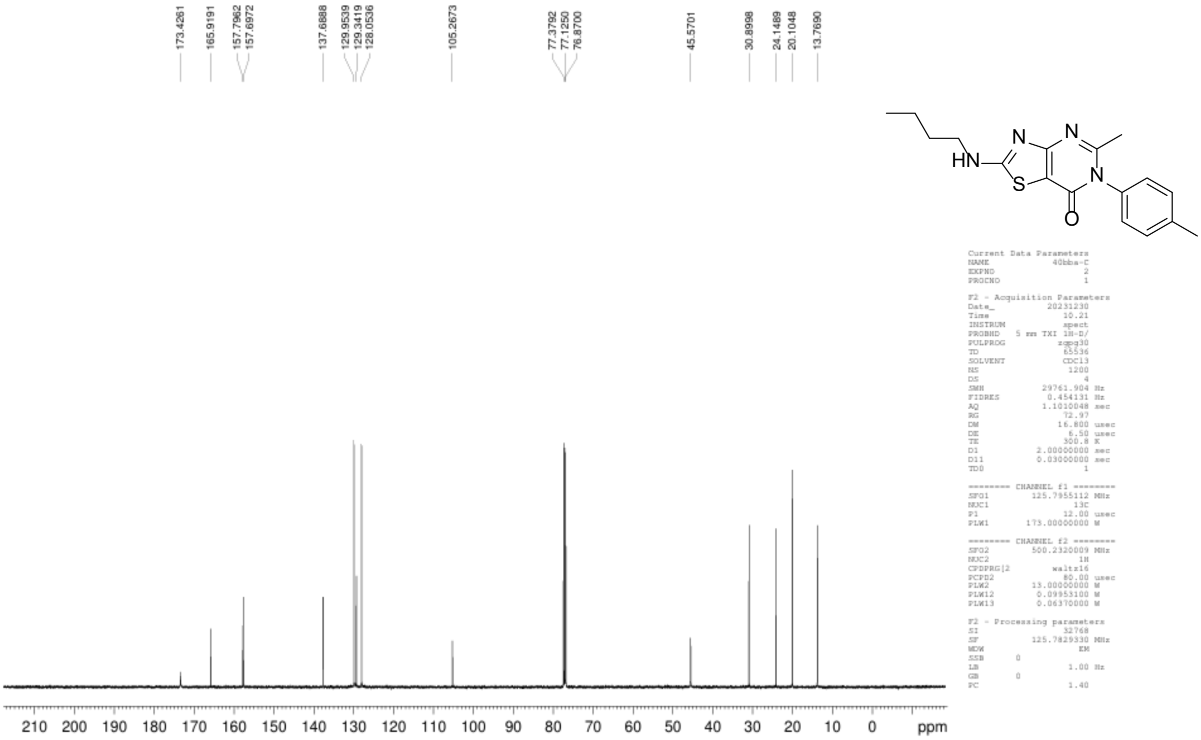
173.4261
165.9191
157.7962
157.6972
137.6888
129.9539
129.3419
128.0536
105.2673
77.3792
77.1250
76.8700
45.5701
30.8998
24.1489
20.1048
13.7690
HN
N
N
S
N
O
Current Data Parameters
NAME 40bba-C
EXPNO 2
PROCNO 1
F2 - Acquisition Parameters
Date_ 20231230
Time 10.21
INSTRUM spect
PROBHD 5 mm TXI 1H-D/
PULPROG zgpg30
TD 65536
SOLVENT CDCl3
NS 1200
DS 4
SWH 29761.904 Hz
FIDRES 0.454131 Hz
AQ 1.1010048 sec
RG 72.97
DW 16.800 usec
DE 6.50 usec
TE 300.8 K
D1 2.00000000 sec
D11 0.03000000 sec
TD0 1
======== CHANNEL f1 ========
SFO1 125.7955112 MHz
NUC1 13C
P1 12.00 usec
PLW1 173.00000000 W
======== CHANNEL f2 ========
SFO2 500.2320009 MHz
NUC2 1H
CPDPRG[2 waltz16
PCPD2 80.00 usec
PLW2 13.00000000 W
PLW12 0.09953100 W
PLW13 0.06370000 W
F2 - Processing parameters
SI 32768
SF 125.7829330 MHz
WDW EM
SSB 0
LB 1.00 Hz
GB 0
PC 1.40
210
200
190
180
170
160
150
140
130
120
110
100
90
80
70
60
50
40
30
20
10
0
ppm

1bbb

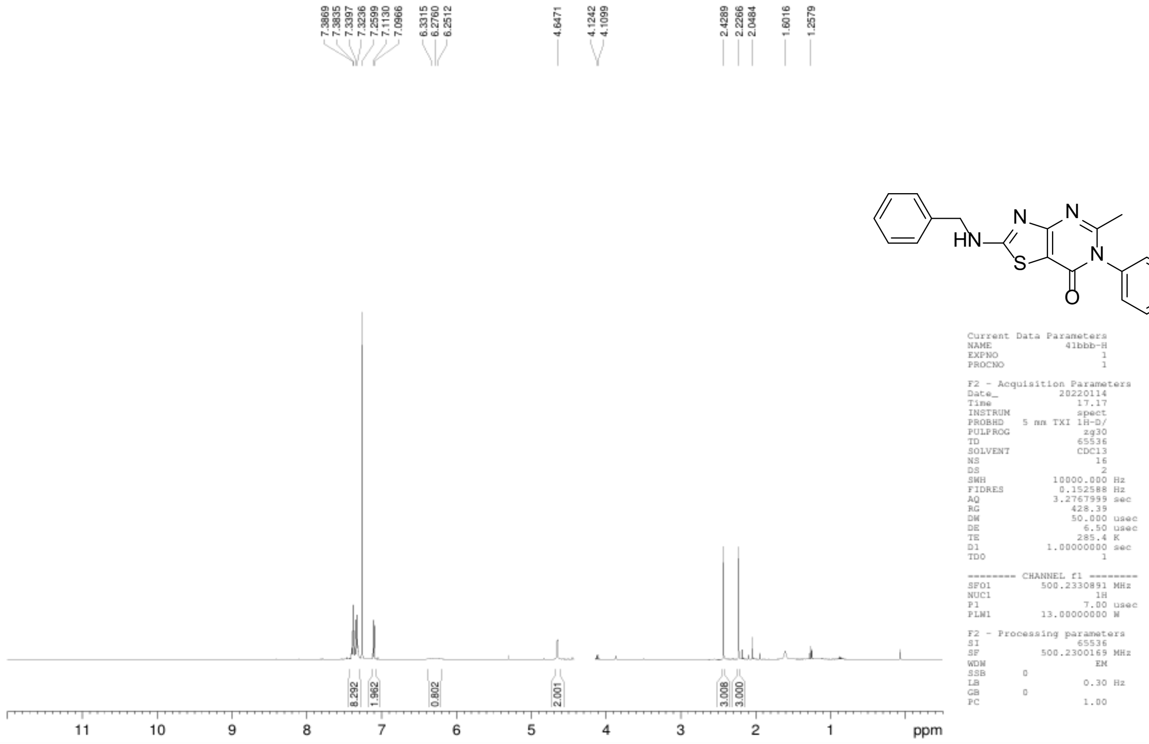
7.3869
7.3835
7.3397
7.3236
7.2599
7.1130
7.0966
6.3315
6.2760
6.2512
4.6471
4.1242
4.1099
2.4289
2.2266
2.0484
1.6016
1.2579
HN
N
N
S
O
Current Data Parameters
NAME 41bbb-H
EXPNO 1
PROCNO 1
F2 - Acquisition Parameters
Date_ 20220114
Time 17.17
INSTRUM spect
PROBHD 5 mm TXI 1H-D/
PULPROG zg30
TD 65536
SOLVENT CDCl3
NS 16
DS 2
SWH 10000.000 Hz
FIDRES 0.152588 Hz
AQ 3.2767999 sec
RG 428.39
DW 50.000 usec
DE 6.50 usec
TE 285.4 K
D1 1.00000000 sec
TD0 1
======== CHANNEL f1 ========
SFO1 500.2330891 MHz
NUC1 1H
P1 7.00 usec
PLW1 13.00000000 W
F2 - Processing parameters
SI 65536
SF 500.2300169 MHz
WDW EM
SSB 0
LB 0.30 Hz
GB 0
PC 1.00
8.292
1.962
0.802
2.001
3.008
3.000
11
10
9
8
7
6
5
4
3
2
1
ppm

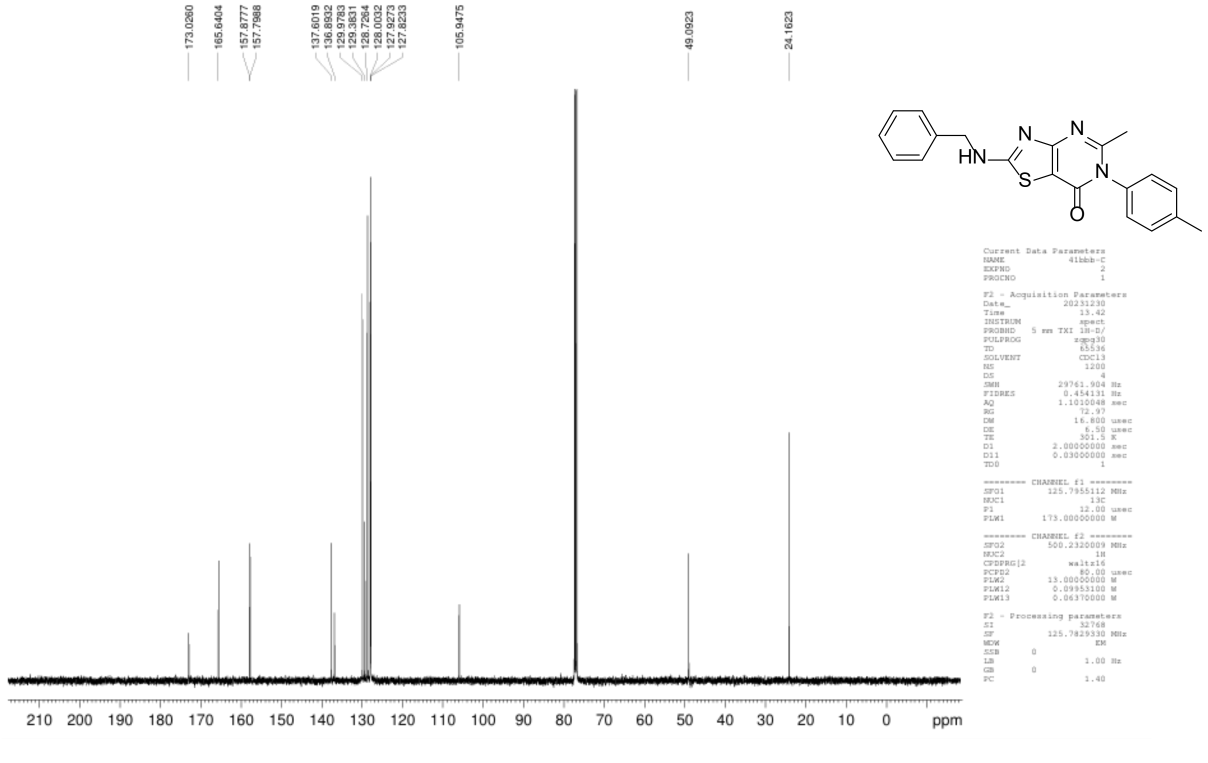
173.0260
165.6404
157.8777
157.7988
137.6019
136.8932
129.9783
129.3831
128.7264
128.0032
127.9273
127.8233
105.9475
49.0923
24.1623
HN
N
N
S
O
Current Data Parameters
NAME 41bbb-C
EXPNO 2
PROCNO 1
F2 - Acquisition Parameters
Date_ 20231230
Time 13.42
INSTRUM spect
PROBHD 5 mm TXI 1H-D/
PULPROG zgpg30
TD 65536
SOLVENT CDCl3
NS 1200
DS 4
SWH 29761.904 Hz
FIDRES 0.454131 Hz
AQ 1.1010048 sec
RG 72.97
DW 16.800 usec
DE 6.50 usec
TE 301.5 K
D1 2.00000000 sec
D11 0.03000000 sec
TD0 1
======== CHANNEL f1 ========
SFO1 125.7955112 MHz
NUC1 13C
P1 12.00 usec
PLW1 173.00000000 W
======== CHANNEL f2 ========
SFO2 500.2320009 MHz
NUC2 1H
CPDPRG[2 waltz16
PCPD2 80.00 usec
PLW2 13.00000000 W
PLW12 0.09953100 W
PLW13 0.06370000 W
F2 - Processing parameters
SI 32768
SF 125.7829330 MHz
WDW EM
SSB 0
LB 1.00 Hz
GB 0
PC 1.40
210
200
190
180
170
160
150
140
130
120
110
100
90
80
70
60
50
40
30
20
10
0
ppm

1bbc

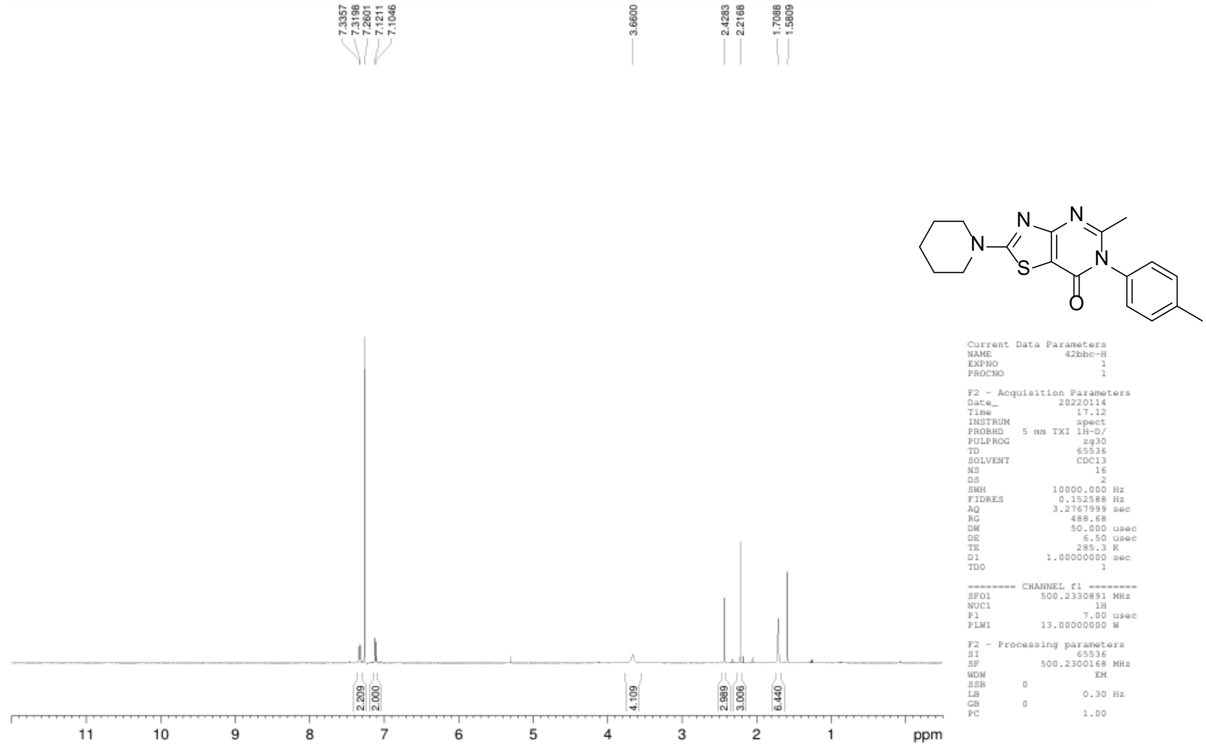
7.3357
7.3198
7.2601
7.1211
7.1046
3.6600
2.4283
2.2168
1.7088
1.5809
2.209
2.000
4.109
2.989
3.006
6.440
ppm
N
N
N
S
N
O

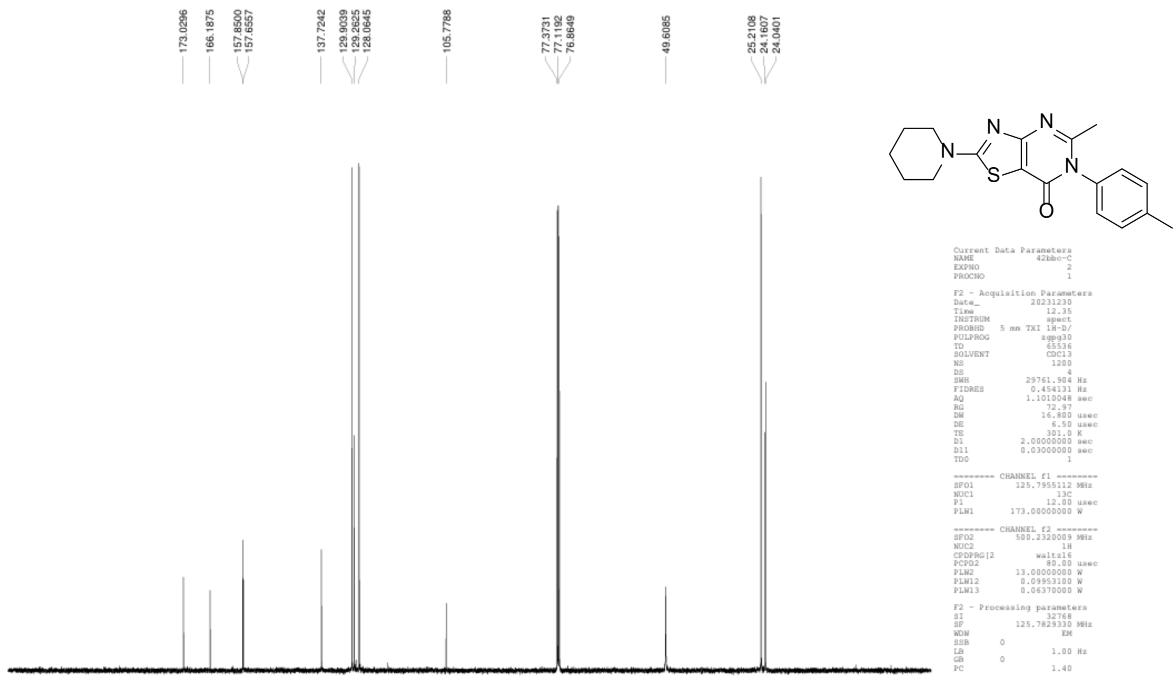
173.0296
166.1875
157.8500
157.6557
137.7242
129.9039
129.2625
128.0645
105.7788
77.3731
77.1192
76.8649
49.6085
25.2108
24.1607
24.0401
ppm
N
N
N
S
N
O

1bbe

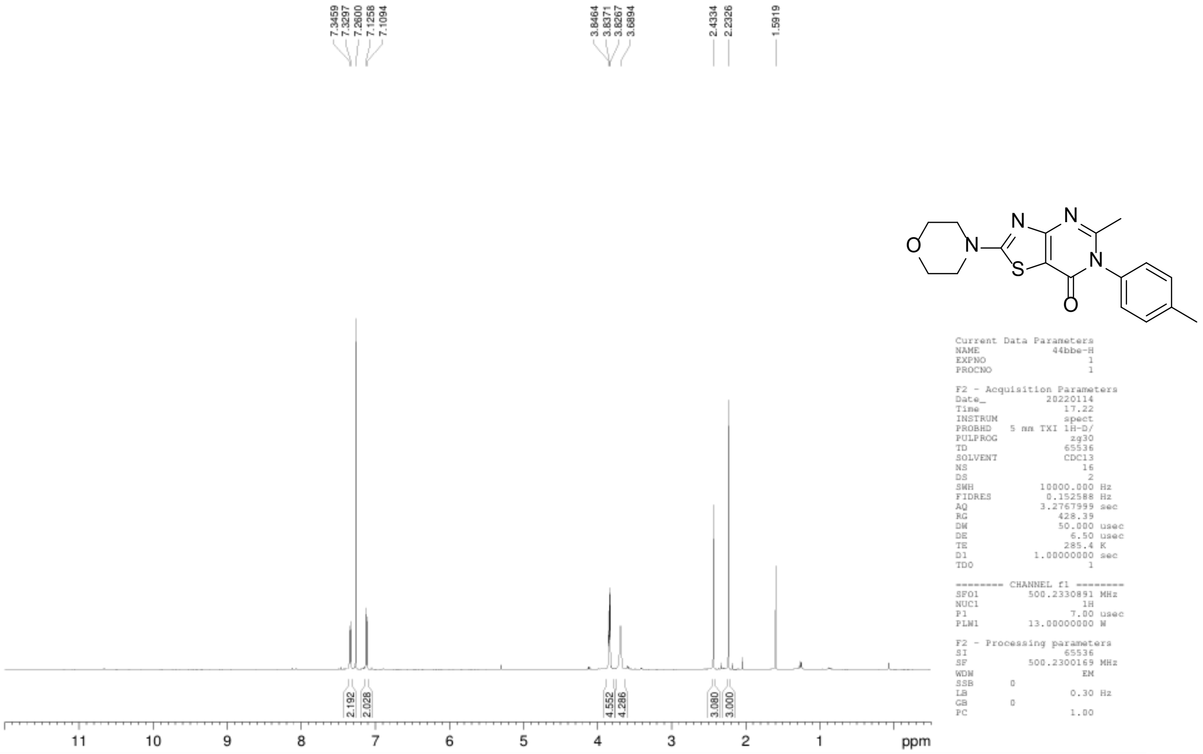
7.3459
7.3297
7.2600
7.1258
7.1094
3.8464
3.8371
3.8267
3.6894
2.4334
2.2326
1.5919
2.192
2.028
4.552
4.286
3.080
3.000
11 10 9 8 7 6 5 4 3 2 1 ppm
N
N
O
N
S
N
O
Current Data Parameters
NAME 44bbe-H
EXPNO 1
PROCNO 1
F2 - Acquisition Parameters
Date_ 20220114
Time 17.22
INSTRUM spect
PROBHD 5 mm TXI 1H-D/
PULPROG zg30
TD 65536
SOLVENT CDCl3
NS 16
DS 2
SWH 10000.000 Hz
FIDRES 0.152588 Hz
AQ 3.2767999 sec
RG 428.39
DW 50.000 usec
DE 6.50 usec
TE 285.4 K
D1 1.00000000 sec
TD0 1
======== CHANNEL f1 ========
SFO1 500.2330891 MHz
NUC1 1H
P1 7.00 usec
PLW1 13.00000000 W
F2 - Processing parameters
SI 65536
SF 500.2300169 MHz
WDW EM
SSB 0
LB 0.30 Hz
GB 0
PC 1.00

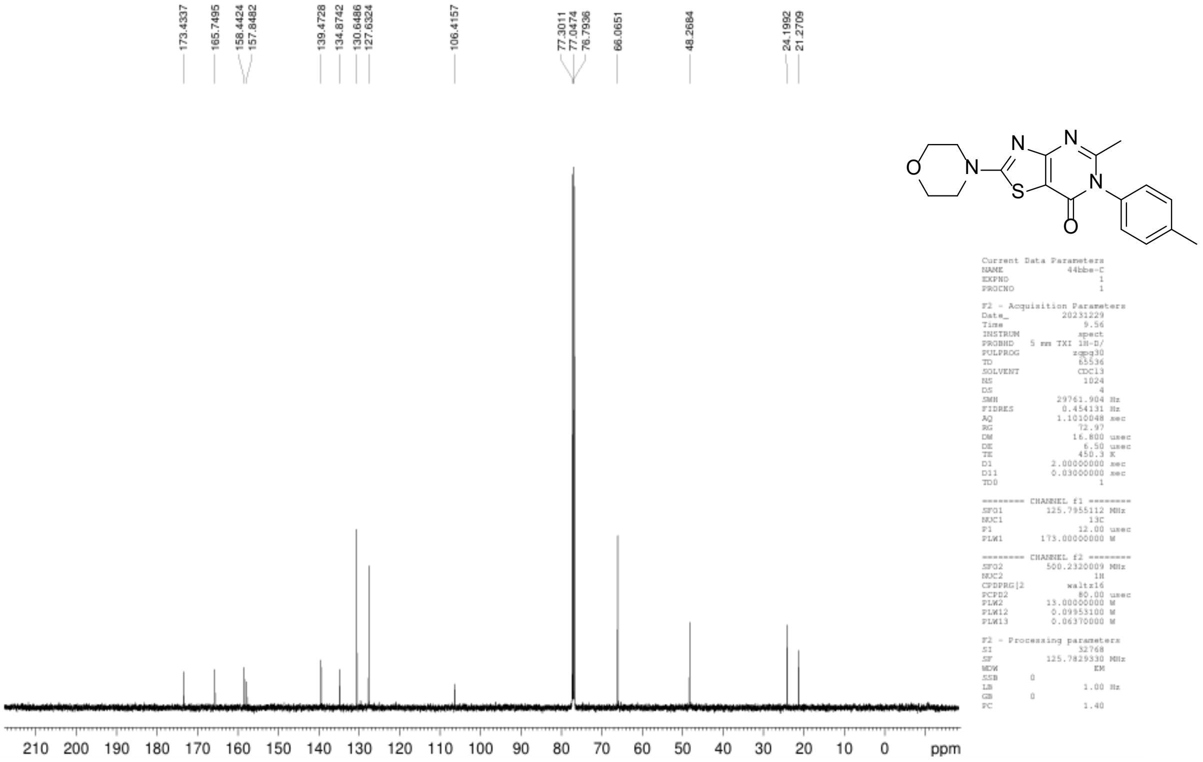
173.4337
165.7495
158.4424
157.8482
139.4728
134.8742
130.6486
127.6324
106.4157
77.3011
77.0474
76.7936
66.0651
48.2684
24.1992
21.2709
210 200 190 180 170 160 150 140 130 120 110 100 90 80 70 60 50 40 30 20 10 0 ppm
N
N
O
N
S
N
O
Current Data Parameters
NAME 44bbe-C
EXPNO 1
PROCNO 1
F2 - Acquisition Parameters
Date_ 20231229
Time 9.56
INSTRUM spect
PROBHD 5 mm TXI 1H-D/
PULPROG zgpg30
TD 65536
SOLVENT CDCl3
NS 1024
DS 4
SWH 29761.904 Hz
FIDRES 0.454131 Hz
AQ 1.1010048 sec
RG 72.97
DW 16.800 usec
DE 6.50 usec
TE 450.3 K
D1 2.00000000 sec
D11 0.03000000 sec
TD0 1
======== CHANNEL f1 ========
SFO1 125.7955112 MHz
NUC1 13C
P1 12.00 usec
PLW1 173.00000000 W
======== CHANNEL f2 ========
SFO2 500.2320009 MHz
NUC2 1H
CPDPRG[2 waltz16
PCPD2 80.00 usec
PLW2 13.00000000 W
PLW12 0.09953100 W
PLW13 0.06370000 W
F2 - Processing parameters
SI 32768
SF 125.7829330 MHz
WDW EM
SSB 0
LB 1.00 Hz
GB 0
PC 1.40

1bbg

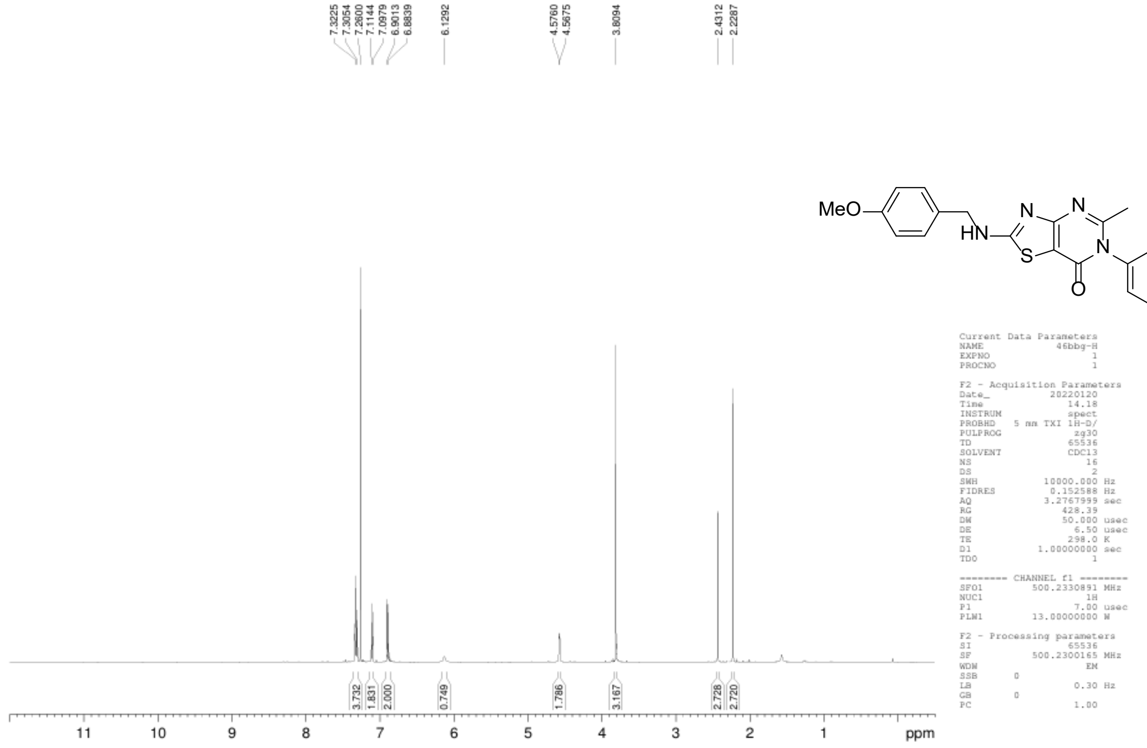

7.3225
7.3054
7.2600
7.1144
7.0979
6.9013
6.8839
6.1292
4.5760
4.5675
3.8094
2.4312
2.2287
MeO
HN
N
S
N
N
O
3.732
1.831
2.000
0.749
1.786
3.167
2.728
2.720
11 10 9 8 7 6 5 4 3 2 1 ppm
Current Data Parameters
NAME 46bbg-H
EXPNO 1
PROCNO 1
F2 - Acquisition Parameters
Date_ 20220120
Time 14.18
INSTRUM spect
PROBHD 5 mm TXI 1H-D/
PULPROG zg30
TD 65536
SOLVENT CDCl3
NS 16
DS 2
SWH 10000.000 Hz
FIDRES 0.152588 Hz
AQ 3.2767999 sec
RG 428.39
DW 50.000 usec
DE 6.50 usec
TE 298.0 K
D1 1.00000000 sec
TD0 1
======== CHANNEL f1 ========
SFO1 500.2330891 MHz
NUC1 1H
P1 7.00 usec
PLW1 13.00000000 W
F2 - Processing parameters
SI 65536
SF 500.2300165 MHz
WDW EM
SSB 0
LB 0.30 Hz
GB 0
PC 1.00

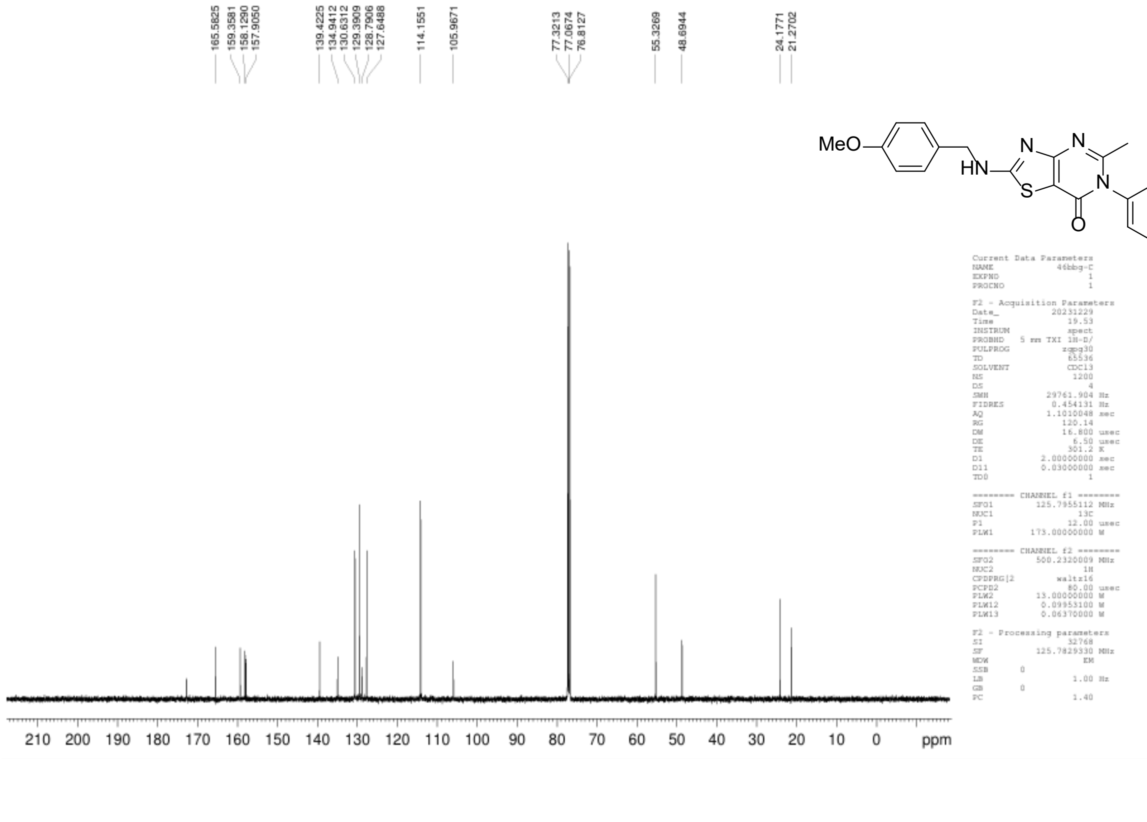

165.5825
159.3581
158.1290
157.9050
139.4225
134.9412
130.6312
129.3909
128.7906
127.6488
114.1551
105.9671
77.3213
77.0674
76.8127
55.3269
48.6944
24.1771
21.2702
MeO
HN
N
S
N
N
O
210 200 190 180 170 160 150 140 130 120 110 100 90 80 70 60 50 40 30 20 10 0 ppm
Current Data Parameters
NAME 46bbg-C
EXPNO 1
PROCNO 1
F2 - Acquisition Parameters
Date_ 20231229
Time 19.53
INSTRUM spect
PROBHD 5 mm TXI 1H-D/
PULPROG zgpg30
TD 65536
SOLVENT CDCl3
NS 1200
DS 4
SWH 29761.904 Hz
FIDRES 0.454131 Hz
AQ 1.1010048 sec
RG 120.14
DW 16.800 usec
DE 6.50 usec
TE 301.2 K
D1 2.00000000 sec
D11 0.03000000 sec
TD0 1
======== CHANNEL f1 ========
SFO1 125.7955112 MHz
NUC1 13C
P1 12.00 usec
PLW1 173.00000000 W
======== CHANNEL f2 ========
SFO2 500.2320009 MHz
NUC2 1H
CPDPRG[2 waltz16
PCPD2 80.00 usec
PLW2 13.00000000 W
PLW12 0.09953100 W
PLW13 0.06370000 W
F2 - Processing parameters
SI 32768
SF 125.7829330 MHz
WDW EM
SSB 0
LB 1.00 Hz
GB 0
PC 1.40

1bbh

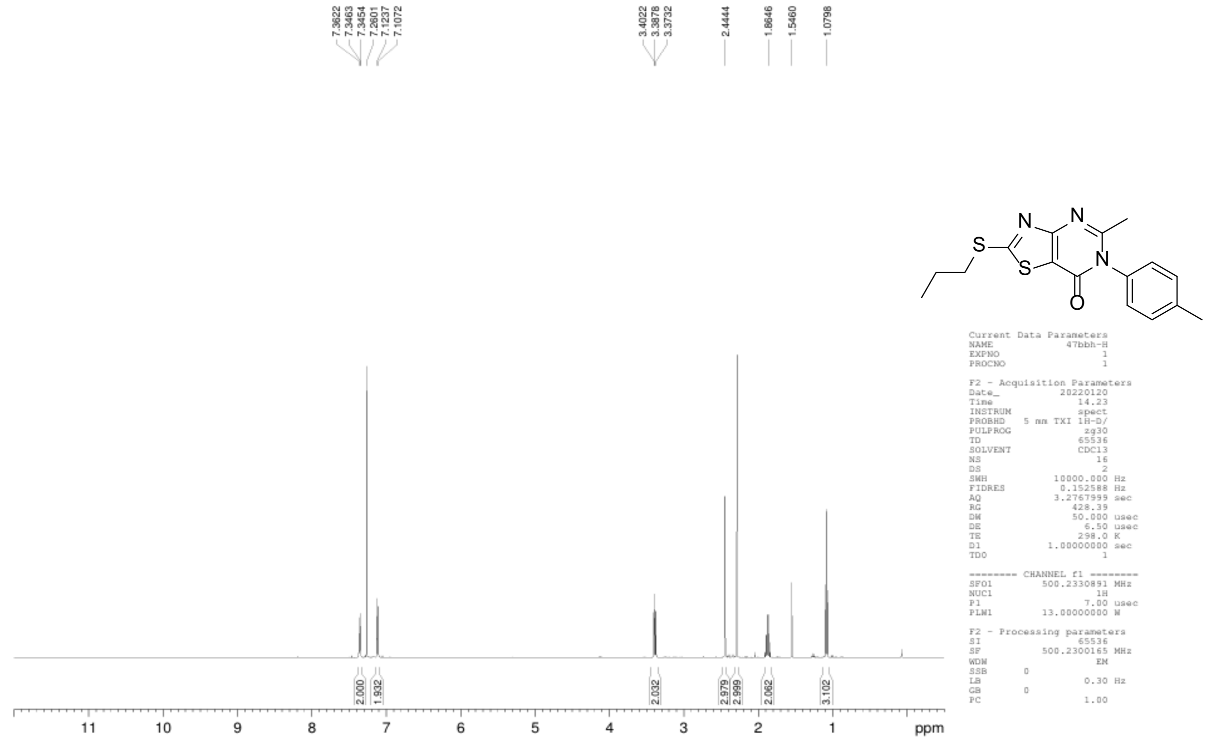
7.3622
7.3463
7.3454
7.2601
7.1237
7.1072
3.4022
3.3878
3.3732
2.4444
1.8646
1.5460
1.0798
2.000
1.932
2.032
2.979
2.999
2.062
3.102
ppm

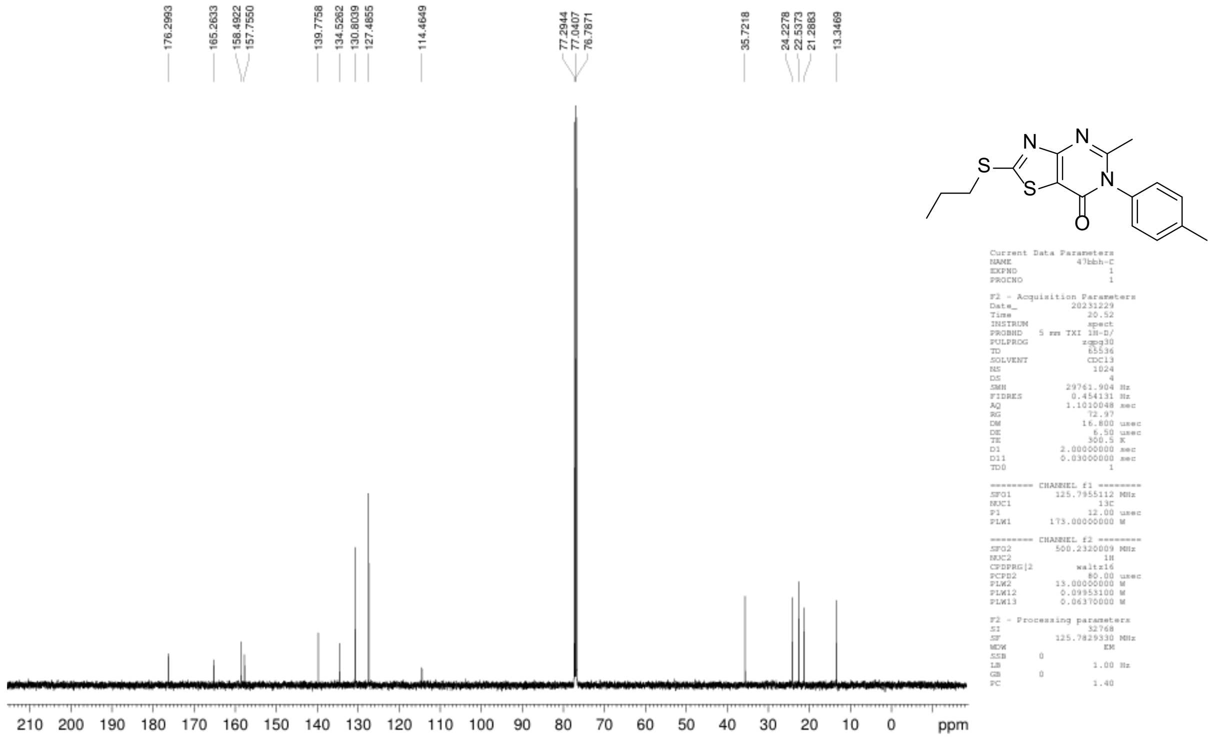
176.2993
165.2633
158.4922
157.7550
139.7758
134.5262
130.8039
127.4855
114.4649
77.2944
77.0407
76.7871
35.7218
24.2278
22.5373
21.2883
13.3469
ppm

1bbj

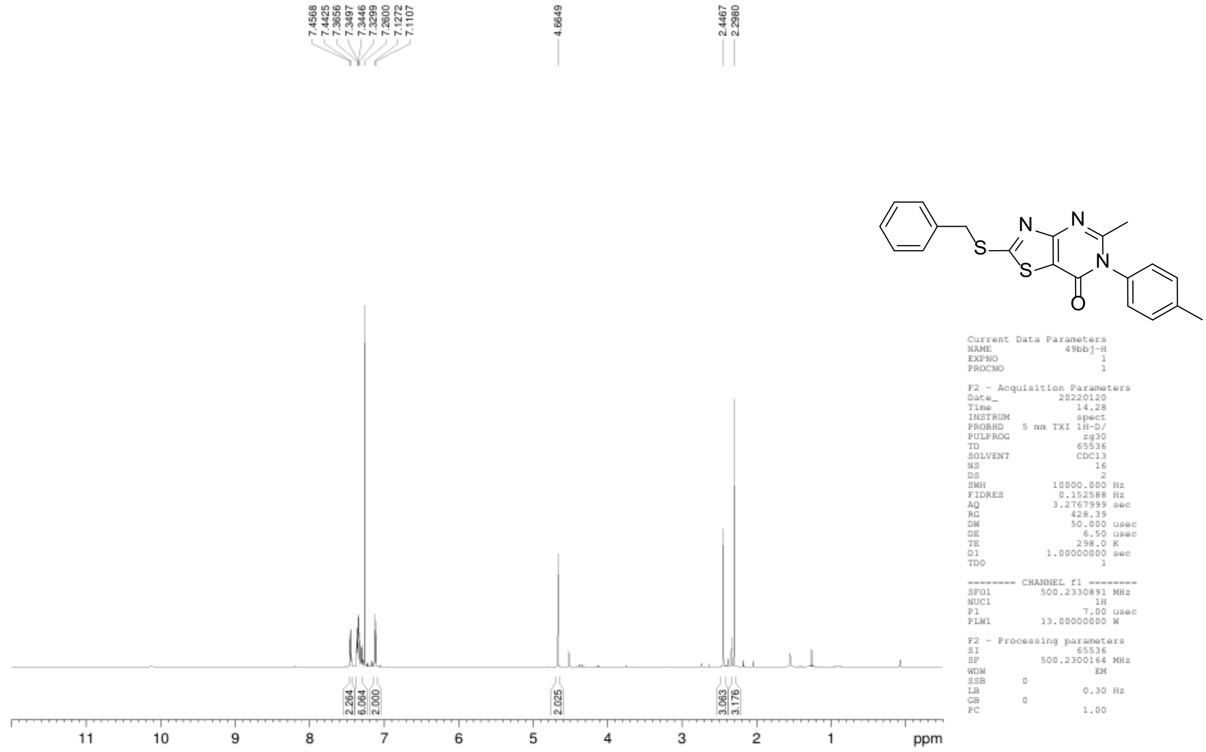
7.4568
7.4425
7.3656
7.3497
7.3446
7.3299
7.2600
7.1272
7.1107
4.6649
2.4467
2.2980
N
N
S
S
N
O
2.264
6.064
2.000
2.025
3.063
3.176
11
10
9
8
7
6
5
4
3
2
1
ppm

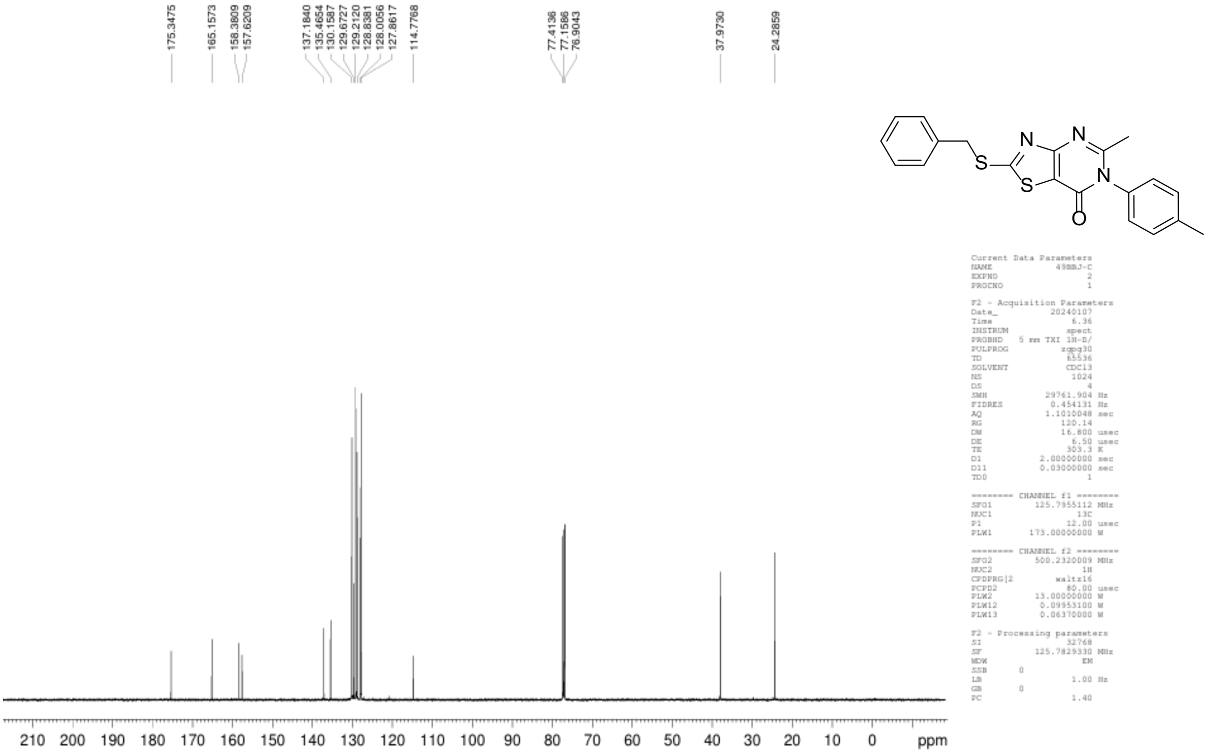
175.3475
165.1573
158.3809
157.6209
137.1840
135.4654
130.1587
129.6727
129.2120
128.8381
128.0056
127.8617
114.7768
77.4136
77.1586
76.9043
37.9730
24.2859
N
N
S
S
N
O
210
200
190
180
170
160
150
140
130
120
110
100
90
80
70
60
50
40
30
20
10
0
ppm

1daa

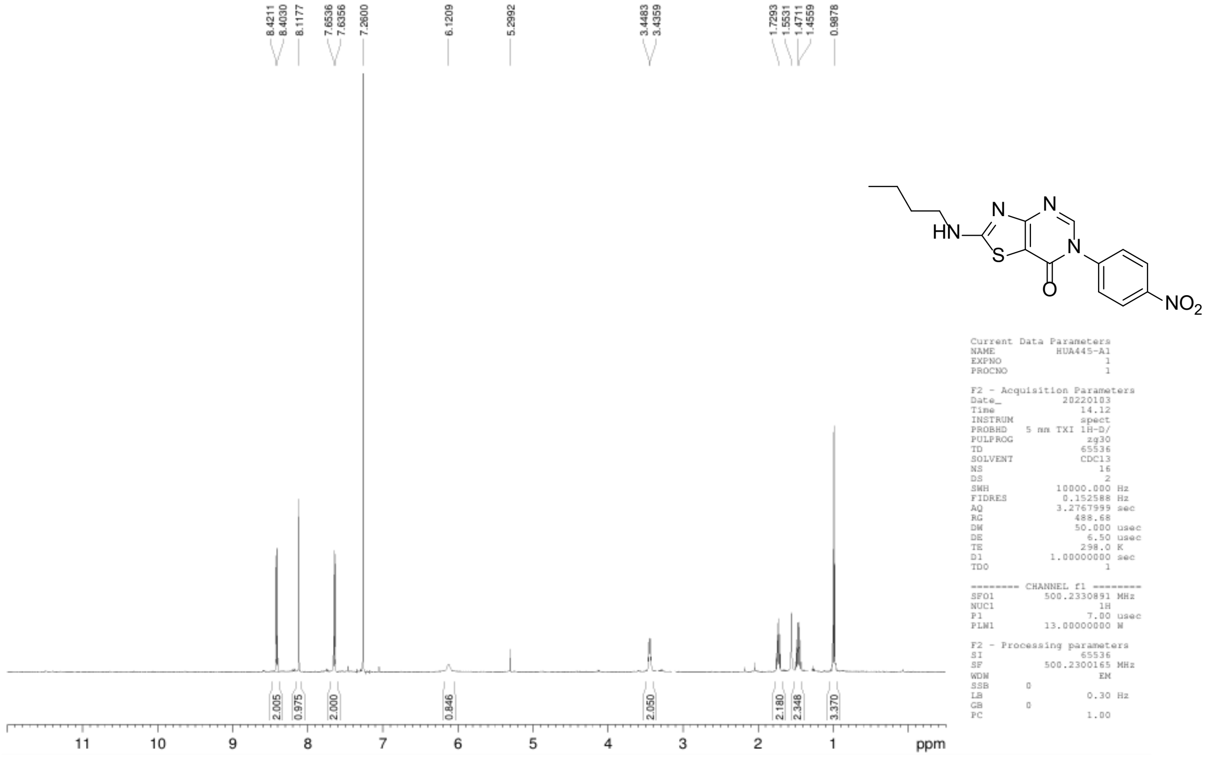
8.4211
8.4030
8.1177
7.6536
7.6356
7.2600
6.1209
5.2992
3.4483
3.4359
1.7293
1.5531
1.4711
1.4559
0.9878
N
N
HN
S
N
O
NO2
Current Data Parameters
NAME HUA445-A1
EXPNO 1
PROCNO 1
F2 - Acquisition Parameters
Date_ 20220103
Time 14.12
INSTRUM spect
PROBHD 5 mm TXI 1H-D/
PULPROG zg30
TD 65536
SOLVENT CDCl3
NS 16
DS 2
SWH 10000.000 Hz
FIDRES 0.152588 Hz
AQ 3.2767999 sec
RG 488.68
DW 50.000 usec
DE 6.50 usec
TE 298.0 K
D1 1.00000000 sec
TD0 1
======== CHANNEL f1 ========
SFO1 500.2330891 MHz
NUC1 1H
P1 7.00 usec
PLW1 13.00000000 W
F2 - Processing parameters
SI 65536
SF 500.2300165 MHz
WDW EM
SSB 0
LB 0.30 Hz
GB 0
PC 1.00
2.005
0.975
2.000
0.846
2.050
2.180
2.348
3.370
11
10
9
8
7
6
5
4
3
2
1
ppm

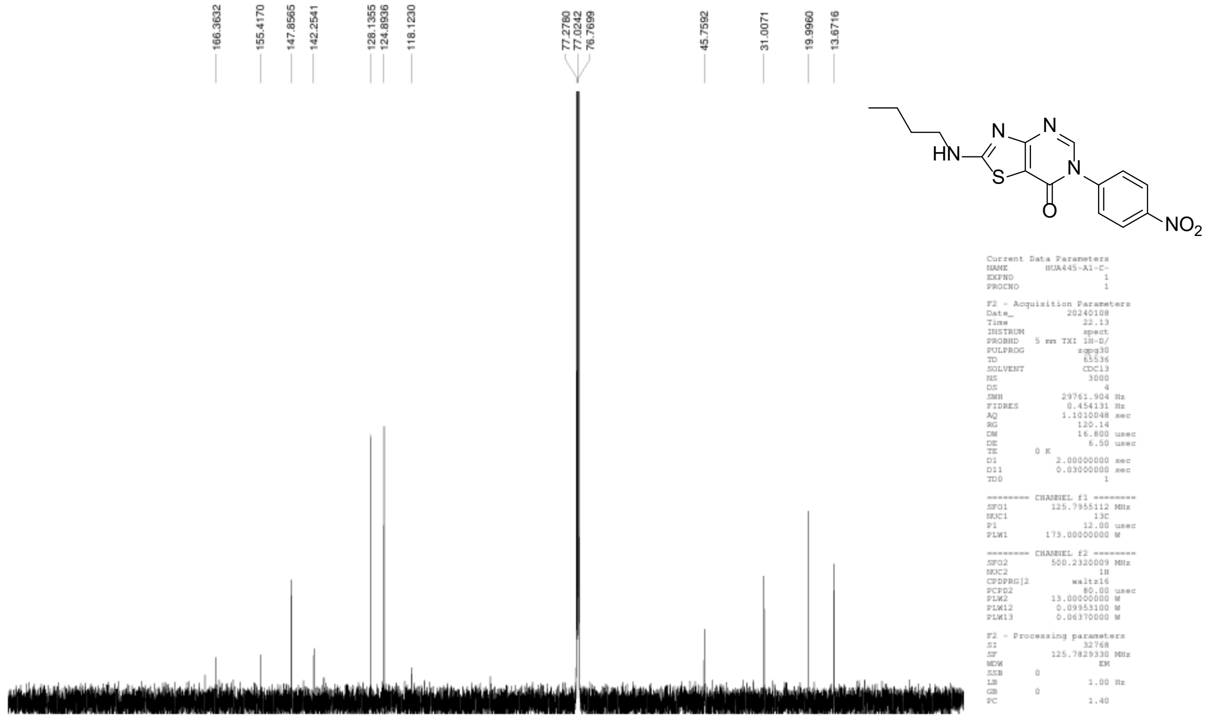
166.3632
155.4170
147.8565
142.2541
128.1355
124.8936
118.1230
77.2780
77.0242
76.7699
45.7592
31.0071
19.9960
13.6716
N
N
HN
S
N
O
NO2
Current Data Parameters
NAME HUA445-A1-C-
EXPNO 1
PROCNO 1
F2 - Acquisition Parameters
Date_ 20240108
Time 22.13
INSTRUM spect
PROBHD 5 mm TXI 1H-D/
PULPROG zgpg30
TD 65536
SOLVENT CDCl3
NS 3000
DS 4
SWH 29761.904 Hz
FIDRES 0.454131 Hz
AQ 1.1010048 sec
RG 120.14
DW 16.800 usec
DE 6.50 usec
TE 0 K
D1 2.00000000 sec
D11 0.03000000 sec
TD0 1
======== CHANNEL f1 ========
SFO1 125.7955112 MHz
NUC1 13C
P1 12.00 usec
PLW1 173.00000000 W
======== CHANNEL f2 ========
SFO2 500.2320009 MHz
NUC2 1H
CPDPRG[2 waltz16
PCPD2 80.00 usec
PLW2 13.00000000 W
PLW12 0.09953100 W
PLW13 0.06370000 W
F2 - Processing parameters
SI 32768
SF 125.7829330 MHz
WDW EM
SSB 0
LB 1.00 Hz
GB 0
PC 1.40
210
200
190
180
170
160
150
140
130
120
110
100
90
80
70
60
50
40
30
20
10
0
ppm

1dac

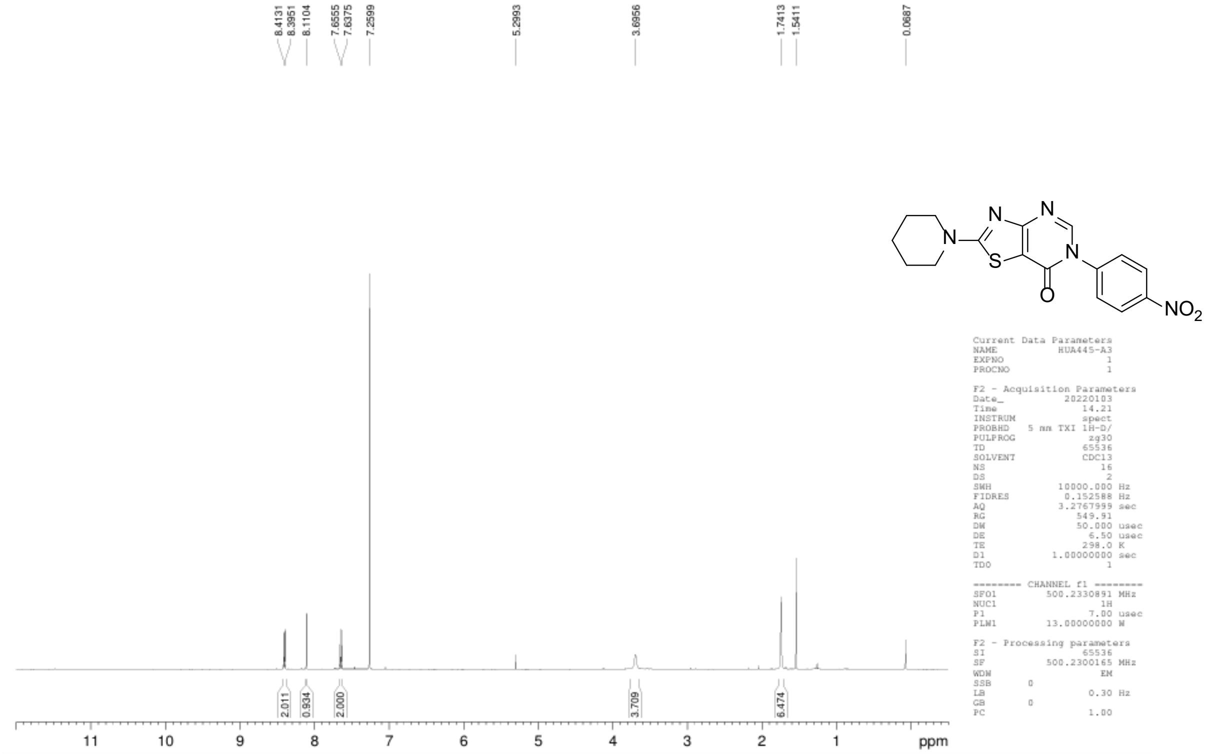
8.4131
8.3951
8.1104
7.6555
7.6375
7.2599
5.2993
3.6956
1.7413
1.5411
0.0687
N
N
N
N
S
O
NO2
2.011
0.934
2.000
3.709
6.474
11 10 9 8 7 6 5 4 3 2 1 ppm

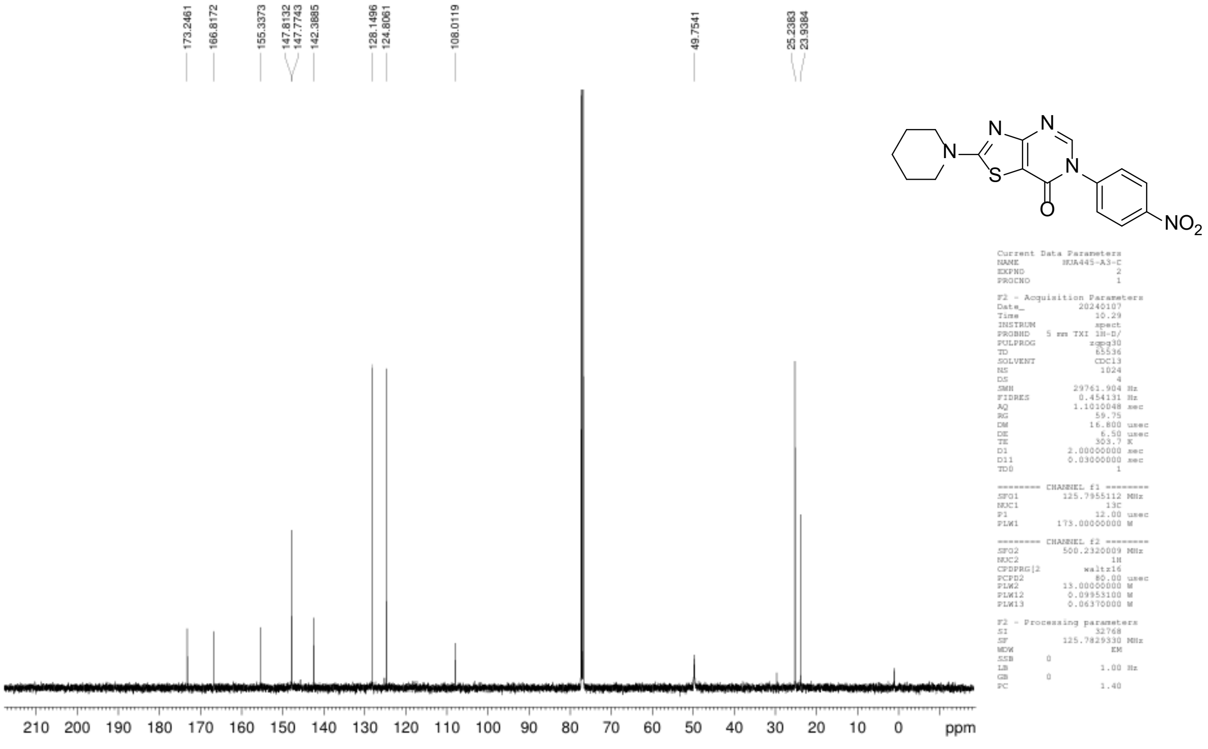
173.2461
166.8172
155.3373
147.8132
147.7743
142.3885
128.1496
124.8061
108.0119
49.7541
25.2383
23.9384
N
N
N
N
S
O
NO2
210 200 190 180 170 160 150 140 130 120 110 100 90 80 70 60 50 40 30 20 10 0 ppm

1dae

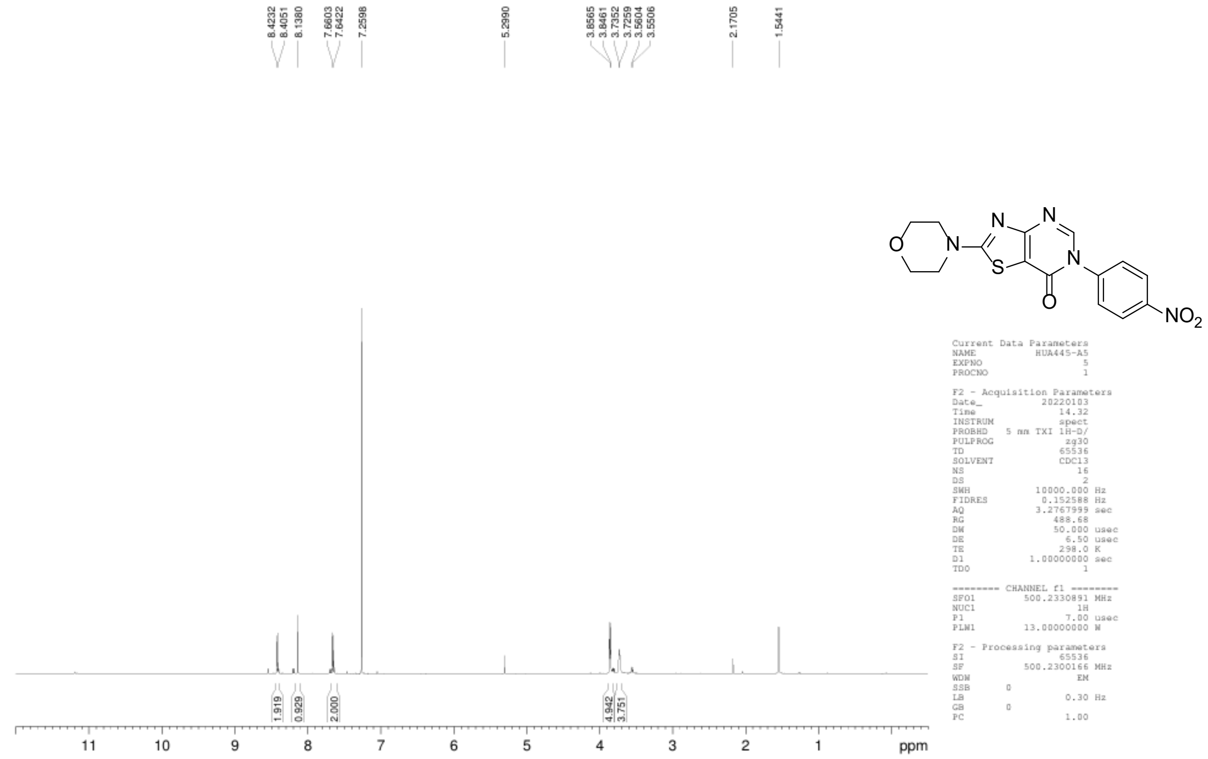
8.4232
8.4051
8.1380
7.6603
7.6422
7.2598
5.2990
3.8565
3.8461
3.7352
3.7259
3.5604
3.5506
2.1705
1.5441
1.919
0.929
2.000
4.942
3.751
11 10 9 8 7 6 5 4 3 2 1 ppm
O
N
S
N
N
N
O
NO2
Current Data Parameters
NAME HUA445-A5
EXPNO 5
PROCNO 1
F2 - Acquisition Parameters
Date_ 20220103
Time 14.32
INSTRUM spect
PROBHD 5 mm TXI 1H-D/
PULPROG zg30
TD 65536
SOLVENT CDCl3
NS 16
DS 2
SWH 10000.000 Hz
FIDRES 0.152588 Hz
AQ 3.2767999 sec
RG 488.68
DW 50.000 usec
DE 6.50 usec
TE 298.0 K
D1 1.00000000 sec
TD0 1
======== CHANNEL f1 ========
SFO1 500.2330891 MHz
NUC1 1H
P1 7.00 usec
PLW1 13.00000000 W
F2 - Processing parameters
SI 65536
SF 500.2300166 MHz
WDW EM
SSB 0
LB 0.30 Hz
GB 0
PC 1.00

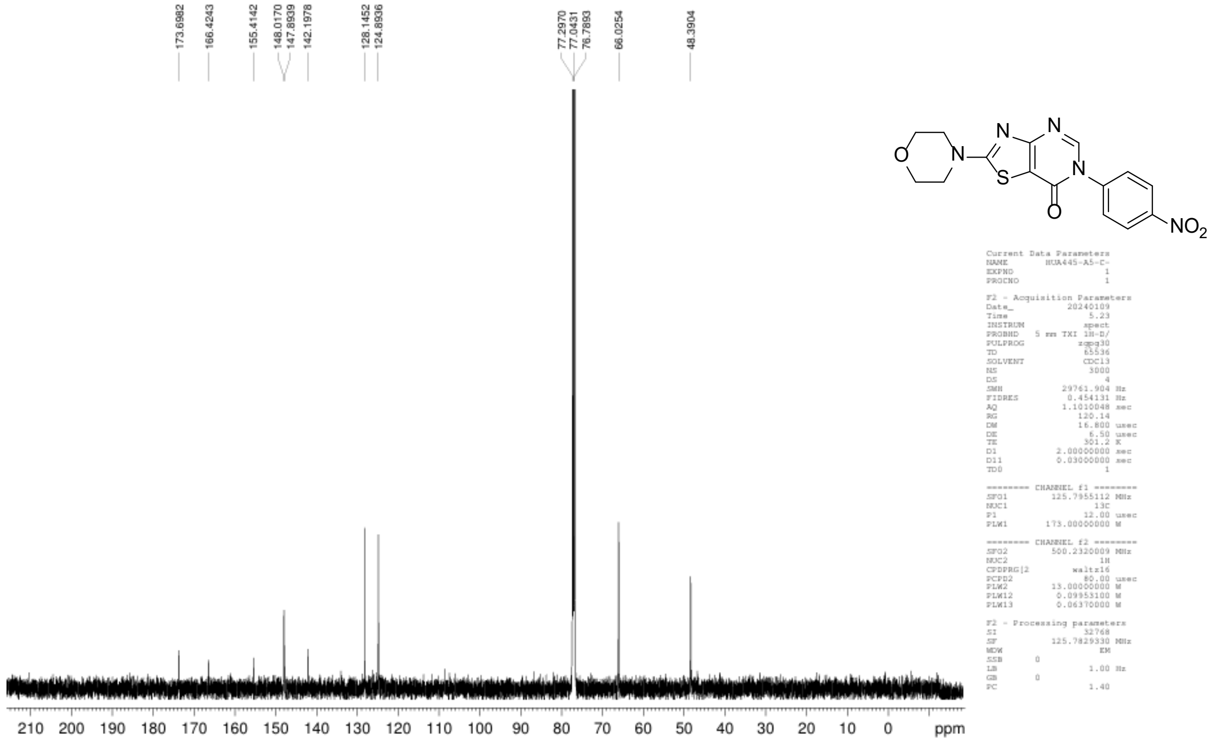
173.6982
166.4243
155.4142
148.0170
147.8939
142.1978
128.1452
124.8936
77.2970
77.0431
76.7893
66.0254
48.3904
210 200 190 180 170 160 150 140 130 120 110 100 90 80 70 60 50 40 30 20 10 0 ppm
O
N
S
N
N
N
O
NO2
Current Data Parameters
NAME HUA445-A5-C-
EXPNO 1
PROCNO 1
F2 - Acquisition Parameters
Date_ 20240109
Time 5.23
INSTRUM spect
PROBHD 5 mm TXI 1H-D/
PULPROG zgpg30
TD 65536
SOLVENT CDCl3
NS 3000
DS 4
SWH 29761.904 Hz
FIDRES 0.454131 Hz
AQ 1.1010048 sec
RG 120.14
DW 16.800 usec
DE 6.50 usec
TE 301.2 K
D1 2.00000000 sec
D11 0.03000000 sec
TD0 1
======== CHANNEL f1 ========
SFO1 125.7955112 MHz
NUC1 13C
P1 12.00 usec
PLW1 173.00000000 W
======== CHANNEL f2 ========
SFO2 500.2320009 MHz
NUC2 1H
CPDPRG[2 waltz16
PCPD2 80.00 usec
PLW2 13.00000000 W
PLW12 0.09953100 W
PLW13 0.06370000 W
F2 - Processing parameters
SI 32768
SF 125.7829330 MHz
WDW EM
SSB 0
LB 1.00 Hz
GB 0
PC 1.40

1daf

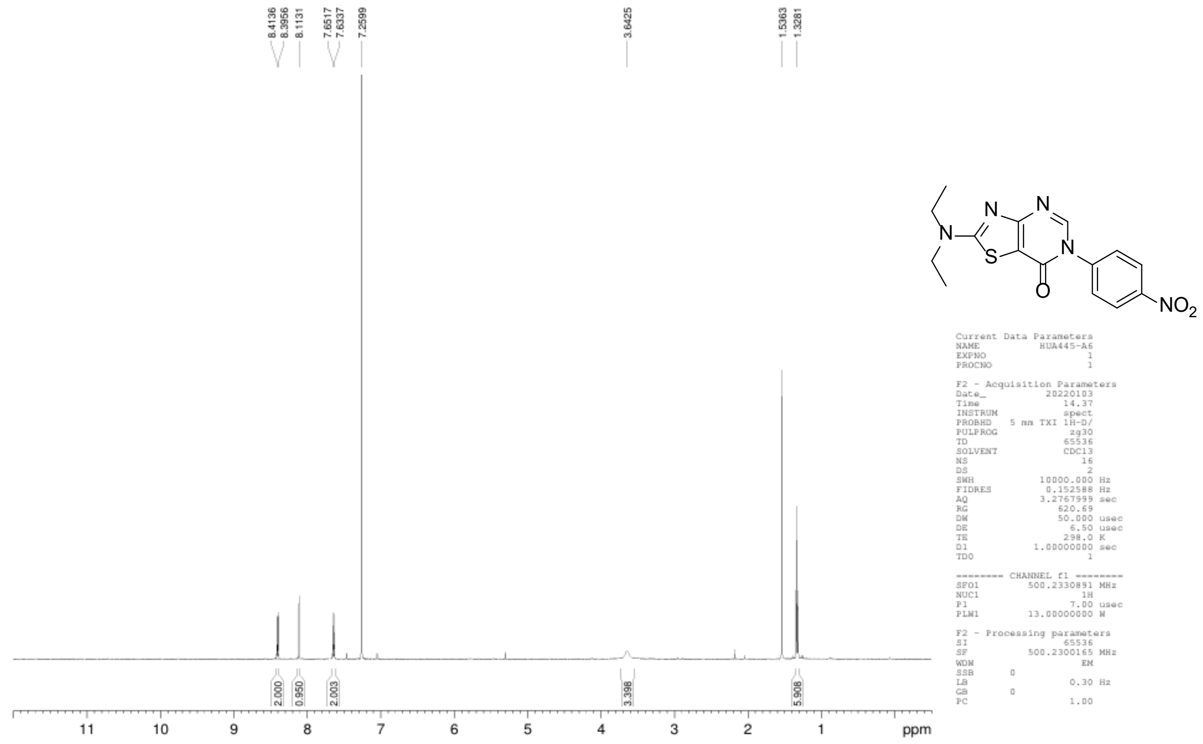

8.4136
8.3956
8.1131
7.6517
7.6337
7.2599
3.6425
1.5363
1.3281
2.000
0.950
2.003
3.398
5.908
11 10 9 8 7 6 5 4 3 2 1 ppm
N
N
N
N
S
O
NO_2
Current Data Parameters
NAME HUA445-A6
EXPNO 1
PROCNO 1
F2 - Acquisition Parameters
Date_ 20220103
Time 14.37
INSTRUM spect
PROBHD 5 mm TXI 1H-D/
PULPROG zg30
TD 65536
SOLVENT CDCl3
NS 16
DS 2
SWH 10000.000 Hz
FIDRES 0.152588 Hz
AQ 3.2767999 sec
RG 620.69
DW 50.000 usec
DE 6.50 usec
TE 298.0 K
D1 1.00000000 sec
TD0 1
======== CHANNEL f1 ========
SFO1 500.2330891 MHz
NUC1 1H
P1 7.00 usec
PLW1 13.00000000 W
F2 - Processing parameters
SI 65536
SF 500.2300165 MHz
WDW EM
SSB 0
LB 0.30 Hz
GB 0
PC 1.00

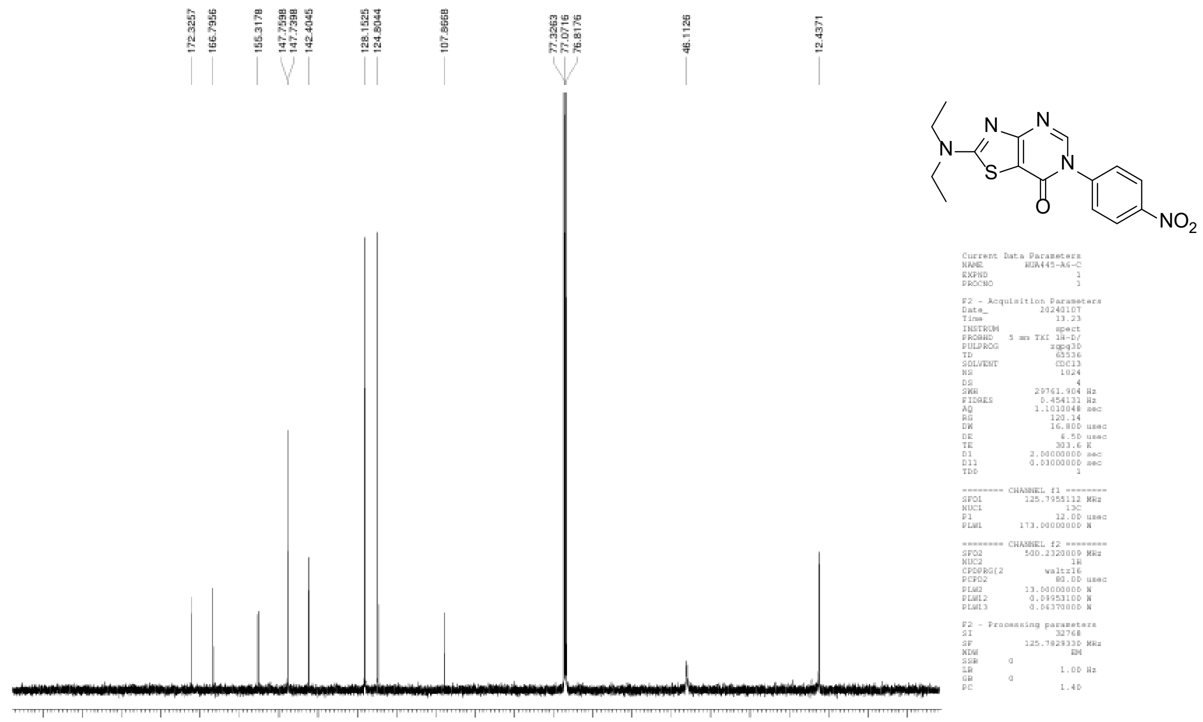

172.3257
166.7956
155.3178
147.7598
147.7398
142.4045
128.1525
124.8044
107.8668
77.3263
77.0716
76.8176
46.1126
12.4371
210 200 190 180 170 160 150 140 130 120 110 100 90 80 70 60 50 40 30 20 10 0 ppm
N
N
N
N
S
O
NO_2
Current Data Parameters
NAME HUA445-A6-C
EXPNO 1
PROCNO 1
F2 - Acquisition Parameters
Date_ 20240107
Time 13.23
INSTRUM spect
PROBHD 5 mm TXI 1H-D/
PULPROG zgpg30
TD 65536
SOLVENT CDCl3
NS 1024
DS 4
SWH 29761.904 Hz
FIDRES 0.454131 Hz
AQ 1.1010048 sec
RG 120.14
DW 16.800 usec
DE 6.50 usec
TE 303.6 K
D1 2.00000000 sec
D11 0.03000000 sec
TD0 1
======== CHANNEL f1 ========
SFO1 125.7955112 MHz
NUC1 13C
P1 12.00 usec
PLW1 173.00000000 W
======== CHANNEL f2 ========
SFO2 500.2320009 MHz
NUC2 1H
CPDPRG[2 waltz16
PCPD2 80.00 usec
PLW2 13.00000000 W
PLW12 0.04953100 W
PLW13 0.04370000 W
F2 - Processing parameters
SI 32768
SF 125.7828330 MHz
WDW EM
SSB 0
LB 1.00 Hz
GB 0
PC 1.40

1dah

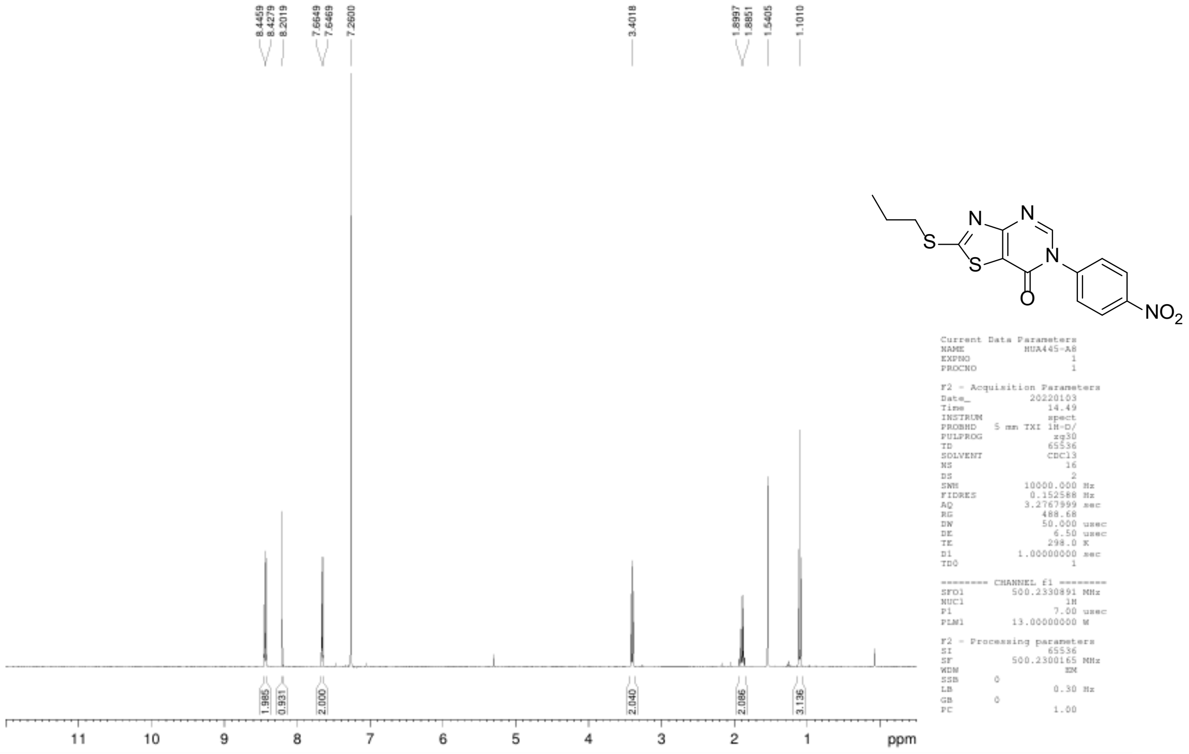
8.4459
8.4279
8.2019
7.6649
7.6469
7.2600
3.4018
1.8997
1.8851
1.5405
1.1010
1.985
0.931
2.000
2.040
2.086
3.136
ppm
N
N
S
S
N
O
NO2

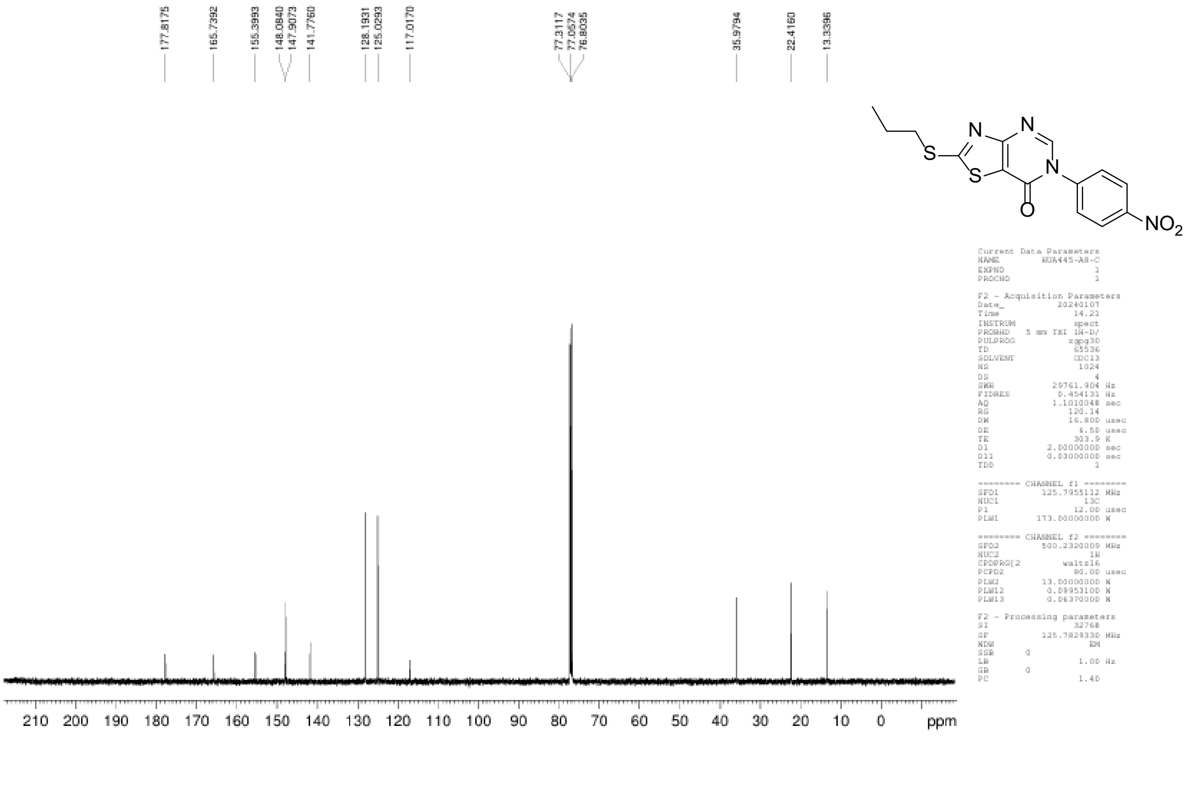
177.8175
165.7392
155.3993
148.0840
147.9073
141.7760
128.1931
125.0293
117.0170
77.3117
77.0574
76.8035
35.9794
22.4160
13.3396
ppm
N
N
S
S
N
O
NO2

1dai

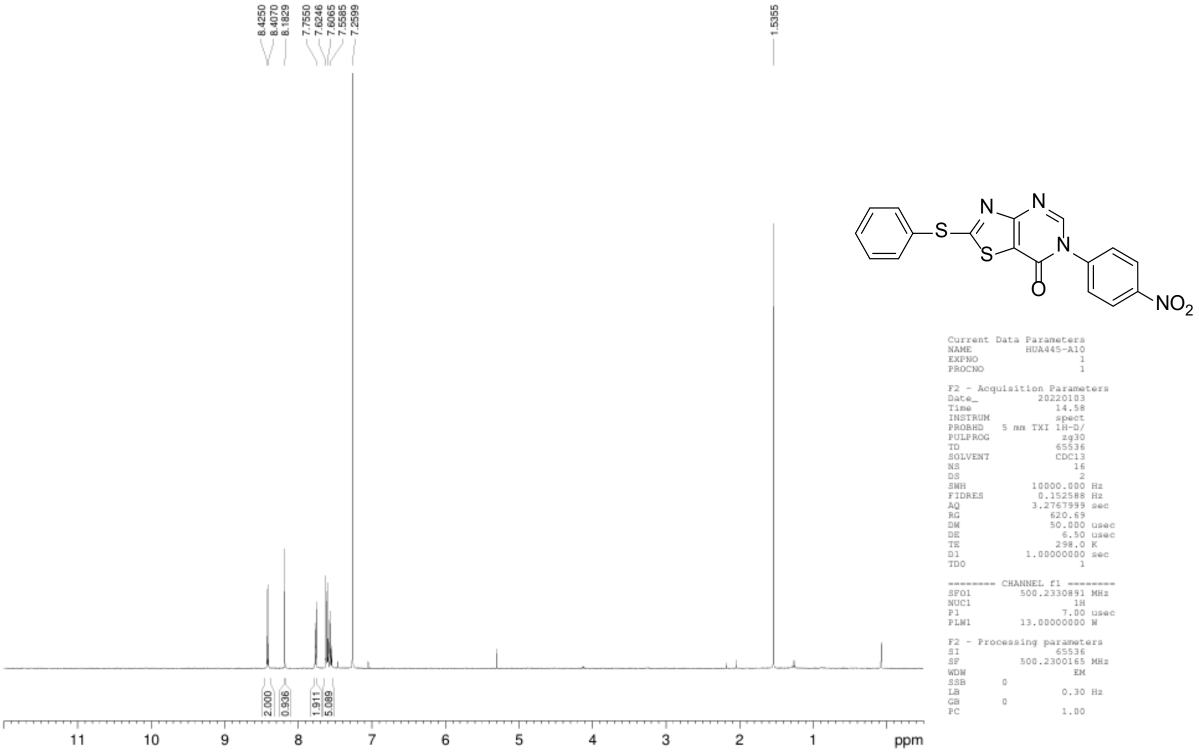
8.4250
8.4070
8.1829
7.7550
7.6246
7.6065
7.5585
7.2599
1.5355
N
N
S
S
N
O
NO2
Current Data Parameters
NAME HUA445-A10
EXPNO 1
PROCNO 1
F2 - Acquisition Parameters
Date_ 20220103
Time 14.58
INSTRUM spect
PROBHD 5 mm TXI 1H-D/
PULPROG zg30
TD 65536
SOLVENT CDCl3
NS 16
DS 2
SWH 10000.000 Hz
FIDRES 0.152588 Hz
AQ 3.2767999 sec
RG 620.69
DW 50.000 usec
DE 6.50 usec
TE 298.0 K
D1 1.00000000 sec
TD0 1
======== CHANNEL f1 ========
SFO1 500.2330891 MHz
NUC1 1H
P1 7.00 usec
PLW1 13.00000000 W
F2 - Processing parameters
SI 65536
SF 500.2300165 MHz
WDW EM
SSB 0
LB 0.30 Hz
GB 0
PC 1.00
2.000
0.936
1.911
5.089
11 10 9 8 7 6 5 4 3 2 1 ppm

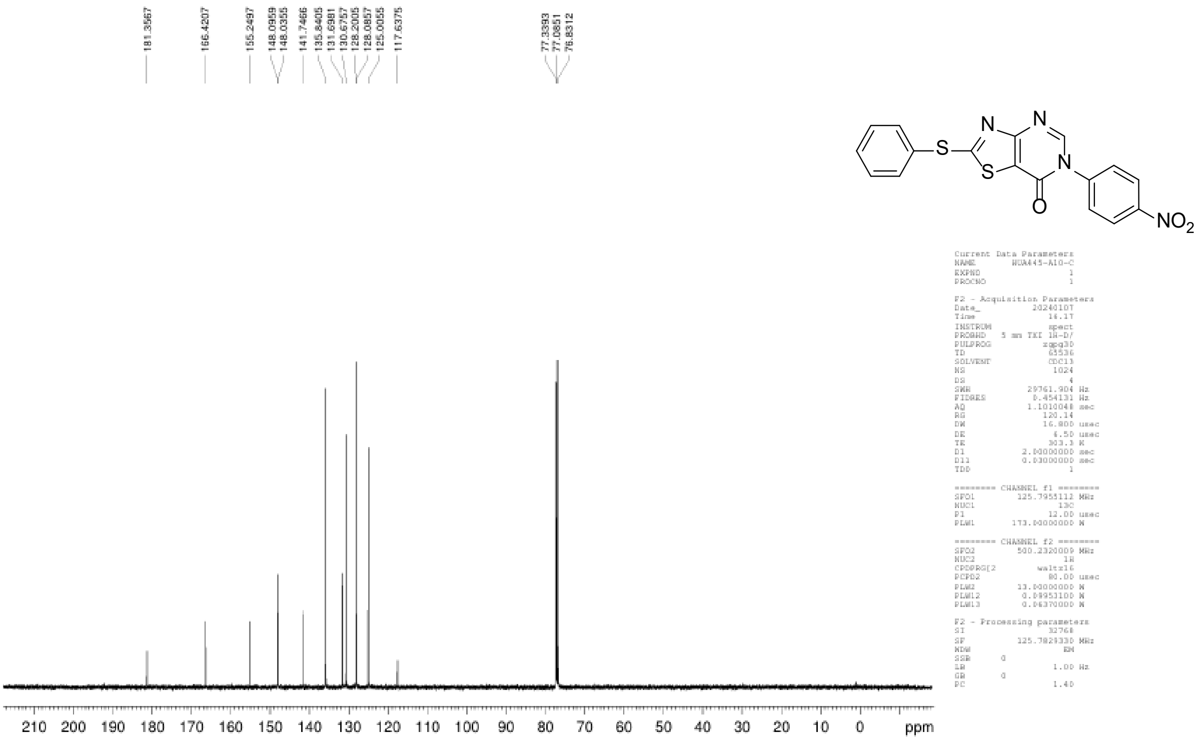
181.3567
166.4207
155.2497
148.0959
148.0355
141.7466
135.8405
131.6981
130.6757
128.2005
128.0857
125.0055
117.6375
77.3393
77.0851
76.8312
N
N
S
S
N
O
NO2
Current Data Parameters
NAME HUA445-A10-C
EXPNO 1
PROCNO 1
F2 - Acquisition Parameters
Date_ 20240107
Time 16.17
INSTRUM spect
PROBHD 5 mm TXI 1H-D/
PULPROG zgpg30
TD 65536
SOLVENT CDCl3
NS 1024
DS 4
SWH 29761.904 Hz
FIDRES 0.454131 Hz
AQ 1.1010048 sec
RG 120.14
DW 16.800 usec
DE 6.50 usec
TE 303.3 K
D1 2.00000000 sec
D11 0.03000000 sec
TD0 1
======== CHANNEL f1 ========
SFO1 125.7955112 MHz
NUC1 13C
P1 12.00 usec
PLW1 173.00000000 W
======== CHANNEL f2 ========
SFO2 500.2320009 MHz
NUC2 1H
CPDPRG[2 waltz16
PCPD2 80.00 usec
PLW2 13.00000000 W
PLW12 0.09953100 W
PLW13 0.06370000 W
F2 - Processing parameters
SI 32768
SF 125.7828330 MHz
WDW EM
SSB 0
LB 1.00 Hz
GB 0
PC 1.40
210 200 190 180 170 160 150 140 130 120 110 100 90 80 70 60 50 40 30 20 10 0 ppm

1daj

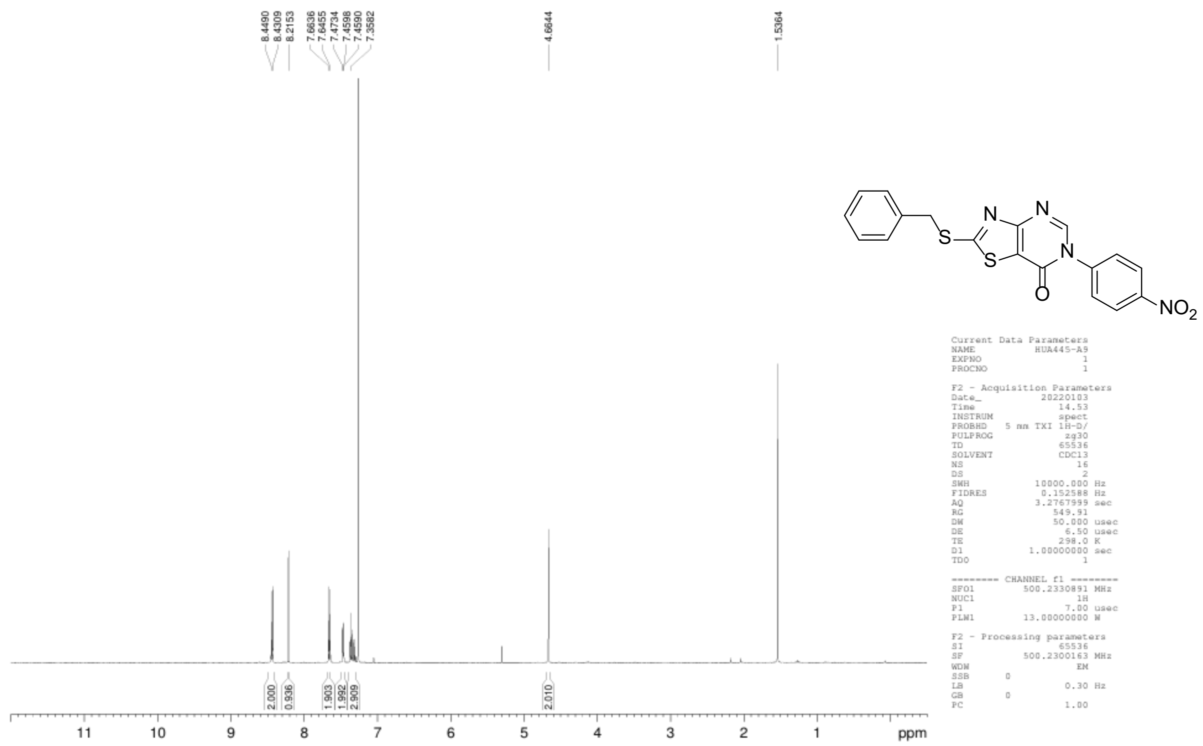

8.4490
8.4309
8.2153
7.6636
7.6455
7.4734
7.4598
7.4590
7.3582
4.6644
1.5364
N
N
S
S
N
O
NO2
Current Data Parameters
NAME HUA445-A9
EXPNO 1
PROCNO 1
F2 - Acquisition Parameters
Date_ 20220103
Time 14.53
INSTRUM spect
PROBHD 5 mm TXI 1H-D/
PULPROG zg30
TD 65536
SOLVENT CDCl3
NS 16
DS 2
SWH 10000.000 Hz
FIDRES 0.152588 Hz
AQ 3.2767999 sec
RG 549.91
DW 50.000 usec
DE 6.50 usec
TE 298.0 K
D1 1.00000000 sec
TD0 1
======== CHANNEL f1 ========
SFO1 500.2330891 MHz
NUC1 1H
P1 7.00 usec
PLW1 13.00000000 W
F2 - Processing parameters
SI 65536
SF 500.2300163 MHz
WDW EM
SSB 0
LB 0.30 Hz
GB 0
PC 1.00
2.000
0.936
1.903
1.992
2.909
2.010
11 10 9 8 7 6 5 4 3 2 1 ppm

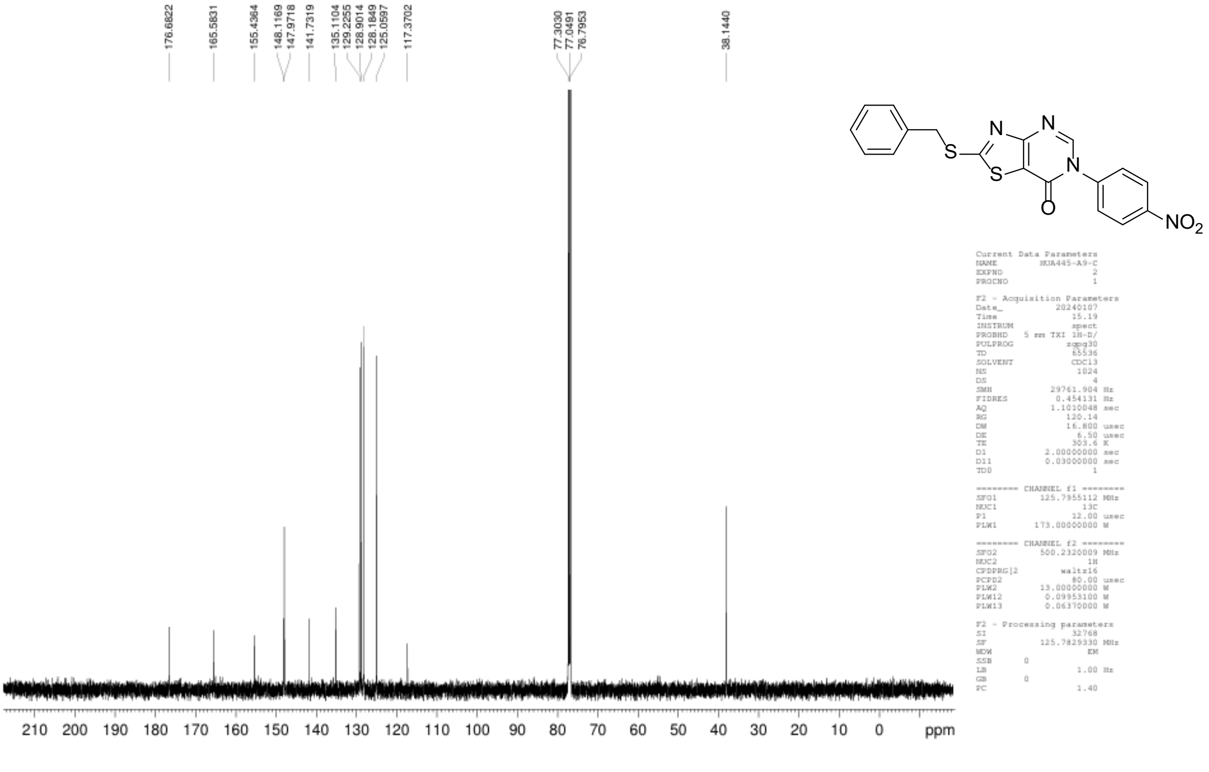

176.6822
165.5831
155.4364
148.1169
147.9718
141.7319
135.1104
129.2255
128.9014
128.1849
125.0597
117.3702
77.3030
77.0491
76.7953
38.1440
N
N
S
S
N
O
NO2
Current Data Parameters
NAME HUA445-A9-C
EXPNO 2
PROCNO 1
F2 - Acquisition Parameters
Date_ 20240107
Time 15.19
INSTRUM spect
PROBHD 5 mm TXI 1H-D/
PULPROG zgpg30
TD 65536
SOLVENT CDCl3
NS 1024
DS 4
SWH 29761.904 Hz
FIDRES 0.454131 Hz
AQ 1.1010048 sec
RG 120.14
DW 16.800 usec
DE 6.50 usec
TE 303.6 K
D1 2.00000000 sec
D11 0.03000000 sec
TD0 1
======== CHANNEL f1 ========
SFO1 125.7955112 MHz
NUC1 13C
P1 12.00 usec
PLW1 173.00000000 W
======== CHANNEL f2 ========
SFO2 500.2320009 MHz
NUC2 1H
CPDPRG[2 waltz16
PCPD2 80.00 usec
PLW2 13.00000000 W
PLW12 0.09953100 W
PLW13 0.06370000 W
F2 - Processing parameters
SI 32768
SF 125.7829330 MHz
WDW EM
SSB 0
LB 1.00 Hz
GB 0
PC 1.40
210 200 190 180 170 160 150 140 130 120 110 100 90 80 70 60 50 40 30 20 10 0 ppm

1dba

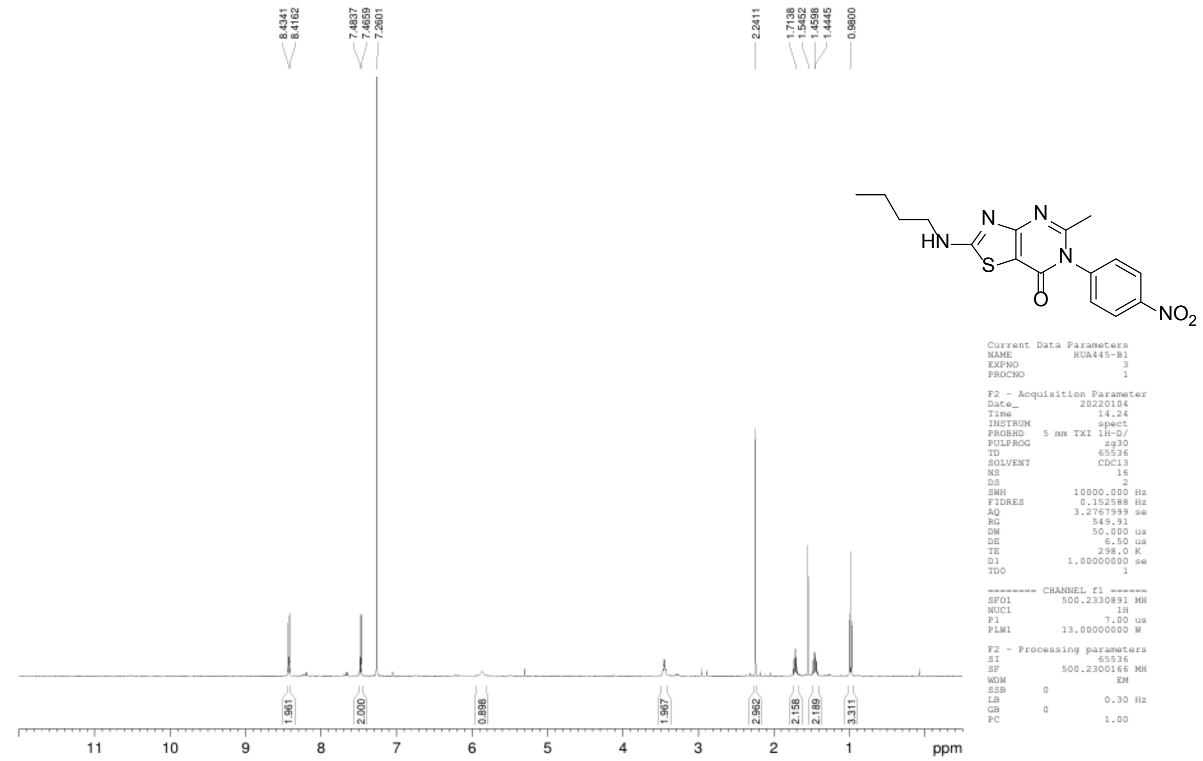
8.4341
8.4162
7.4837
7.4659
7.2601
2.2411
1.7138
1.5452
1.4598
1.4445
0.9800
HN
N
N
S
N
O
NO_2
Current Data Parameters
NAME HUA445-B1
EXPNO 3
PROCNO 1
F2 - Acquisition Parameter
Date_ 20220104
Time 14.24
INSTRUM spect
PROBHD 5 mm TXI 1H-D/
PULPROG zg30
TD 65536
SOLVENT CDCl3
NS 16
DS 2
SWH 10000.000 Hz
FIDRES 0.152588 Hz
AQ 3.2767999 se
RG 549.91
DW 50.000 us
DE 6.50 us
TE 298.0 K
D1 1.00000000 se
TD0 1
======== CHANNEL f1 ======
SFO1 500.2330891 MH
NUC1 1H
P1 7.00 us
PLW1 13.00000000 W
F2 - Processing parameters
SI 65536
SF 500.2300166 MH
WDW EM
SSB 0
LB 0.30 Hz
GB 0
PC 1.00
1.961
2.000
0.898
1.967
2.962
2.158
2.189
3.311
11 10 9 8 7 6 5 4 3 2 1 ppm

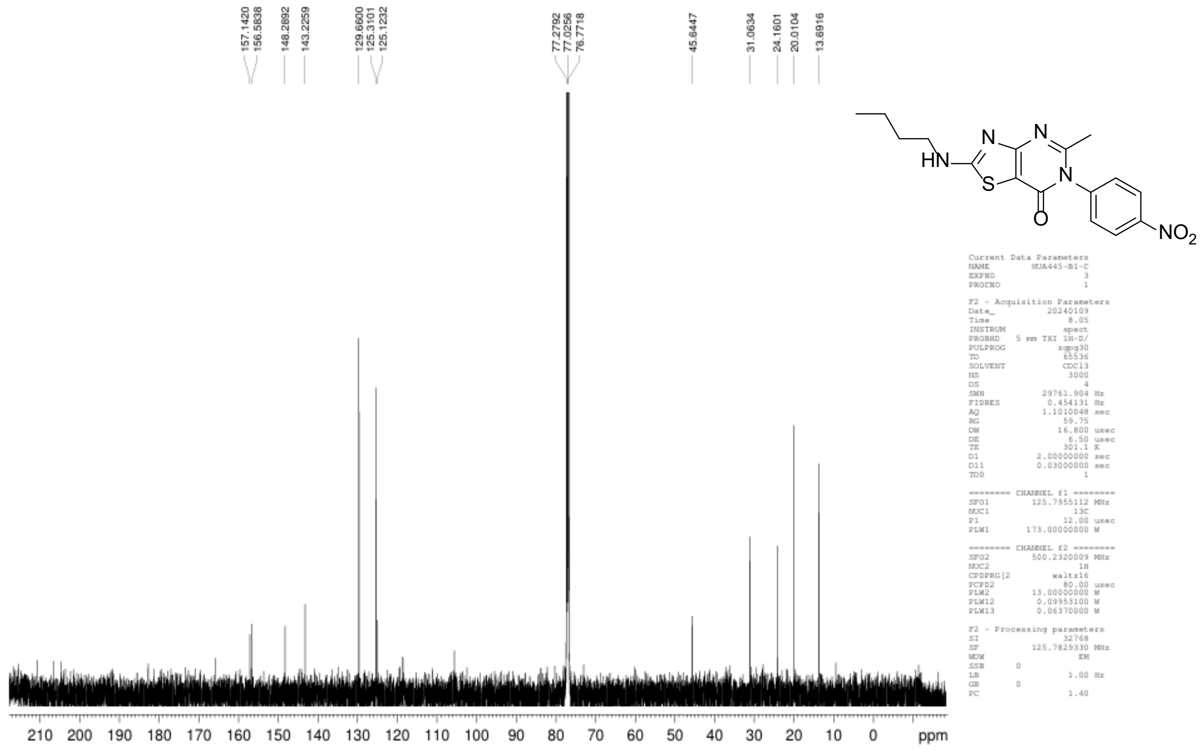
157.1420
156.5838
148.2892
143.2259
129.6600
125.3101
125.1232
77.2792
77.0256
76.7718
45.6447
31.0634
24.1601
20.0104
13.6916
HN
N
N
S
N
O
NO_2
Current Data Parameters
NAME HUA445-B1-C
EXPNO 3
PROCNO 1
F2 - Acquisition Parameters
Date_ 20240109
Time 8.05
INSTRUM spect
PROBHD 5 mm TXI 1H-D/
PULPROG zgpg30
TD 65536
SOLVENT CDCl3
NS 3000
DS 4
SWH 29761.904 Hz
FIDRES 0.454131 Hz
AQ 1.1010048 sec
RG 59.75
DW 16.800 usec
DE 6.50 usec
TE 301.1 K
D1 2.00000000 sec
D11 0.03000000 sec
TD0 1
======== CHANNEL f1 ========
SFO1 125.7955112 MHz
NUC1 13C
P1 12.00 usec
PLW1 173.00000000 W
======== CHANNEL f2 ========
SFO2 500.2320009 MHz
NUC2 1H
CPDPRG[2 waltz16
PCPD2 80.00 usec
PLW2 13.00000000 W
PLW12 0.09953100 W
PLW13 0.06370000 W
F2 - Processing parameters
SI 32768
SF 125.7829330 MHz
WDW EM
SSB 0
LB 1.00 Hz
GB 0
PC 1.40
210 200 190 180 170 160 150 140 130 120 110 100 90 80 70 60 50 40 30 20 10 0 ppm

1dbb

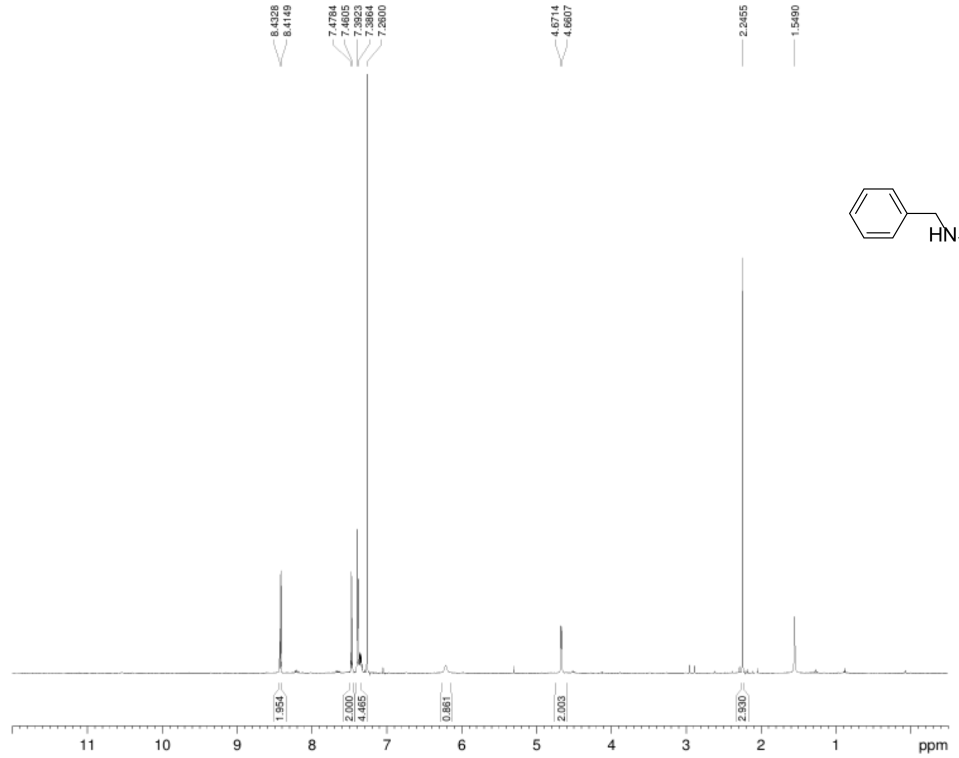

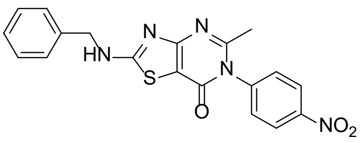

Current Data Parameters
NAME HUA445-B4
EXPNO 1
PROCNO 1

F2 - Acquisition Parameters
Date_ 20220104
Time 14.36
INSTRUM spect
PROBHD 5 mm TXI 1H-D/
PULPROG zg30
TD 65536
SOLVENT CDCl3
NS 16
DS 2
SWH 10000.000 Hz
FIDRES 0.152588 Hz
AQ 3.2767999 sec
RG 488.68
DW 50.000 usec
DE 6.50 usec
TE 298.0 K
D1 1.00000000 sec
TD0 1

======== CHANNEL f1 ========
SFO1 500.2330891 MHz
NUC1 1H
P1 7.00 usec
PLW1 13.00000000 W

F2 - Processing parameters
SI 65536
SF 500.2300167 MHz
WDW EM
SSB 0
LB 0.30 Hz
GB 0
PC 1.00

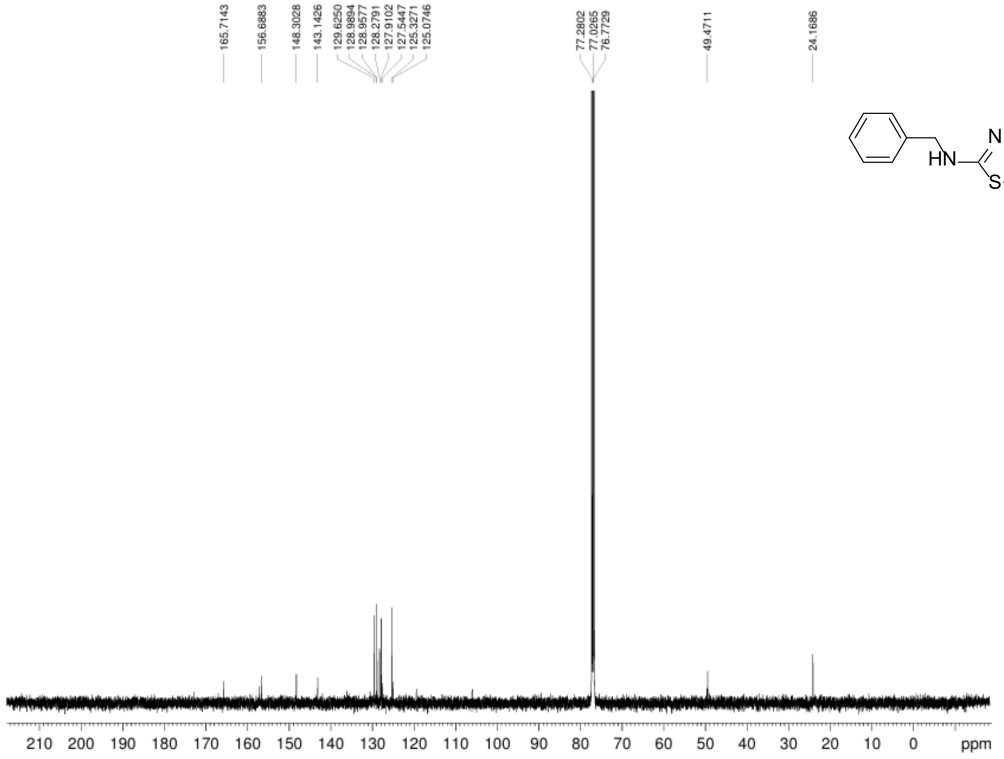

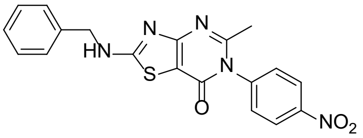

Current Data Parameters
NAME HUA445-B4-C
EXPNO 2
PROCNO 1

F2 - Acquisition Paramete
Date_ 20240109
Time 22.36
INSTRUM spect
PROBHD 5 mm TXI 1H-D/
PULPROG zgpg30
TD 65536
SOLVENT CDCl3
NS 4000
DS 4
SWH 29761.904 H
FIDRES 0.454131 H
AQ 1.1010048 s
RG 120.14
DW 16.800 u
DE 6.50 u
TE 0 K
D1 2.00000000 s
D11 0.03000000 s
TD0 1

======== CHANNEL f1 =====
SFO1 125.7955112 M
NUC1 13C
P1 12.00 u
PLW1 173.00000000 W

======== CHANNEL f2 =====
SFO2 500.2320009 M
NUC2 1H
CPDPRG[2 waltz16
PCPD2 80.00 u
PLW2 13.00000000 W
PLW12 0.09953100 W
PLW13 0.06370000 W

F2 - Processing parameter
SI 32768
SF 125.7829330 M
WDW EM
SSB 0
LB 1.00 H
GB 0
PC 1.40

1dbd

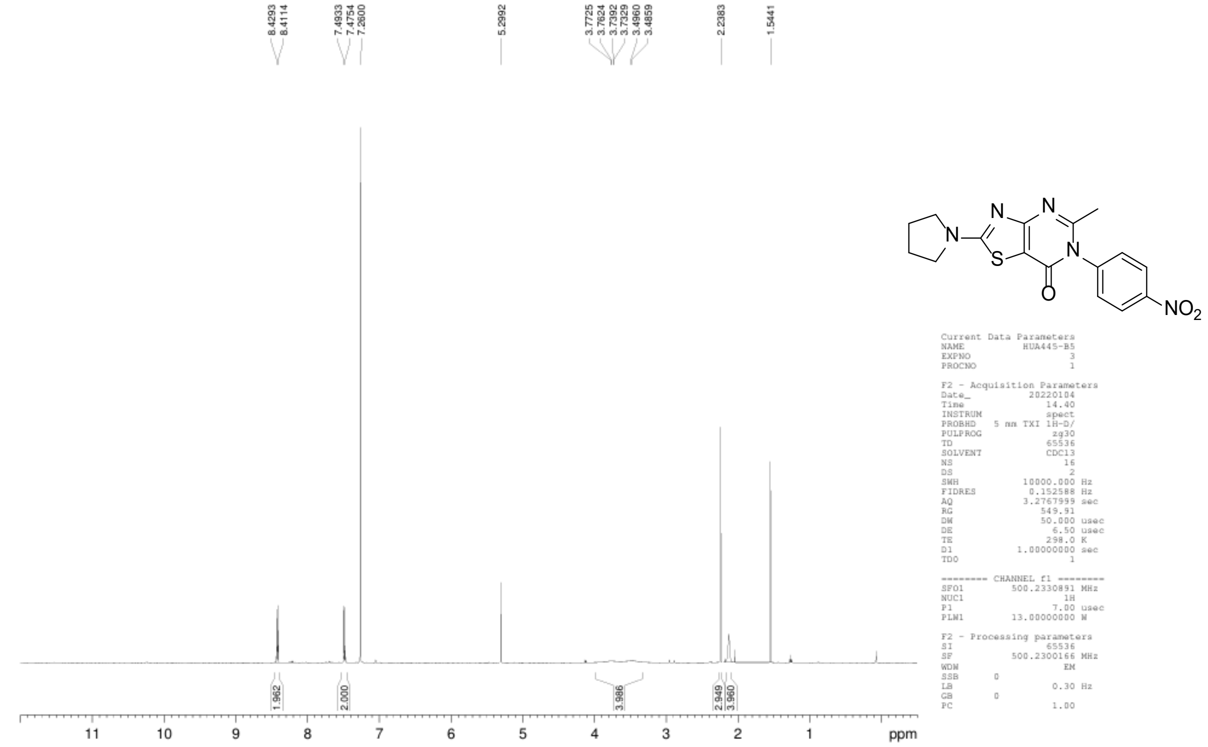
8.4293
8.4114
7.4933
7.4754
7.2600
5.2992
3.7725
3.7624
3.7392
3.7329
3.4960
3.4859
2.2383
1.5441
1.962
2.000
3.986
2.949
3.960
11 10 9 8 7 6 5 4 3 2 1 ppm
N
N
N
S
N
O
NO2
Current Data Parameters
NAME HUA445-B5
EXPNO 3
PROCNO 1
F2 - Acquisition Parameters
Date_ 20220104
Time 14.40
INSTRUM spect
PROBHD 5 mm TXI 1H-D/
PULPROG zg30
TD 65536
SOLVENT CDCl3
NS 16
DS 2
SWH 10000.000 Hz
FIDRES 0.152588 Hz
AQ 3.2767999 sec
RG 549.91
DW 50.000 usec
DE 6.50 usec
TE 298.0 K
D1 1.00000000 sec
TD0 1
======== CHANNEL f1 ========
SFO1 500.2330891 MHz
NUC1 1H
P1 7.00 usec
PLW1 13.00000000 W
F2 - Processing parameters
SI 65536
SF 500.2300166 MHz
WDW EM
SSB 0
LB 0.30 Hz
GB 0
PC 1.00

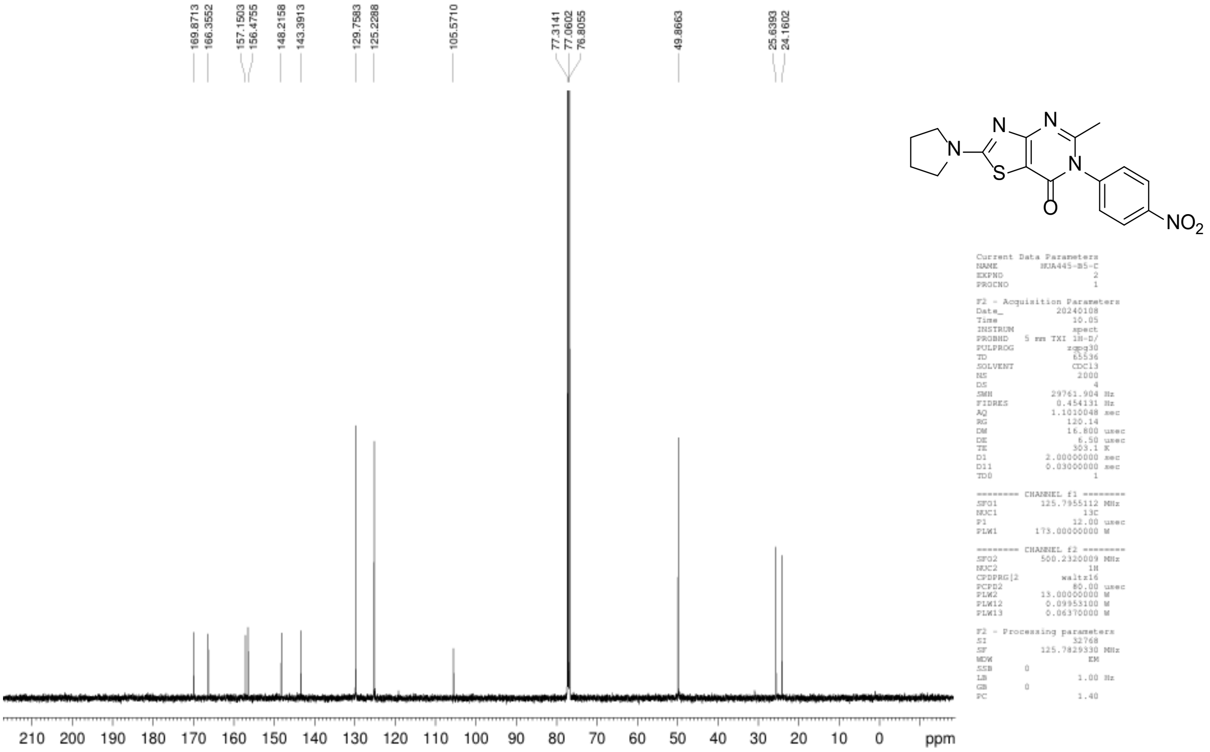
169.8713
166.3552
157.1503
156.4755
148.2158
143.3913
129.7583
125.2288
105.5710
77.3141
77.0602
76.8055
49.8663
25.6393
24.1602
210 200 190 180 170 160 150 140 130 120 110 100 90 80 70 60 50 40 30 20 10 0 ppm
N
N
N
S
N
O
NO2
Current Data Parameters
NAME HUA445-B5-C
EXPNO 2
PROCNO 1
F2 - Acquisition Parameters
Date_ 20240108
Time 10.05
INSTRUM spect
PROBHD 5 mm TXI 1H-D/
PULPROG zgpg30
TD 65536
SOLVENT CDCl3
NS 2000
DS 4
SWH 29761.904 Hz
FIDRES 0.454131 Hz
AQ 1.1010048 sec
RG 120.14
DW 16.800 usec
DE 6.50 usec
TE 303.1 K
D1 2.00000000 sec
D11 0.03000000 sec
TD0 1
======== CHANNEL f1 ========
SFO1 125.7955112 MHz
NUC1 13C
P1 12.00 usec
PLW1 173.00000000 W
======== CHANNEL f2 ========
SFO2 500.2320009 MHz
NUC2 1H
CPDPRG[2 waltz16
PCPD2 80.00 usec
PLW2 13.00000000 W
PLW12 0.09953100 W
PLW13 0.06370000 W
F2 - Processing parameters
SI 32768
SF 125.7829330 MHz
WDW EM
SSB 0
LB 1.00 Hz
GB 0
PC 1.40

1dbe

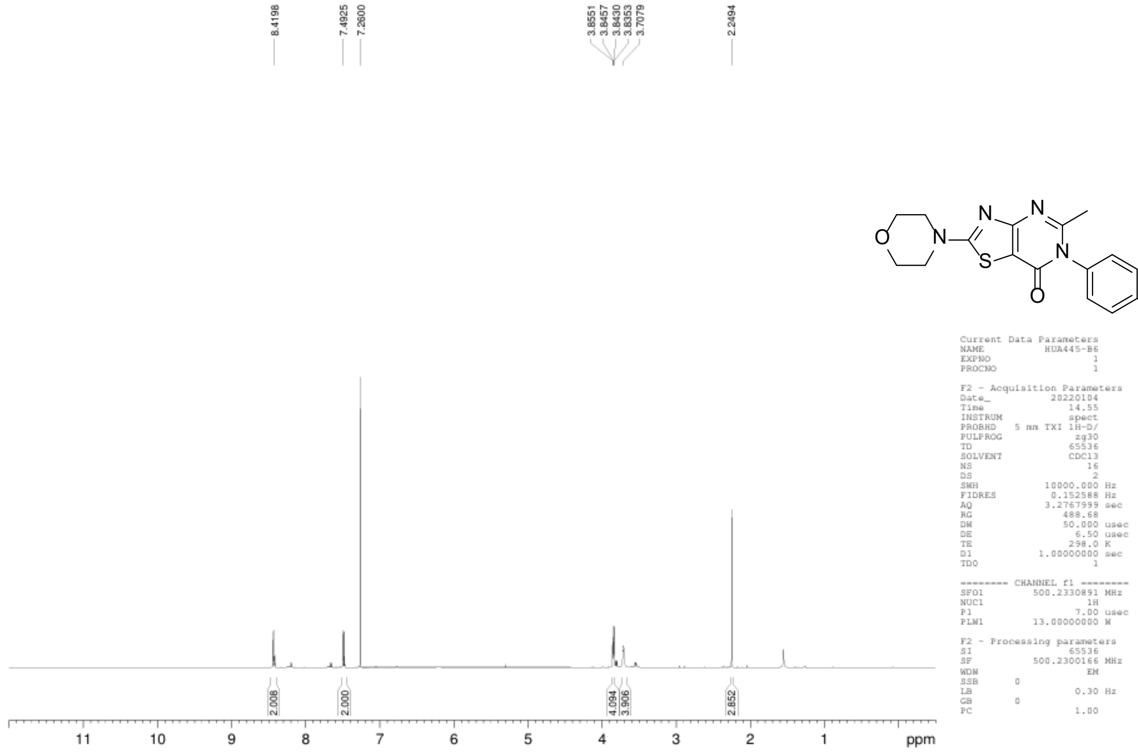
8.4198
7.4925
7.2600
3.8551
3.8457
3.8430
3.8353
3.7079
2.2494
N
N
O
N
S
N
O
NO2
2.008
2.000
4.094
3.906
2.852
11 10 9 8 7 6 5 4 3 2 1 ppm
Current Data Parameters
NAME HUA445-B6
EXPNO 1
PROCNO 1
F2 - Acquisition Parameters
Date_ 20220104
Time 14.55
INSTRUM spect
PROBHD 5 mm TXI 1H-D/
PULPROG zg30
TD 65536
SOLVENT CDCl3
NS 16
DS 2
SWH 10000.000 Hz
FIDRES 0.152588 Hz
AQ 3.2767999 sec
RG 488.68
DW 50.000 usec
DE 6.50 usec
TE 298.0 K
D1 1.00000000 sec
TD0 1
======== CHANNEL f1 ========
SFO1 500.2330891 MHz
NUC1 1H
P1 7.00 usec
PLW1 13.00000000 W
F2 - Processing parameters
SI 65536
SF 500.2300166 MHz
WDW EM
SSB 0
LB 0.30 Hz
GB 0
PC 1.00

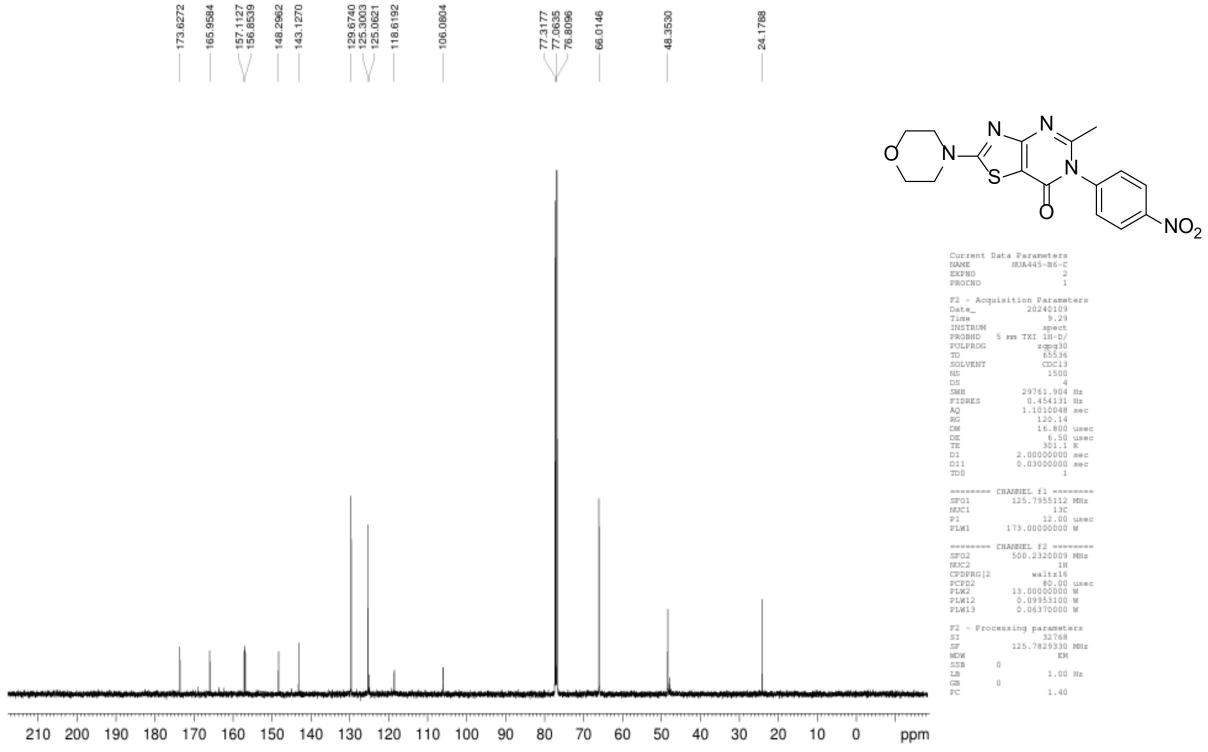
173.6272
165.9584
157.1127
156.8539
148.2962
143.1270
129.6740
125.3003
125.0621
118.6192
106.0804
77.3177
77.0635
76.8096
66.0146
48.3530
24.1788
N
N
O
N
S
N
O
NO2
210 200 190 180 170 160 150 140 130 120 110 100 90 80 70 60 50 40 30 20 10 0 ppm

1dbj

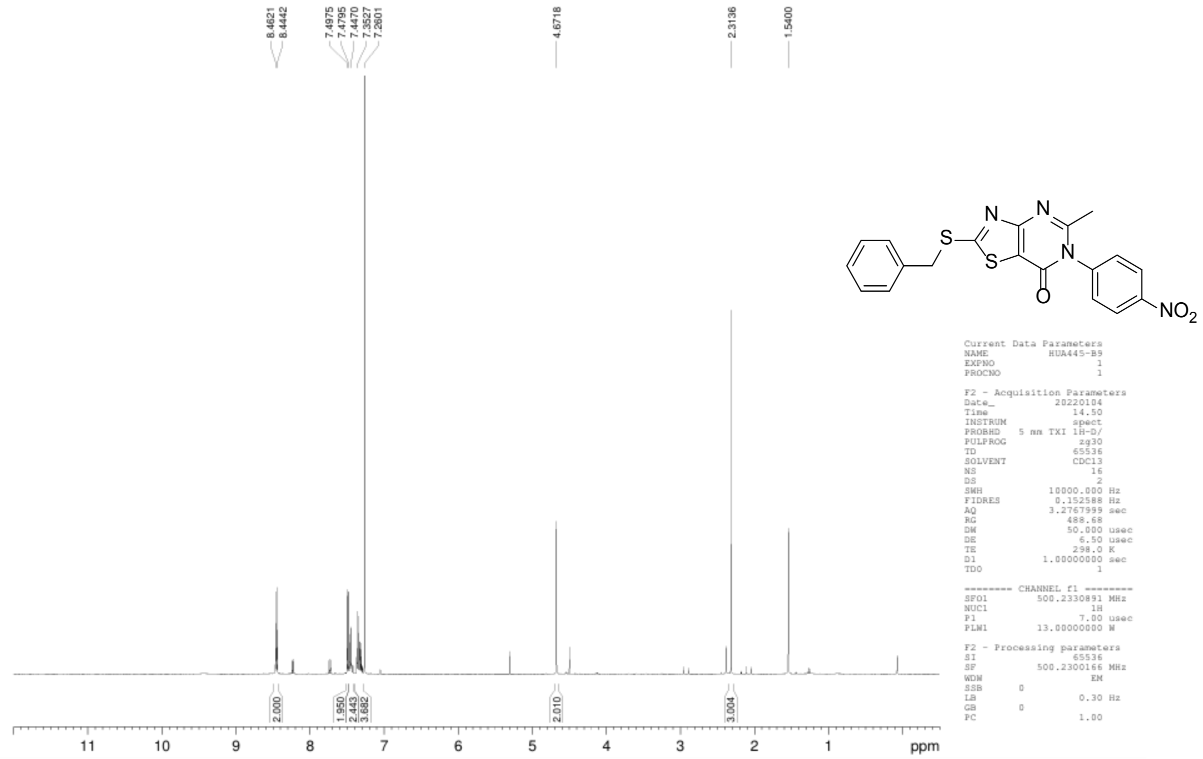

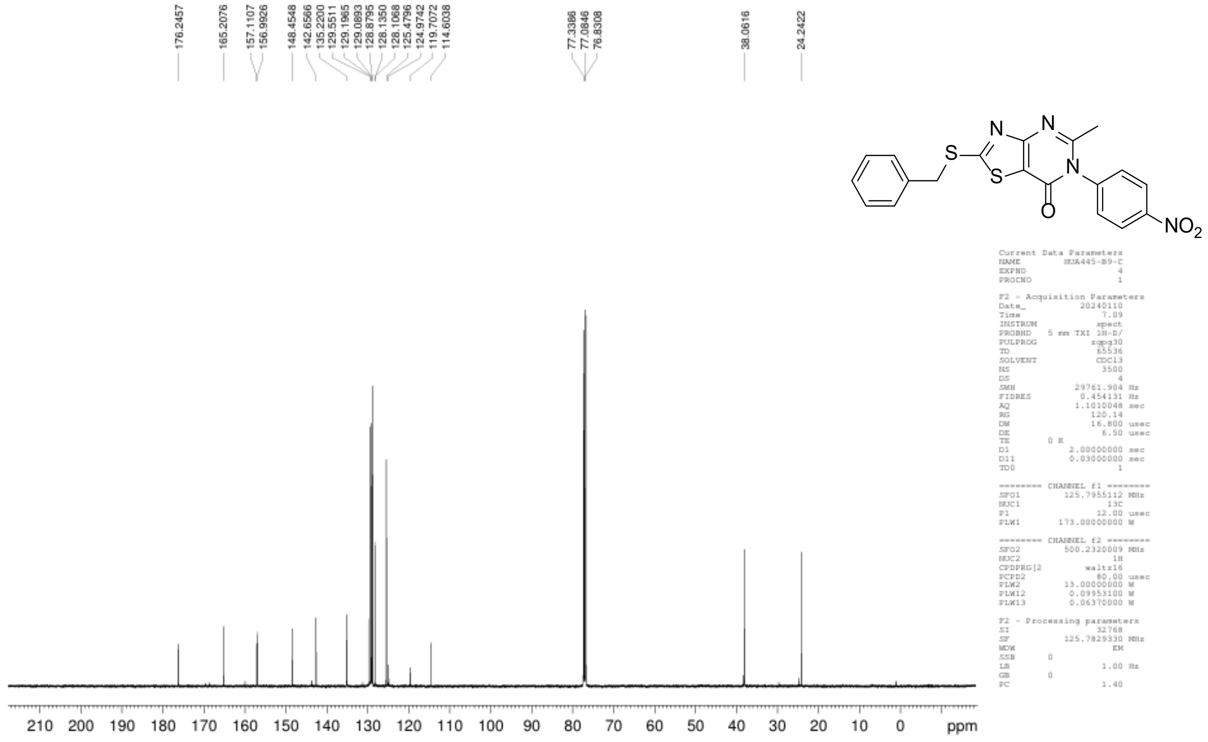

Supplement: Supplementary file 1 [file molecules-30-00430-s001.zip › molecules-3407108-supplementary.pdf]
